# Andrographolide contributes to spinal cord injury repair *via* inhibition of apoptosis, oxidative stress and inflammation

**DOI:** 10.3389/fphar.2022.949502

**Published:** 2022-10-07

**Authors:** Zhen Li, Zehui Li, Zhenyue Chen, He Sun, Zhagen Yuan, Xiaochao Wang, Jinqiang Wei, Xuewei Cao, Decai Zheng

**Affiliations:** ^1^ The Second Clinical College of Guangzhou University of Chinese Medicine, Guangzhou, Guangdong, China; ^2^ Guangdong Provincial Hospital of Chinese Medicine, The Second Affiliated Hospital of Guangzhou University of Chinese Medicine, Guangzhou, Guangdong, China; ^3^ The First Clinical College of Guangzhou University of Chinese Medicine, Guangzhou, Guangdong, China; ^4^ Lingnan Medical Research Center of Guangzhou University of Chinese Medicine, Guangzhou, Guangdong, China

**Keywords:** andrographolide, spinal cord injury, apoptosis, oxidative stress, inflammation

## Abstract

**Background:** Spinal cord injury (SCI) is a common disorder of the central nervous system with considerable socio-economic burden. Andrographolide (Andro), the main active component of *Andrographis paniculata*, has exhibited neuroprotective effects in different models of neurological diseases. The aim of this study was to evaluate the neuroprotective effects of Andro against SCI and explore the related mechanisms.

**Methods:** SCI was induced in rats by the Allen method, and the modeled animals were randomly divided into sham-operated, SCI, SCI + normal saline (NS) and SCI + Andro groups. The rats were injected intraperitoneally with Andro (1 mg/kg) or the same volume of NS starting day one after the establishment of the SCI model for 28 consecutive days. Post-SCI tissue repair and functional recovery were evaluated by measuring the spinal cord water content, footprint tests, Basso-Beattie-Bresnahan (BBB) scores, hematoxylin-eosin (HE) staining and Nissl staining. Apoptosis, oxidative stress and inflammation, as well as axonal regeneration and remyelination were analyzed using suitable markers. The *in vitro* model of SCI was established by treating cortical neurons with H_2_O_2_. The effects of Andro on apoptosis, oxidative stress and inflammation were evaluated as indicated.

**Results:** Andro treatment significantly improved tissue repair and functional recovery after SCI by reducing apoptosis, oxidative stress and inflammation through the nuclear factor E2-related factor 2/heme oxygenase-1 (Nrf-2/HO-1) and nuclear factor-kappa B (NF-κB) signaling pathways. Furthermore, Andro treatment promoted M2 polarization of the microglial cells and contributed to axonal regeneration and remyelination to improve functional recovery after SCI. In addition, Andro also attenuated apoptosis, oxidative stress and inflammation in H_2_O_2_-stimulated cortical neurons *in vitro*.

**Conclusion:** Andro treatment alleviated SCI by reducing apoptosis, oxidative stress and inflammation in the injured tissues and cortical neurons, and promoted axonal regeneration and remyelination for functional recovery.

## Introduction

Spinal cord injury (SCI) refers to the complete or incomplete spinal cord nerve dysfunction involving motor, sensory, reflex and autonomic nerves that is caused by an external mechanical force on the spinal cord (Lee et al., 2014). About 180,000 cases of SCI are reported each year, which place a heavy socioeconomic burden owing to the high disability and mortality rates (Jazayeri et al., 2015; Yang et al., 2016). The pathophysiological process of SCI can be divided into the primary injury and the subsequent secondary injury (Ahuja et al., 2017a; Ahuja et al., 2017b). Primary injury refers to the irreversible mechanical damage to the spinal cord tissue, which causes edema, microcirculation disturbance, thrombosis, vasospasm and tissue hypoxia, leading to secondary injuries such as spinal cord ischemia, hemorrhage and necrosis. Secondary injury is characterized by oxidative stress, inflammation, excitotoxicity, apoptosis and microcirculatory disturbances, which further damage the spinal cord and even lead to paraplegia (Ahuja et al., 2017b; Orr and Gensel, 2018; Wang C et al., 2018). Therefore, alleviating these secondary pathological changes can promote nerve regeneration, and aid in functional recovery after SCI (Amar and Levy, 1999; Kjell and Olson, 2016).

Andrographolide (Andro) is a diterpene lactone extracted from *Andrographis paniculata*, which is used in various traditional Chinese medicine formulations (Dai et al., 2019; Ren et al., 2021; Zeng et al., 2022). Pharmacological studies have shown that Andro has anti-inflammatory, antibacterial, antiviral, immunomodulatory and other biological activities. In addition, Andro exerts neuroprotective effects in Alzheimer’s disease (AD) (Zhang et al., 2021), Parkinson’s disease (PD) (Geng et al., 2019; Ahmed et al., 2021) and chronic cerebral hypoperfusion (CCH) (Wang et al., 2019) by reducing apoptosis, oxidative stress and inflammation through multiple signaling pathways. Patel et al. (2021) showed that Andro inhibited the expression of AchE, Aβ_1-42_ and *p*-tau in an ICV-STZ-induced model of cognitive impairment in rats, which alleviated inflammation and oxidative stress in the affected tissues (Patel et al., 2021). In the CCH rat model, Andro suppressed neuronal apoptosis and neuroinflammation by activating the hippocampal BDNF-TrkB signaling pathway (Wang et al., 2019). Furthermore, Ahmed et al. (2021) found that Andro rescued dopaminergic neuron loss in a mouse model of PD by inhibiting the activation of NLR Family Pyrin Domain Containing 3 (NLRP3) inflammasome in the microglia. However, little is known regarding the possible neuroprotective effects of Andro in SCI. To this end, the aim of this study was to evaluate the effects of Andro on neuronal apoptosis, oxidative stress and inflammation in a rat model of SCI, and explore the mechanisms underlying functional recovery.

## Materials and methods

### Isolation and culture of cortical neurons

Cortical neurons were extracted from the embryos of pregnant SD rats as described previously (Hou et al., 2021). Briefly, after digesting the cortical tissues with 200 μg/ml papain (Sigma, Beijing, China) for 25 min at 37°C, the homogenate was passed through a 100-μm cell strainer (BD Falcon) and then centrifuged at 800 rpm for 5 min. The supernatant was discarded, and the pellets were resuspended in Dulbecco’s Modified Eagle Medium (DMEM; Gibco, Life Technologies, United States) supplemented with 10% fetal bovine serum (FBS; Gibco, Life Technologies, United States), 100 U/mL penicillin and 100 mg/ml streptomycin (Gibco, Life Technologies, United States). The cells were seeded in poly-d-lysine-coated plates and incubated at 37°C for 4 h under 5% CO_2_ and saturated humidity. After washing once, the cells were cultured in Neurobasal medium (Gibco, Life Technologies, United States) containing 0.5 mM l-glutamine (Gibco, Life Technologies, United States) and 2% B27 (B-27™ Supplement, Gibco). Half of the neurobasal medium was replaced every 2 days. After 7–8 days, cortical neurons were harvested for *in vitro* experiments. The cells were characterized by phase contrast microscopy and by immunostaining for neuron-specific proteins. The purity of the cortical neurons in this primary culture was evaluated to be >90%.

### Viability assay

Cell viability was measured using the Cell Counting Kit-8 (CCK-8, KeyGEN, China) according to the manufacturer’s protocols (Luo et al., 2022). Briefly, cortical neurons were seeded into 96-well plates at the density of 5,000 cells per well and incubated with H_2_O_2_ (300 μM) and different concentrations of Andro (0, 5, 10 and 20 μM) for 24 h. The CCK-8 solution was added to each well, and the cells were incubated further for 2 h. The absorbance of each well at 450 nm (OD_450_) was measured using a multifunctional microplate reader (Bio-Rad).

### Biochemical analysis

Glutathione (GSH) and superoxide dismutase (SOD) activity in the injured spinal cord tissues were measured using specific biochemical kits (Jiancheng Institute of Biology, China) at 7 days post-SCI (Zhao et al., 2021). The levels of interleukin 1β (IL-1β) and tumor necrotic factor-α (TNF-α) in each sample were measured using ELISA kits (Sigma Aldrich, United States) according to the manufacturer’s instructions at 28 days post-SCI (Zeng et al., 2019).

### Reactive oxygen species generation

The cellular ROS levels were measured using the 2,7-dichlorodihydrofluorescein diacetate (2,7-DCF-DA) staining kit (Sigma Aldrich) according to the manufacturer’s protocols (Li et al., 2019). The suitably treated cells were washed twice with PBS, and incubated with 10 μM DCFH-DA fluorescent probe at 37°C for 30 min. The stained cells were observed under a fluorescence microscope (Olympus IX73).

### Establishment of SCI model and treatment

Forty-eight 8- and 12-weeks-old specific pathogen-free (SPF) male Sprague-Dawley (SD) rats (200–250 g) were supplied by the Experimental Animal Center of Guangzhou University of Chinese Medicine. The animals were housed in cages under a 12-h light/dark cycle and fed adaptively for approximately 1 week. All animal experiments met ethical standards and underwent ethical review. The SCI model was constructed according to the Allen method (Zhan et al., 2020). The rats were anesthetized and their dorsum was depilated, and a 2 cm midline incision was made at the T10 level. Laminectomy was performed after separating the muscle from the pedicle and stripping the T10 spinous process. A longitudinal midline incision measuring approximately 0.5 cm was then made to expose the T10 spinal cord, which was struck with a pneumatic vertical device to simulate SCI. The impact speed was set to 1.2 m/s, the impact depth to 1 mm, and the rest time to 85 ms. Finally, the wound was sutured and the body temperature of rats was maintained at 37 ± 0.5°C using an electric blanket. Urination was facilitated twice a day thereafter by squeezing the bladder. The sham-operated group (controls) only underwent the incision without SCI impact.

Andro (≥98% purity) was purchased from Sigma-Aldrich Chemical Corporation (St Louis, MO, United States) and dissolved in 1% dimethyl sulfoxide. The animals were randomly divided into the sham-operated, SCI, SCI + normal saline and SCI + Andro groups (*n* = 12 each). The rats in the SCI + Andro group were intraperitoneally injected with 1 mg/2 ml/kg Andro once daily starting day 1 after SCI induction for 28 days. In the SCI + NS group, the animals received equal volume of normal saline. The other groups did not receive any drugs or saline.

### Water content of the spinal cord

Spinal cord edema was evaluated by the wet/dry method 28 days post-SCI (Luo et al., 2022). Briefly, 1 cm-long pieces were cut from the injured site and weighed. The tissues were then dried in an oven at 60°C for 48 h, and the dehydrated specimens were weighed. The water content of the spinal cord was calculated as (wet weight-dry weight)/wet weight.

### Behavioral assessment

To analyze the gait of the modeled rats, the animals were encouraged to walk straight through a narrow, white-paper-covered path after immersing their hind feet in ink, and the footprints were recorded (Hou et al., 2021). The footprint test was performed on day 28 post-SCI. Hindlimb motor function was analyzed on days 1, 7, 14, 21 and 28 post-SCI by the Basso, Beattie and Bresnahan (BBB) locomotor test as previously described (Basso et al., 1995). The test was scored across a scale of 0–21, with 0 indicating no significant movement of the hind limbs and 21 indicating normal movement. The footprint and BBB tests were conducted by investigators blinded to the experimental grouping.

### Histological assessment

The animals were euthanized 28 days post-SCI, and their spinal cord tissues were removed under anesthesia by transcardial perfusion with 0.9% saline and 4% paraformaldehyde (PFA). After fixing with 4% PFA, the tissues were dehydrated through an ethanol gradient (70%, 80%, 95%–100%) and embedded in paraffin. The wax block was sliced into 5 µm-thick sections that were baked overnight in an oven at 37°C. Hematoxylin-eosin (HE) staining was performed as per standard protocols, and the inflammation around the lesions was scored. To determine the neuron survival, slides from three animals per group were used for Nissl staining, and the number of positively stained cells was counted in a blinded manner.

### Western blotting

Total protein was extracted from the tissues by sonicating with the radio immunoprecipitation assay (RIPA) lysis buffer (Gibco, Grand Island, NY, United States), and centrifuging twice at 12,000 g for 10 min at 4°C. The supernatant was extracted and the protein was measured using a bicinchoninic acid (BCA) kit (Bio-Rad Laboratories, CA, United States). Twenty micrograms protein per sample was diluted in the loading buffer and denatured at 99°C for 10 min. The protein samples were separated by 10% SDS-PAGE and transferred to polyvinylidene difluoride (PVDF) membranes. After blocking with 5% skimmed milk, the blots were incubated overnight with primary antibodies targeting Bcl-2, Bax, cleaved caspase-3, nuclear factor E2-related factor 2 (Nrf-2), heme oxygenase-1 (HO-1), p-IκBα, IκBα, p-NF-κB, nuclear factor-kappa B (NF-κB), glial fibrillary acidic protein (GFAP), growth-associated protein 43 (GAP43), myelin basic protein (MBP),Interleukin-1β(IL-1β), tumor necrosis factor-α (TNF-α) and GAPDH at 4°C. All primary antibodies were from Abcam and used at 1:1000 dilution. The membranes were washed in Tris buffer solution (TBST) and incubated with secondary antibody (1:1000) for 90 min at room temperature (RT). The positive bands were visualized using a ChemiDoc™ MP Imaging System (Bio-Rad), and the relative intensities of each band were determined using ImageJ (National Institutes of Health, Bethesda,MD). GAPDH was used as the internal reference.

### Quantitative real-time polymerase chain reaction

Total RNA was extracted from rat cortical neurons using an RNAiso Plus Kit, and reverse transcribed using the PrimeScriptRT kit (Takara Biotechnology, Dalian,China). The SYBR^®^ Green Premix *Pro Taq* HS qPCR kit Ⅱ (Accurate Biotechnology, Guangzhou, China) was used for qPCR analyses. The reaction mixture consisting of 2 µL cDNA template, 10 µL 2xSYBR^®^ Green *Pro Taq* HS Premix Ⅱ, 0.8 µL each forward and reverse primer (10 µM) and 20 µL RNase free water. The following two-step PCR reaction program was used: Step 1–30 s pre-denaturation at 95°C; Step 2–5 s denaturation at 95°C and 20 s annealing for 40 cycles, and extension at 60 °C. The expression of IL-1β and TNF-α were evaluated using the 2^−ΔΔCt^ method with U6 as the internal standard. Primer sequences are listed in Table 1.

**TABLE 1:** Primer sequences for qRT-PCR.

Gene	Primer sequence
IL-1β	F:5′-GGCTGCTCTGGGATTCTCTT-3′
	R:5′-ATTTCACTGGCGAGCTCAGG-3′
TNF-α	F:5′-TGGGATCATTGCCCTGTGAG-3′
	R:5′-GGTGTCTGAAGGAGGGGGTA-3′
GAPDH	F:5′-ACCCAGAAGACTGTGGATGG-3′
	R:5′-GAGGCAGGGATGATGTTCTG-3′

F, forward; R, reverse.

### Immunofluorescence staining

The spinal cord tissue sections were treated with xylene and graded alcohol, and heated in citric acid for antigen retrieval. After blocking with 10% fetal bovine serum (FBS; Gibco, Life Technologies, United States) for 1 h at RT, the sections were incubated overnight with primary antibodies against ionized calcium binding adaptor molecule 1 (Iba-1, 1:200, and CST), CD68 (1:300, Boster Biological Engineering Co.), CD163 (1:30, Santa Cruz), GFAP (1:600, Abcam), GAP43 (1:200, Abcam) and MBP (1:200, Abcam) at 4°C. The following day, the sections were incubated with fluorochrome-conjugate rabbit or mouse secondary antibodies (1:300, Invitrogen), and mounted with DAPI medium (Solarbio). The sections were observed under a fluorescence microscope (Olympus IX73). The fluorescence intensity of the positively stained area was measured using ImageJ, and the average fluorescence intensity in each field of view was calculated.

### TUNEL assay

Apoptotic cells were identified by TUNEL staining using the Apoptosis Detection Kit (Yeasen Biotech, Shanghai, China) according to the manufacturer’s protocols. Briefly, cells were fixed for 25 min and then permeabilized with proteinase K (20 μg/ml) for 20 min. After incubating with TdT for 60 min, the sections were counterstained with DAPI. The slides were observed under a fluorescence microscope (Olympus IX73), and the number and proportion of apoptotic cells were counted.

### Statistical analysis

The SPSS 20.0 software (SPSS, Chicago, IL, United States) was used for statistical analyses. Data are presented as mean ± standard deviation (SD). Multiple groups were compared using one-way ANOVA, and the differences between two groups were analyzed using unpaired Student’s *t*-test. *p* < 0.05 was considered statistically significant.

## Results

### Andro treatment accelerated tissue regeneration and functional recovery after SCI

**FIGURE 1:**
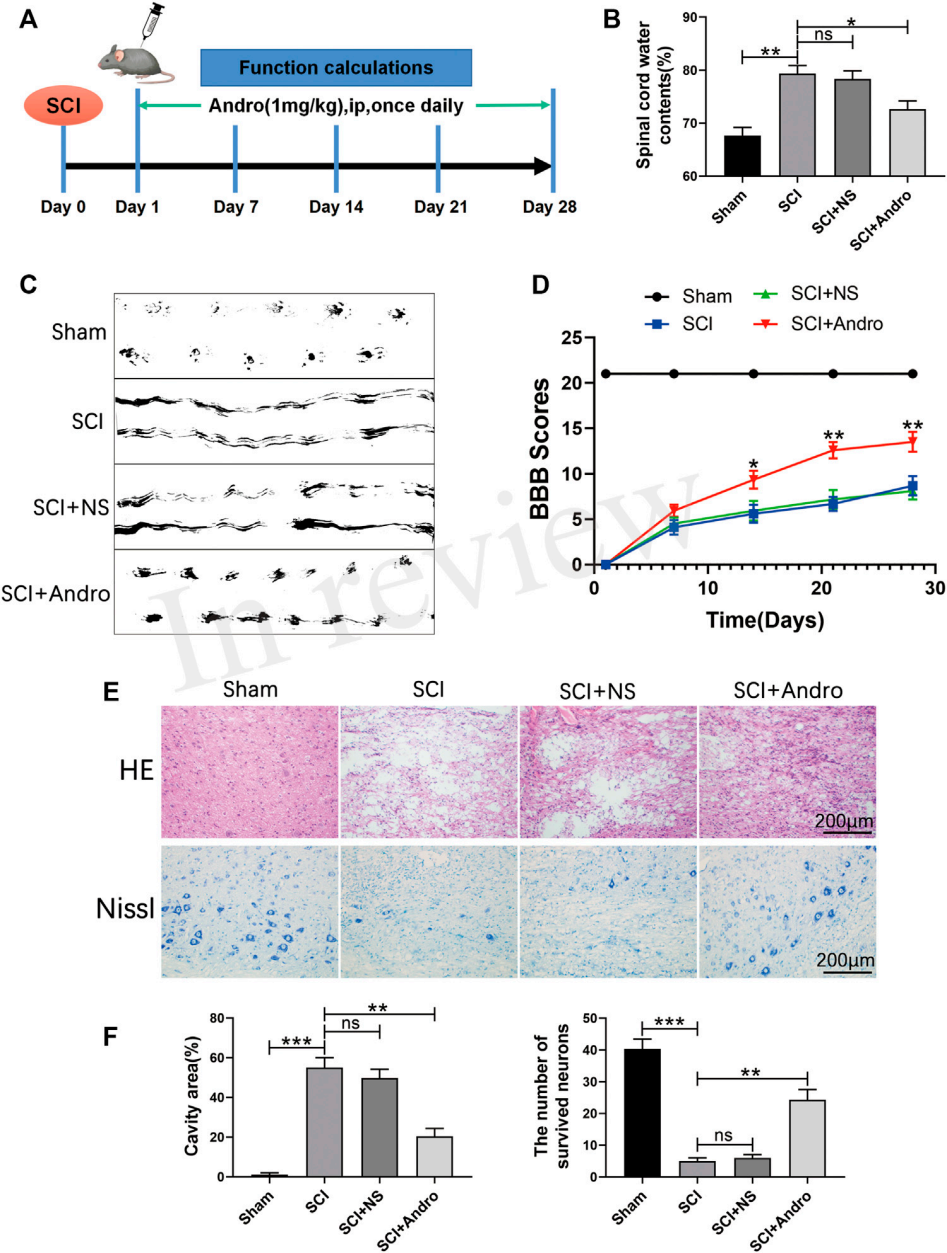
Andro treatment accelerated tissue regeneration and functional recovery after SCI. **(A)** Establishment of rat SCI model and Andro treatment protocol. **(B–C)** Spinal cord water content and footprint test of the different groups at 28 days post-SCI. **(D)** The Basso, Beattie and Bresnahan (BBB) locomotor scores of the different groups. **(E)** Representative images of the HE and Nissl stained sections of the lesion areas showing histological changes and surviving neurons of the different groups at 28 days post-SCI. **(F)** Relative lesion cavity area and the number of surviving neurons. Data are presented as the mean ± SD, *n* = 3 per group.*p < 0.05,**p < 0.01,***p < 0.001, ns *p*>0.05.

Rats were intraperitoneally injected with Andro (1 mg/kg) once daily for 28 days after SCI induction (Figure 1A). Tissue regeneration and functional recovery were evaluated by standard histological and behavioral tests. As shown in Figure 1B, the water content was significantly increased in the spinal cords after SCI, and restored by Andro treatment. In the footprint test, the untreated SCI rats showed dragging of both hindlimbs and were unable to walk, whereas those treated with Andro had relatively consistent footprints of both hindlimbs and partly regained their strength 28 days after SCI. Thus, Andro treatment improved the gait of the animals after SCI (Figure 1C). Unlike the sham-operated group, the groups with SCI induction had zero hindlimb movement post-operation. No significant differences were observed in the BBB scores between the untreated and saline-treated SCI groups on days 7, 14, 21 and 28 post-operation. However, Andro treatment significantly improved the BBB scores starting from the 14th day post-SCI, which remained consistently higher than that of the other SCI groups on days 21 and 28 (Figure 1D).

Histological examination of the spinal cords by HE staining showed significant deformities and cavities in the injured tissues compared to that in the healthy tissues of the sham-operated rats. However, Andro treatment significantly decreased the lesion area, as well as the degree of inflammation and tissue damage compared to that in the SCI and SCI + NS groups (Figures 1E,F). Neuronal survival was assessed by Nissl staining, which showed that the animals treated with Andro had numerous surviving neurons in the lesion area compared to the untreated and saline-treated rats (Figures 1E,F). Taken together, Andro treatment enhanced post-SCI tissue repair and functional recovery *in vivo*.

### Andro treatment reduced neuronal apoptosis after SCI

**FIGURE 2:**
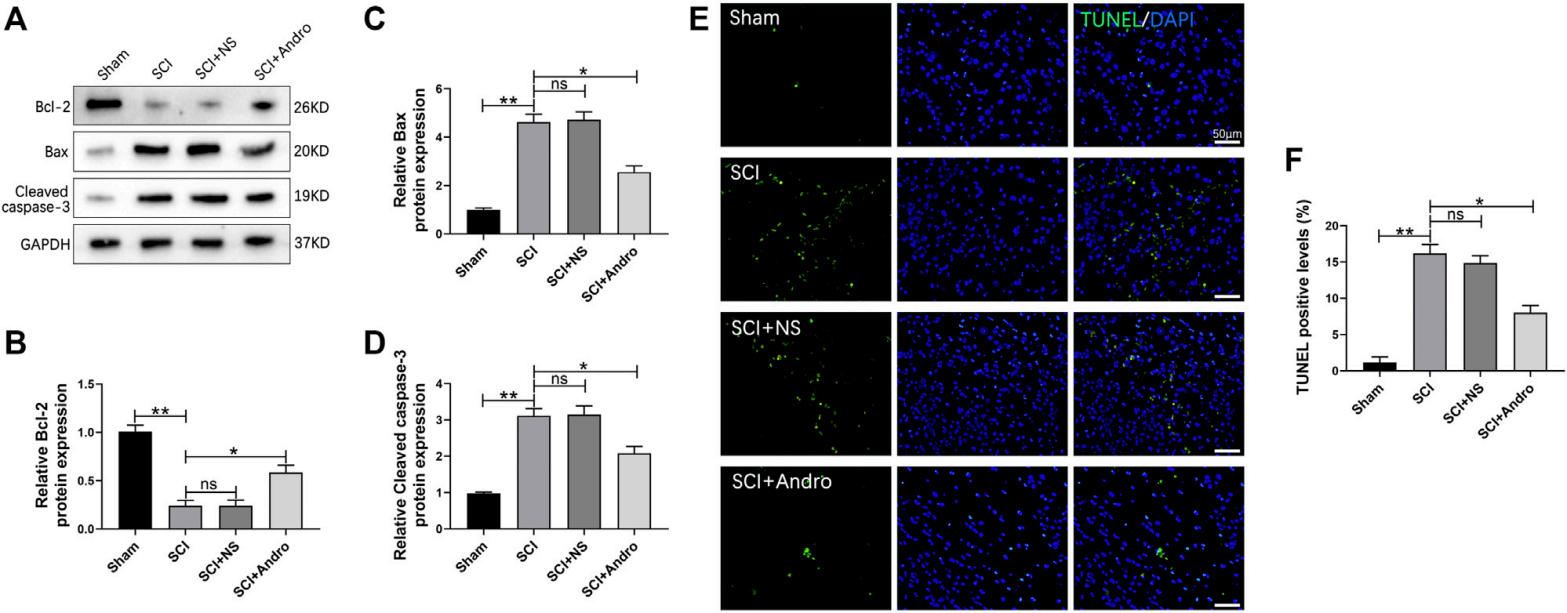
Andro treatment reduced neuronal apoptosis after SCI. **(A)** Western blot analysis showing the expression levels of apoptosis related proteins (Bcl-2, Bax and Cleaved caspase-3). **(B–D)** Quantification of relative Bcl-2, Bax and Cleaved caspase-3 protein levels. **(E)** TUNEL staining of neuronal apoptosis at 28 days after Andro treatment. **(F)** Quantitative estimation of the number of TUNEL positive cells. Data are presented as the mean ± SD, *n* = 3 per group.*p < 0.05,**p < 0.01, ns *p*>0.05.

SCI leads to the apoptosis of nerve cells surrounding the traumatized tissue. Consistent with this, the anti-apoptotic Bcl-2 protein was markedly decreased, while the pro-apoptotic Bax and cleaved caspase-3 proteins were significantly increased in the injured spinal cord tissues. Andro treatment not only restored the expression levels of Bcl-2 but also downregulated Bax and cleaved caspase-3 (Figures 2A–D). Furthermore, TUNEL staining also showed a significant increase in the number of TUNEL-positive apoptotic cells after SCI, which was mitigated by Andro treatment compared to the SCI and SCI + NS groups (Figures 2E,F). These results indicated that Andro treatment reduced SCI-induced apoptosis.

### Andro treatment reduced oxidative stress and inflammation of SCI rats

**FIGURE 3:**
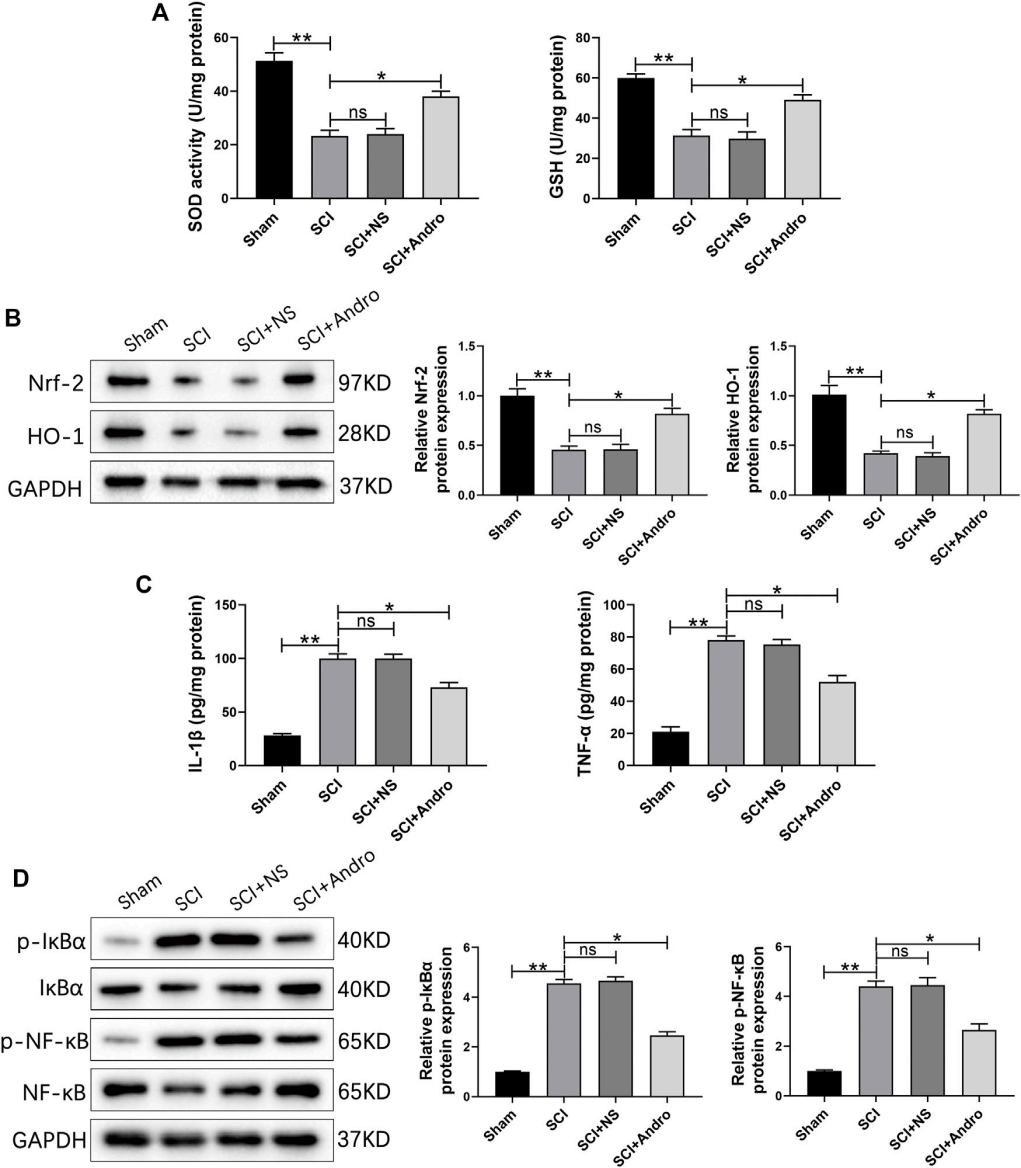
Andro treatment reduced oxidative stress and inflammation of SCI rats. **(A)** SOD levels and GSH activity in the indicated groups 7 days post-SCI. **(B)** Immunoblot and relative quantification of Nrf-2/HO-1 pathway-related proteins 7 days post-SCI. **(C)** IL-1β and TNF-α levels at 28 days post-SCI. **(D)** Immunoblot and relative quantification of NF-κB pathway-related proteins (p-IκBα and p-NF-κB) 28 days post-SCI. Data are presented as the mean ± SD, *n* = 3 per group.*p < 0.05,**p < 0.01, ns *p*>0.05.

Oxidative stress is a major driver of secondary injury after SCI, which is accompanied by a decrease in the activity of antioxidant enzymes such as SOD and GSH. As shown in Figure 3A, SCI significantly reduced SOD and GSH activities in the spinal cord tissues compared to that in the sham-operated rats after 7 days, which was restored to normal levels by Andro treatment. Nrf-2 is a “master regulator” of the cellular antioxidant response and activates the downstream HO-1 in response to oxidative stress. Both Nrf-2 and HO-1 were significantly increased in the Andro-treated versus the untreated SCI rats at 7 days post-SCI (Figure 3B), indicating that Andro protected the damaged tissues against oxidative stress by activating the Nrf-2/HO-1 signaling pathway.

**FIGURE 4:**
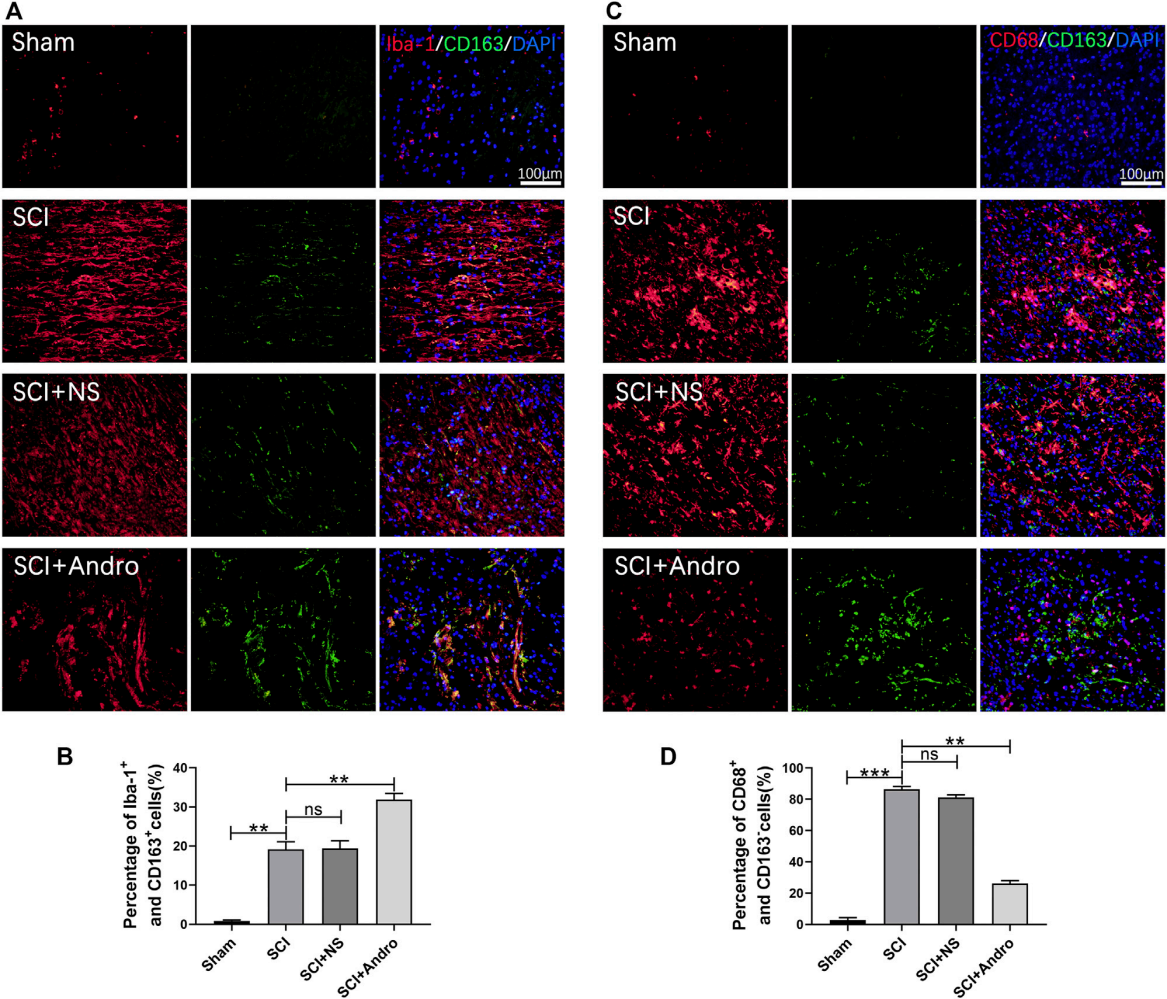
Andro treatment promoted microglia polarization toward the M2 phenotype after SCI. **(A–B)** Co-immunofluorescent staining of Iba-1 (red)/CD163 (green) and relative quantification of Iba-1^+^/CD163^+^ cells in spinal cord tissues 28 days post-SCI. **(C–D)** Immunofluorescent staining of CD68 (red)/CD163 (green) and relative quantification of CD68^+^/CD163^-^ cells in spinal cord tissues 28 days post-SCI. Data are presented as the mean ± SD, *n* = 3 per group.*p < 0.05,**p < 0.01,***p < 0.001, ns *p*>0.05.

Inhibiting excessive tissue inflammation is critical for reducing apoptosis and promoting functional recovery after SCI. We found that the pro-inflammatory cytokines (IL-1β and TNF-α) were elevated in the SCI and SCI + NS groups compared to the sham-operated group, and decreased significantly after Andro treatment (Figure 3C). The NF-κB signaling pathway mediates inflammatory responses by promoting cytokine secretion, and also regulates oxidative stress and apoptosis. As shown in Figure 3D, the expression of IκBα and NF-κB were significantly increased in SCI rats compared to that in the sham-operated rats, and downregulated by Andro treatment. Thus, Andro inhibited the release of pro-inflammatory cytokines by blocking the activation of NF-κB pathway. Microglia with M1 phenotype release inflammatory factors and neurotoxins, thereby aggravating tissue damage and neuronal death. On the other hand, the M2 microglia produce anti-inflammatory factors to promote the clearance of invading pathogens and necrotic cells. The microglial polarization in the injured tissues was assessed by immunostaining for the microglia marker Iba-1 and M2 marker CD163. The findings suggested that Iba-1-labeled microglia and CD163-labeled M2 macrophages were significantly increased in SCI rats, whereas Andro treatment significantly decreased Iba-1 and increased CD163 expression (Figures 4A,B). Meanwhile, CD68-labeled macrophages and CD163-labeled M2 macrophages were significantly increased in SCI rats, but Andro treatment significantly downregulated CD68 and upregulated CD163 (Figures 4C,D). Thus, Andro inhibited M1 polarization and promoted the M2 phenotype of macrophages, which mitigated excessive inflammatory response and promoted tissue repair after SCI. Taken together, Andro treatment reduced oxidative stress and inflammation in the injured spinal cords.

### Andro treatment promoted axonal regeneration and remyelination after SCI

**FIGURE 5:**
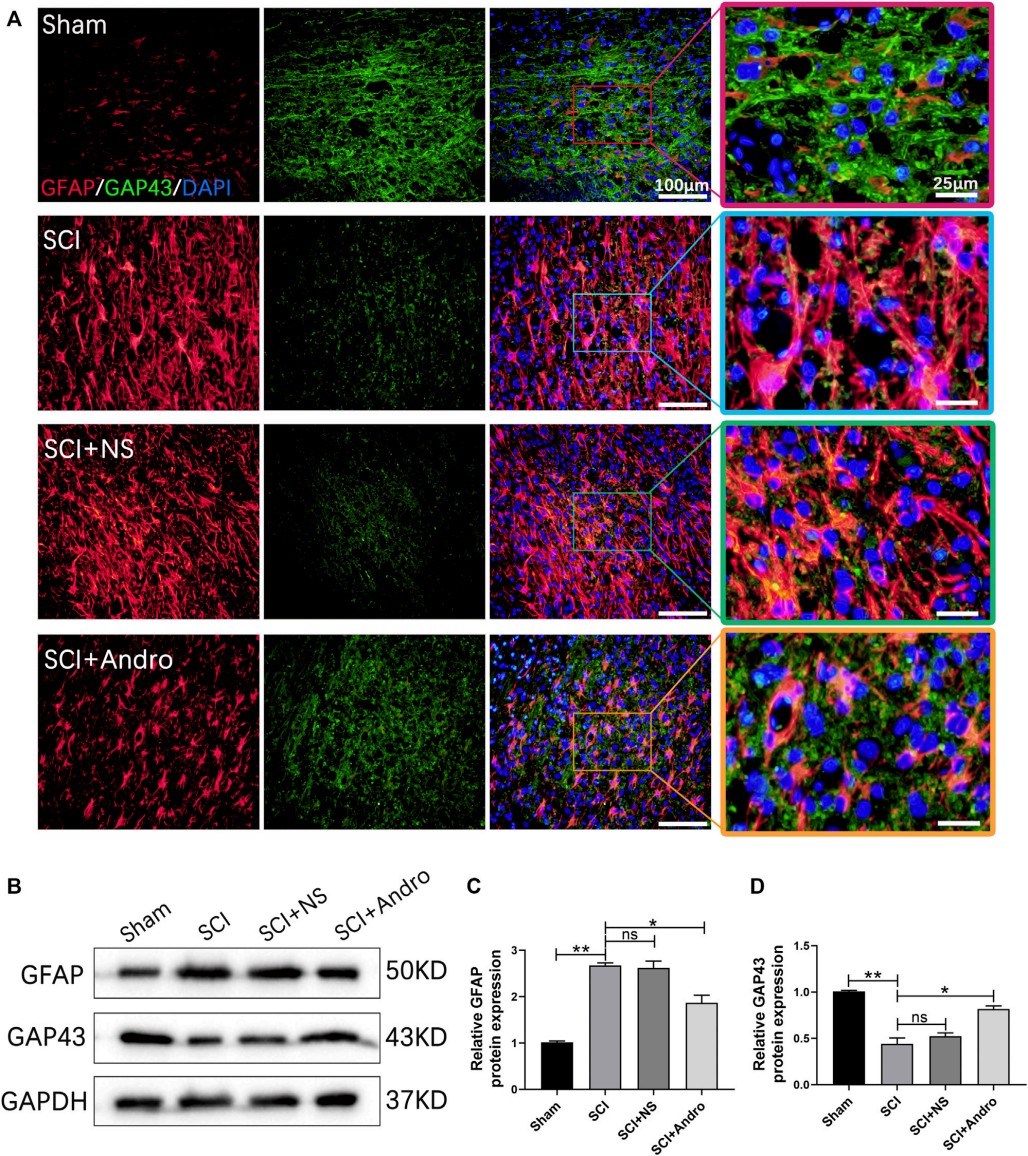
Andro treatment promoted axonal regeneration of SCI rats. **(A)** Co-immunofluorescent staining showing the glial scar (GFAP, red) and the axonal regeneration (GAP43,green) in spinal cord tissues 28 days post-SCI. **(B–D)** Immunoblot and relative quantification of GFAP and GAP43 proteins. Data are presented as the mean ± SD, *n* = 3 per group.*p < 0.05,**p < 0.01, ns *p*>0.05.

The formation of glial scars is a major physiological barrier to axonal regeneration after SCI. Therefore, we analyzed the *in situ* expression of GFAP and GAP43 in the injured tissues to evaluate glial scar formation and axonal regeneration. GAP43 is closely associated with neuronal regeneration and can regulate axonal growth and new axonal connections. In the SCI and SCI + NS groups, GFAP + glial scars were abundant and a only few GAP43 + axons were detected. However, treatment with Andro significantly decreased GFAP + scars and increased the number of GAP43 + axons (Figure 5A). The immunostaining results were confirmed by the increased expression level of GAP43 protein in the immunoblots from Andro-treated group (Figures 5B–D). In general, the findings suggested that Andro treatment decreased the formation of glial scars and promoted axonal regeneration after SCI.

**FIGURE 6:**
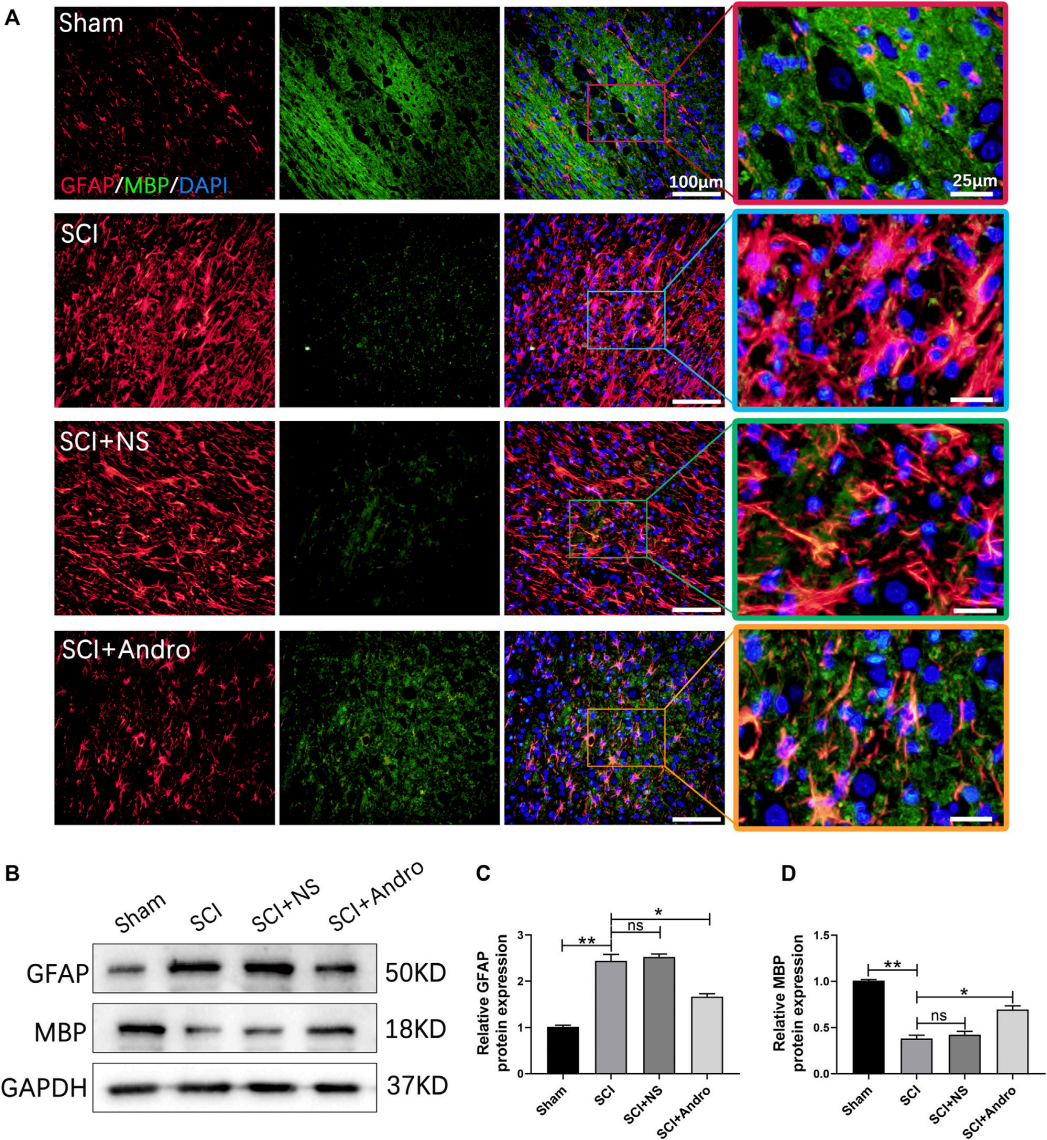
Andro treatment enhanced axonal remyelination in SCI rats. **(A)** Co-immunofluorescent staining showed the glial scar (GFAP, red) and the axonal remyelination (MBP, green) in spinal cord tissues 28 days post-SCI. **(B–D)** Immunoblot and relative quantification of GFAP and MBP proteins. Data are presented as the mean ± SD, *n* = 3 per group.*p < 0.05,**p < 0.01, ns *p*>0.05.

Axonal myelination plays a key role in maintaining neurological functions. Therefore, we also analyzed the effect of Andro on remyelination by immunostaining the tissues with GFAP and MBP, a major myelin protein and a marker of active remyelination. As shown in Figure 6A, the expression of MBP was lower at the lesion sites in the untreated rats but increased significantly in the Andro-treated group. In contrast, GFAP levels were significantly decreased in the SCI + Andro group compared to the SCI and SCI + NS groups (Figure 6A). Similar results were obtained in western blotting experiments (Figures 6B–D). These findings suggested that Andro treatment promoted axonal remyelination after SCI.

### Andro treatment abrogated H_2_O_2_-induced apoptosis, oxidative stress and inflammation in cortical neurons

**FIGURE 7:**
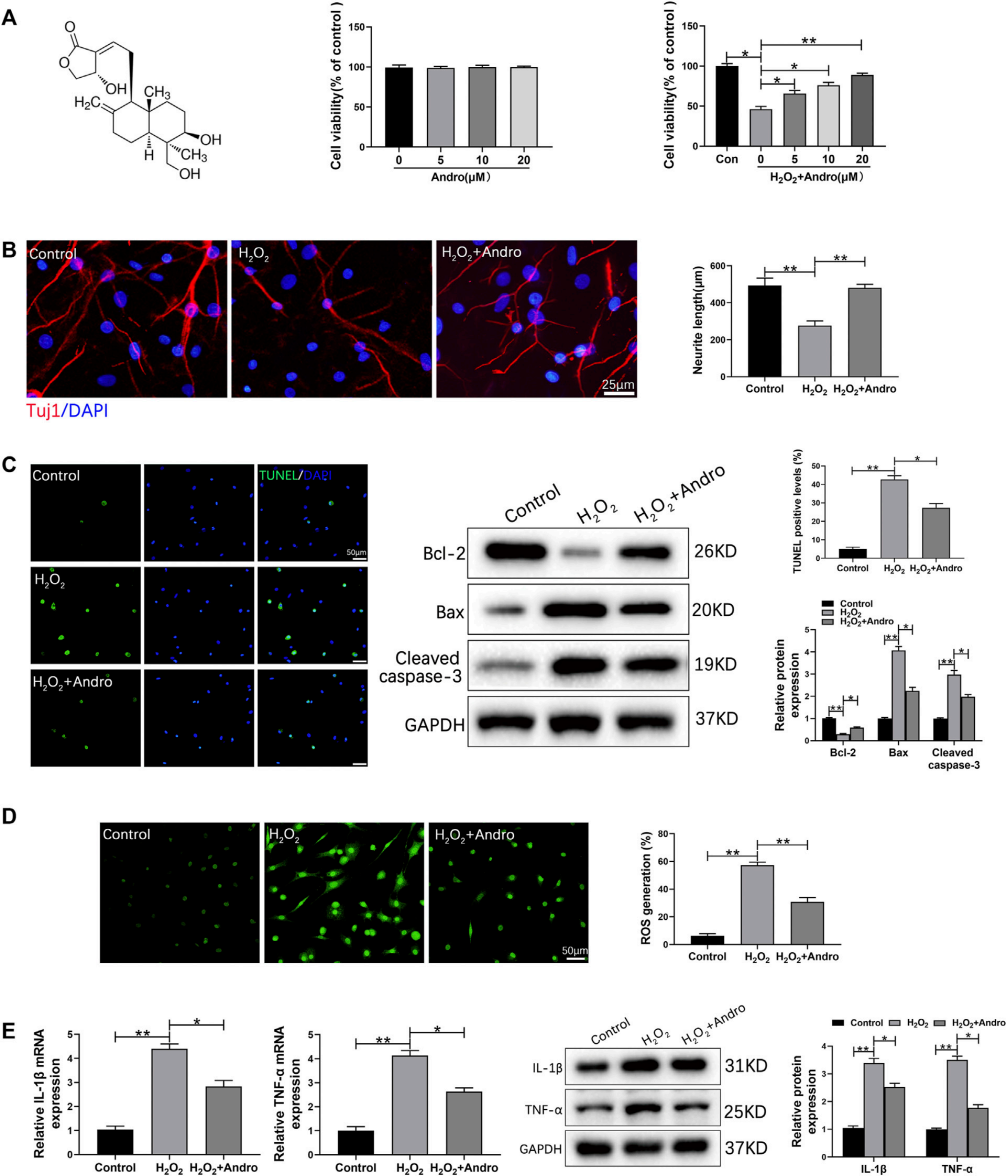
Andro treatment abrogated H_2_O_2_-induced apoptosis, oxidative stress and inflammation in cortical neurons. **(A)** The molecular structure of Andro and cell viability in response to different concentrations of Andro. **(B)** Immunofluorescence images showing the axon labeled with Tuj1 in primary cortical neurons and the mean neurite length ranges was counted and ploted. **(C)** TUNEL staining showing apoptotic cells and immunoblot showing the expression levels of Bcl-2, Bax and Cleaved caspase-3. **(D)** ROS production in cortical neurons was measured by DCF staining. **(E)** IL-1β and TNF-α mRNA and protein levels in the indicated groups. Data are presented as the mean ± SD, *n* = 3 per group.*p < 0.05,**p < 0.01, ns *p*>0.05.

To further investigate the effects of Andro on apoptosis, inflammation and oxidative stress *in vitro*, we treated rat cortical neurons with 300 μM H_2_O_2_ in the presence or absence of Andro. The chemical structure of Andro is shown in Figure 7A. Rat cortical neurons treated with different concentrations of Andro (0, 5, 10 and 20 μM) for 24 h were viable. Furthermore, Andro enhanced viability of the H_2_O_2_-treated cells in a dose-dependent manner, and the 20 μM dose was used for subsequent experiments (Figure 7A). First of all, we found that Andro promoted the generation capacity of neurons by H_2_O_2_ treatment (Figure 7B).As shown in Figure 7C, H_2_O_2_ markedly increased the number of apoptotic cells, which was decreased by Andro treatment. Consistent with this, Andro significantly upregulated Bcl-2, and inhibited the expression of Bax and cleaved caspase-3. Furthermore, DCF analysis suggested that ROS production was increased in H_2_O_2_-treated rat cortical neurons compared to the controls, and decreased by Andro (Figure 7D). In addition, the expression levels of IL-1β and TNF-α transcripts also increased in H_2_O_2_-treated rat cortical neurons, and were downregulated by Andro (Figure 7E). Taken together, Andro treatment alleviated H_2_O_2_-induced apoptosis, oxidative stress and inflammation in rat cortical neurons.

## Discussion

Secondary injury following SCI induction is characterized by neuronal apoptosis, oxidative stress and inflammation, which leads to neuronal demyelination and axonal degeneration, and exacerbates neurological dysfunction (Zhang and Shi, 2010; Ahuja et al., 2017a; Hou et al., 2021). However, there are currently no treatment strategies that can effectively inhibit secondary injury and promote functional recovery after SCI. Therefore, it is crucial to develop therapeutic strategies that can alleviate secondary injury in a timely manner and promote neurological recovery.

Andro, a bioactive compound of the medicinal herb *A. paniculate*, has exhibited neuroprotective, anti-apoptotic, antioxidant and anti-inflammatory in different models of neurological diseases (Geng et al., 2019; Xu et al., 2019; Zhang et al., 2021; Gong et al., 2022). It attenuated neuronal apoptosis and oxidative stress in subarachnoid hemorrhage by activating the Nrf2/HO-1 signaling pathway (Gong et al., 2022), and protected PC12 neuronal cell line against inflammation- and oxidative stress-related injury by respectively inhibiting the NF-κB pathway and activating the Nrf2/HO-1 pathway (Xu et al., 2019). Furthermore, APP/PS1 transgenic mice treated with Andro exhibited lower microglial activation and secretion of pro-inflammatory factors (Zhang et al., 2021).

However, the neuroprotective effects of Andro in SCI have not been elucidated completely. To this end, we explored its therapeutic effects and the possible underlying mechanisms in a rat model of SCI. Andro accelerated tissue repair and functional recovery after SCI, alleviated neuronal apoptosis, oxidative stress and inflammation through the Nrf-2/HO-1 and NF-κB signaling pathways, promoted M2 polarization of macrophages, and enhanced axonal regeneration and remyelination. In addition, Andro treatment relieved spinal cord edema and reduced the cavity area, which coincided with neuron survival. The rats treated with Andro exhibited significantly improved gait and locomotive performance compared to the untreated group, indicating that it can restore motor function after SCI.

The loss spinal neurons and glial cells after SCI is attributed to apoptosis, which also affects neuronal function (Toescu, 1998; Bahney and von Bartheld, 2018). Therefore, promoting neuronal survival and inhibiting apoptosis can increase the chances of nerve regeneration after injury. We found that Andro treatment significantly increased the expression of Bcl-2, and inhibited Bax and cleaved caspase-3. Consistent with this, rats treated with Andro had fewer apoptotic cells in the injured spinal cord tissues. Similar results were observed for the H_2_O_2_-stimulated cortical neurons *in vitro*. Thus, Andro alleviated secondary injury after SCI by inhibiting neuronal apoptosis. Consistent with these results, a recent study has shown that Andro inhibited bupivacaine-induced apoptosis by downregulating Bax and active caspase three and upregulating Bcl2 in SH-SY5Y cells (Zhang et al., 2019).

Futhermore, studies show that oxidative stress and inflammatory responses are critical factors in the progression of secondary injury after SCI (Li et al., 2019; Liu et al., 2020). Andro can protect neurons against inflammation-mediated injury and oxidative damage *via* NF-κB inhibition and Nrf-2/HO-1 activation (Tao et al., 2018; Xu et al., 2019). We found that Andro treatment reversed the decrease in the activity of antioxidant enzymes SOD and GSH after SCI, indicating a protective function against oxidative stress. Nrf-2 is a key regulatory protein in the endogenous antioxidant defense system that initiates the transcription of antioxidant enzymes (Xia et al., 2018; Gong et al., 2022). HO-1 is an antioxidant protein downstream of the Nrf-2/ARE signaling pathway (Li et al., 2019). Upregulation of HO-1 can reduce inflammation and oxidative stress following SCI (Li et al., 2019), which translates to neuronal repair (Xia et al., 2018; Dai et al., 2021)and increased functional recovery. Andro treatment increased the expression of Nrf-2 and HO-1, indicating that its antioxidant is likely mediated *via* activation of the Nrf-2/HO-1 signaling pathway. In the *in vitro* studies, we found that Andro inhibited intercellular ROS generation, suggesting that the H_2_O_2_-induced oxidative stress in the cultured cortical neurons was also alleviated by Andro treatment. These results are consistent with those of previous studies showing that Andro can alleviate oxidative stress by directly inhibiting ROS production *via* the Nrf2/HO-1 signaling pathway (Xu et al., 2019; Gong et al., 2022).

The NF-κB signaling pathway is a classical inflammatory pathway involved in various neurological diseases (Lawrence, 2009; Liu et al., 2021). SCI results in significant local inflammation, as indicated by elevated pro-inflammatory cytokines (IL-1β and TNF-α) and activated IκBα/NF-κB signaling pathway. However, Andro treatment not only mitigated cytokine levels but also inhibited IκBα/NF-κB, indicating that Andro can alleviate inflammation-mediated injury by inhibiting the NF-κB signaling pathway. These results are consistent with previous findings (Xu et al., 2019). The anti-inflammatory effect of Andro was further validated in the H_2_O_2_-treated cortical neurons model. Overall, these results indicated that Andro treatment reduced oxidative stress and inflammation in the injured spinal tissues.

Oxidative stress and inflammation are the key drivers of secondary local SCI, and exhibit a functional crosstalk. ROS deplete cellular antioxidants and damage cells and organelles, which initiates or intensifies inflammation (Eastman et al., 2020). In addition, the activation of oxidative stress and inflammatory responses aggravate apoptosis of residual neurons, which further deteriorates the local microenvironment at the injury site, and inhibit neuronal regeneration and the recovery of neural function (Hou et al., 2021; Fakhri et al., 2022).

Microglia are specialized macrophage-like cells that constitute the innate immune population in the central nervous system. The M1 microglia produce pro-inflammatory cytokines that cause neurotoxicity, while the M2 phenotype protect injured nerves by producing anti-inflammatory cytokines (Xu et al., 1999; Gaojian et al., 2020). The predominant microglial population in the injured spinal cord microenvironment is of the M1 type, which not only aggravates secondary injury but also hinders nerve regeneration and remodeling (Wang W et al., 2018; Fan et al., 2019). Therefore, blocking the hyperpolarization of microglia to the M1 phenotype, and promoting M2 polarization can aid in functional recovery after SCI by maintaining an optimal M1/M2 balance. Studies show that Andro could reduce inflammation-mediated neuronal damage by modulating macrophage and microglia overactivation (Wang et al., 2004; Yang et al., 2017). We observed M1 microglia in the fibrotic component of the glial scars. Andro treatment significantly reduced the number and distribution of M1 microglia and increased that of M2 microglia, suggesting that Andro can attenuate the excessive inflammatory response after SCI by restoring the M1/M2 ratio.

SCI activates multiple cells in the vicinity of the affected tissue, which then proliferate and form glial scars. Although glial scars can control the progression of secondary injury, they also act as a physical and chemical barrier against axonal regeneration (Rolls et al., 2009; Tran et al., 2018). Therefore, reducing glial scars and cavities can promote axonal regeneration and remyelination, and improve functional recovery after SCI. In our study as well, we detected extensive glial scar formation in the injured spinal cord tissue that was characterized by accumulation of GFAP + astrocytes. However, Andro treatment significantly increased the expression of GAP43 in the injured tissues and decreased the number of astrocytes, which in turn mitigated glial scarring and promoted axonal regeneration, eventually bridging the gaps at lesion sites and restoring tissue continuity. These results are consistent with previous findings (Liang et al., 2017; Li et al., 2021).

Myelin is critical for maintaining the integrity and neurological functions of axons (Wu and Ren, 2009; Fan et al., 2016). Secondary injury after SCI causes edema, inflammation, oxidative stress and oligodendrocyte necrosis and apoptosis, leading to axonal demyelination (Hassannejad et al., 2019). The degree of remyelination directly affects tissue repair after SCI. Although spontaneous remyelination occurs in a fraction of the axons, persistent demyelination eventually leads to irreversible neurological dysfunction (Mi et al., 2013). Previous studies have shown that remyelination can prevent axonal degeneration and restore function following SCI (Alizadeh and Karimi-Abdolrezaee, 2016). Consistent with this, Andro treatment significantly increased the expression of the remyelination protein MBP in the injured spinal cord, indicating that it promotes axonal remyelination after SCI.

## Conclusion

**FIGURE 8:**
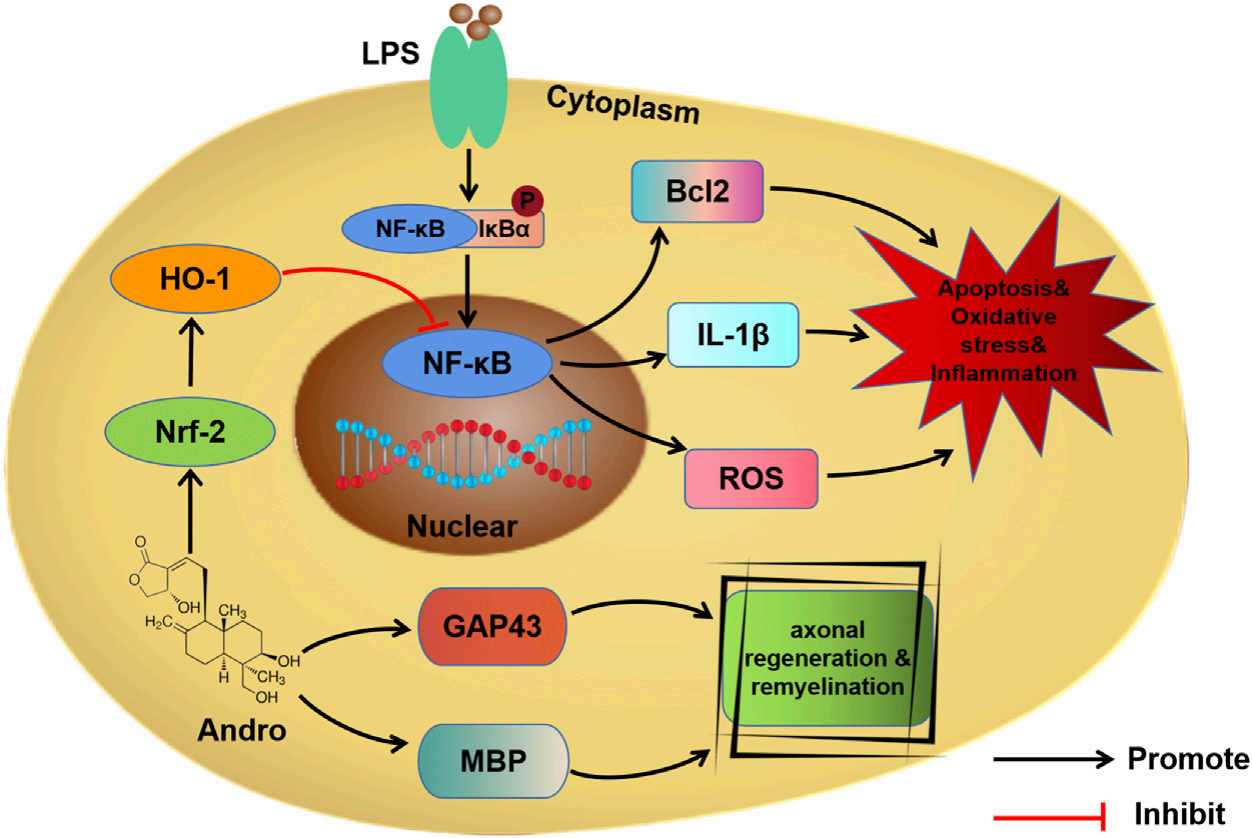
Schematic diagram showing the mechanism of Andro.

Andro alleviated apoptosis, oxidative stress and inflammation in the *in vitro* and *in vivo* models of SCI *via* the Nrf-2/HO-1 and NF-κB signaling pathways (Figure 8). Furthermore, Andro treatment polarized macrophages to the M2 phenotype, and promoted axonal regeneration and remyelination. Thus, Andro can promote motor functional recovery after SCI and warrants clinical validation.

## Data Availability

The original contributions presented in the study are included in the article/Supplementary Materials, further inquiries can be directed to the corresponding authors.

## Glossary

SCI: spinal cord injury
Andro: Andrographolide;
AD: Alzheimer’s disease
PD: Parkinson’s disease
CCH: chronic cerebral hypoperfusion
NLRP3: NLR Family Pyrin Domain Containing 3
SPF: specific pathogen-free
SD: Sprague-Dawley
NS: sterile saline;
AST: Astrocytes
FBS: fetal bovine serum
CCK-8: Cell Counting Kit-8
GSH: glutathione
SOD: superoxide dismutase
IL-1β: interleukin 1β
TNF-α: tumor necrotic factor-α
ROS: Reactive oxygen species
2,7- DCF-DA: 2,7-dichlorodihydrofluorescein diacetate
BBB: Basso, Beattie, and Bresnahan
PFA: paraformaldehyde
HE: hematoxylin-eosin
RIPA: Radio Immunoprecipitation Assay
BCA: bicinchoninic acid
PVDF: polyvinylidene difluoride; Nrf-2:Nuclear factor E2-related factor 2
HO-1: Heme oxygenase-1; NF-κB:Nuclear factor-kappa B
GFAP: Glial fibrillary acidic protein
GAP43: Growth-associated protein 43
MBP: Myelin basic protein
TBST: Tris buffer solution
RT: room temperature
qRT-PCR: Quantitative real-time polymerase chain reaction
SDs: standard deviations.

## References

[B1] Ahmed S. Kwatra M. Ranjan P. S. Murty U. Naidu V. (2021). Andrographolide suppresses NLRP3 inflammasome activation in microglia through induction of parkin-mediated mitophagy in *in-vitro* and *in-vivo* models of Parkinson disease. Brain Behav. Immun. 91, 142–158. 10.1016/j.bbi.2020.09.017 32971182

[B2] Ahuja C. S. Nori S. Tetreault L. Wilson J. Kwon B. Harrop J. (2017a). Traumatic spinal cord injury—Repair and regeneration. Neurosurgery 80 (3S), S9–S22. 10.1093/neuros/nyw080 28350947

[B3] Ahuja C. S. Wilson J. R. Nori S. Kotter M. Druschel C. Curt A. (2017b). Traumatic spinal cord injury. Nat. Rev. Dis. Prim. 3 (1), 17018–17021. 10.1038/nrdp.2017.18 28447605

[B4] Alizadeh A. Karimi-Abdolrezaee S. (2016). Microenvironmental regulation of oligodendrocyte replacement and remyelination in spinal cord injury. J. Physiol. 594 (13), 3539–3552. 10.1113/JP270895 26857216 PMC4929323

[B5] Amar A. P. Levy M. L. (1999). Pathogenesis and pharmacological strategies for mitigating secondary damage in acute spinal cord injury. Neurosurgery 44 (5), 1027–1039. discussion 1039-40. 10.1097/00006123-199905000-00052 10232536

[B6] Bahney J. von Bartheld C. S. (2018). The cellular composition and glia–neuron ratio in the spinal cord of a human and a nonhuman primate: Comparison with other species and brain regions. Anat. Rec. 301 (4), 697–710. 10.1002/ar.23728 PMC5845477 29150977

[B7] Basso D. M. Beattie M. S. Bresnahan J. C. (1995). A sensitive and reliable locomotor rating scale for open field testing in rats. J. Neurotrauma 12 (1), 1–21. 10.1089/neu.1995.12.1 7783230

[B8] Dai X. Cai Z. Liu J. (2021). Up-regulation of miR-338-5p after spinal cord injury enhances the neuronal repair via inhibition of inflammation aggravation and oxidative stress. Minerva Med. 112 (4), 533–534. 10.23736/S0026-4806.19.06280-3 31833735

[B9] Dai Y. Chen S. R. Chai L. Zhao J. Wang Y. Wang Y. (2019). Overview of pharmacological activities of Andrographis paniculata and its major compound andrographolide. Crit. Rev. Food Sci. Nutr. 59, S17–S29. 10.1080/10408398.2018.1501657 30040451

[B10] Eastman C. L. D'Ambrosio R. Ganesh T. (2020). Modulating neuroinflammation and oxidative stress to prevent epilepsy and improve outcomes after traumatic brain injury. Neuropharmacology 172, 107907. 10.1016/j.neuropharm.2019.107907 31837825 PMC7274911

[B11] Fakhri S. Abbaszadeh F. Moradi S. Z. Cao H. Khan H. Xiao J. (2022). Effects of polyphenols on oxidative stress, inflammation, and interconnected pathways during spinal cord injury. Oxid. Med. Cell. Longev. 2022, 8100195. 10.1155/2022/8100195 35035667 PMC8759836

[B12] Fan H. Tang H. B. Shan L. Q. Liu S. C. Huang D. G. Chen X. (2019). Quercetin prevents necroptosis of oligodendrocytes by inhibiting macrophages/microglia polarization to M1 phenotype after spinal cord injury in rats. J. Neuroinflammation 16 (1), 206. 10.1186/s12974-019-1613-2 31699098 PMC6839267

[B13] Fan H. Zhang K. Shan L. Kuang F. Chen K. Zhu K. (2016). Reactive astrocytes undergo M1 microglia/macrohpages-induced necroptosis in spinal cord injury. Mol. Neurodegener. 11, 14. 10.1186/s13024-016-0081-8 26842216 PMC4740993

[B14] Gaojian T. Dingfei Q. Linwei L. Xiaowei W. Zheng Z. Wei L. (2020). Parthenolide promotes the repair of spinal cord injury by modulating M1/M2 polarization via the NF-κB and STAT 1/3 signaling pathway. Cell. Death Discov. 6 (1), 97. 10.1038/s41420-020-00333-8 33083018 PMC7538575

[B15] Geng J. Liu W. Gao J. Jiang C. Fan T. Sun Y. (2019). Andrographolide alleviates Parkinsonism in MPTP-PD mice via targeting mitochondrial fission mediated by dynamin-related protein 1. Br. J. Pharmacol. 176 (23), 4574–4591. 10.1111/bph.14823 31389613 PMC6932945

[B16] Gong P. Zhang W. Zou C. Han S. Tian Q. Wang J. (2022). Andrographolide attenuates blood-brain barrier disruption, neuronal apoptosis, and oxidative stress through activation of Nrf2/HO-1 signaling pathway in subarachnoid hemorrhage. Neurotox. Res. 40 (2), 508–519. 10.1007/s12640-022-00486-7 35305248

[B17] Hassannejad Z. Yousefifard M. Azizi Y. Zadegan S. A. Sajadi K. Sharif-Alhoseini M. (2019). Axonal degeneration and demyelination following traumatic spinal cord injury: A systematic review and meta-analysis. J. Chem. Neuroanat. 97, 9–22. 10.1016/j.jchemneu.2019.01.009 30726717

[B18] Hou Y. Luan J. Huang T. Deng T. Li X. Xiao Z. (2021). Tauroursodeoxycholic acid alleviates secondary injury in spinal cord injury mice by reducing oxidative stress, apoptosis, and inflammatory response. J. Neuroinflammation 18 (1), 216. 10.1186/s12974-021-02248-2 34544428 PMC8454169

[B19] Jazayeri S. B. Beygi S. Shokraneh F. Hagen E. M. Rahimi-Movaghar V. (2015). Incidence of traumatic spinal cord injury worldwide: A systematic review. Eur. Spine J. 24 (5), 905–918. 10.1007/s00586-014-3424-6 24952008

[B20] Kjell J. Olson L. (2016). Rat models of spinal cord injury: From pathology to potential therapies. Dis. Model. Mech. 9 (10), 1125–1137. 10.1242/dmm.025833 27736748 PMC5087825

[B21] Lawrence T. (2009). The nuclear factor NF-kappaB pathway in inflammation. Cold Spring Harb. Perspect. Biol. 1 (6), a001651. 10.1101/cshperspect.a001651 20457564 PMC2882124

[B22] Lee B. B. Cripps R. A. Fitzharris M. Wing P. C. (2014). The global map for traumatic spinal cord injury epidemiology: Update 2011, global incidence rate. Spinal Cord. 52 (2), 110–116. 10.1038/sc.2012.158 23439068

[B23] Li Y. Xiang L. L. Miao J. X. Miao M. S. Wang C. (2021). Protective effects of andrographolide against cerebral ischemiareperfusion injury in mice. Int. J. Mol. Med. 48 (4), 186. 10.3892/ijmm.2021.5019 34368862 PMC8416143

[B24] Li Z. Wu F. Xu D. Zhi Z. Xu G. (2019). Inhibition of TREM1 reduces inflammation and oxidative stress after spinal cord injury (SCI) associated with HO-1 expressions. Biomed. Pharmacother. 109, 2014–2021. 10.1016/j.biopha.2018.08.159 30551457

[B25] Liang Y. Li M. Lu T. Peng W. Wu J. H. (2017). Andrographolide promotes neural differentiation of rat adipose tissue-derived stromal cells through wnt/β-catenin signaling pathway. Biomed. Res. Int. 2017, 4210867. 10.1155/2017/4210867 29085837 PMC5632471

[B26] Liu H. Zhang J. Xu X. Lu S. Yang D. Xie C. (2021). SARM1 promotes neuroinflammation and inhibits neural regeneration after spinal cord injury through NF-κB signaling. Theranostics 11 (9), 4187–4206. 10.7150/thno.49054 33754056 PMC7977471

[B27] Liu Z. Yao X. Jiang W. Li W. Zhu S. Liao C. (2020). Advanced oxidation protein products induce microglia-mediated neuroinflammation via MAPKs-NF-κB signaling pathway and pyroptosis after secondary spinal cord injury. J. Neuroinflammation 17 (1), 90. 10.1186/s12974-020-01751-2 32192500 PMC7082940

[B28] Luo D. Li X. Hou Y. Hou Y. Luan J. Weng J. (2022). Sodium tanshinone IIA sulfonate promotes spinal cord injury repair by inhibiting blood spinal cord barrier disruption *in vitro* and *in vivo* . Drug Dev. Res. 83 (3), 669–679. 10.1002/ddr.21898 34842291

[B29] Mi S. Pepinsky R. B. Cadavid D. (2013). Blocking LINGO-1 as a therapy to promote CNS repair: From concept to the clinic. CNS Drugs 27 (7), 493–503. 10.1007/s40263-013-0068-8 23681979

[B30] Orr M. B. Gensel J. C. (2018). Spinal cord injury scarring and inflammation: Therapies targeting glial and inflammatory responses. Neurotherapeutics 15 (3), 541–553. 10.1007/s13311-018-0631-6 29717413 PMC6095779

[B31] Patel R. Kaur K. Singh S. (2021). Protective effect of andrographolide against STZ induced Alzheimer's disease in experimental rats: Possible neuromodulation and aβ_(1-42)_ analysis. Inflammopharmacology 29 (4), 1157–1168. 10.1007/s10787-021-00843-6 34235591

[B32] Ren X. Xu W. Sun J. Dong B. Awala H. Wang L. (2021). Current trends on repurposing and pharmacological enhancement of andrographolide. Curr. Med. Chem. 28 (12), 2346–2368. 10.2174/0929867327666200810135604 32778020

[B33] Rolls A. Shechter R. Schwartz M. (2009). The bright side of the glial scar in CNS repair. Nat. Rev. Neurosci. 10 (3), 235–241. 10.1038/nrn2591 19229242

[B34] Tao L. Zhang L. Gao R. Jiang F. Cao J. Liu H. (2018). Andrographolide alleviates acute brain injury in a rat model of traumatic brain injury: Possible involvement of inflammatory signaling. Front. Neurosci. 12, 657. 10.3389/fnins.2018.00657 30294256 PMC6158349

[B35] Toescu E. C. (1998). Apoptosis and cell death in neuronal cells: Where does Ca2+ fit in? Cell. Calcium 24 (5-6), 387–403. 10.1016/s0143-4160(98)90062-8 10091008

[B36] Tran A. P. Warren P. M. Silver J. (2018). The Biology of regeneration failure and success after spinal cord injury. Physiol. Rev. 98 (2), 881–917. 10.1152/physrev.00017.2017 29513146 PMC5966716

[B37] Wang C. Wang Q. Lou Y. Xu J. Feng Z. Chen Y. (2018). Salidroside attenuates neuroinflammation and improves functional recovery after spinal cord injury through microglia polarization regulation. J. Cell. Mol. Med. 22 (2), 1148–1166. 10.1111/jcmm.13368 29148269 PMC5783886

[B38] Wang D. Yin H. Lin Q. Fang S. Shen J. Wu Y. (2019). Andrographolide enhances hippocampal BDNF signaling and suppresses neuronal apoptosis, astroglial activation, neuroinflammation, and spatial memory deficits in a rat model of chronic cerebral hypoperfusion. Naunyn. Schmiedeb. Arch. Pharmacol. 392 (10), 1277–1284. 10.1007/s00210-019-01672-9 31187188

[B39] Wang T. Liu B. Zhang W. Wilson B. Hong J. S. (2004). Andrographolide reduces inflammation-mediated dopaminergic neurodegeneration in mesencephalic neuron-glia cultures by inhibiting microglial activation. J. Pharmacol. Exp. Ther. 308 (3), 975–983. 10.1124/jpet.103.059683 14718612

[B40] Wang W. Liu R. Su Y. Li H. Xie W. Ning B. (2018). MicroRNA-21-5p mediates TGF-beta-regulated fibrogenic activation of spinal fibroblasts and the formation of fibrotic scars after spinal cord injury. Int. J. Biol. Sci. 14 (2), 178–188. 10.7150/ijbs.24074 29483836 PMC5821039

[B41] Wu B. Ren X. (2009). Promoting axonal myelination for improving neurological recovery in spinal cord injury. J. Neurotrauma 26 (10), 1847–1856. 10.1089/neu.2008.0551 19785544

[B42] Xia P. Gao X. Duan L. Zhang W. Sun Y. F. (2018). Mulberrin (Mul) reduces spinal cord injury (SCI)-induced apoptosis, inflammation and oxidative stress in rats via miroRNA-337 by targeting Nrf-2. Biomed. Pharmacother. 107, 1480–1487. 10.1016/j.biopha.2018.07.082 30257365

[B43] Xu K. Malouf A. T. Messing A. Silver J. (1999). Glial fibrillary acidic protein is necessary for mature astrocytes to react to beta-amyloid. Glia 25 (4), 390–403. 10.1002/(sici)1098-1136(19990215)25:4<390::aid-glia8>3.0.co;2-7 10028921

[B44] Xu Y. Tang D. Wang J. Wei H. Gao J. (2019). Neuroprotection of andrographolide against microglia-mediated inflammatory injury and oxidative damage in PC12 neurons. Neurochem. Res. 44 (11), 2619–2630. 10.1007/s11064-019-02883-5 31562575

[B45] Yang H. Liu C. Wang C. Zhang Q. An J. Zhang L. (2016). Therapeutical strategies for spinal cord injury and a promising autologous astrocyte-based therapy using efficient reprogramming techniques. Mol. Neurobiol. 53 (5), 2826–2842. 10.1007/s12035-015-9157-7 25863960

[B46] Yang R. Liu S. Zhou J. Bu S. Zhang J. (2017). Andrographolide attenuates microglia-mediated Aβ neurotoxicity partially through inhibiting NF-κB and JNK MAPK signaling pathway. Immunopharmacol. Immunotoxicol. 39 (5), 276–284. 10.1080/08923973.2017.1344989 28669260

[B47] Zeng B. Wei A. Zhou Q. Yuan M. Lei K. Liu Y. (2022). Andrographolide: A review of its pharmacology, pharmacokinetics, toxicity and clinical trials and pharmaceutical researches. Phytother. Res. 36 (1), 336–364. 10.1002/ptr.7324 34818697

[B48] Zeng H. Liu N. Yang Y. Y. Xing H. Y. Liu X. X. Li F. (2019). Lentivirus-mediated downregulation of alpha-synuclein reduces neuroinflammation and promotes functional recovery in rats with spinal cord injury. J. Neuroinflammation 16 (1), 283. 10.1186/s12974-019-1658-2 31888724 PMC6936070

[B49] Zhan J. Li X. Luo D. Hou Y. Hou Y. Chen S. (2020). Polydatin promotes the neuronal differentiation of bone marrow mesenchymal stem cells *in vitro* and *in vivo*: Involvement of Nrf2 signalling pathway. J. Cell. Mol. Med. 24 (9), 5317–5329. 10.1111/jcmm.15187 32299154 PMC7205798

[B50] Zhang H. Wang W. Du Q. (2019). Andrographolide attenuates bupivacaine-induced cytotoxicity in SH-SY5Y cells through preserving Akt/mTOR activity. Drug Des. devel. Ther. 13, 1659–1666. 10.2147/DDDT.S201122 PMC6529178 31190744

[B51] Zhang J. Zheng Y. Zhao Y. Zhang Y. Liu Y. Ma F. (2021). Andrographolide ameliorates neuroinflammation in APP/PS1 transgenic mice. Int. Immunopharmacol. 96, 107808. 10.1016/j.intimp.2021.107808 34162168

[B52] Zhang Z. Shi L. (2010). Anti-inflammatory and analgesic properties of cis-mulberroside A from Ramulus mori. Fitoterapia 81 (3), 214–218. 10.1016/j.fitote.2009.09.005 19755140

[B53] Zhao X. Zhao X. Wang Z. (2021). Synergistic neuroprotective effects of hyperbaric oxygen and N-acetylcysteine against traumatic spinal cord injury in rat. J. Chem. Neuroanat. 118, 102037. 34601074 10.1016/j.jchemneu.2021.102037

