# Supplementary figures and images for: Andrographolide contributes to spinal cord injury repair via inhibition of apoptosis, oxidative stress and inflammation (part 2 of 2)
Source: Front Pharmacol. 2022 Oct 7;13:949502. doi: 10.3389/fphar.2022.949502 (PMC9585304; doi:10.3389/fphar.2022.949502)

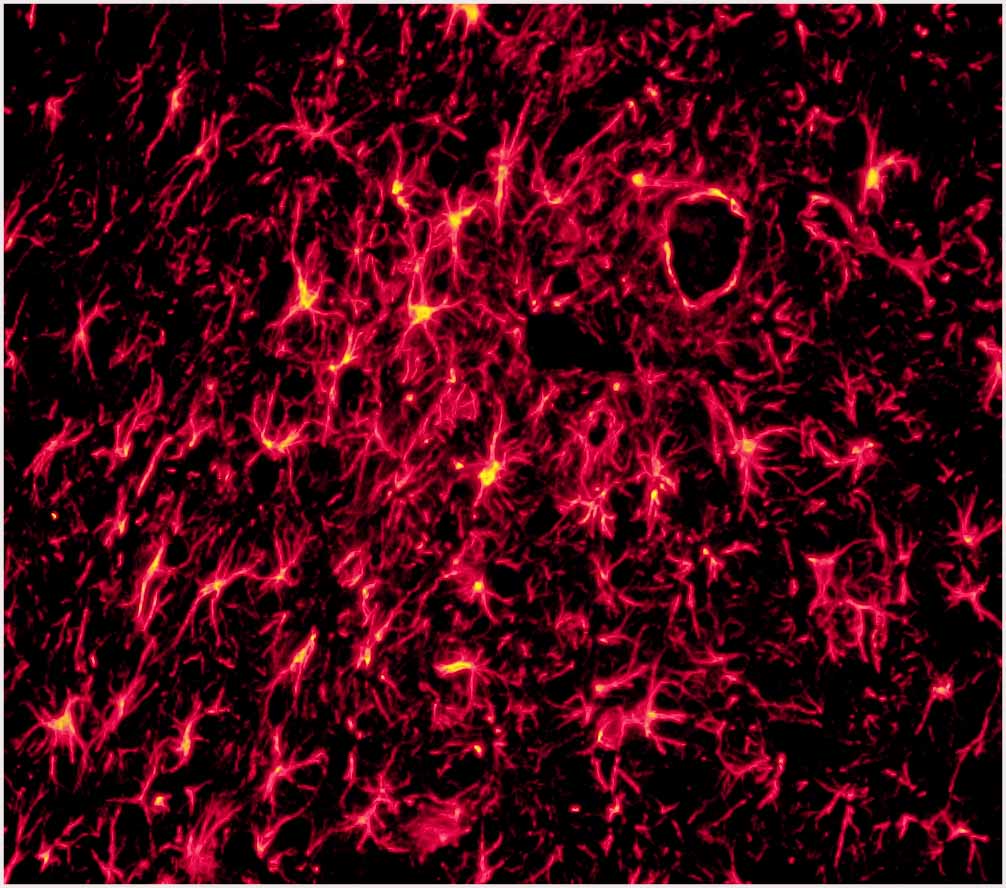

Supplement: Supplementary file 1 [file DataSheet1.ZIP › raw data/Fig.6/IF/SCI+NS/1.jpg]

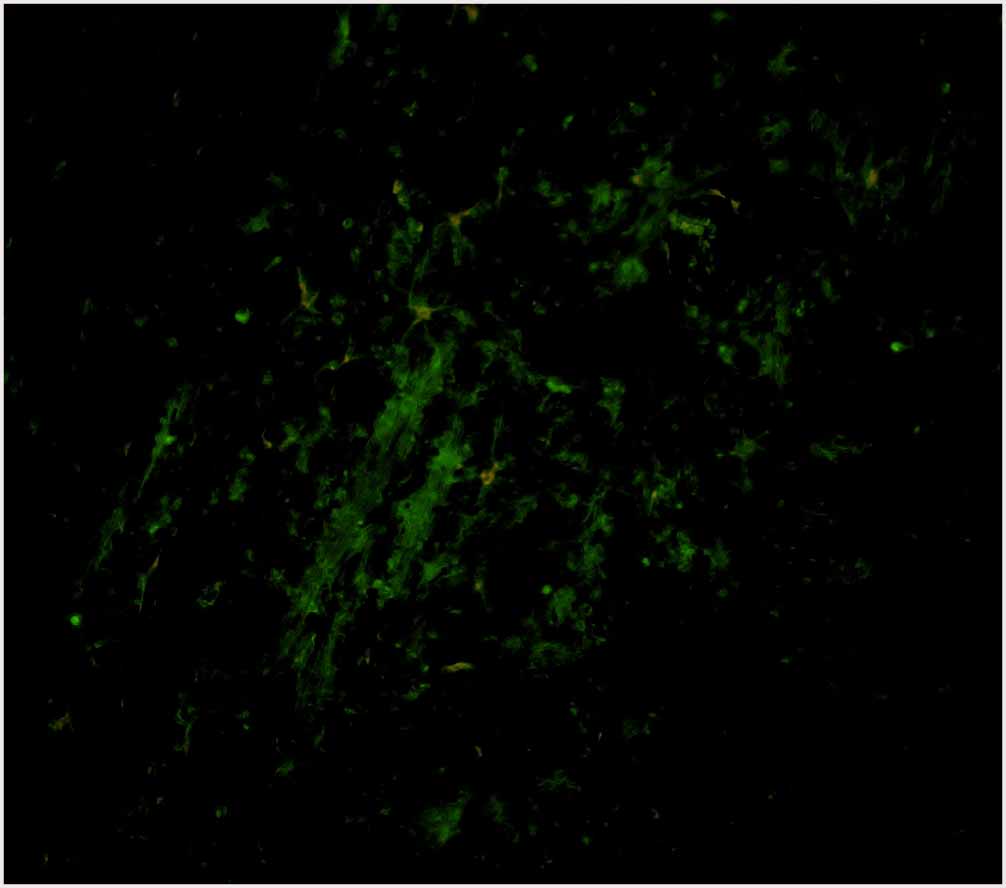

Supplement: Supplementary file 1 [file DataSheet1.ZIP › raw data/Fig.6/IF/SCI+NS/2.jpg]

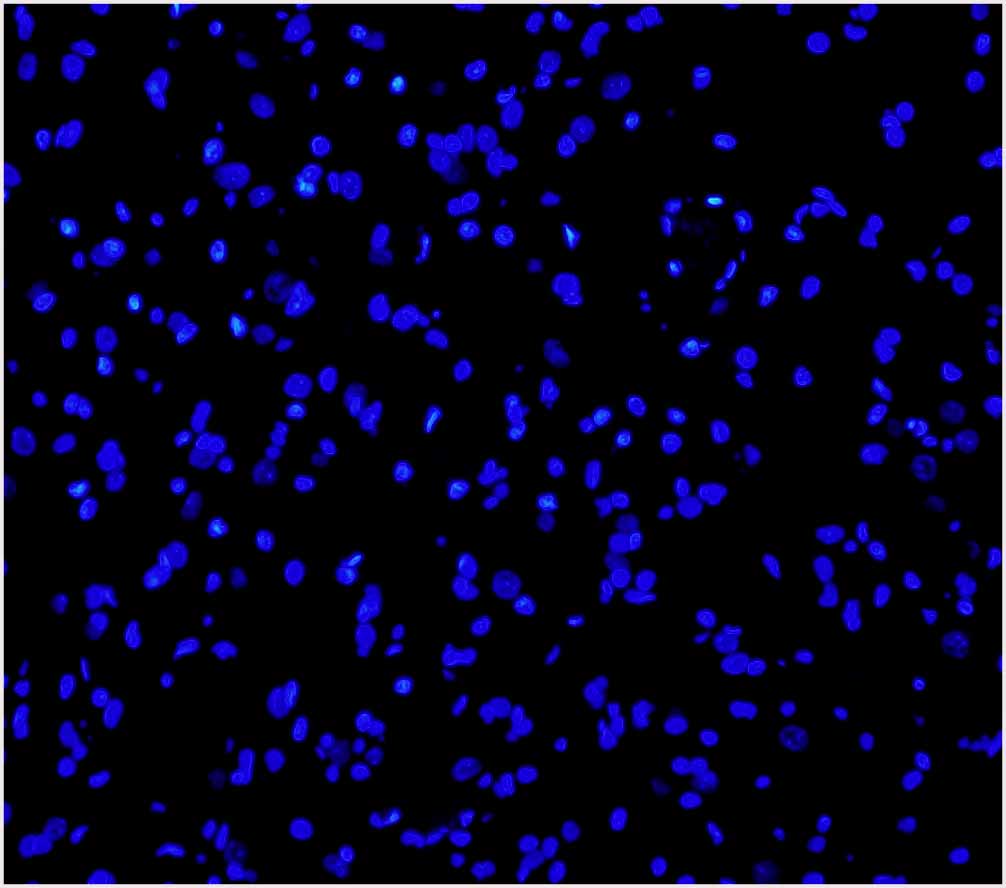

Supplement: Supplementary file 1 [file DataSheet1.ZIP › raw data/Fig.6/IF/SCI+NS/3.jpg]

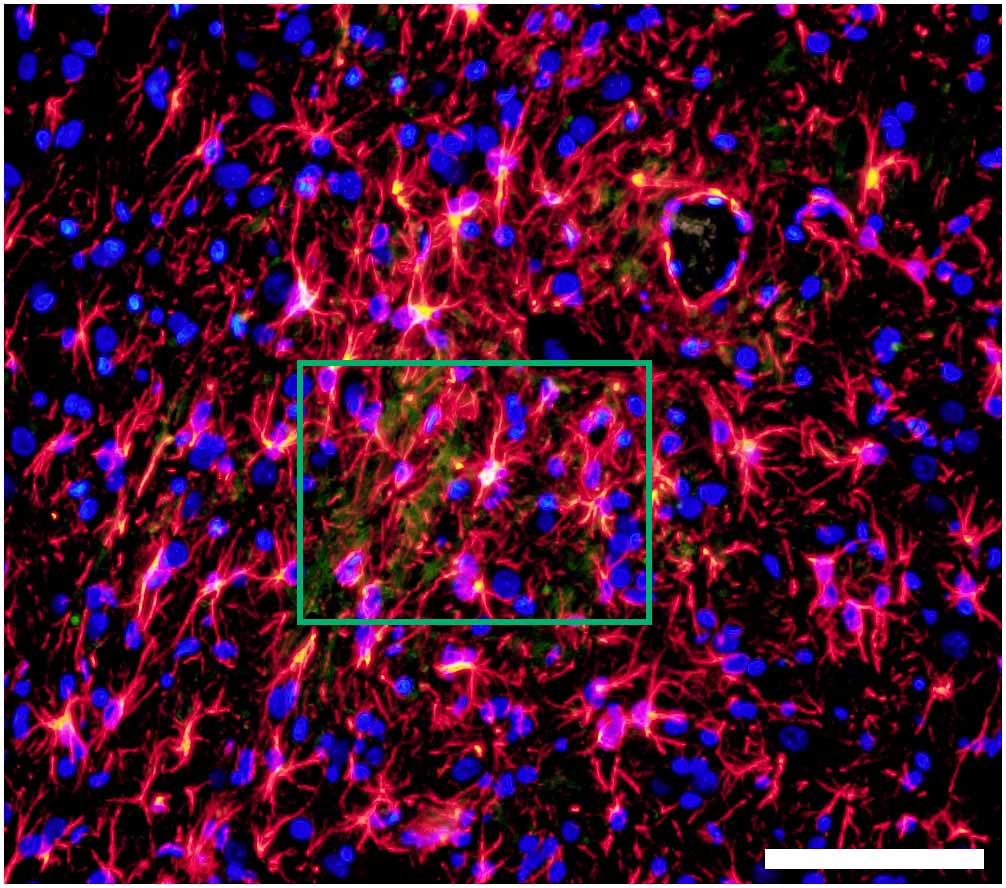

Supplement: Supplementary file 1 [file DataSheet1.ZIP › raw data/Fig.6/IF/SCI+NS/4.jpg]

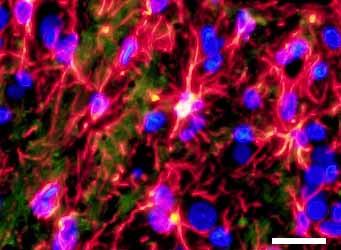

Supplement: Supplementary file 1 [file DataSheet1.ZIP › raw data/Fig.6/IF/SCI+NS/5.jpg]

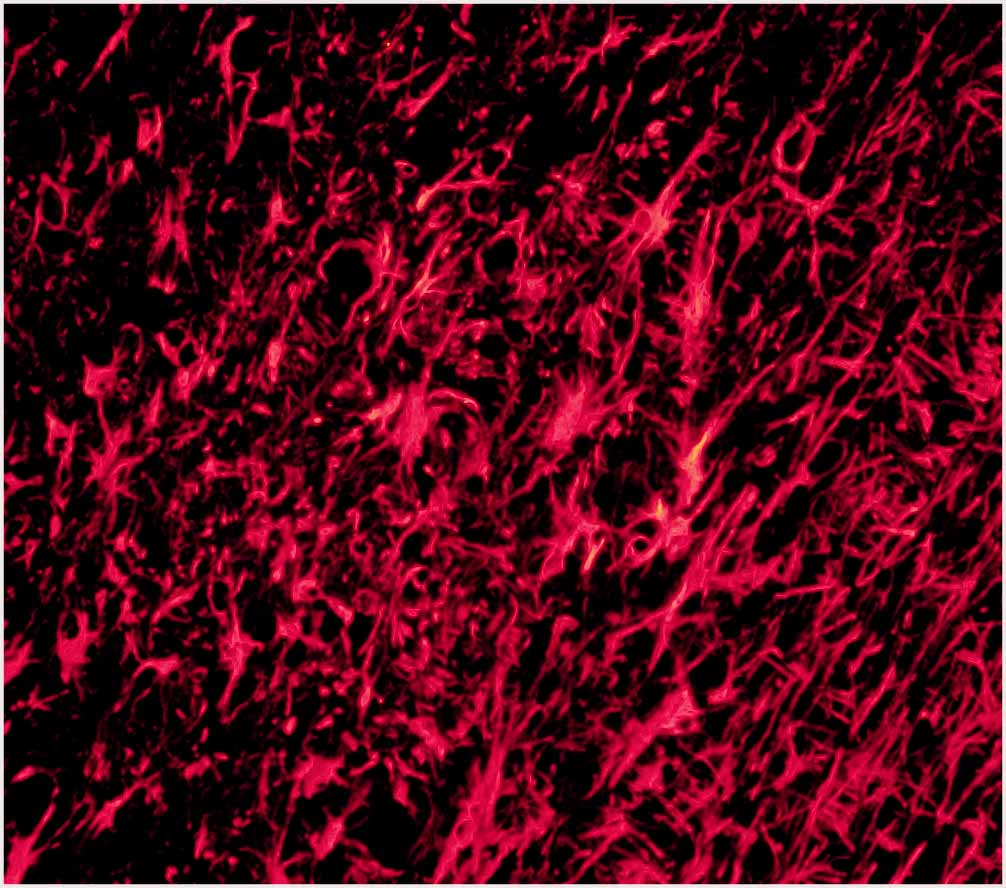

Supplement: Supplementary file 1 [file DataSheet1.ZIP › raw data/Fig.6/IF/SCI/1.jpg]

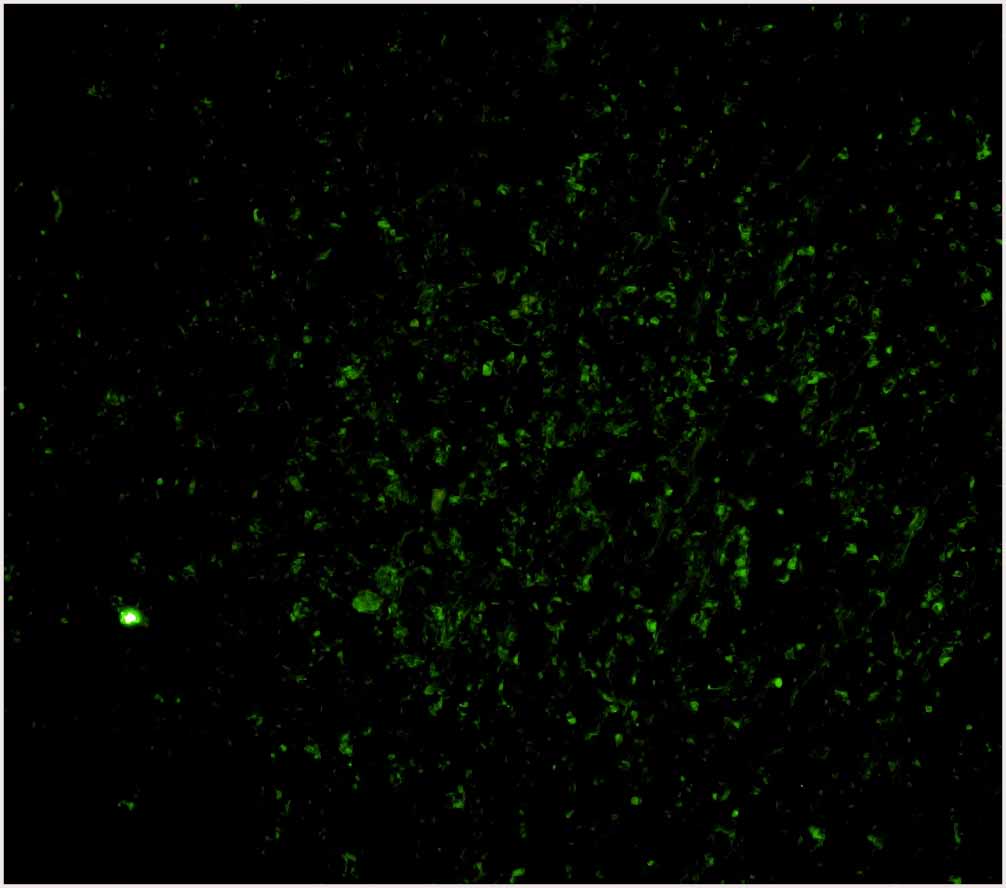

Supplement: Supplementary file 1 [file DataSheet1.ZIP › raw data/Fig.6/IF/SCI/2.jpg]

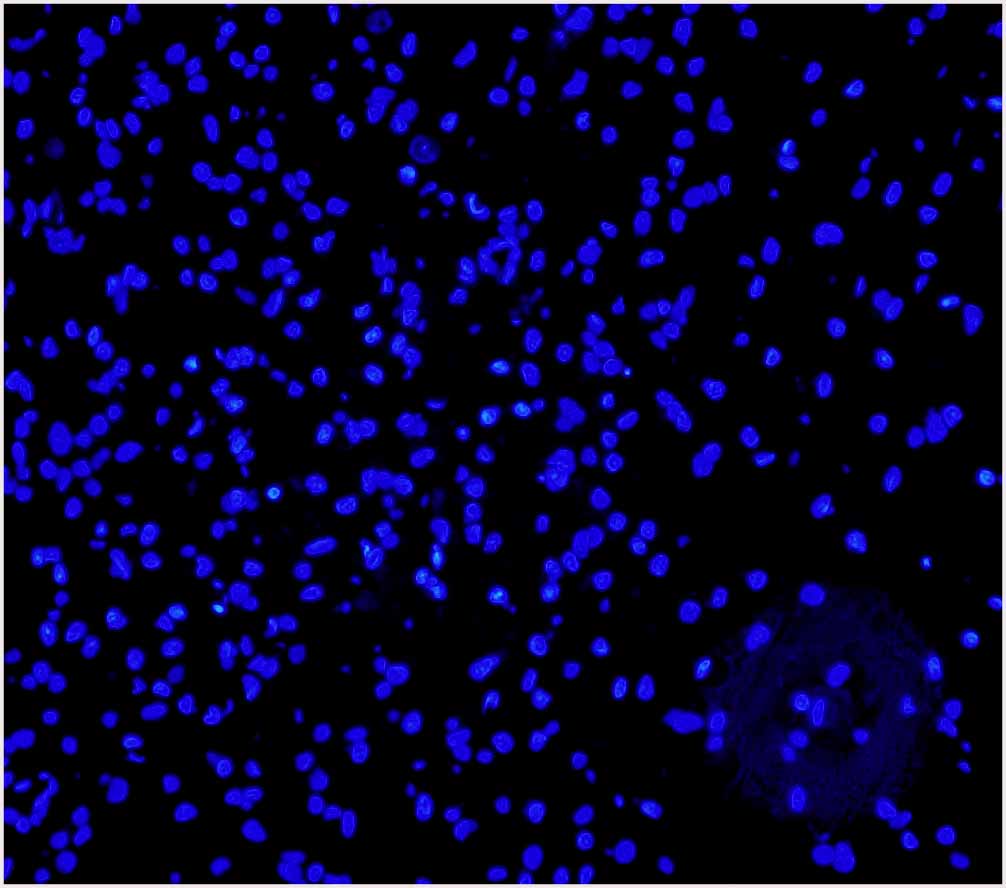

Supplement: Supplementary file 1 [file DataSheet1.ZIP › raw data/Fig.6/IF/SCI/3.jpg]

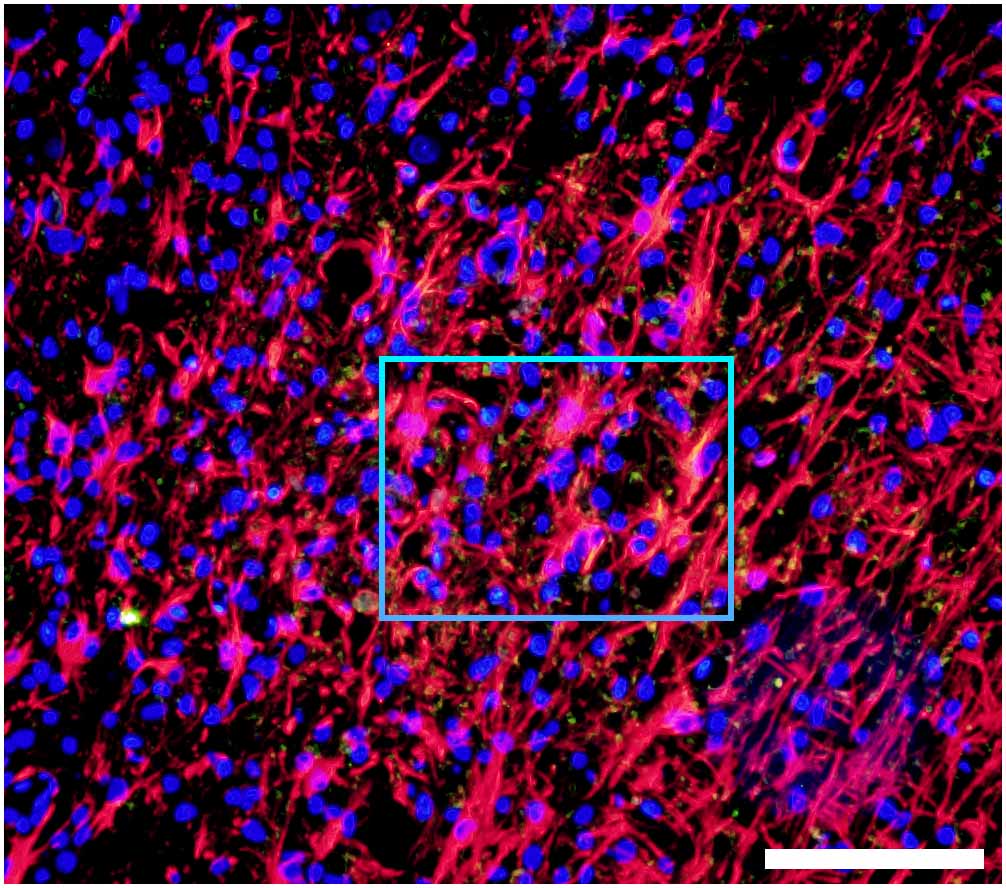

Supplement: Supplementary file 1 [file DataSheet1.ZIP › raw data/Fig.6/IF/SCI/4.jpg]

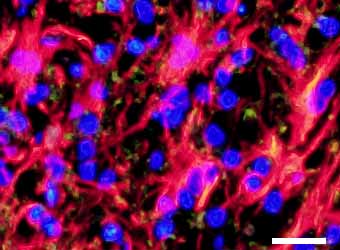

Supplement: Supplementary file 1 [file DataSheet1.ZIP › raw data/Fig.6/IF/SCI/5.jpg]

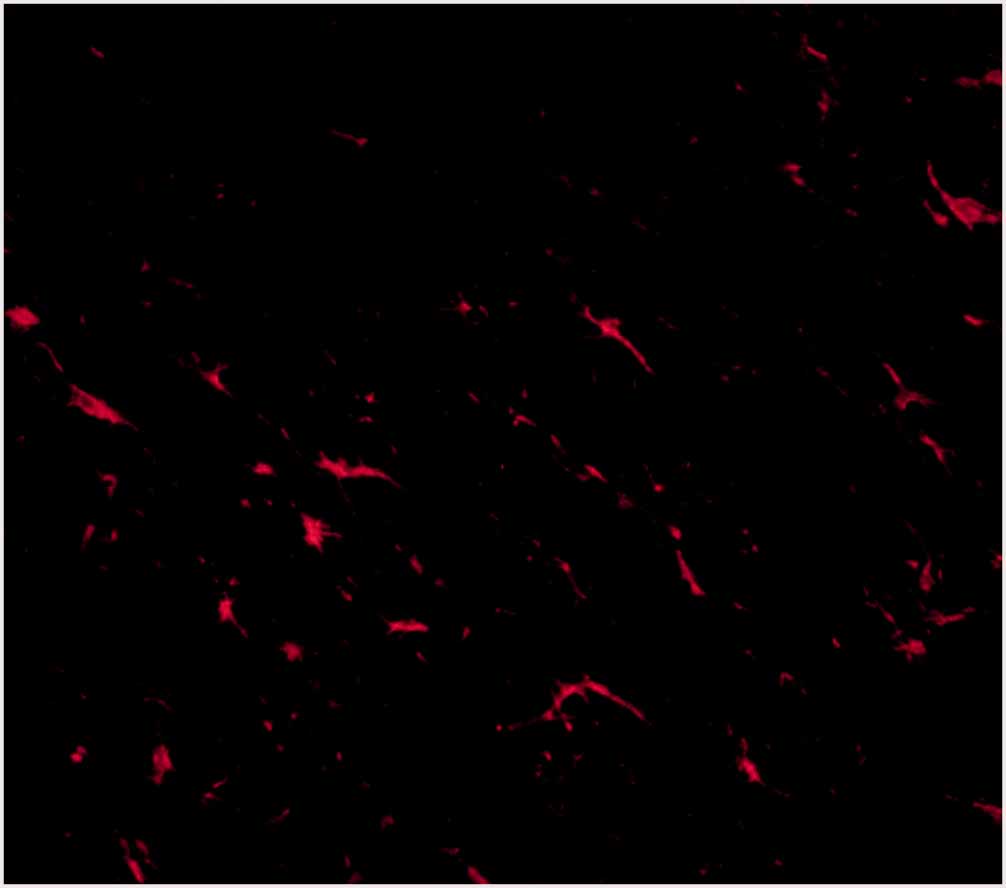

Supplement: Supplementary file 1 [file DataSheet1.ZIP › raw data/Fig.6/IF/Sham/1-.jpg]

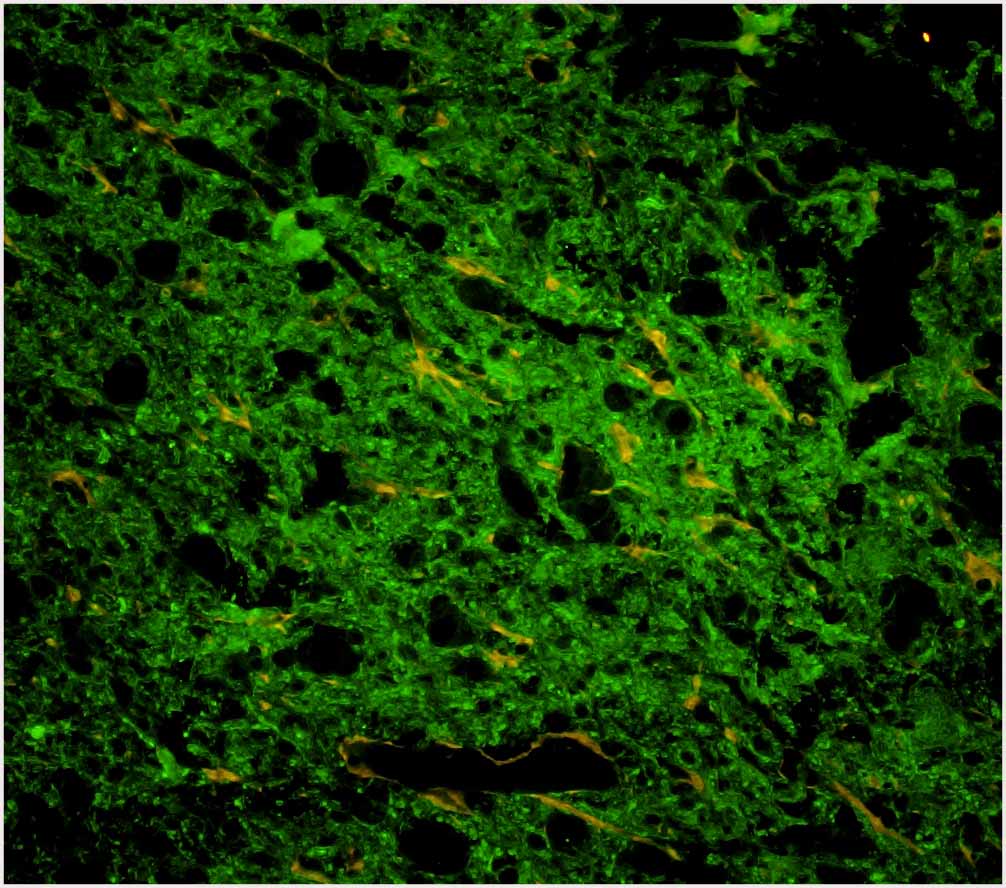

Supplement: Supplementary file 1 [file DataSheet1.ZIP › raw data/Fig.6/IF/Sham/2-.jpg]

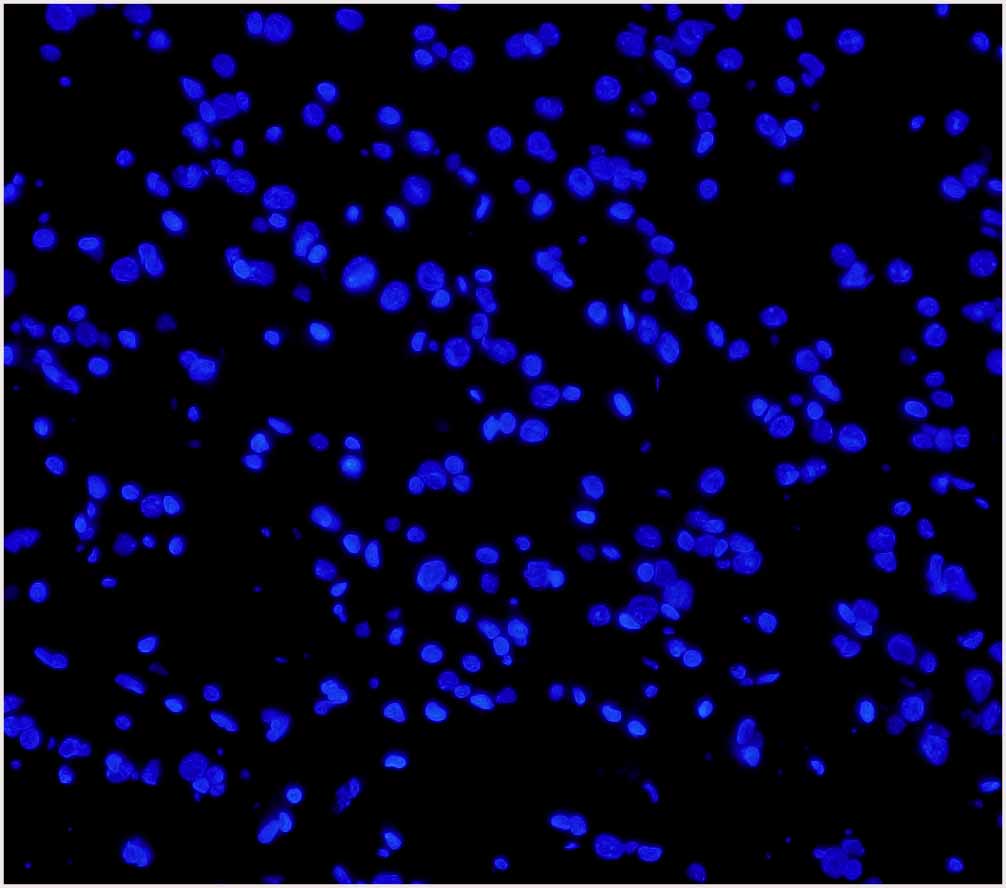

Supplement: Supplementary file 1 [file DataSheet1.ZIP › raw data/Fig.6/IF/Sham/3-.jpg]

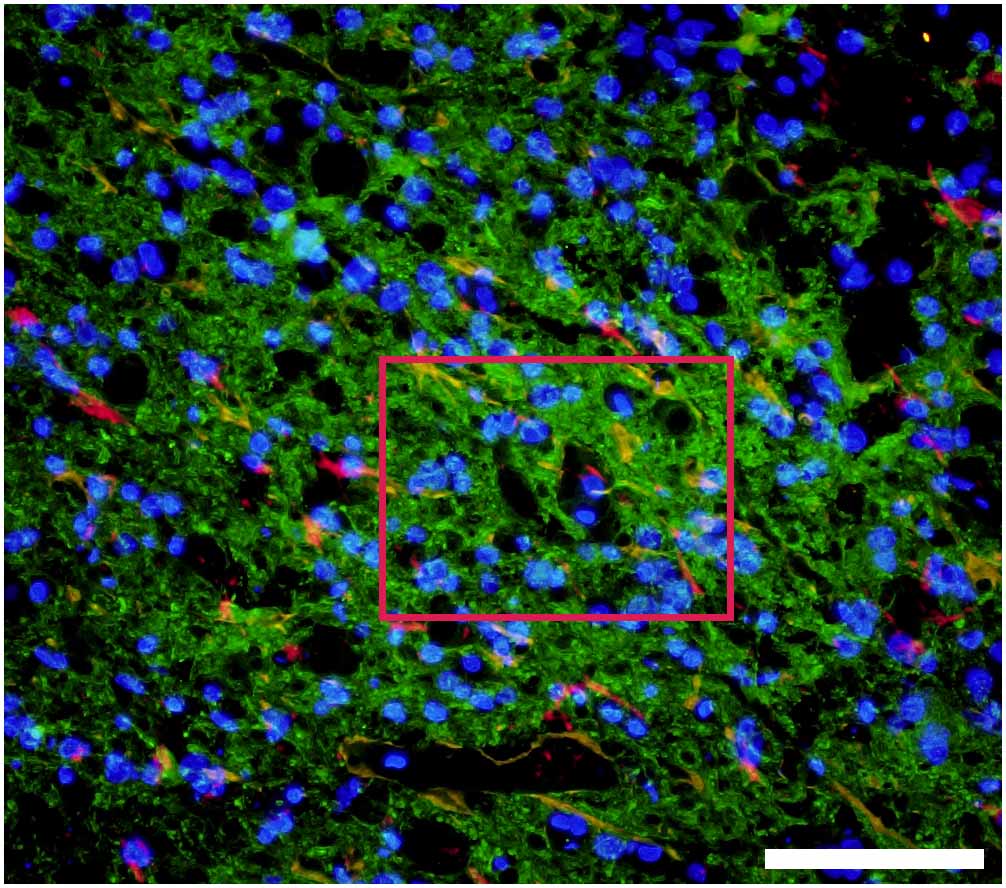

Supplement: Supplementary file 1 [file DataSheet1.ZIP › raw data/Fig.6/IF/Sham/4-.jpg]

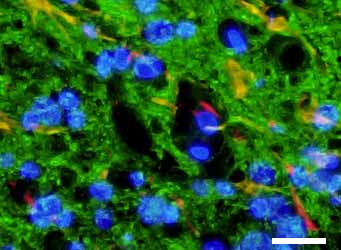

Supplement: Supplementary file 1 [file DataSheet1.ZIP › raw data/Fig.6/IF/Sham/5-.jpg]

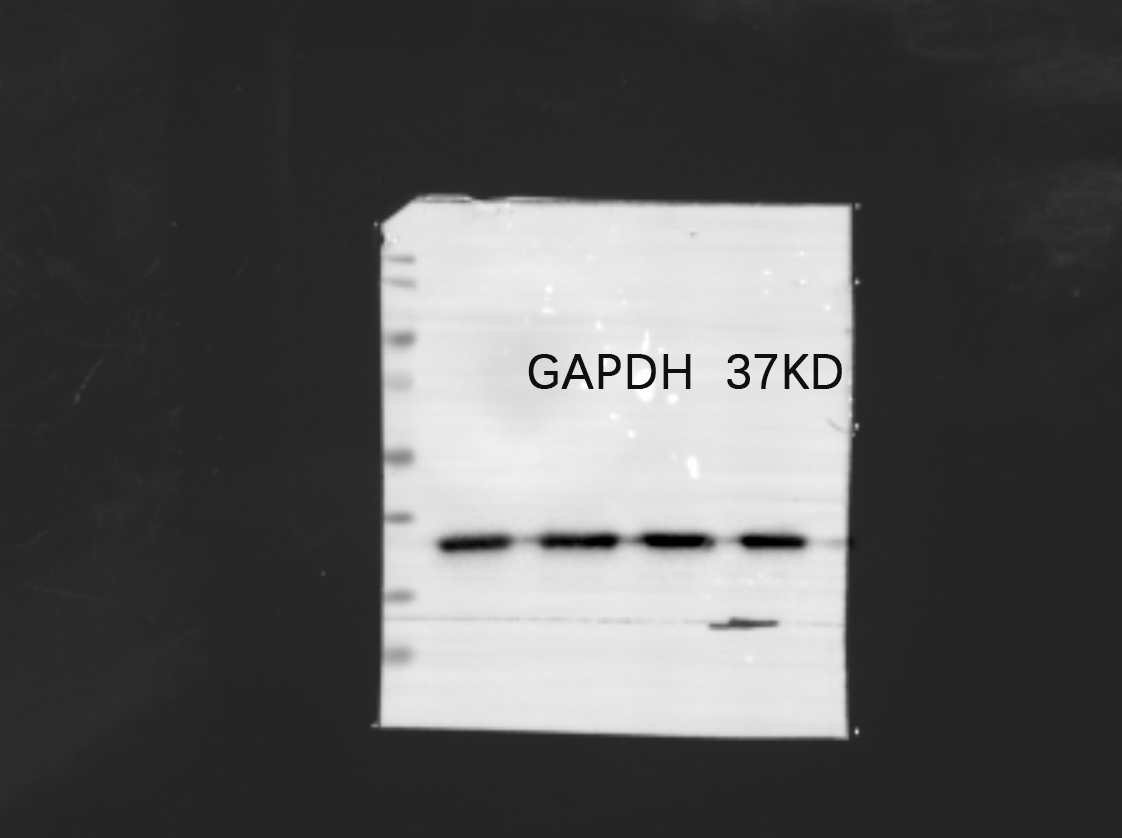

Supplement: Supplementary file 1 [file DataSheet1.ZIP › raw data/Fig.6/Western blot images/GAPDH.tif]

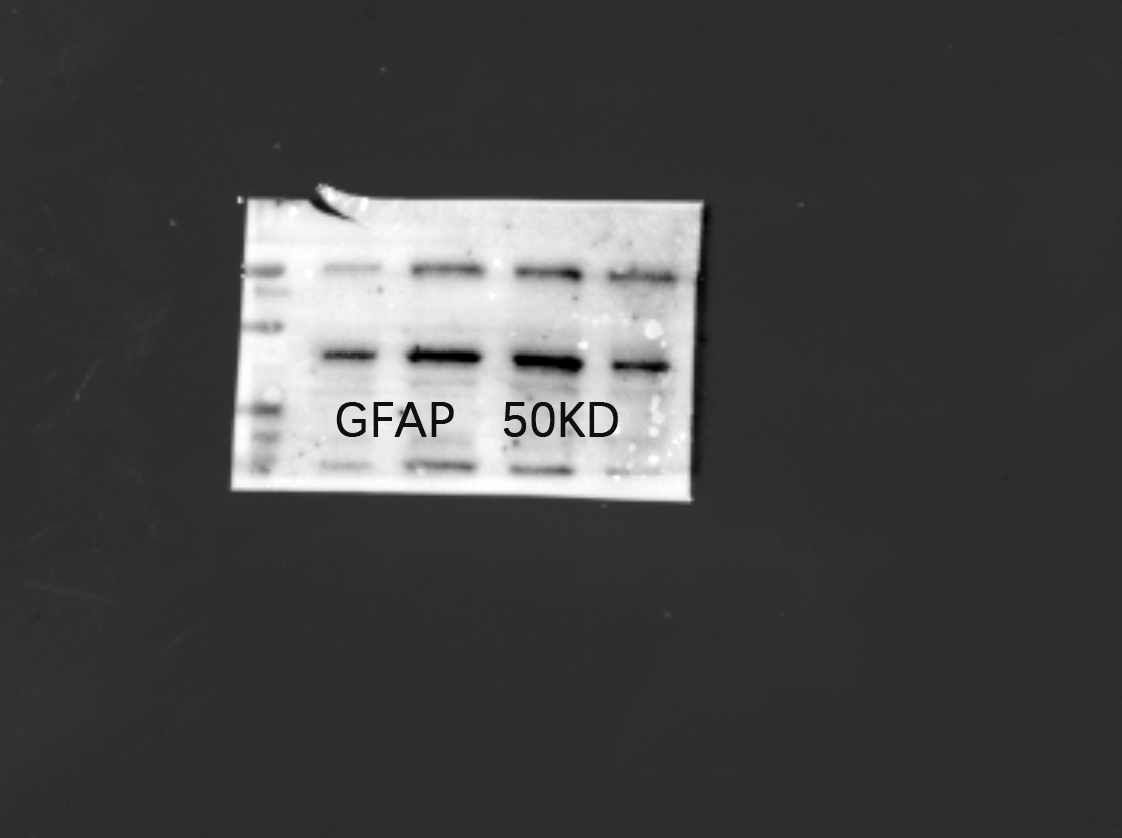

Supplement: Supplementary file 1 [file DataSheet1.ZIP › raw data/Fig.6/Western blot images/GFAP.tif]

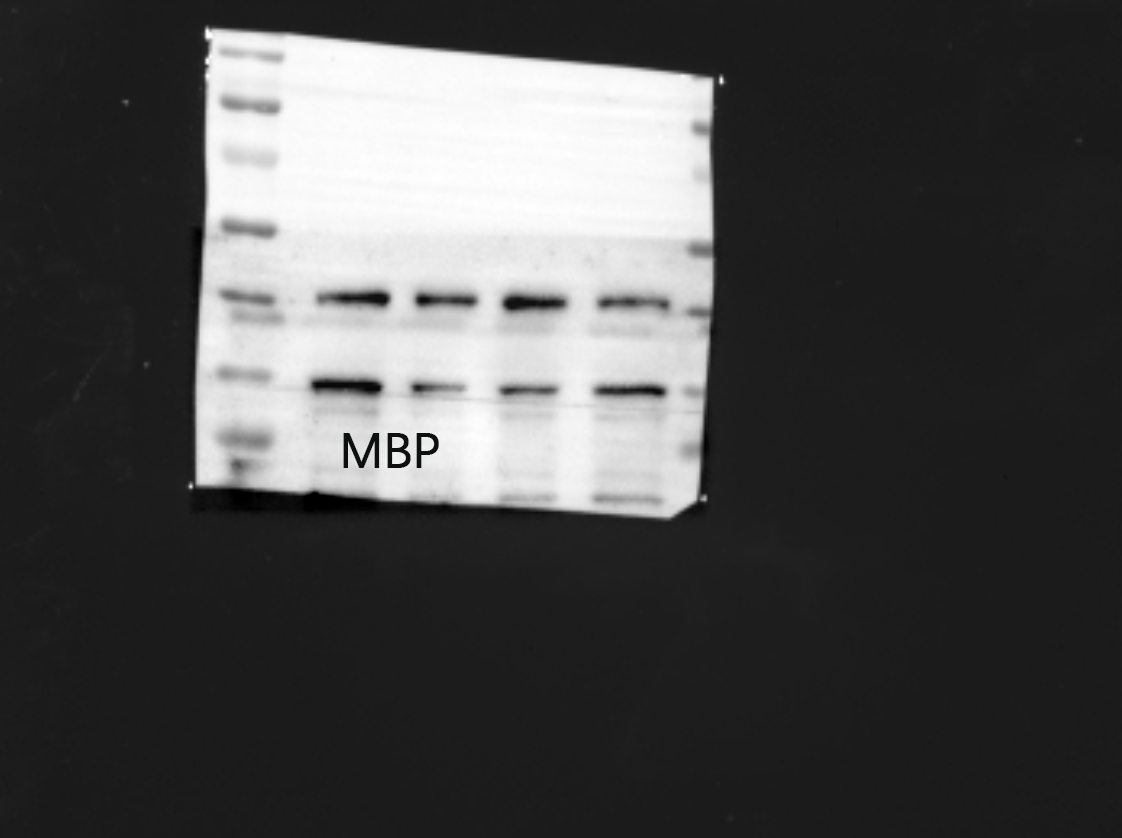

Supplement: Supplementary file 1 [file DataSheet1.ZIP › raw data/Fig.6/Western blot images/MBP.tif]

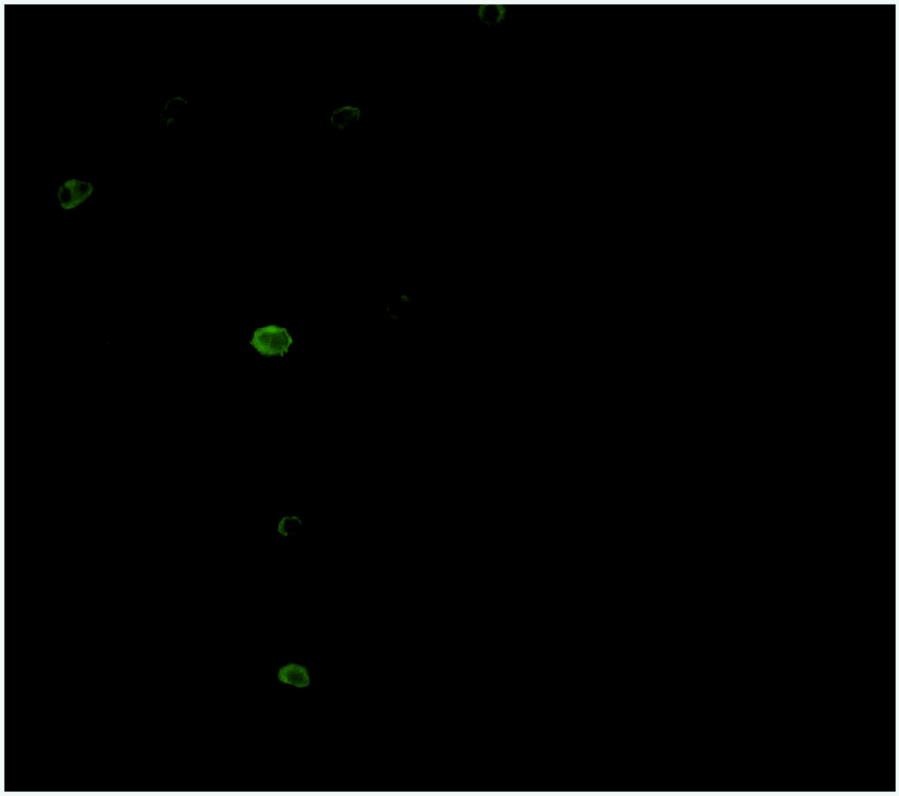

Supplement: Supplementary file 1 [file DataSheet1.ZIP › raw data/Fig.7/TUNEL/Control/1.tif]

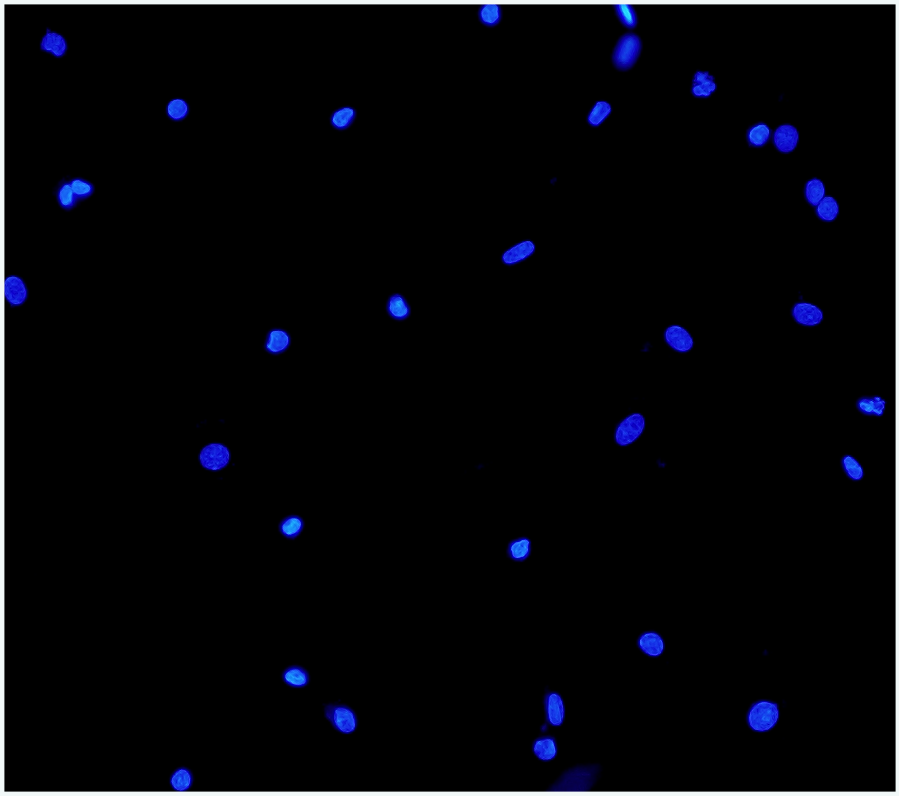

Supplement: Supplementary file 1 [file DataSheet1.ZIP › raw data/Fig.7/TUNEL/Control/2.tif]

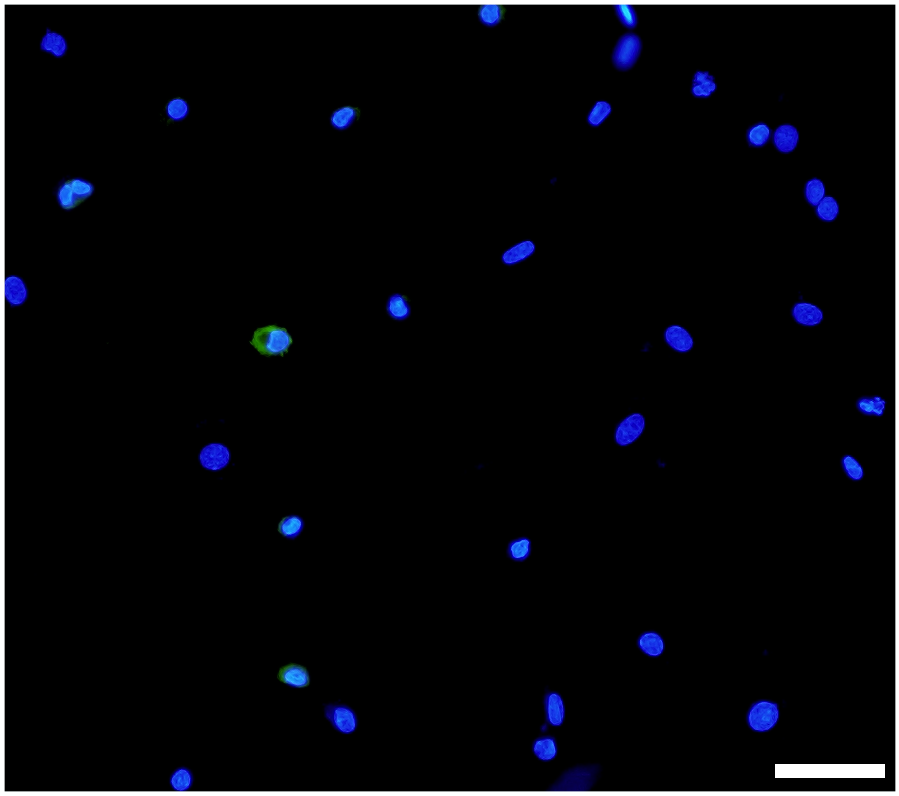

Supplement: Supplementary file 1 [file DataSheet1.ZIP › raw data/Fig.7/TUNEL/Control/3.tif]

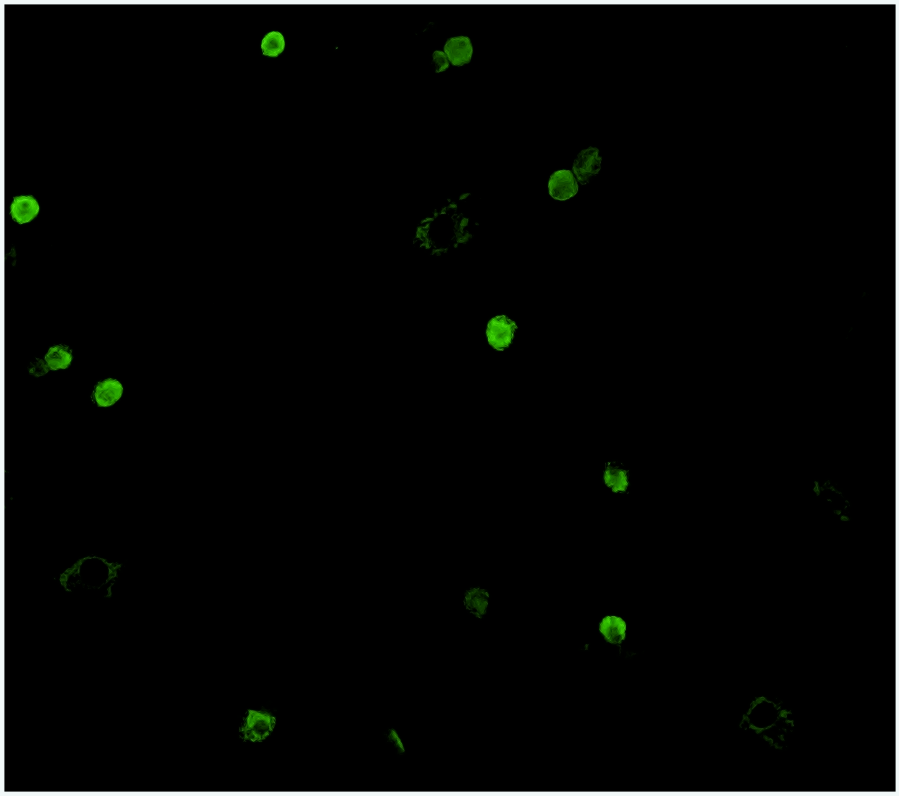

Supplement: Supplementary file 1 [file DataSheet1.ZIP › raw data/Fig.7/TUNEL/LPS+Andro/1.tif]

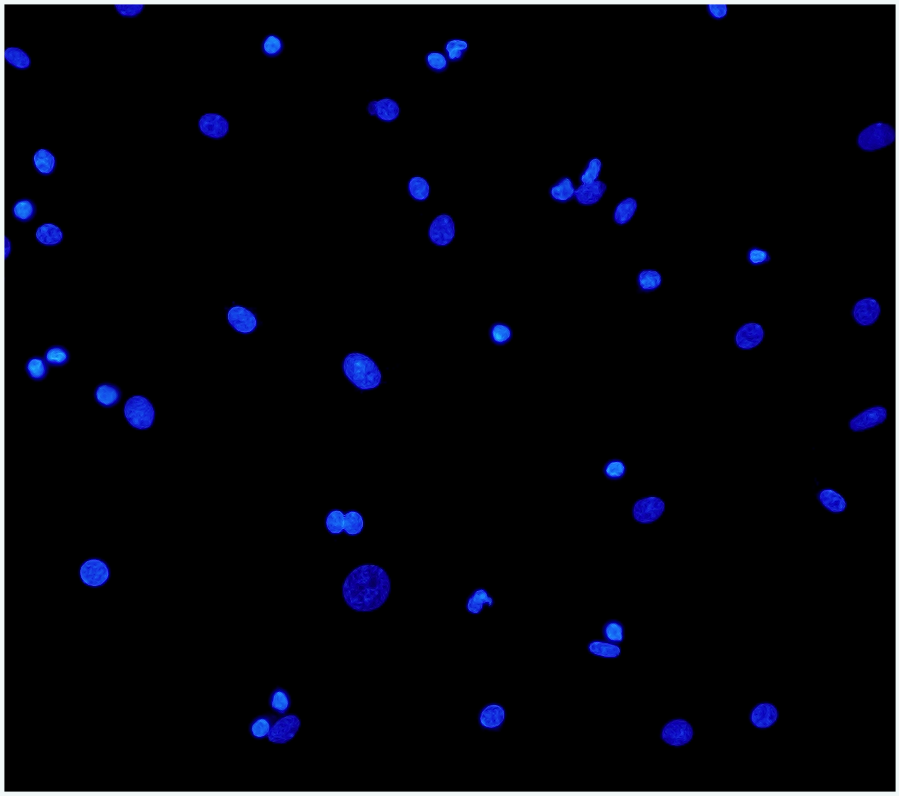

Supplement: Supplementary file 1 [file DataSheet1.ZIP › raw data/Fig.7/TUNEL/LPS+Andro/2.tif]

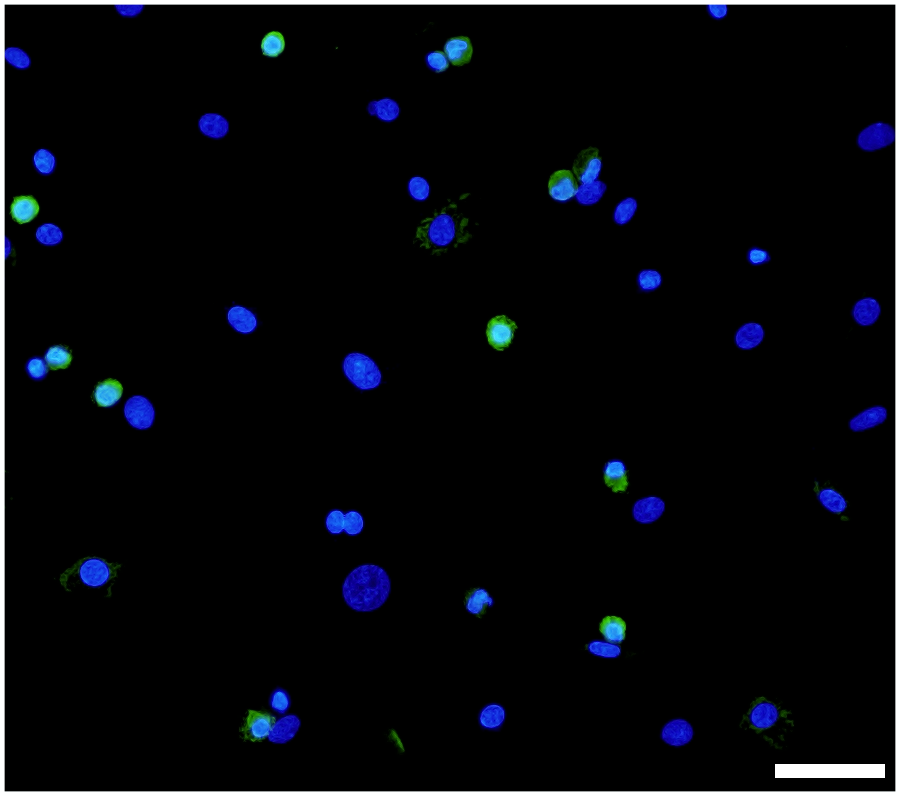

Supplement: Supplementary file 1 [file DataSheet1.ZIP › raw data/Fig.7/TUNEL/LPS+Andro/3.tif]

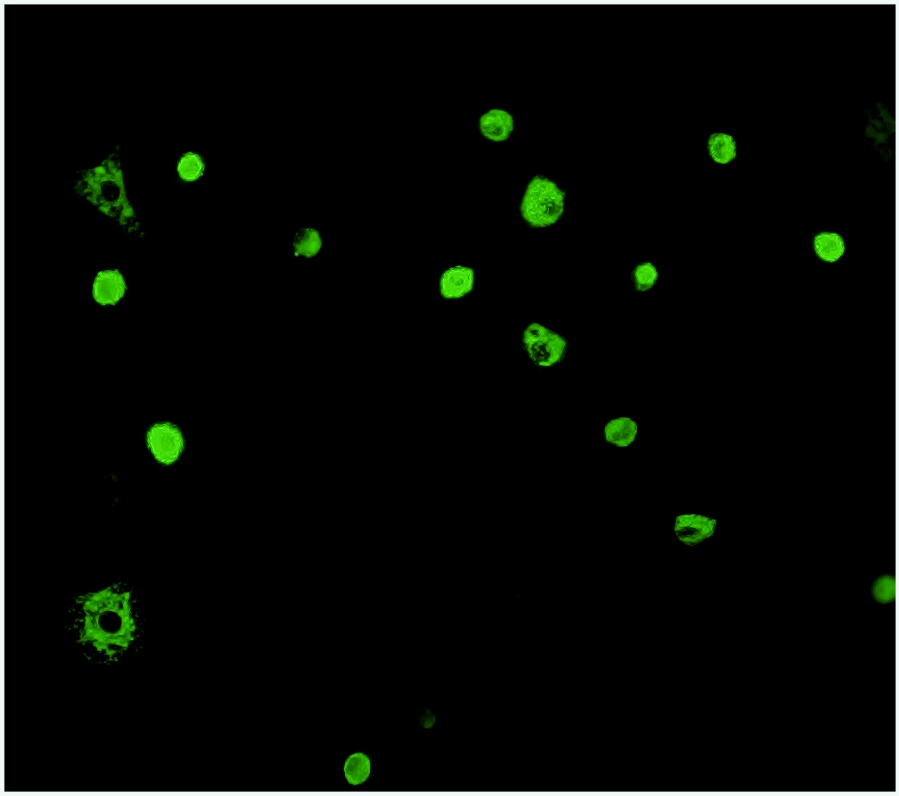

Supplement: Supplementary file 1 [file DataSheet1.ZIP › raw data/Fig.7/TUNEL/LPS/1.tif]

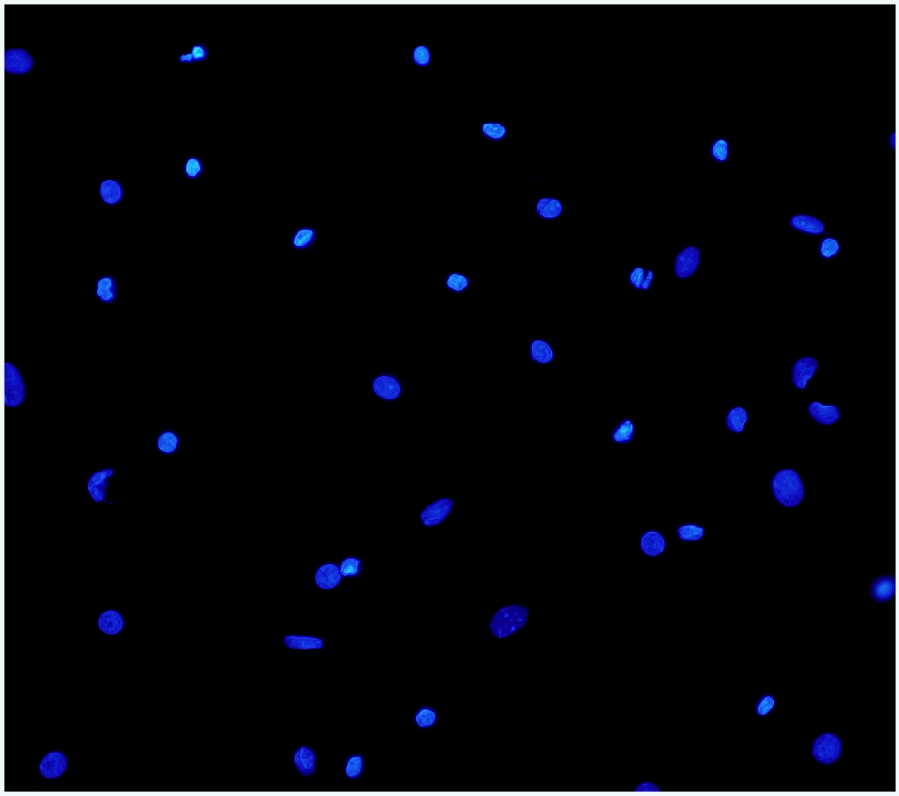

Supplement: Supplementary file 1 [file DataSheet1.ZIP › raw data/Fig.7/TUNEL/LPS/2.tif]

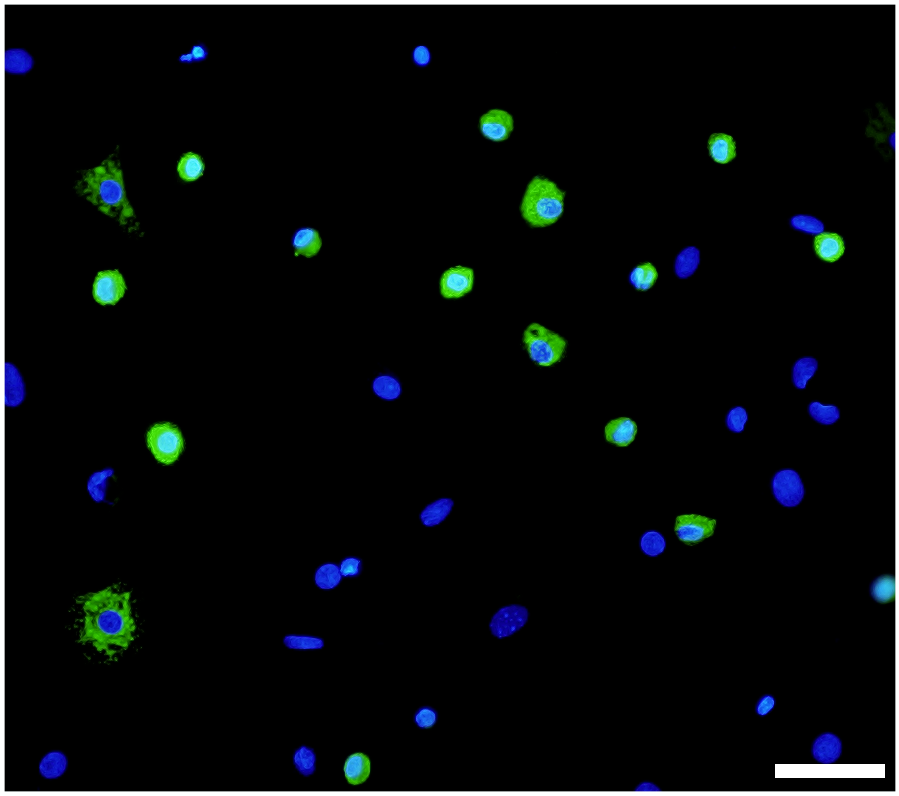

Supplement: Supplementary file 1 [file DataSheet1.ZIP › raw data/Fig.7/TUNEL/LPS/3.tif]

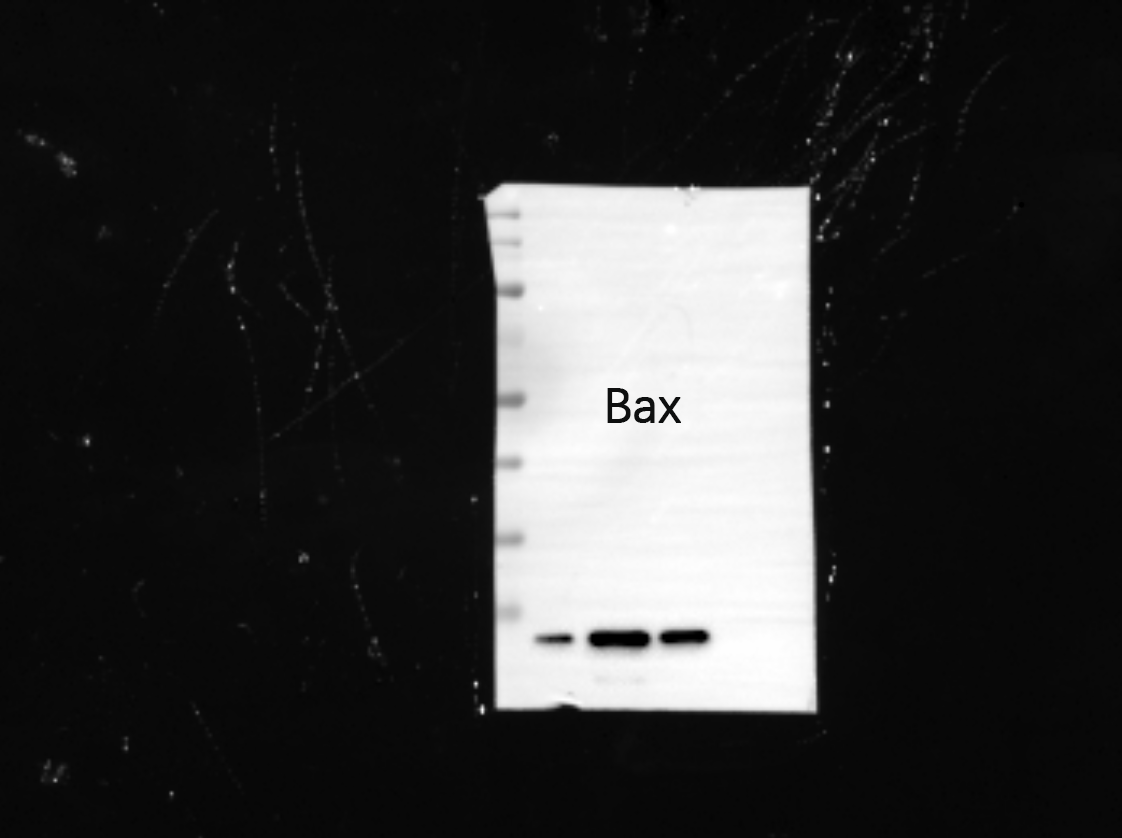

Supplement: Supplementary file 1 [file DataSheet1.ZIP › raw data/Fig.7/Western blot images/Bax.tif]

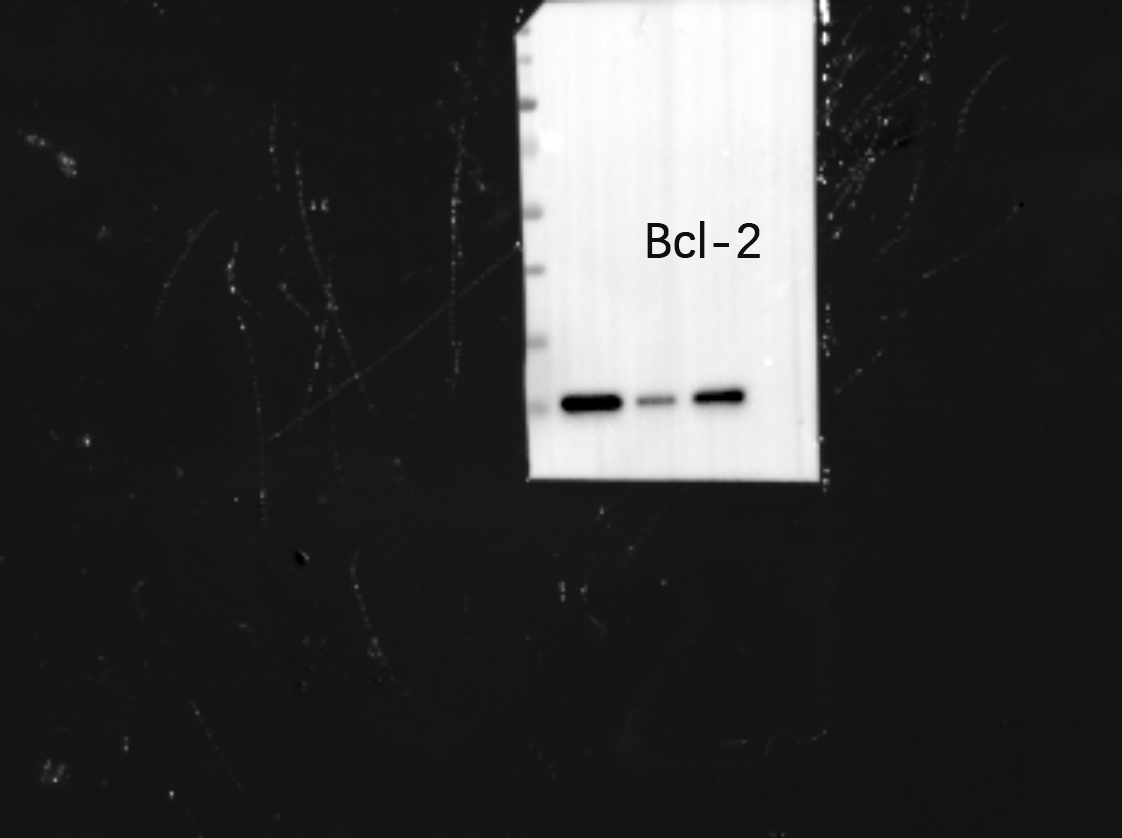

Supplement: Supplementary file 1 [file DataSheet1.ZIP › raw data/Fig.7/Western blot images/Bcl2.tif]

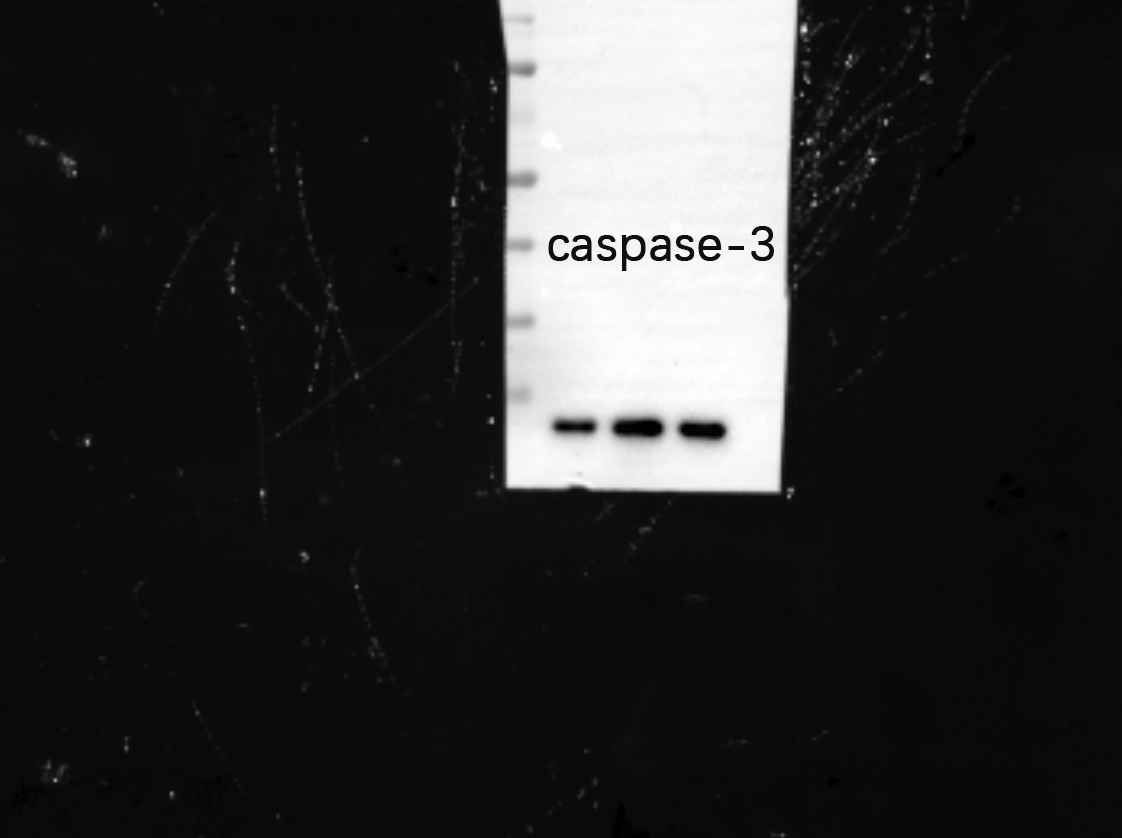

Supplement: Supplementary file 1 [file DataSheet1.ZIP › raw data/Fig.7/Western blot images/Cleaved caspase-3.tif]

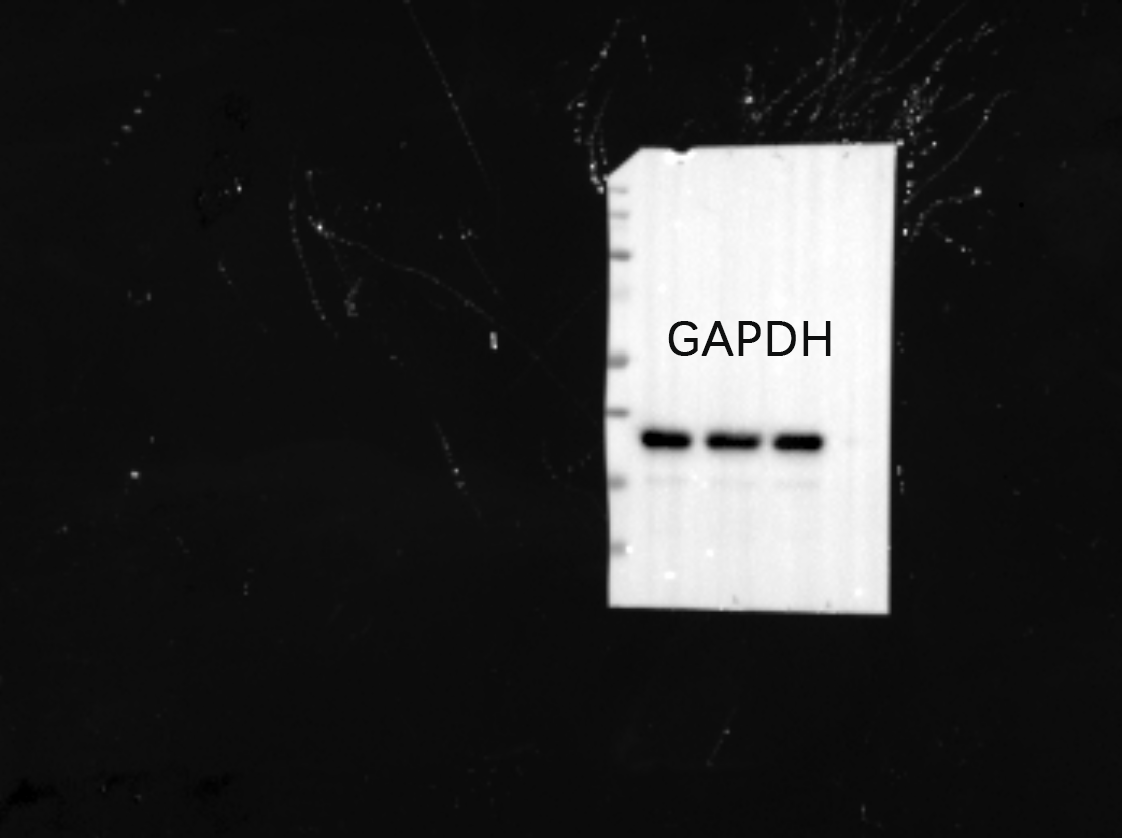

Supplement: Supplementary file 1 [file DataSheet1.ZIP › raw data/Fig.7/Western blot images/GAPDH.tif]
