# Supplementary figures and images for: Andrographolide contributes to spinal cord injury repair via inhibition of apoptosis, oxidative stress and inflammation (part 1 of 2)
Source: Front Pharmacol. 2022 Oct 7;13:949502. doi: 10.3389/fphar.2022.949502 (PMC9585304; doi:10.3389/fphar.2022.949502)

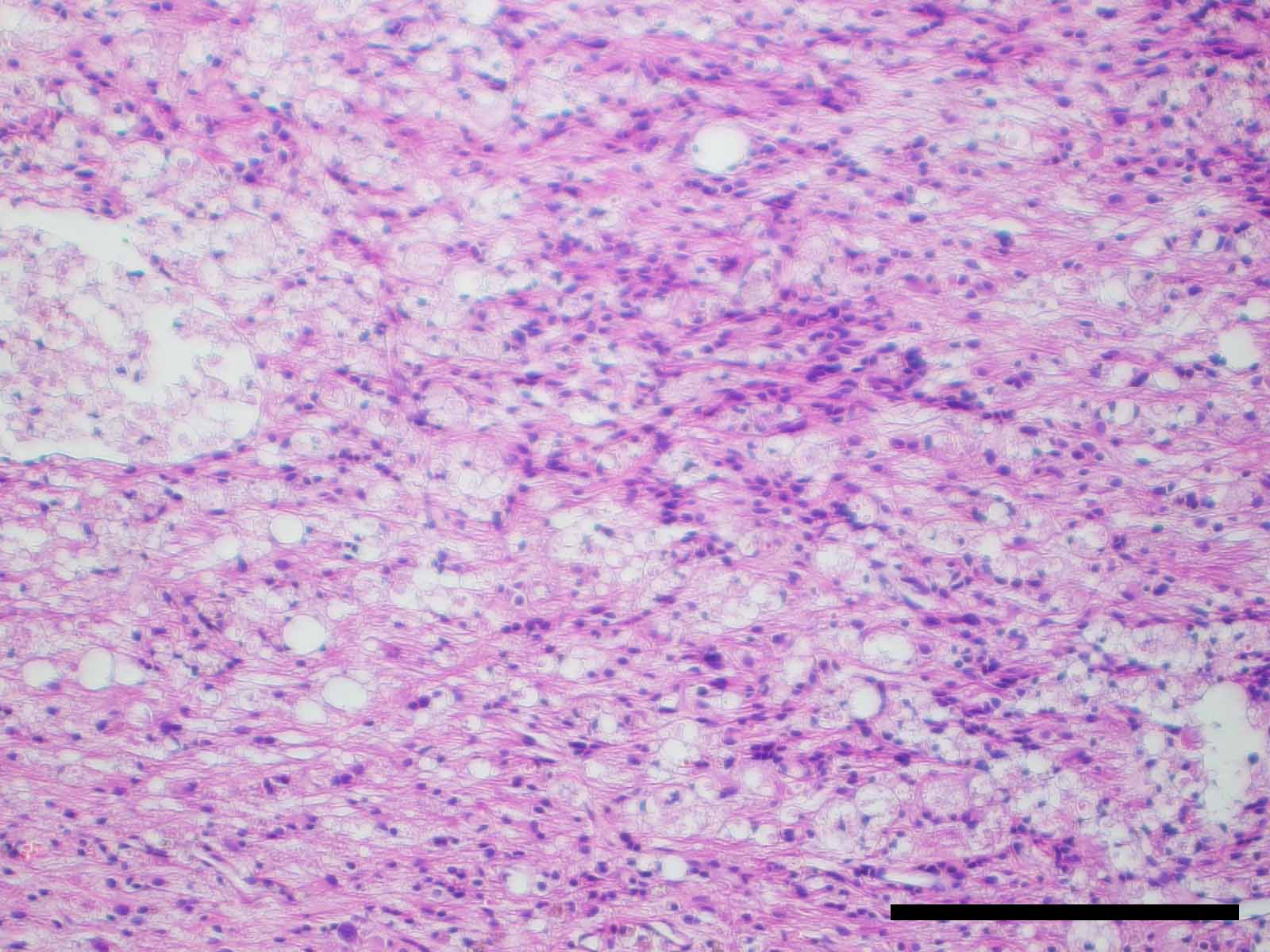

Supplement: Supplementary file 1 [file DataSheet1.ZIP › raw data/Fig.1/HE/SCI+Andro.jpg]

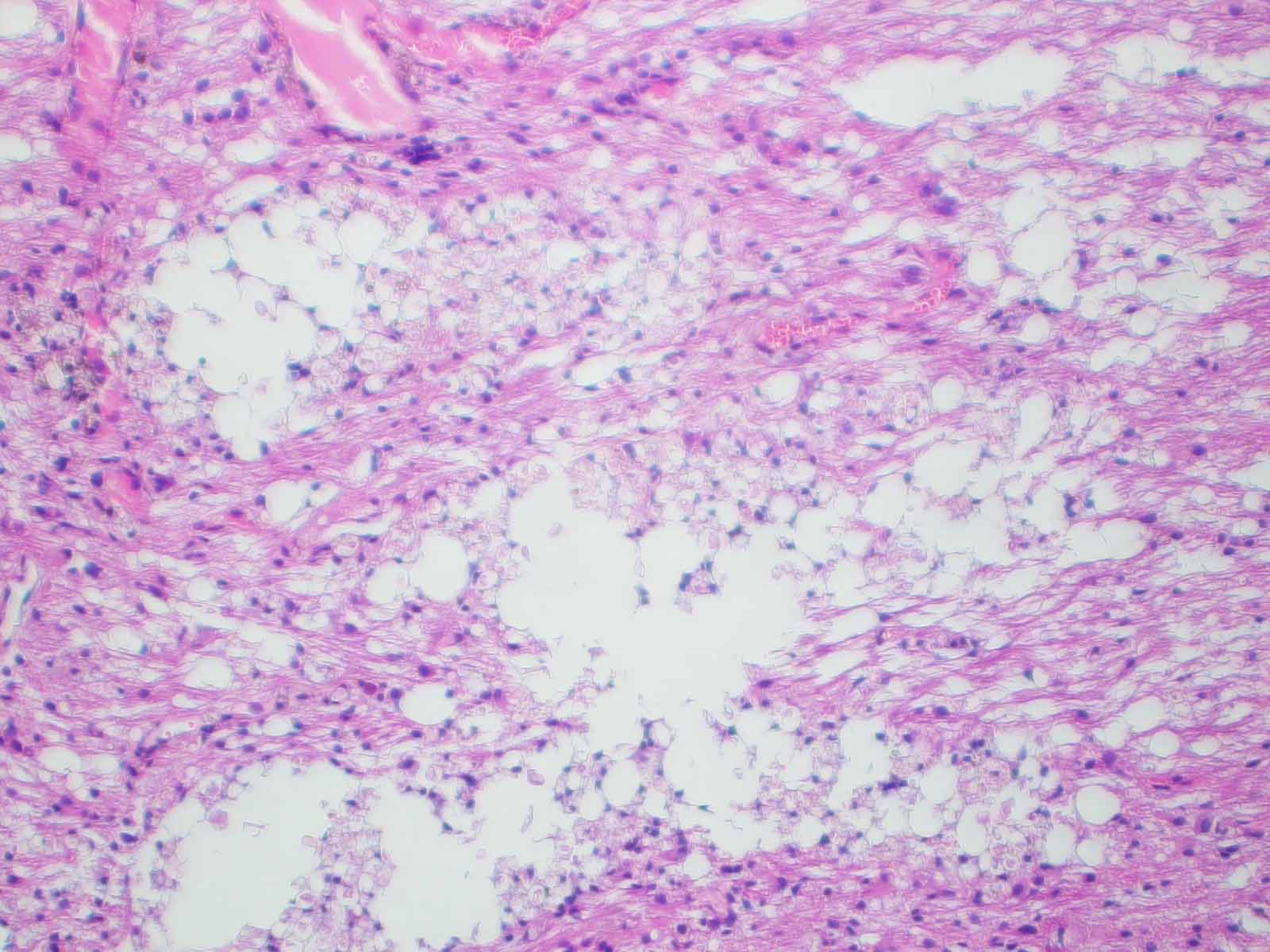

Supplement: Supplementary file 1 [file DataSheet1.ZIP › raw data/Fig.1/HE/SCI+NS.jpg]

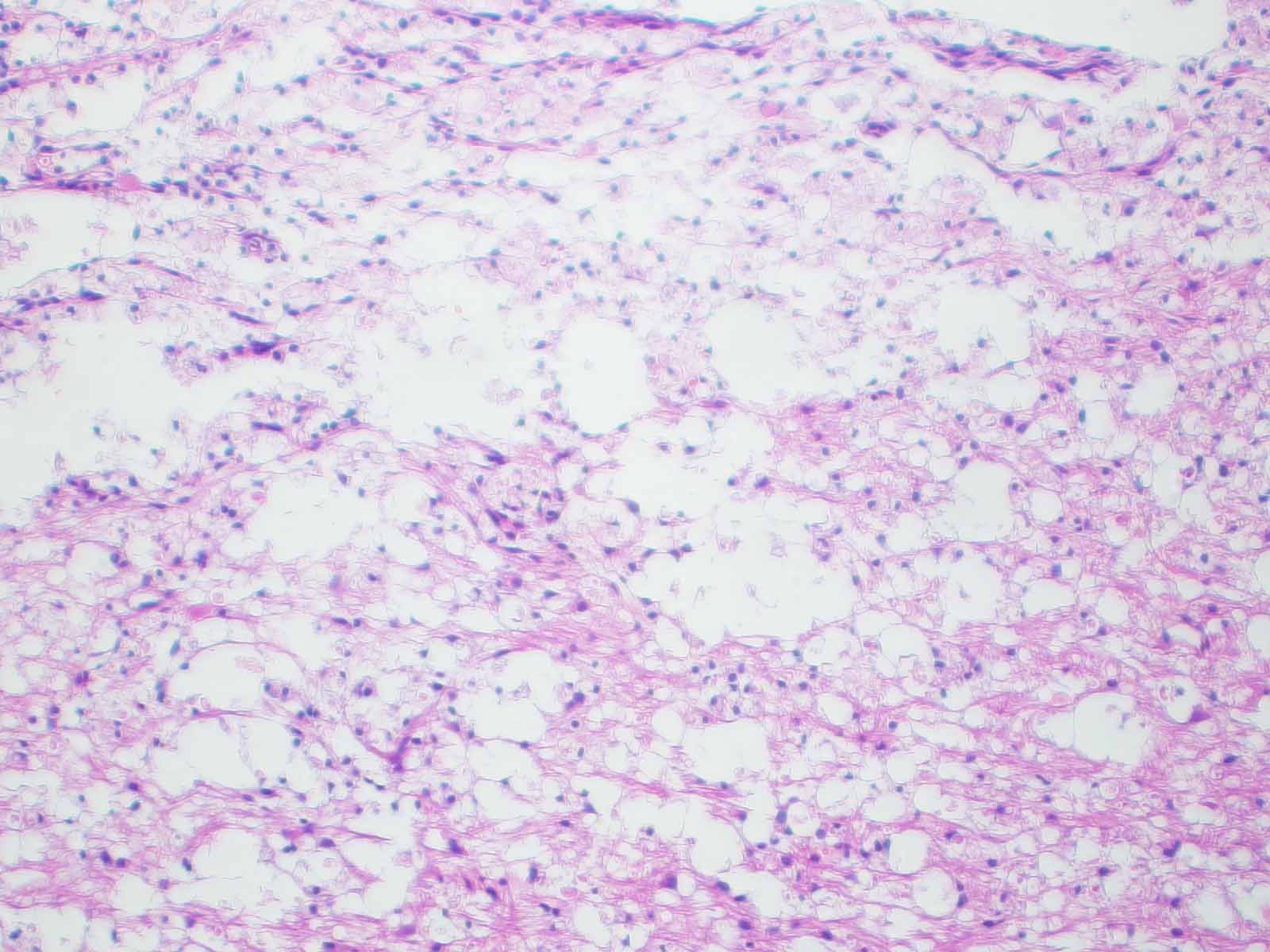

Supplement: Supplementary file 1 [file DataSheet1.ZIP › raw data/Fig.1/HE/SCI.jpg]

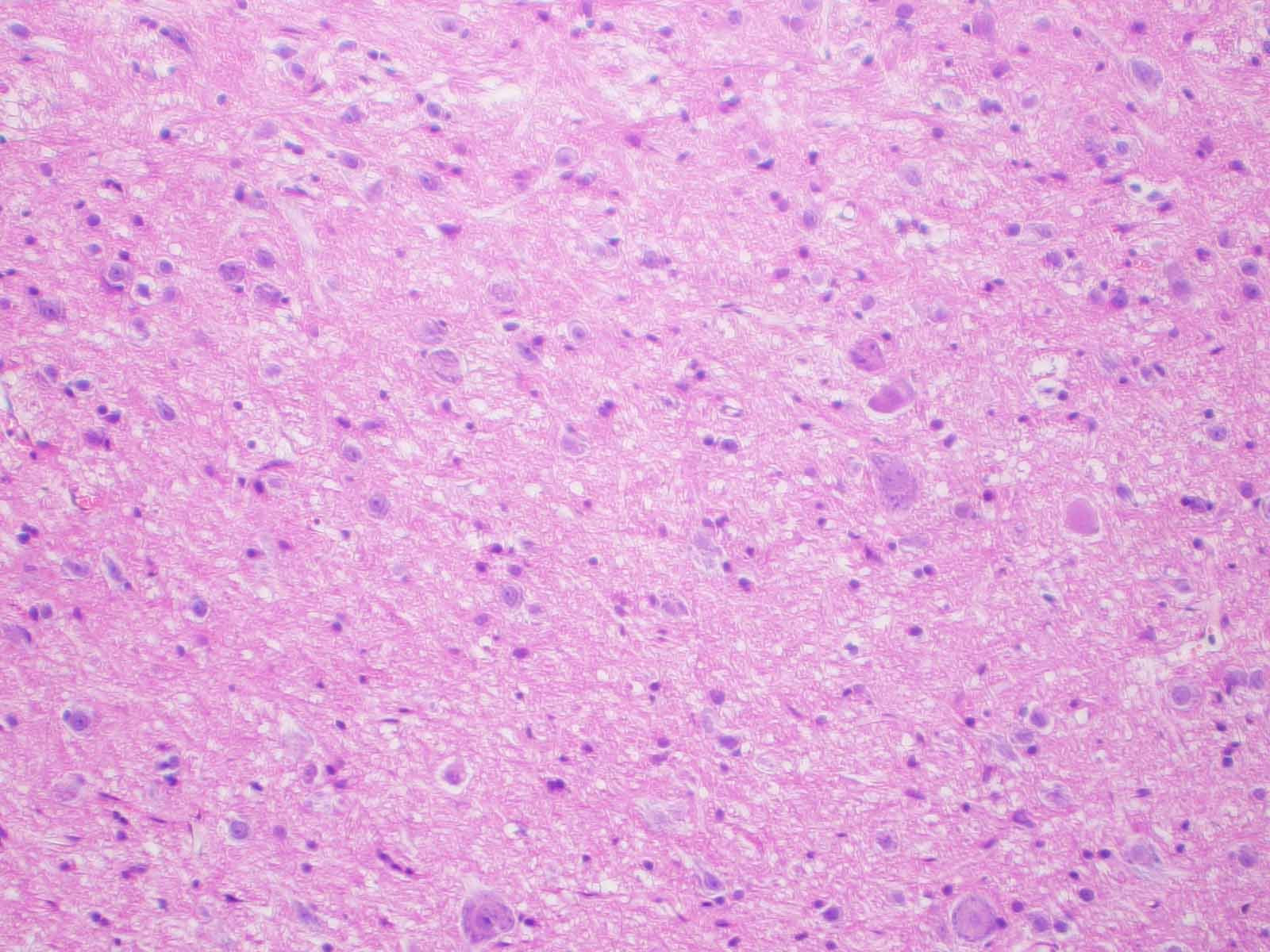

Supplement: Supplementary file 1 [file DataSheet1.ZIP › raw data/Fig.1/HE/Sham.jpg]

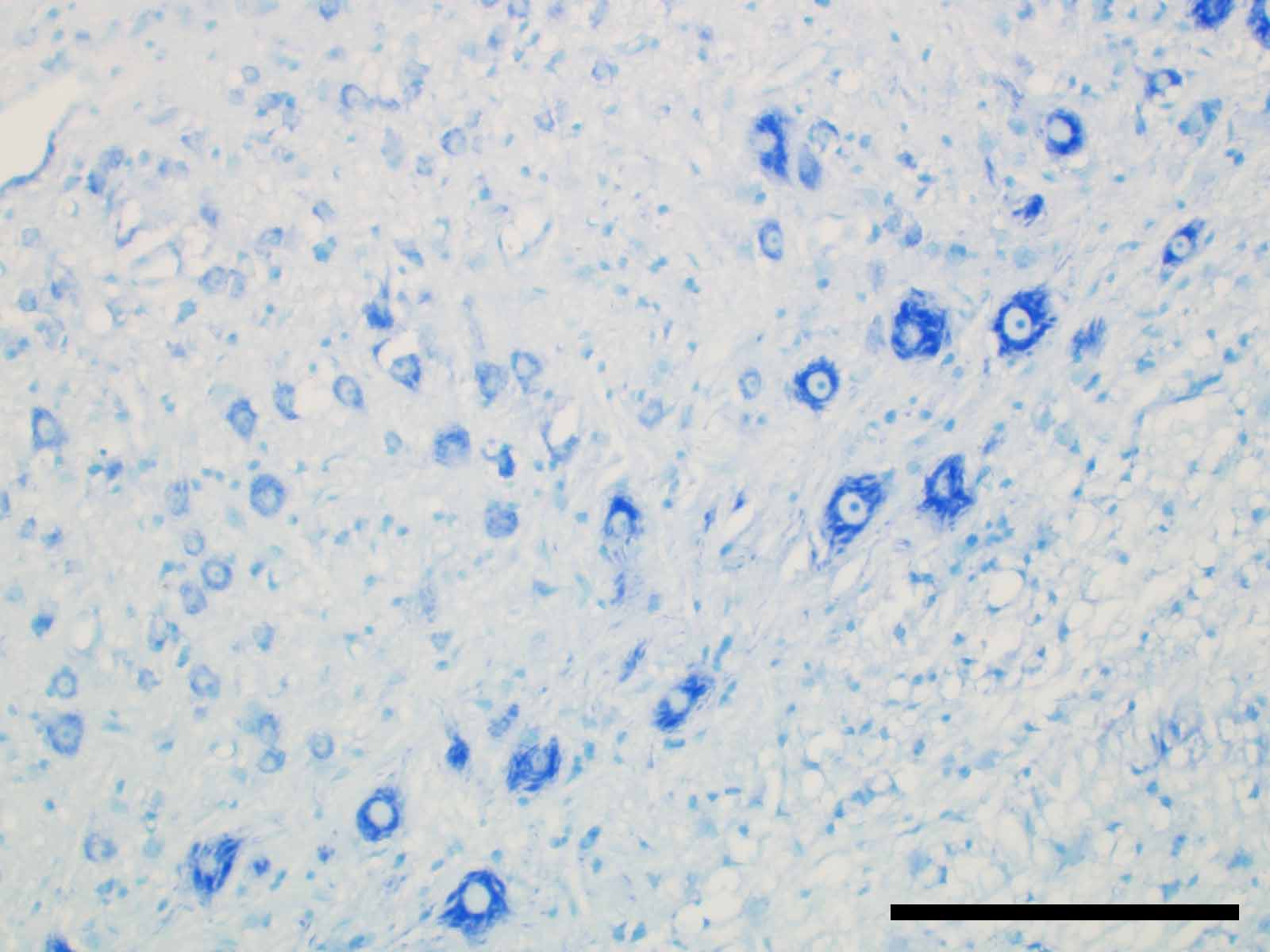

Supplement: Supplementary file 1 [file DataSheet1.ZIP › raw data/Fig.1/Nissl/SCI+Andro.jpg]

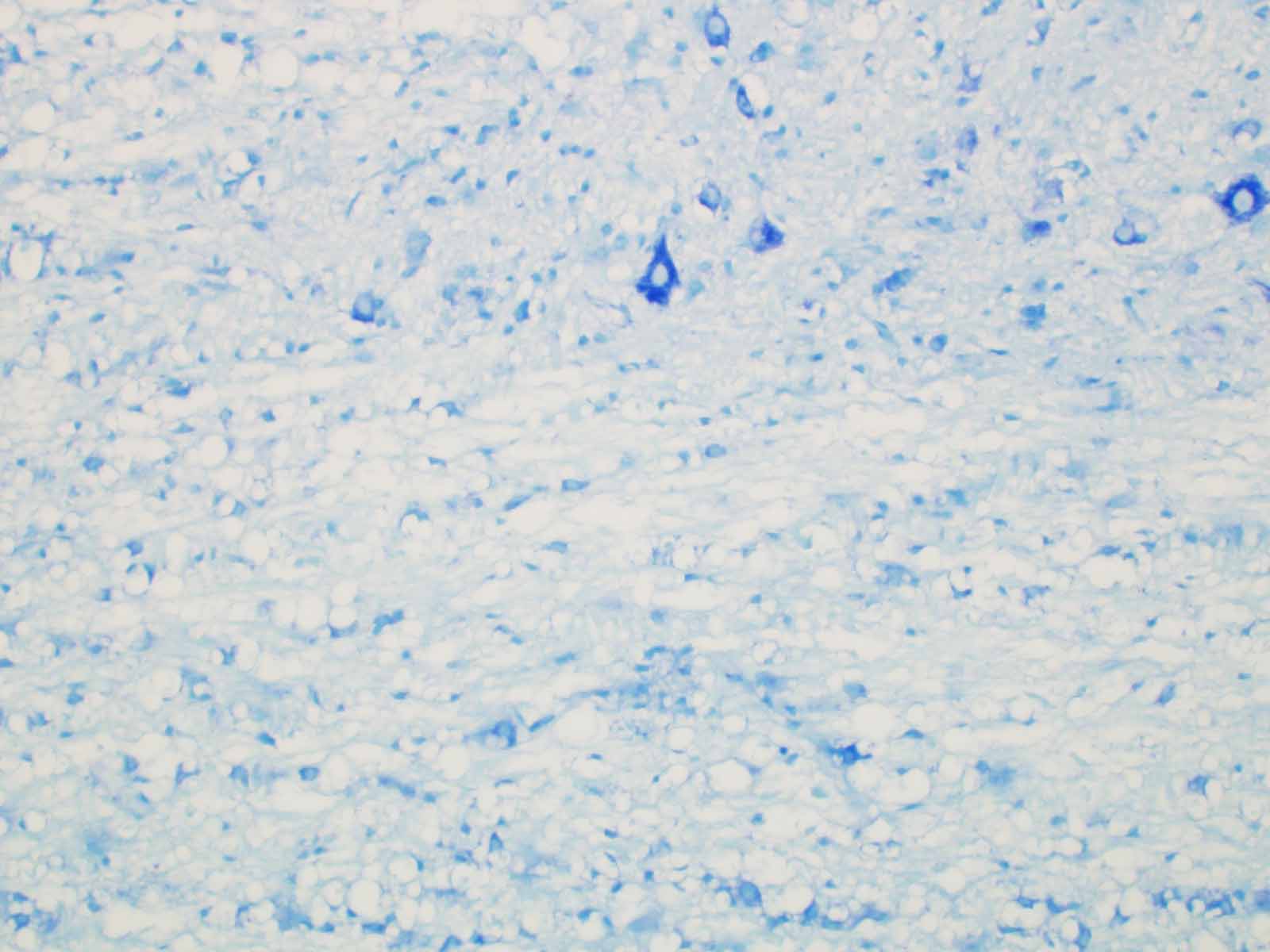

Supplement: Supplementary file 1 [file DataSheet1.ZIP › raw data/Fig.1/Nissl/SCI+NS.jpg]

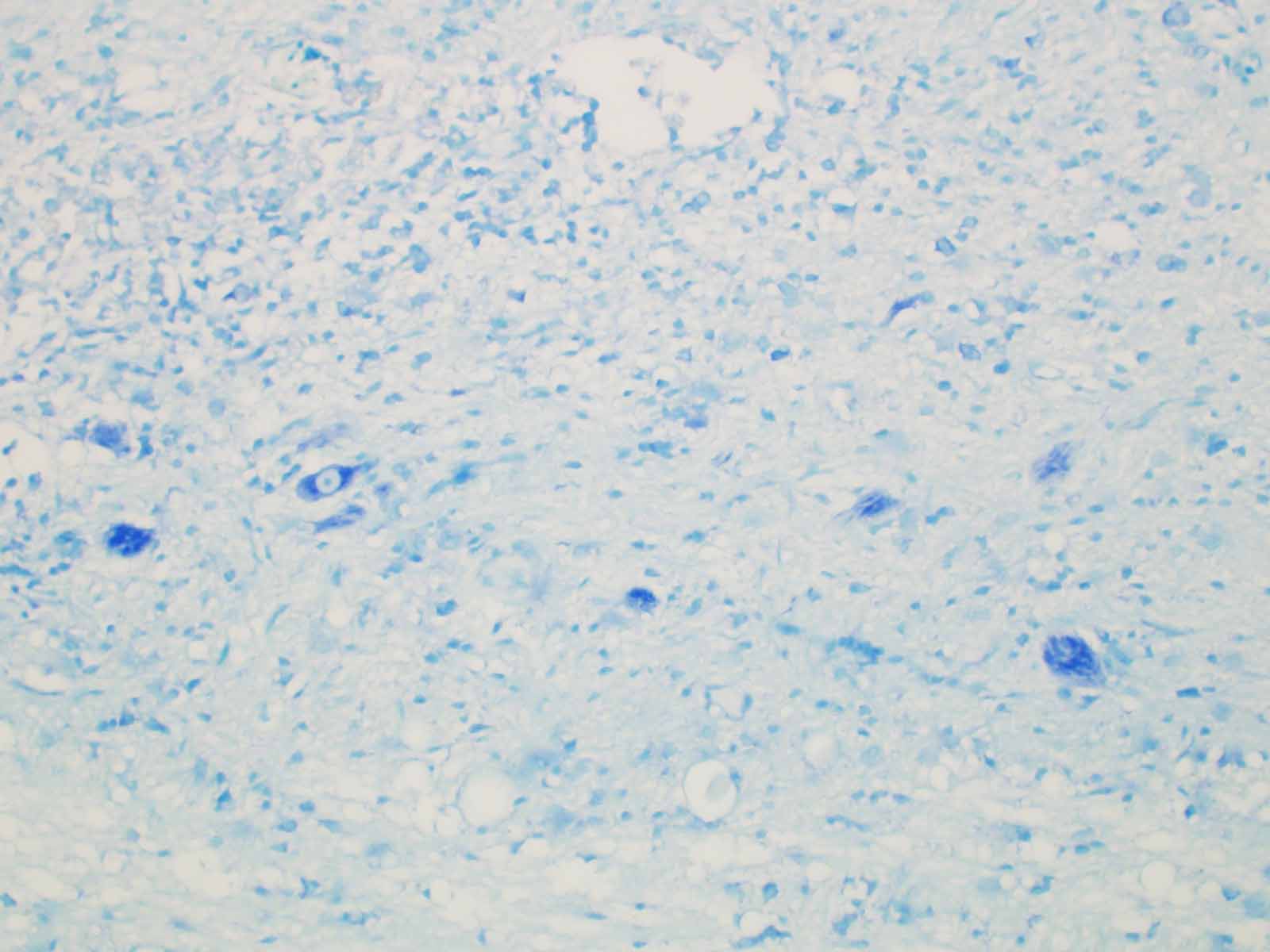

Supplement: Supplementary file 1 [file DataSheet1.ZIP › raw data/Fig.1/Nissl/SCI.jpg]

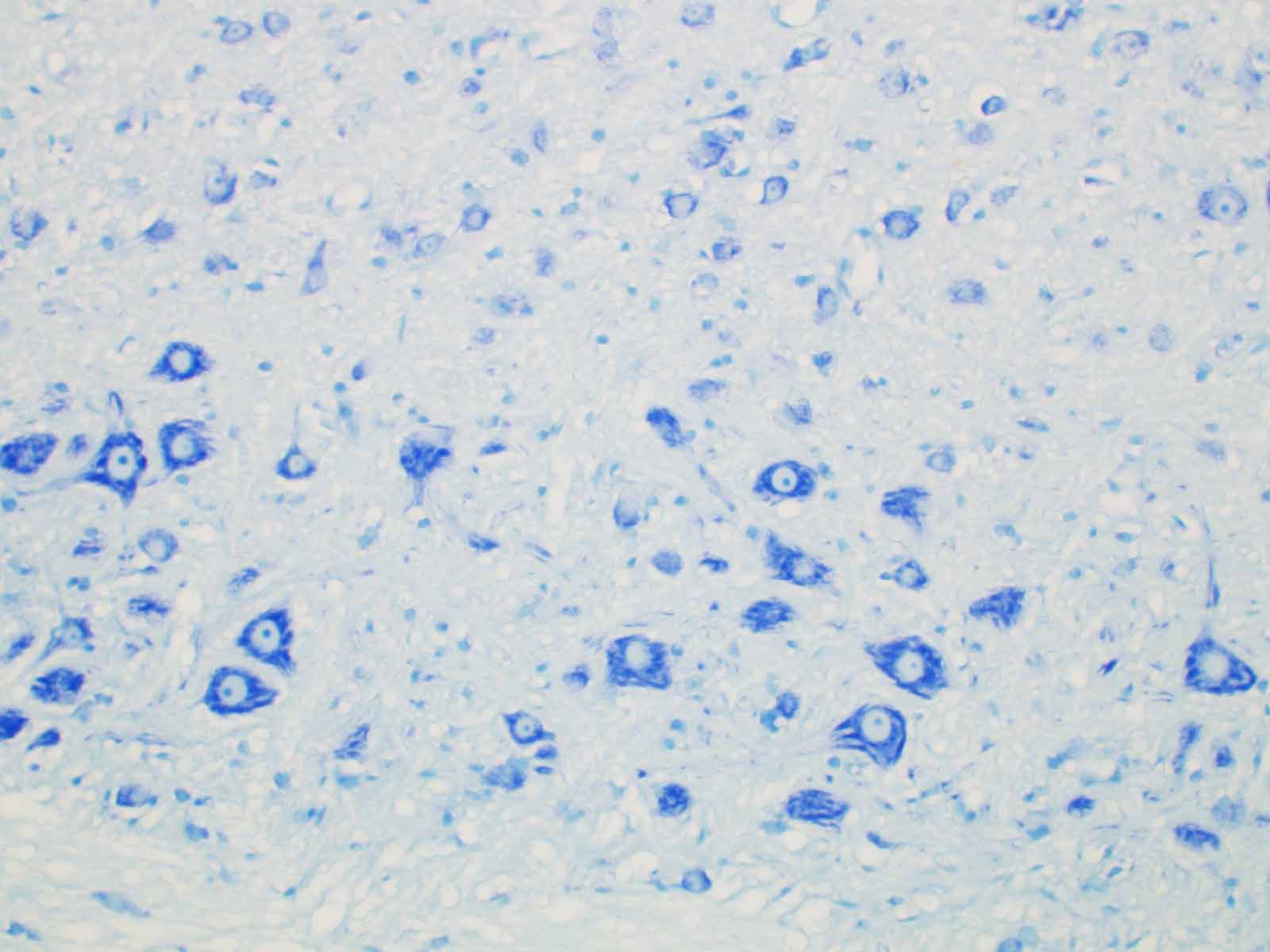

Supplement: Supplementary file 1 [file DataSheet1.ZIP › raw data/Fig.1/Nissl/Sham.jpg]

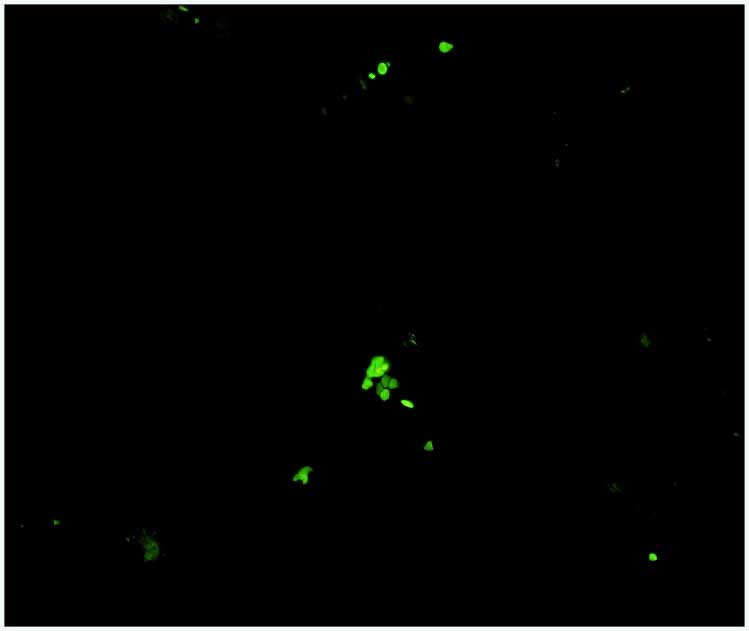

Supplement: Supplementary file 1 [file DataSheet1.ZIP › raw data/Fig.2/Tunel/SCI+Andro/1.jpg]

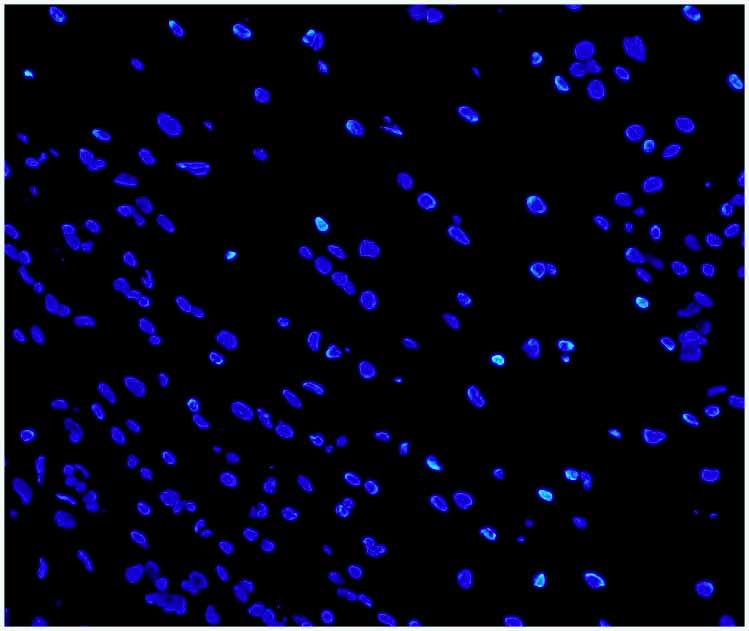

Supplement: Supplementary file 1 [file DataSheet1.ZIP › raw data/Fig.2/Tunel/SCI+Andro/2.jpg]

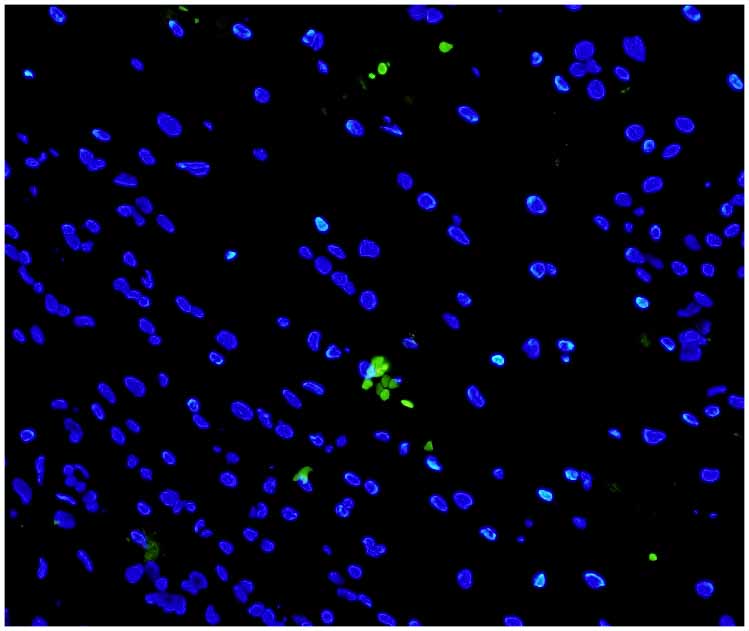

Supplement: Supplementary file 1 [file DataSheet1.ZIP › raw data/Fig.2/Tunel/SCI+Andro/3.jpg]

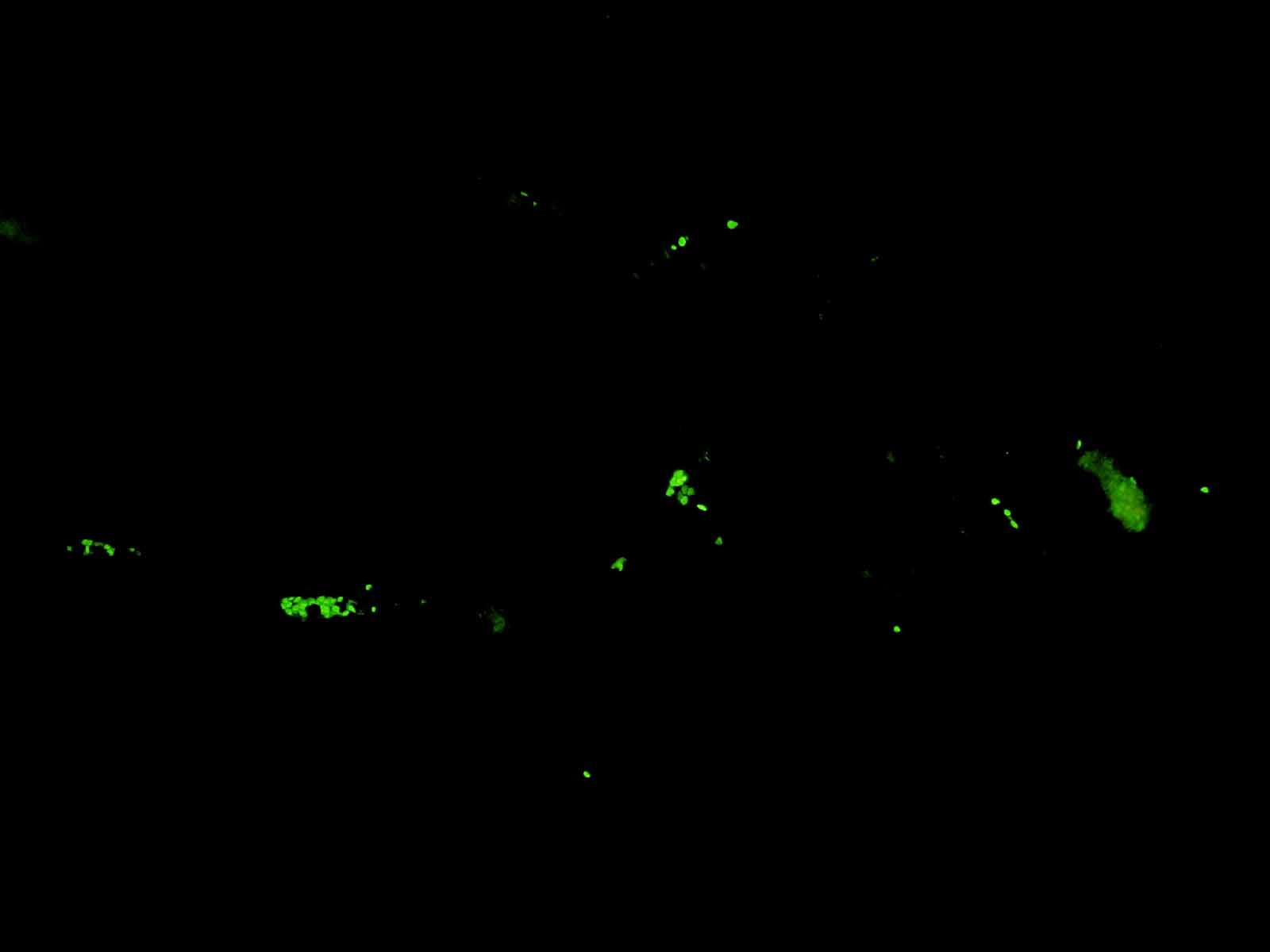

Supplement: Supplementary file 1 [file DataSheet1.ZIP › raw data/Fig.2/Tunel/SCI+Andro/═╝╧±_51963.jpg]

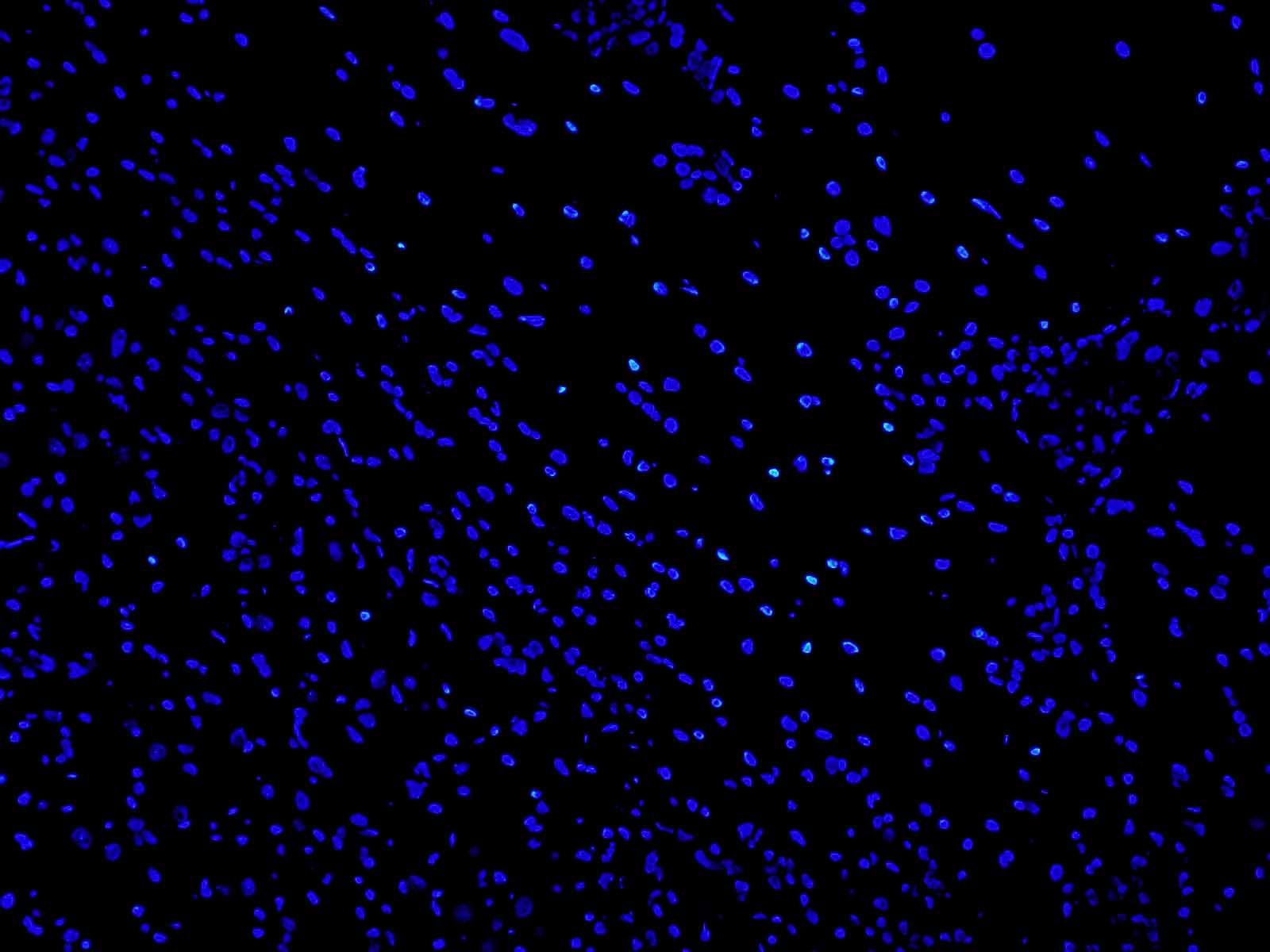

Supplement: Supplementary file 1 [file DataSheet1.ZIP › raw data/Fig.2/Tunel/SCI+Andro/═╝╧±_51973.jpg]

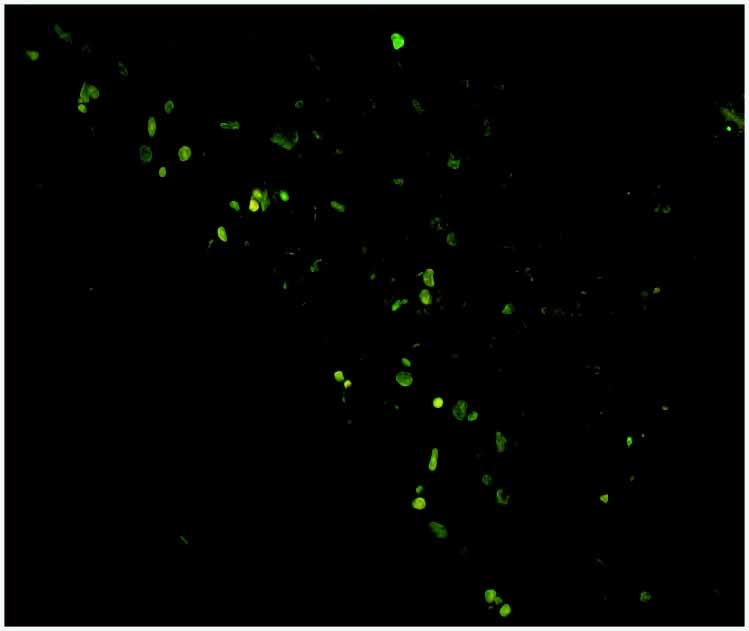

Supplement: Supplementary file 1 [file DataSheet1.ZIP › raw data/Fig.2/Tunel/SCI+NS/1.jpg]

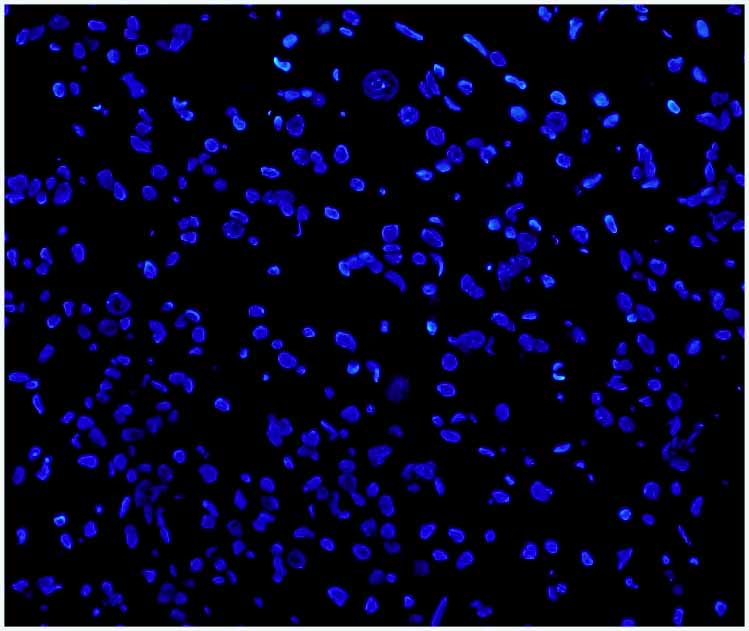

Supplement: Supplementary file 1 [file DataSheet1.ZIP › raw data/Fig.2/Tunel/SCI+NS/2.jpg]

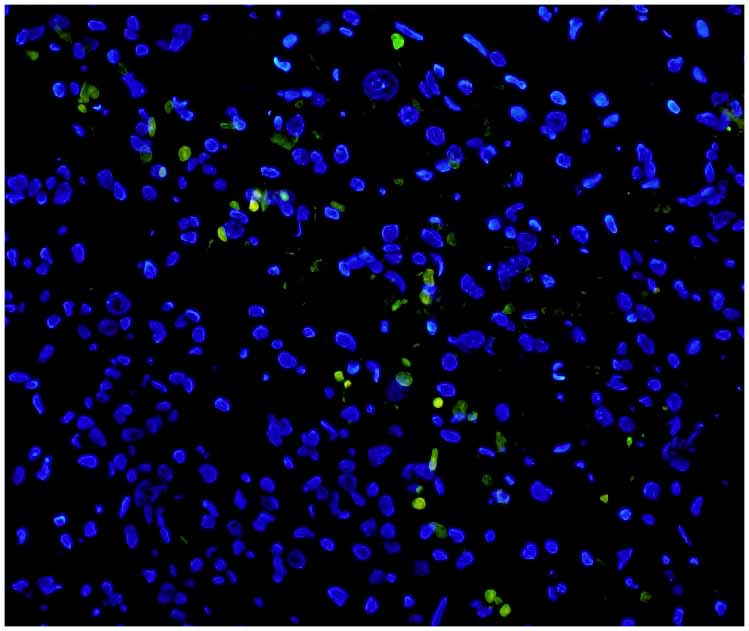

Supplement: Supplementary file 1 [file DataSheet1.ZIP › raw data/Fig.2/Tunel/SCI+NS/3.jpg]

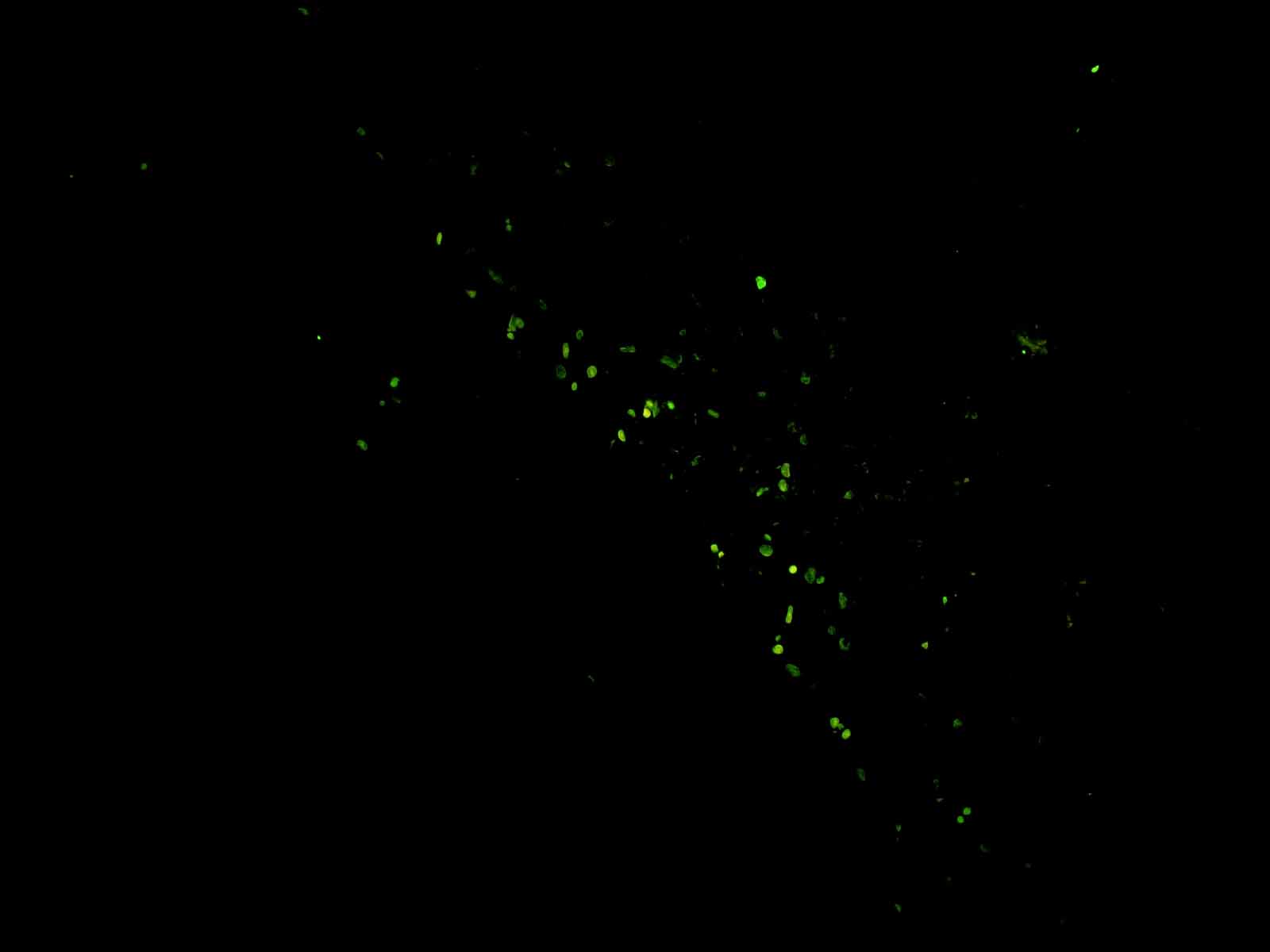

Supplement: Supplementary file 1 [file DataSheet1.ZIP › raw data/Fig.2/Tunel/SCI+NS/═╝╧±_51960.jpg]

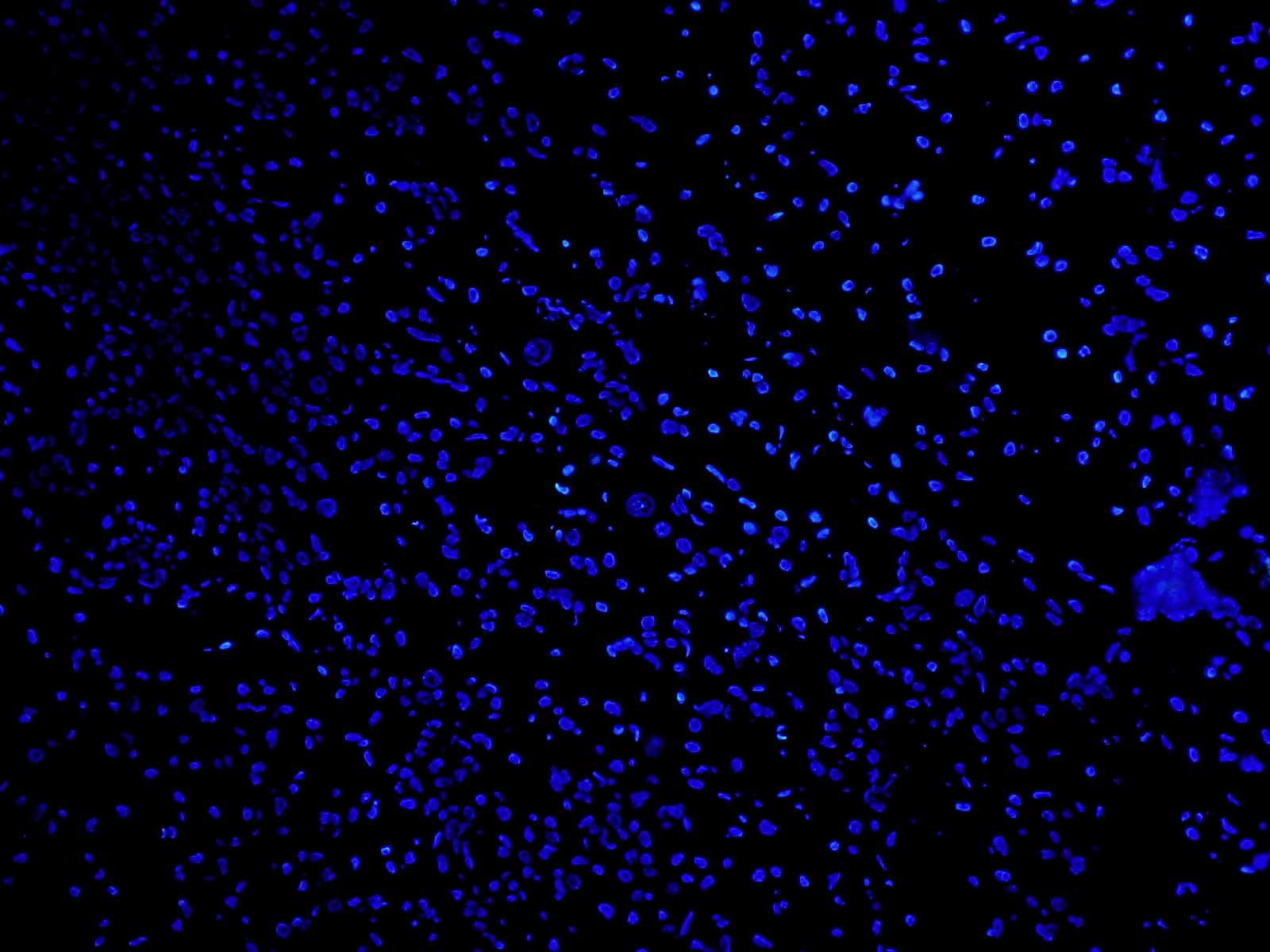

Supplement: Supplementary file 1 [file DataSheet1.ZIP › raw data/Fig.2/Tunel/SCI+NS/═╝╧±_51979.jpg]

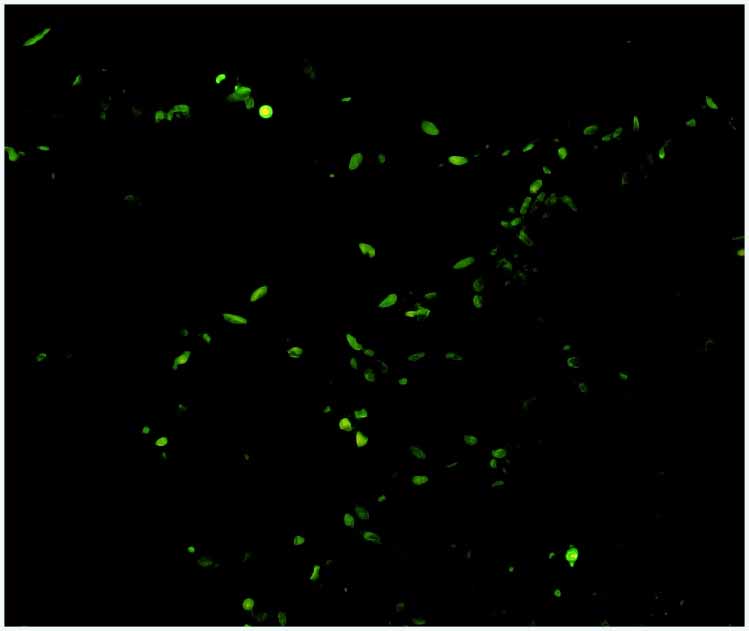

Supplement: Supplementary file 1 [file DataSheet1.ZIP › raw data/Fig.2/Tunel/SCI/1.jpg]

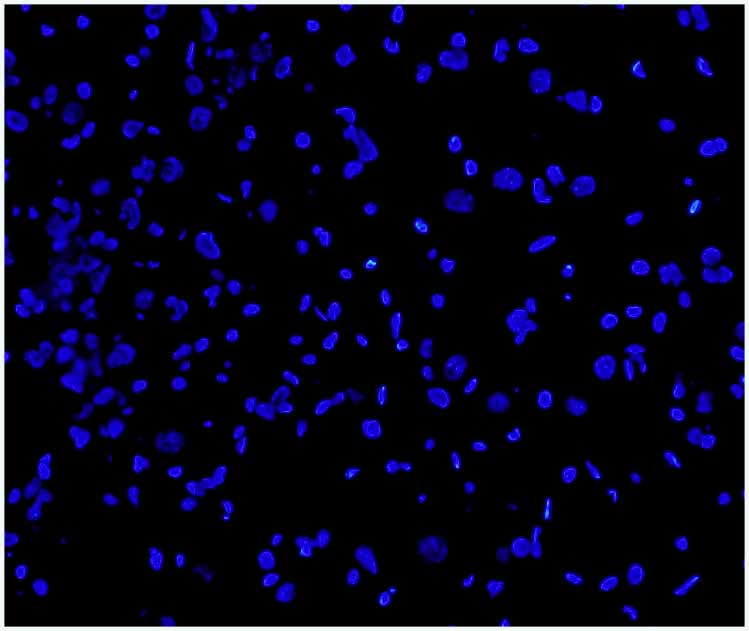

Supplement: Supplementary file 1 [file DataSheet1.ZIP › raw data/Fig.2/Tunel/SCI/2.jpg]

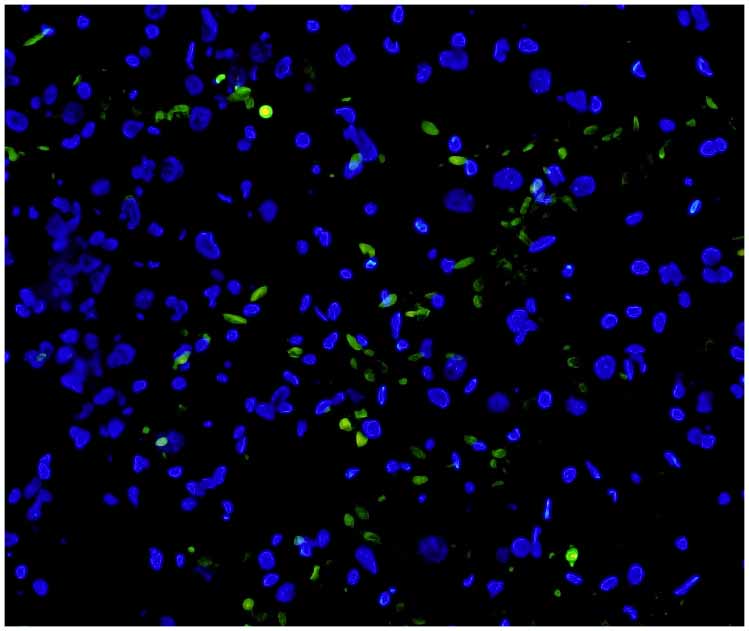

Supplement: Supplementary file 1 [file DataSheet1.ZIP › raw data/Fig.2/Tunel/SCI/3.jpg]

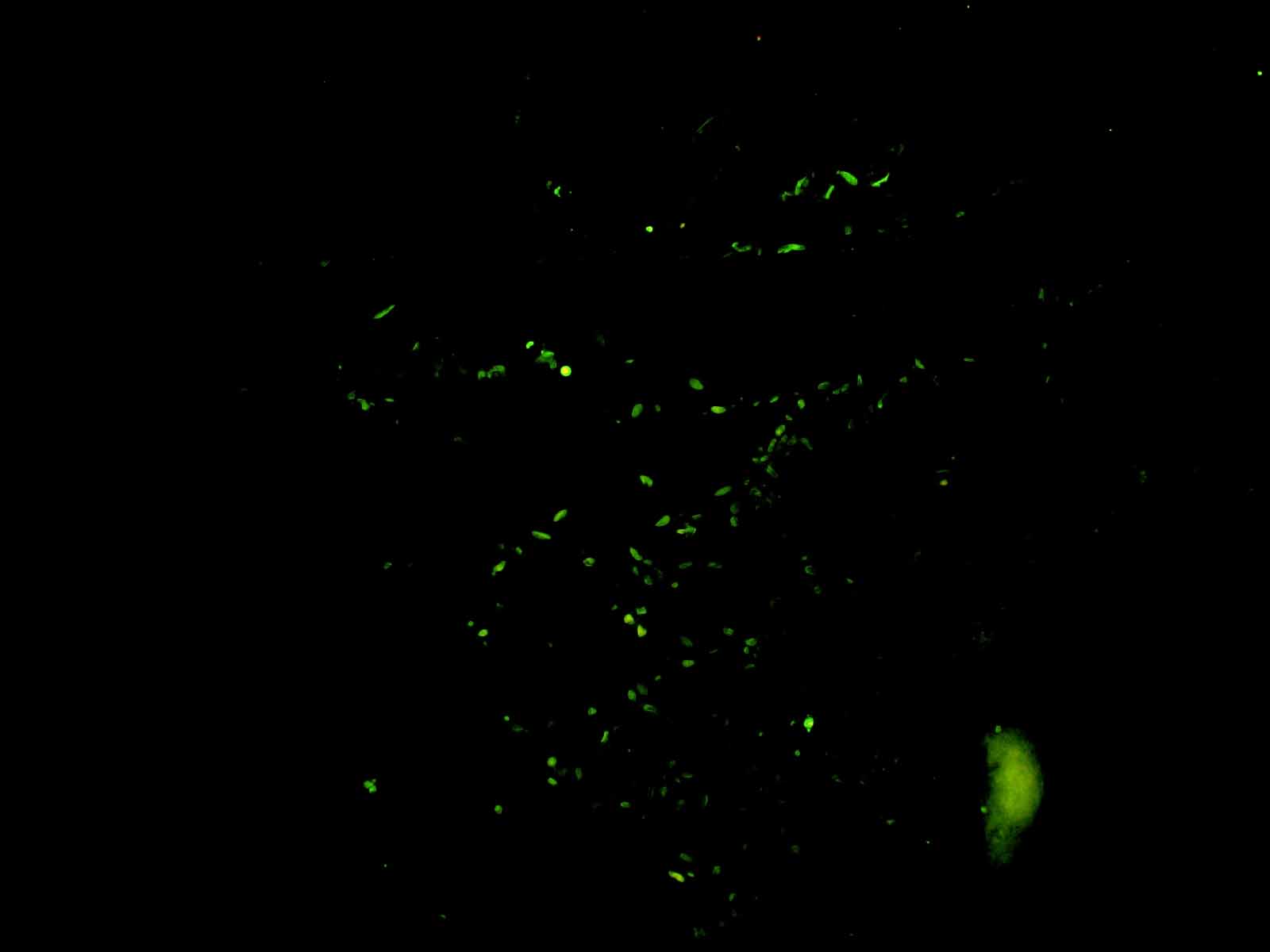

Supplement: Supplementary file 1 [file DataSheet1.ZIP › raw data/Fig.2/Tunel/SCI/═╝╧±_51959.jpg]

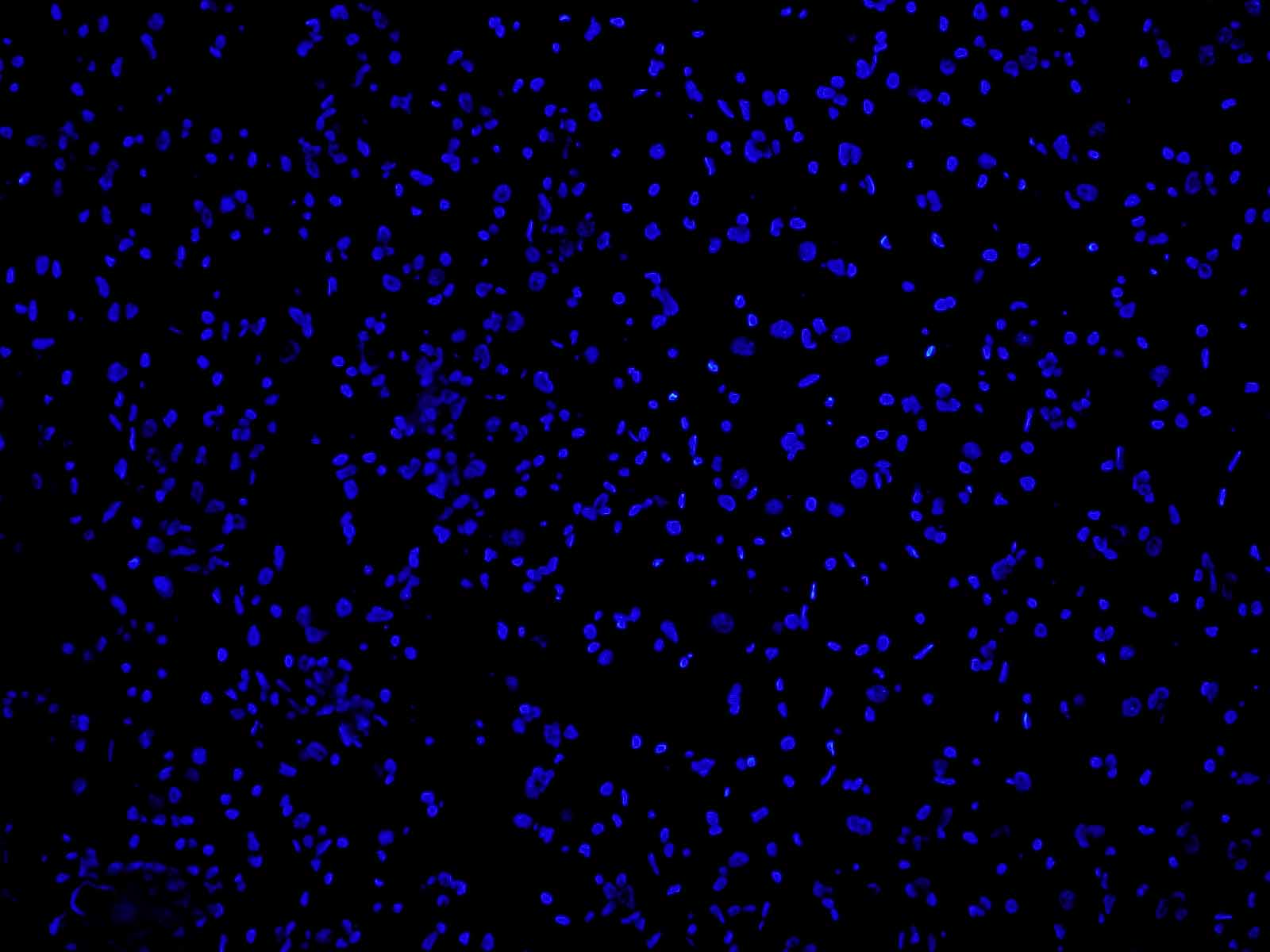

Supplement: Supplementary file 1 [file DataSheet1.ZIP › raw data/Fig.2/Tunel/SCI/═╝╧±_51999.jpg]

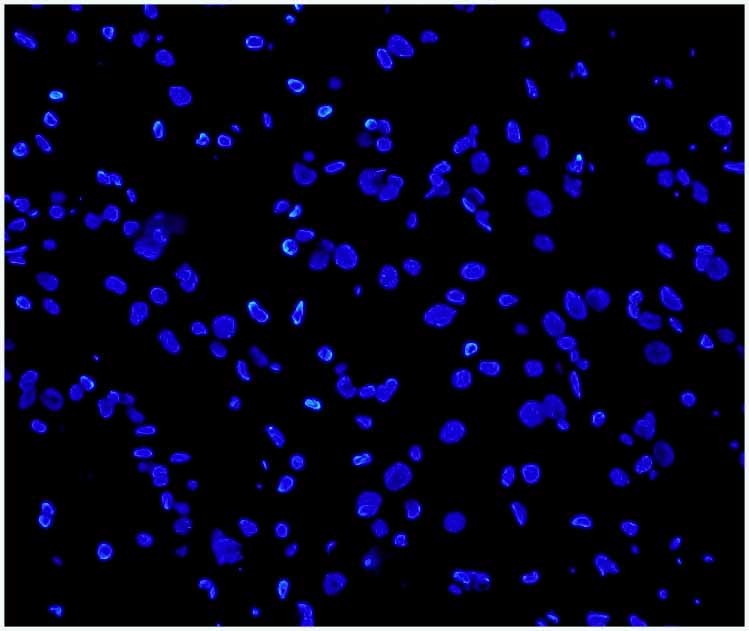

Supplement: Supplementary file 1 [file DataSheet1.ZIP › raw data/Fig.2/Tunel/Sham/1.jpg]

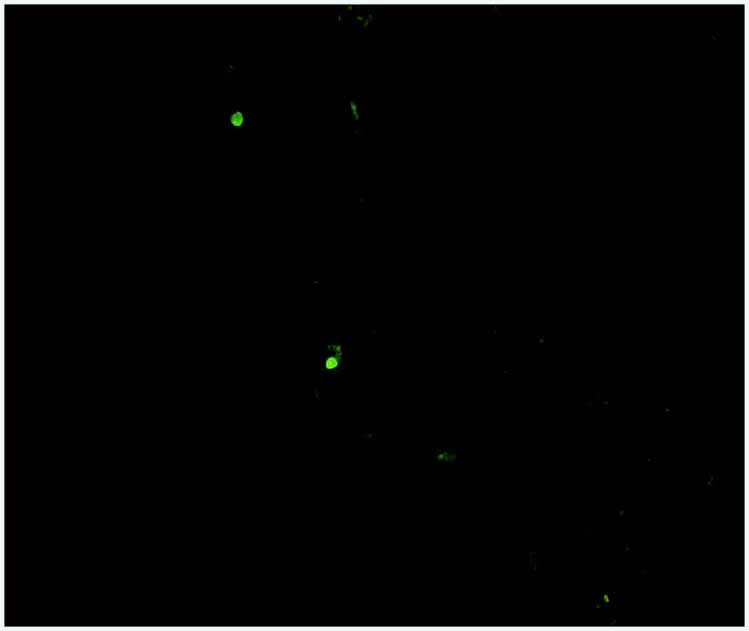

Supplement: Supplementary file 1 [file DataSheet1.ZIP › raw data/Fig.2/Tunel/Sham/2.jpg]

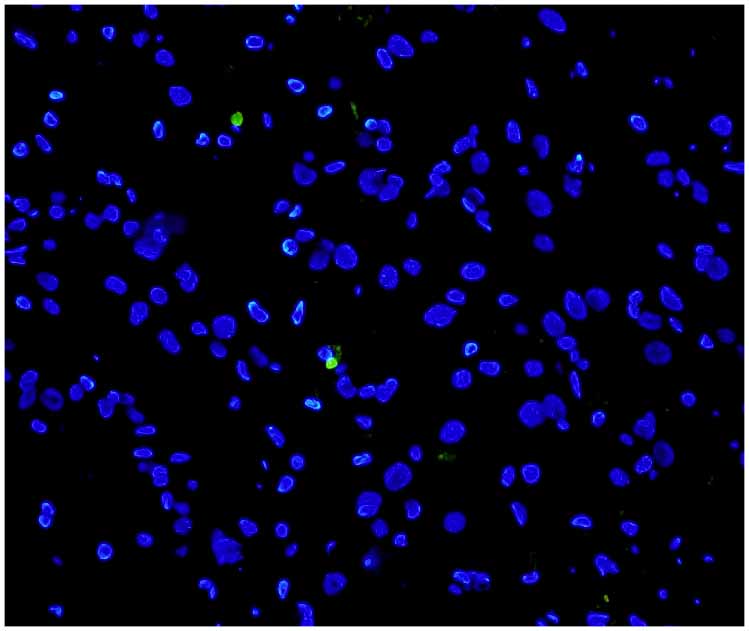

Supplement: Supplementary file 1 [file DataSheet1.ZIP › raw data/Fig.2/Tunel/Sham/3.jpg]

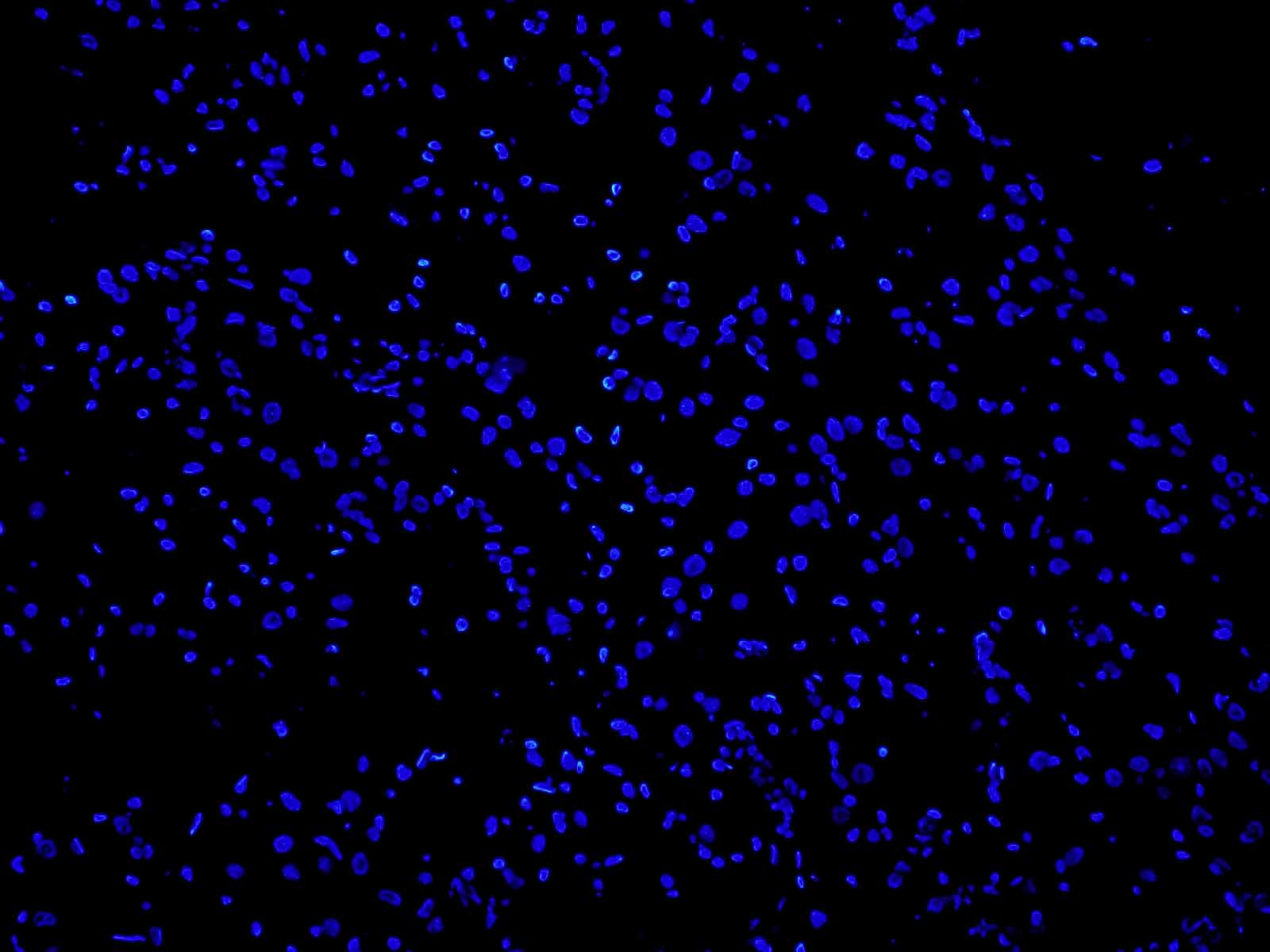

Supplement: Supplementary file 1 [file DataSheet1.ZIP › raw data/Fig.2/Tunel/Sham/═╝╧±_51955.jpg]

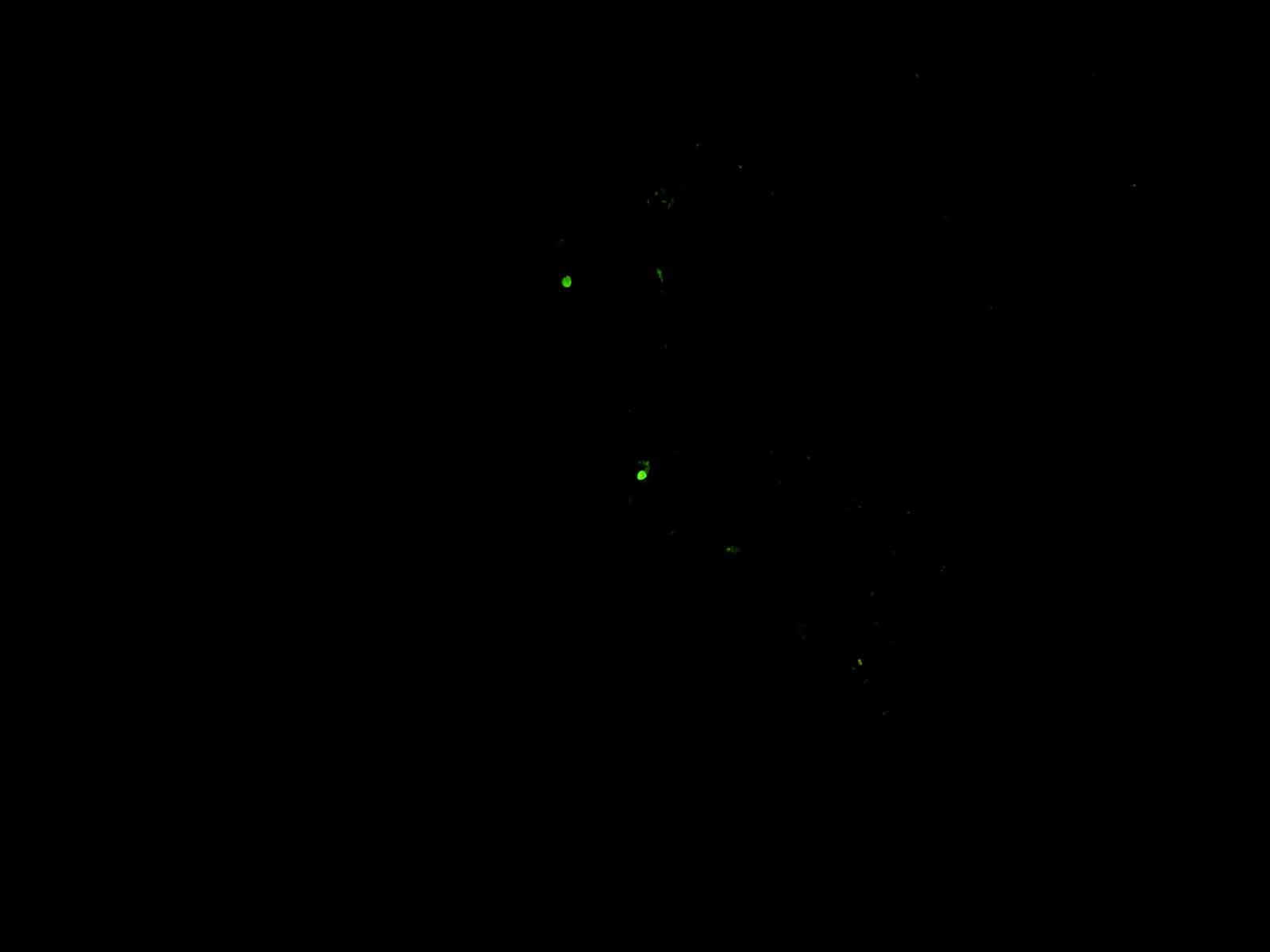

Supplement: Supplementary file 1 [file DataSheet1.ZIP › raw data/Fig.2/Tunel/Sham/═╝╧±_51956.jpg]

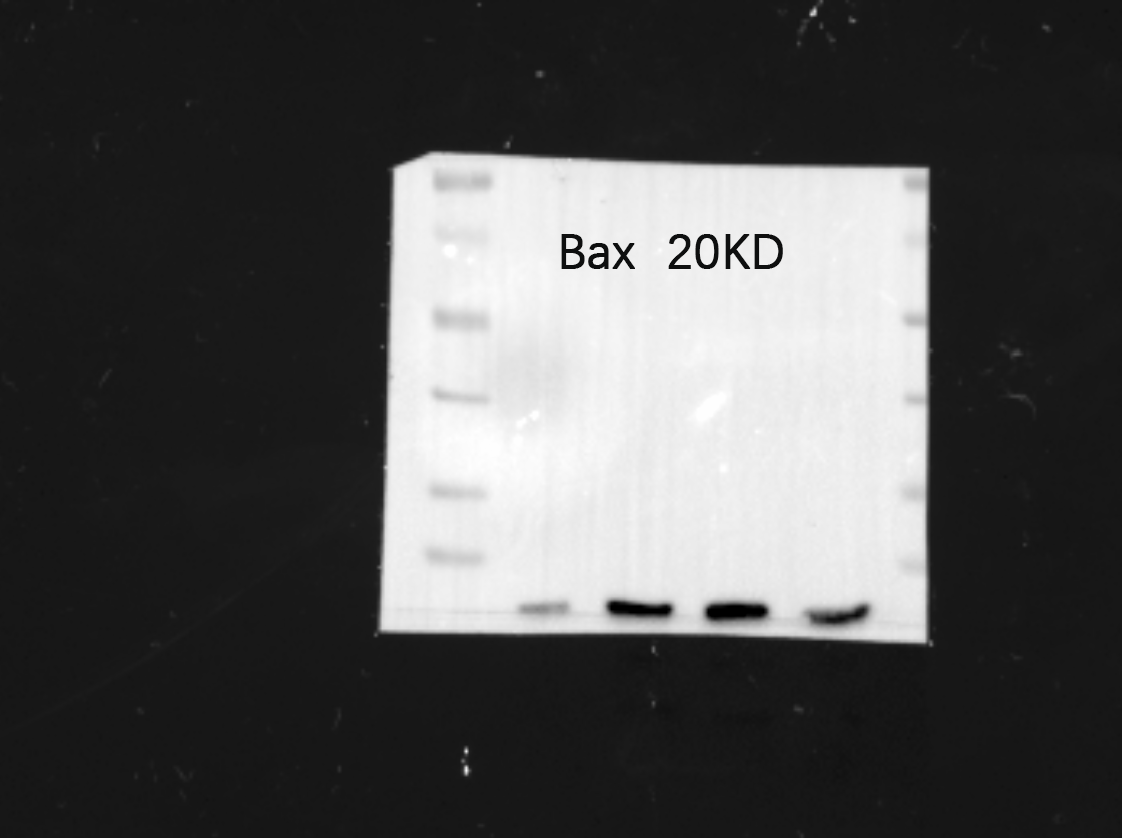

Supplement: Supplementary file 1 [file DataSheet1.ZIP › raw data/Fig.2/Western blot images/Bax.tif]

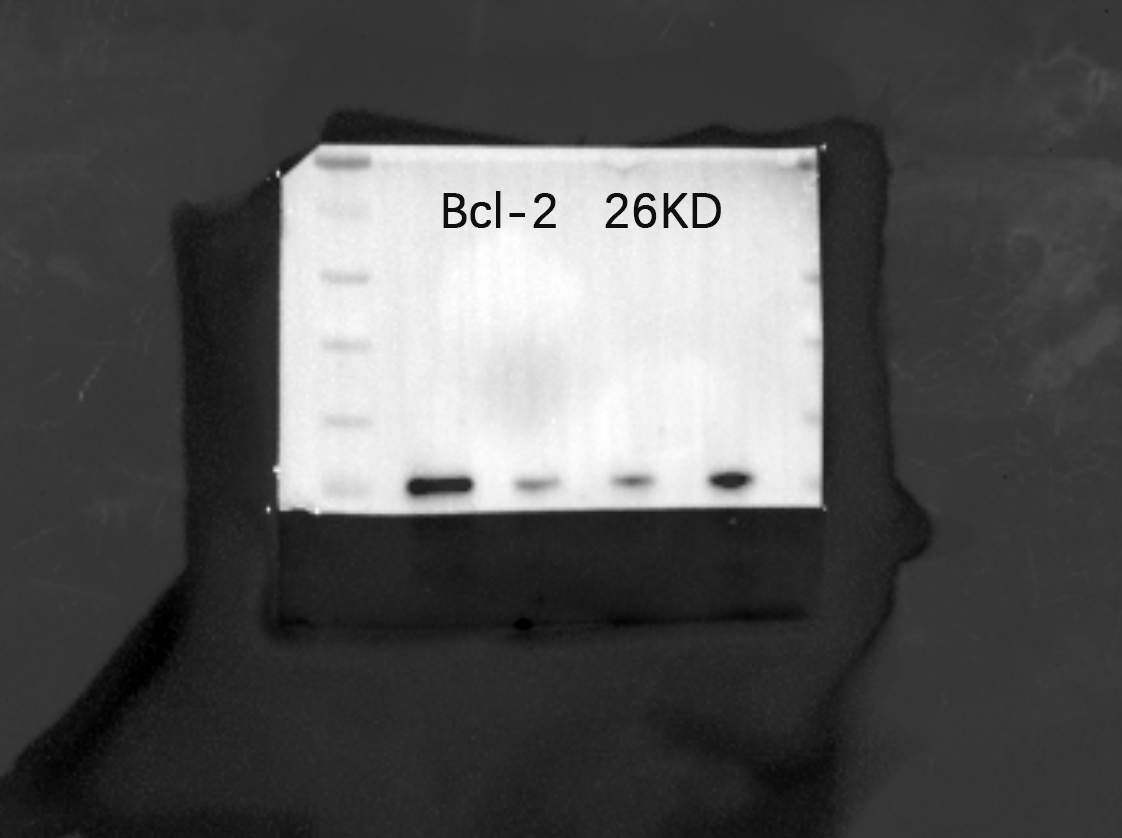

Supplement: Supplementary file 1 [file DataSheet1.ZIP › raw data/Fig.2/Western blot images/Bcl-2.tif]

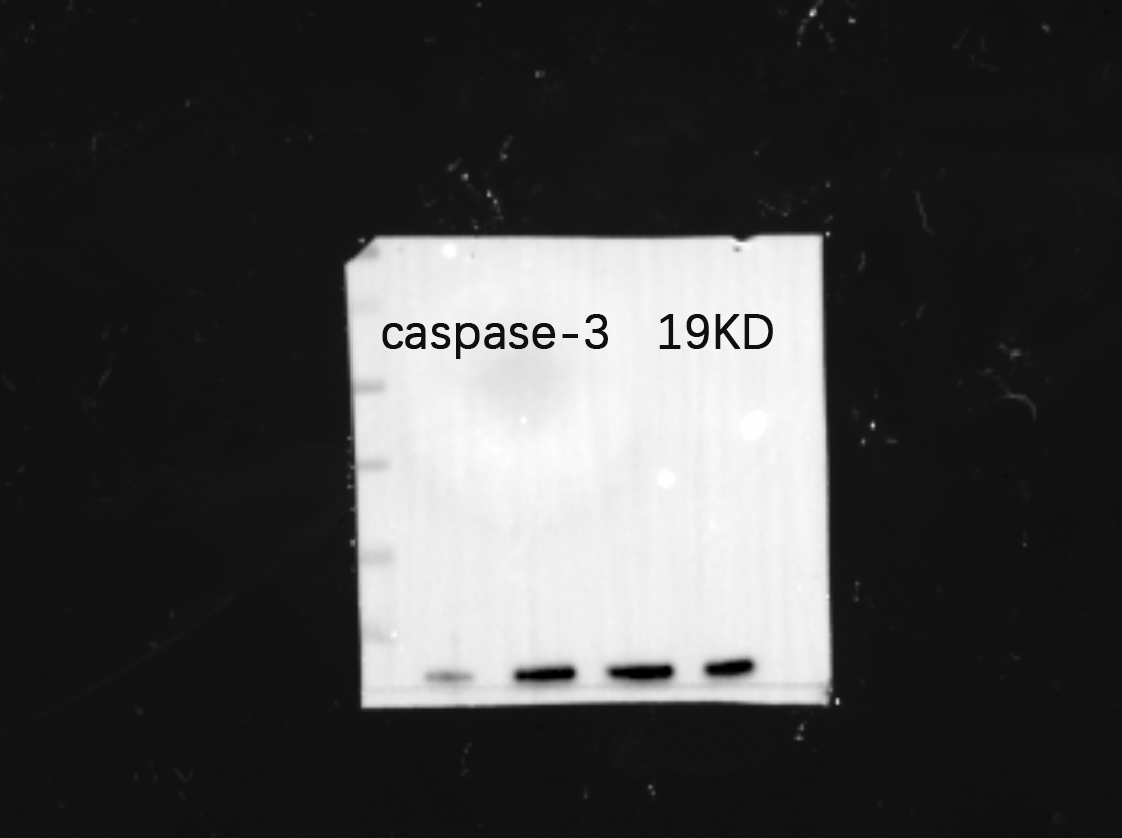

Supplement: Supplementary file 1 [file DataSheet1.ZIP › raw data/Fig.2/Western blot images/Cleaved caspase-3.tif]

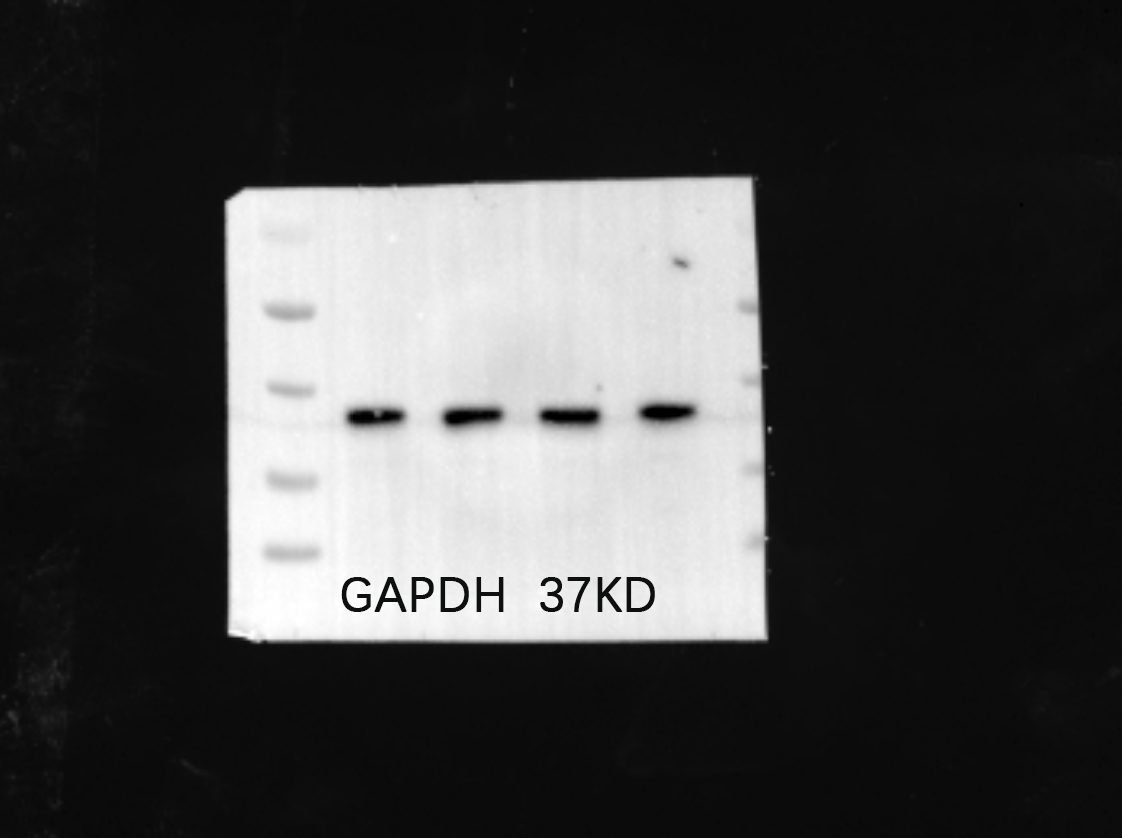

Supplement: Supplementary file 1 [file DataSheet1.ZIP › raw data/Fig.2/Western blot images/GAPDH.tif]

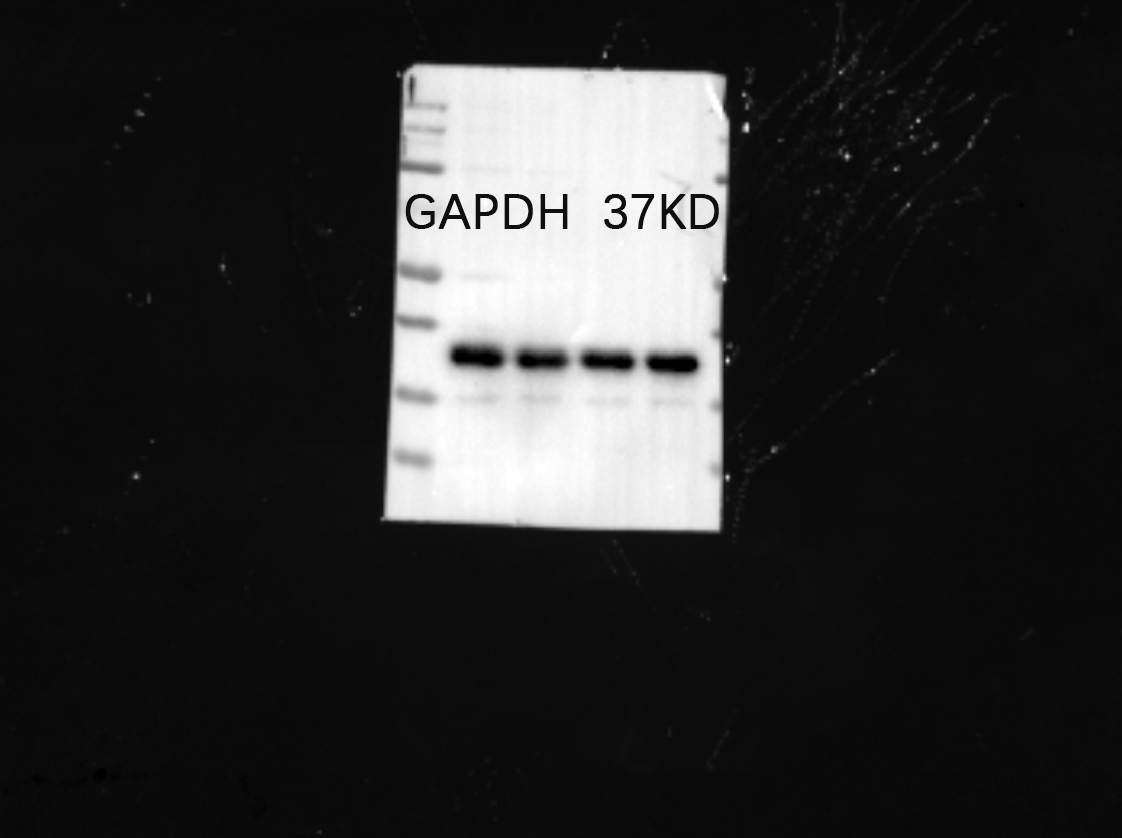

Supplement: Supplementary file 1 [file DataSheet1.ZIP › raw data/Fig.3/Western blot images/NF-a╩B/GAPDH.tif]

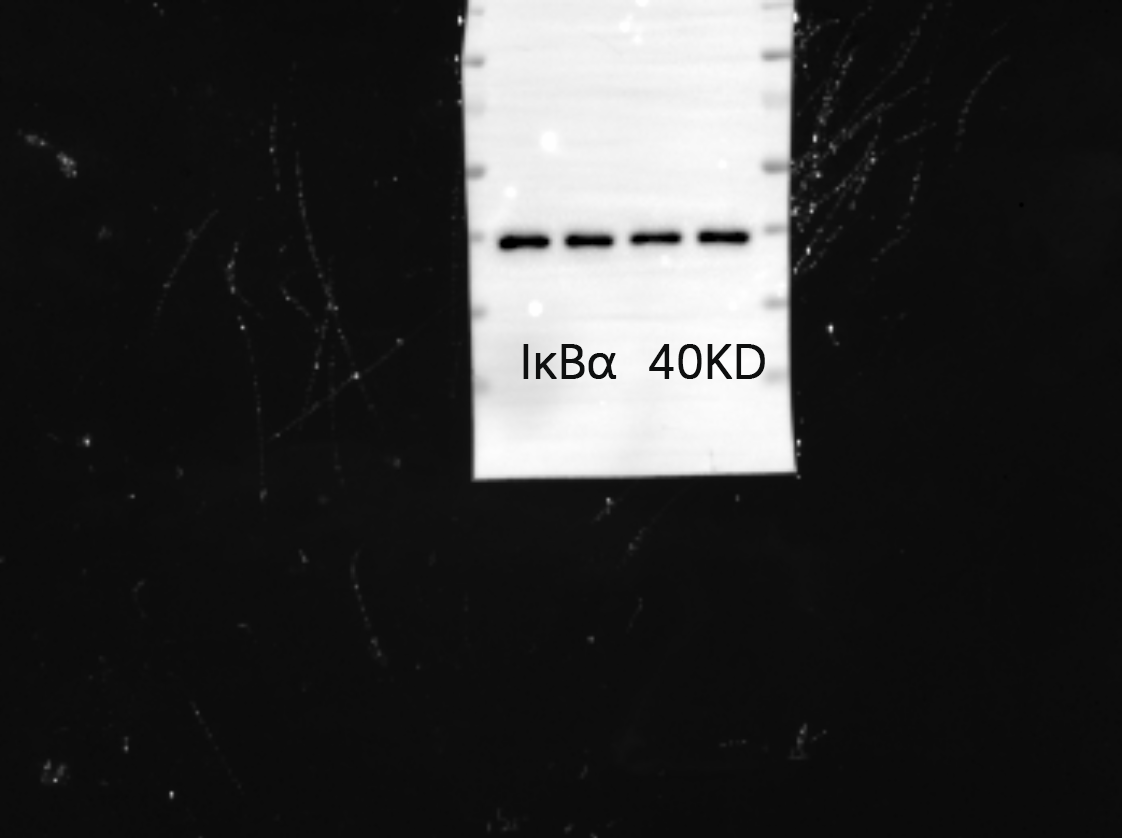

Supplement: Supplementary file 1 [file DataSheet1.ZIP › raw data/Fig.3/Western blot images/NF-a╩B/Ia╩Ba┴.tif]

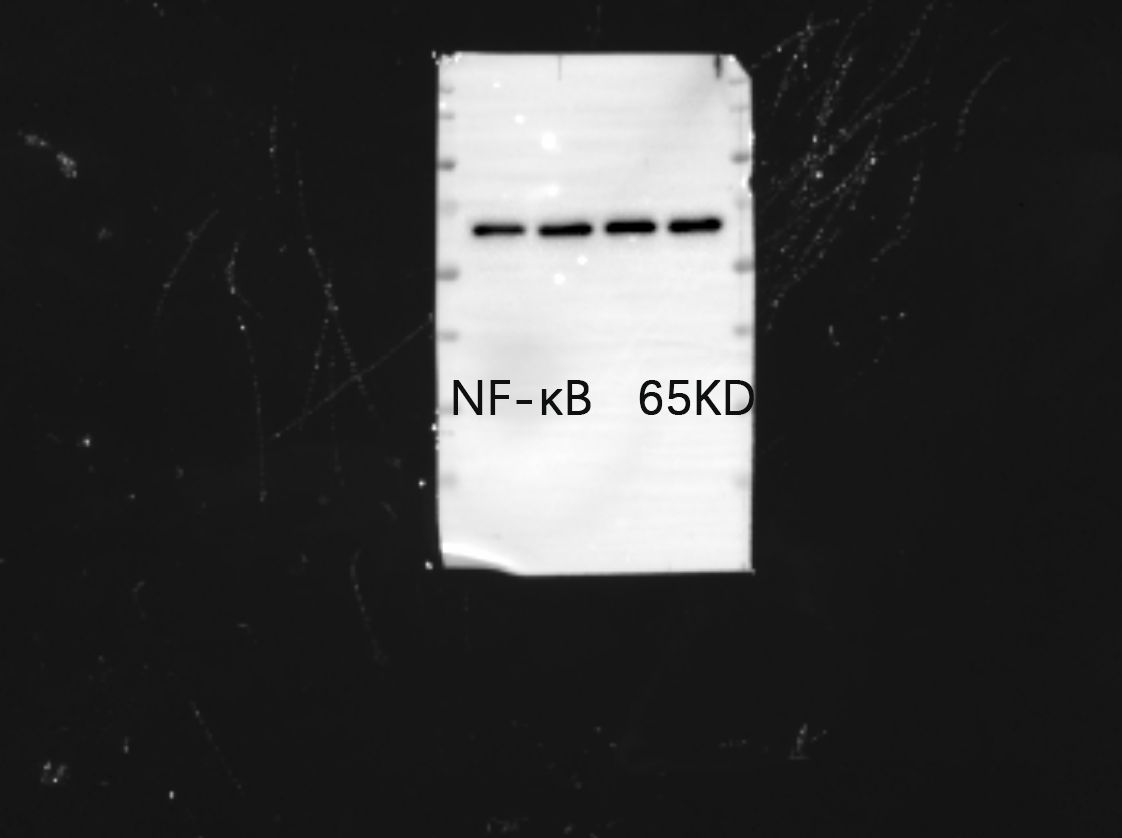

Supplement: Supplementary file 1 [file DataSheet1.ZIP › raw data/Fig.3/Western blot images/NF-a╩B/NF-a╩B.tif]

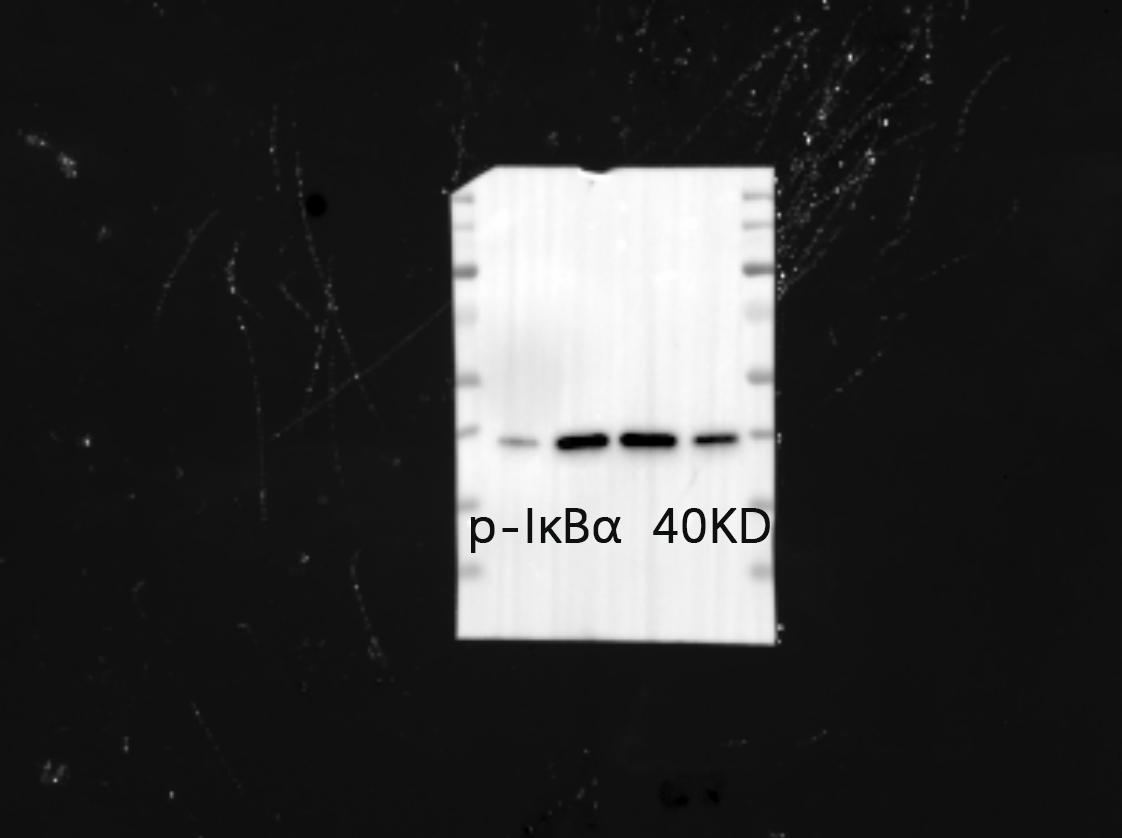

Supplement: Supplementary file 1 [file DataSheet1.ZIP › raw data/Fig.3/Western blot images/NF-a╩B/p-Ia╩Ba┴.tif]

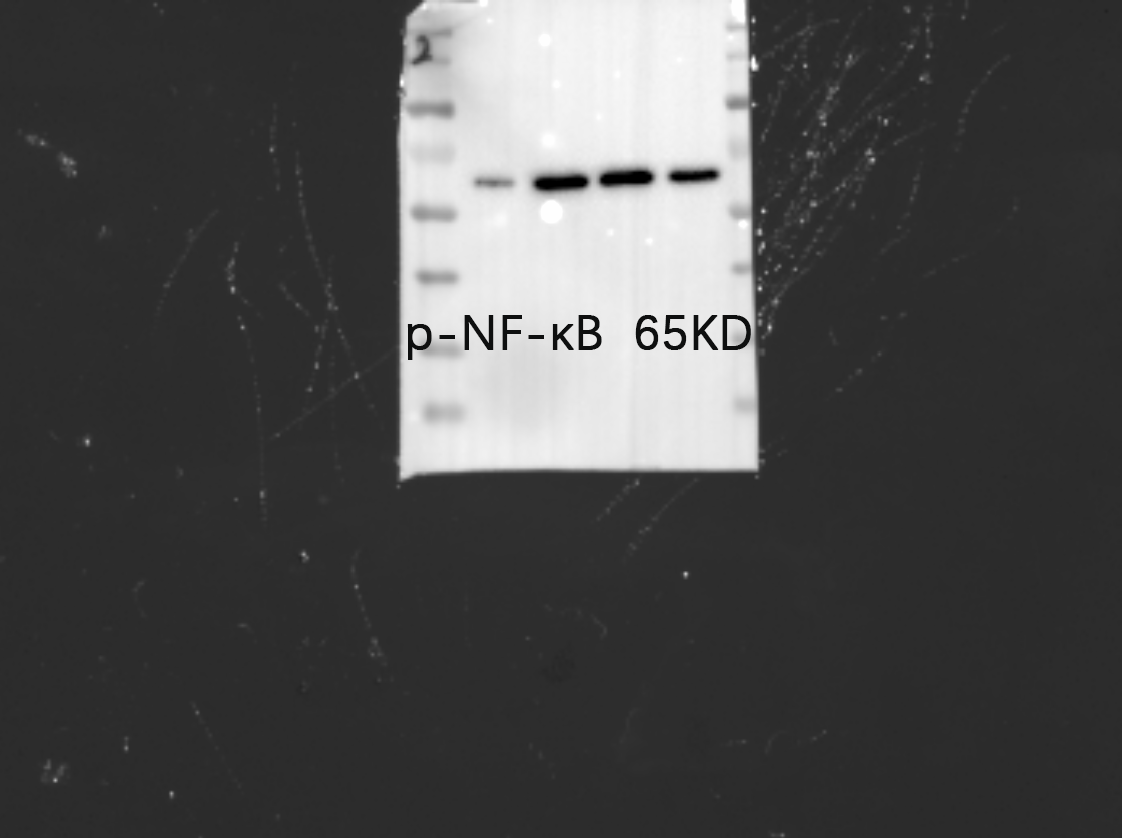

Supplement: Supplementary file 1 [file DataSheet1.ZIP › raw data/Fig.3/Western blot images/NF-a╩B/p-NF-a╩B.tif]

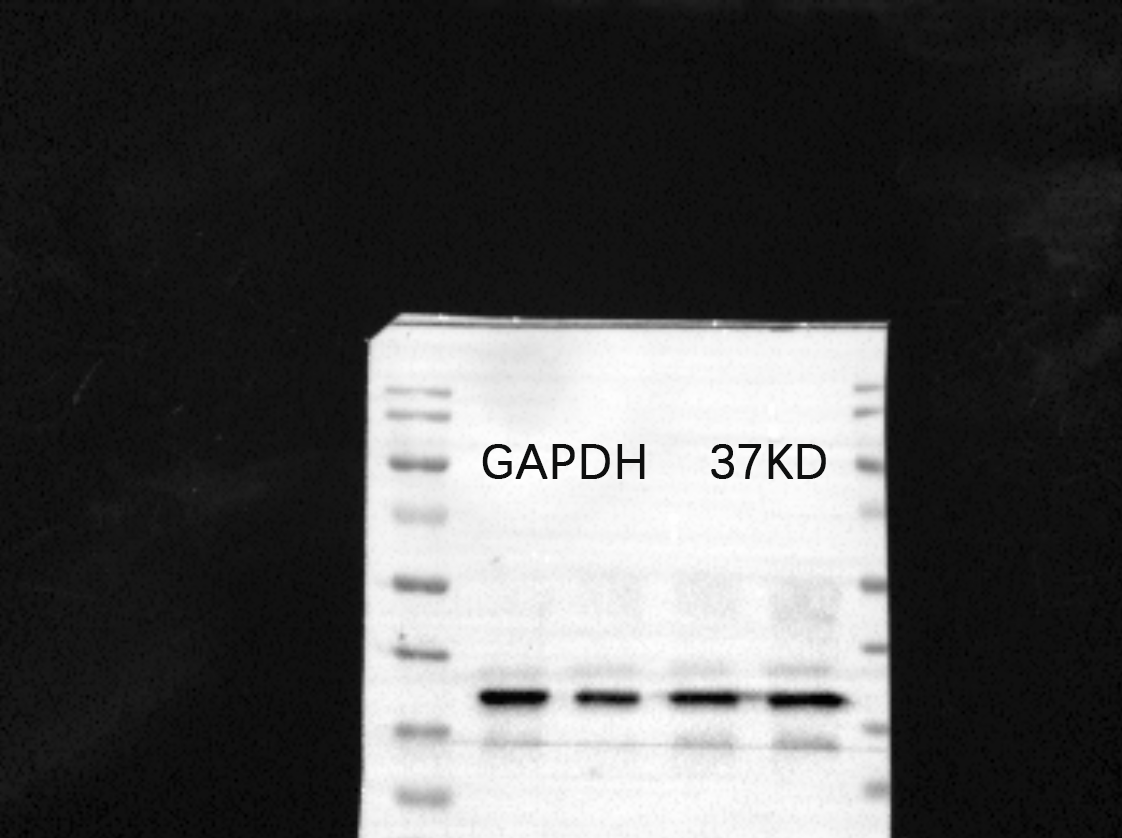

Supplement: Supplementary file 1 [file DataSheet1.ZIP › raw data/Fig.3/Western blot images/Nrf2-HO1/GAPDH.tif]

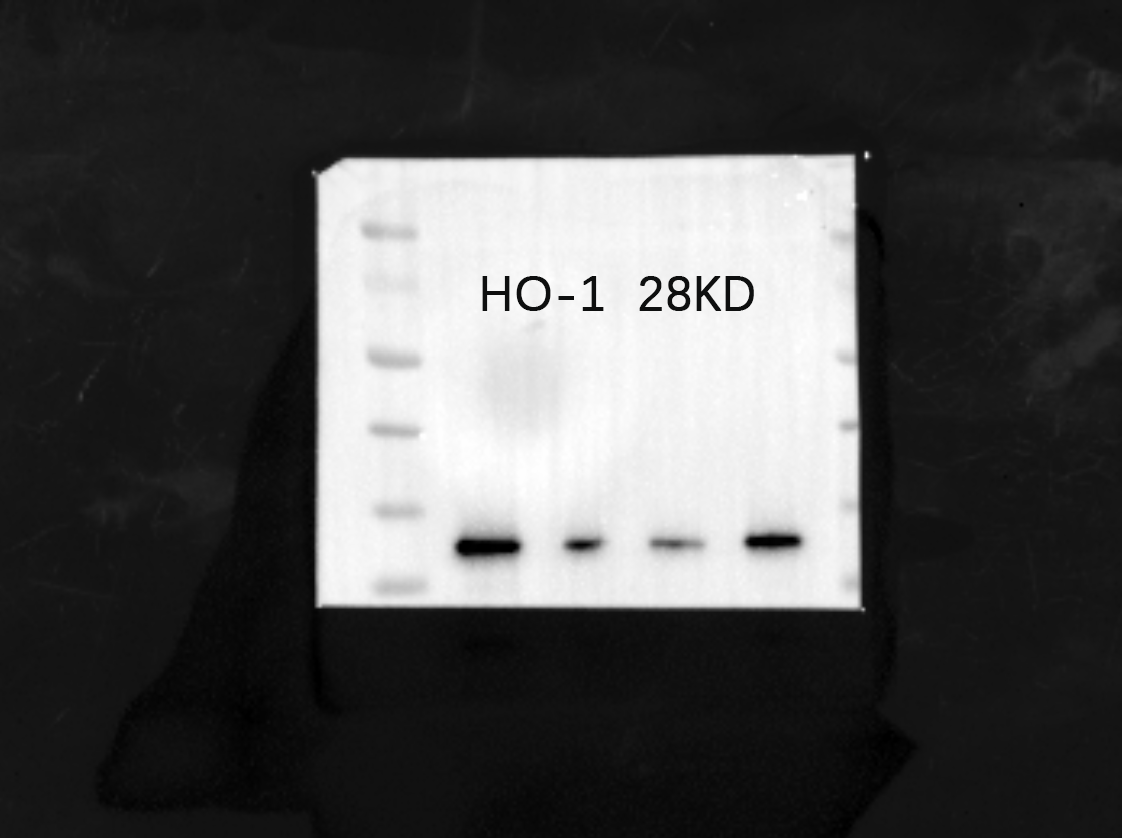

Supplement: Supplementary file 1 [file DataSheet1.ZIP › raw data/Fig.3/Western blot images/Nrf2-HO1/HO-1.tif]

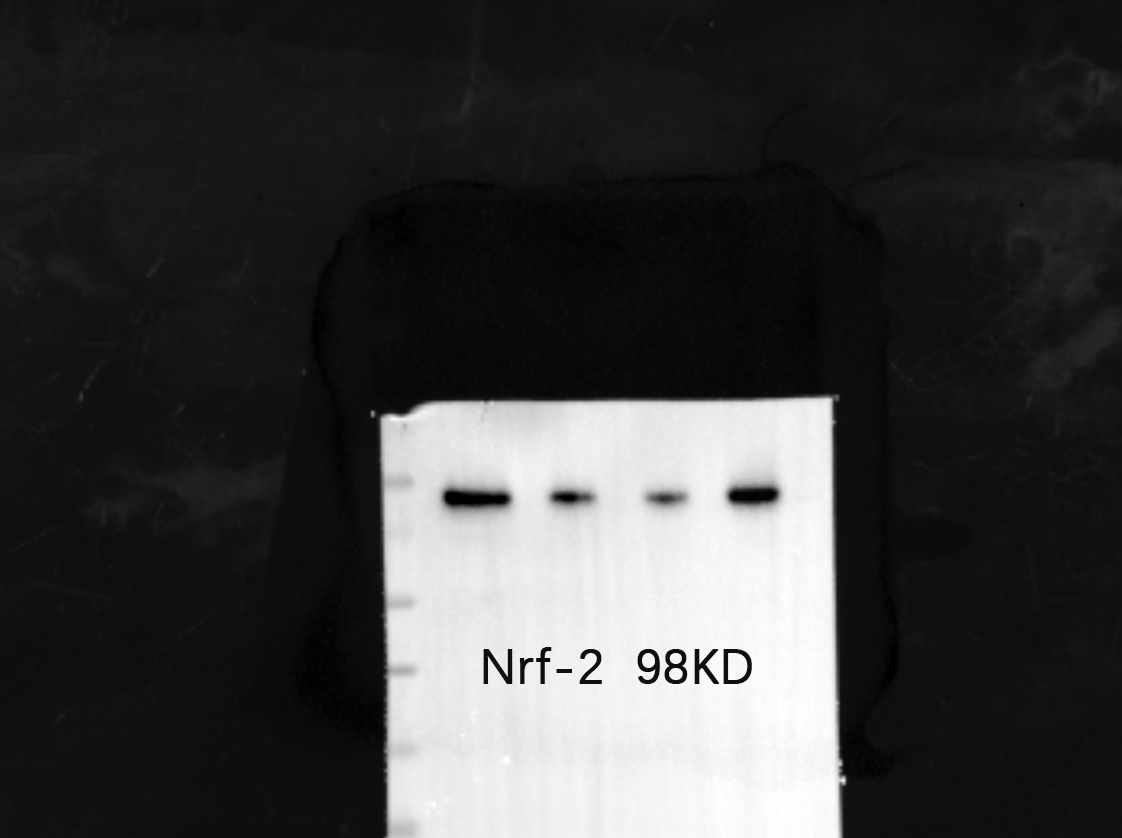

Supplement: Supplementary file 1 [file DataSheet1.ZIP › raw data/Fig.3/Western blot images/Nrf2-HO1/Nrf-2.tif]

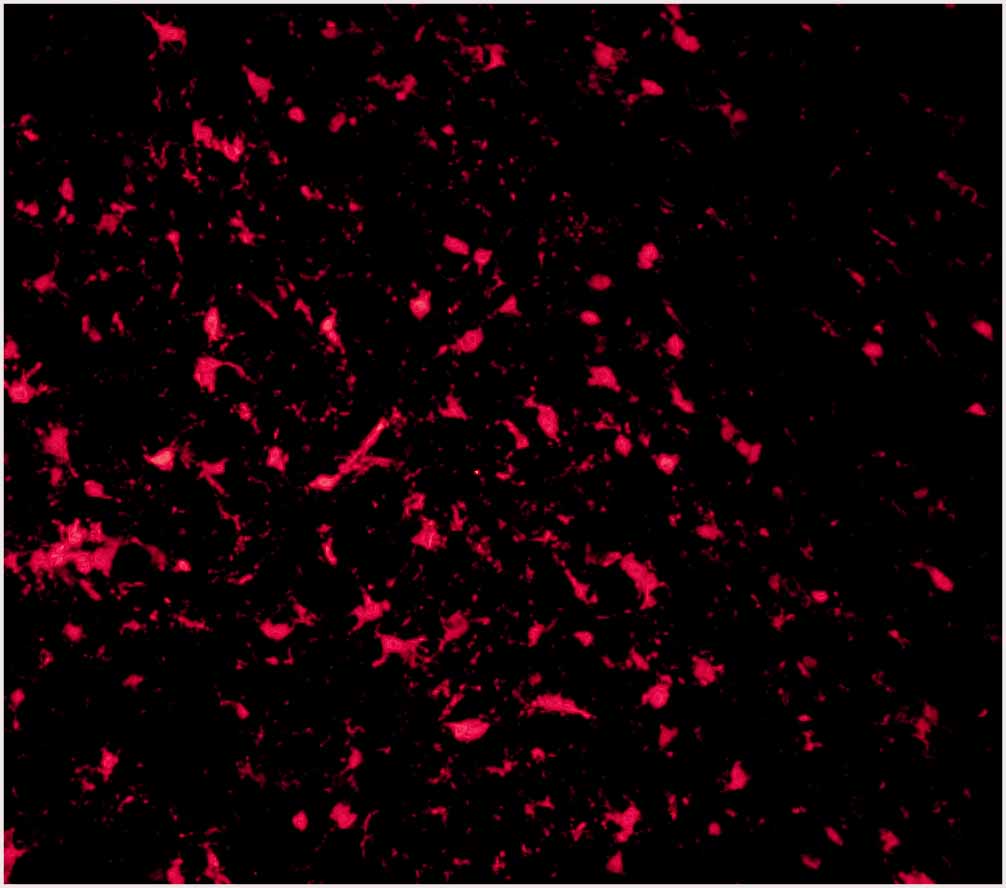

Supplement: Supplementary file 1 [file DataSheet1.ZIP › raw data/Fig.4/CD68/Andro+SCI/1.jpg]

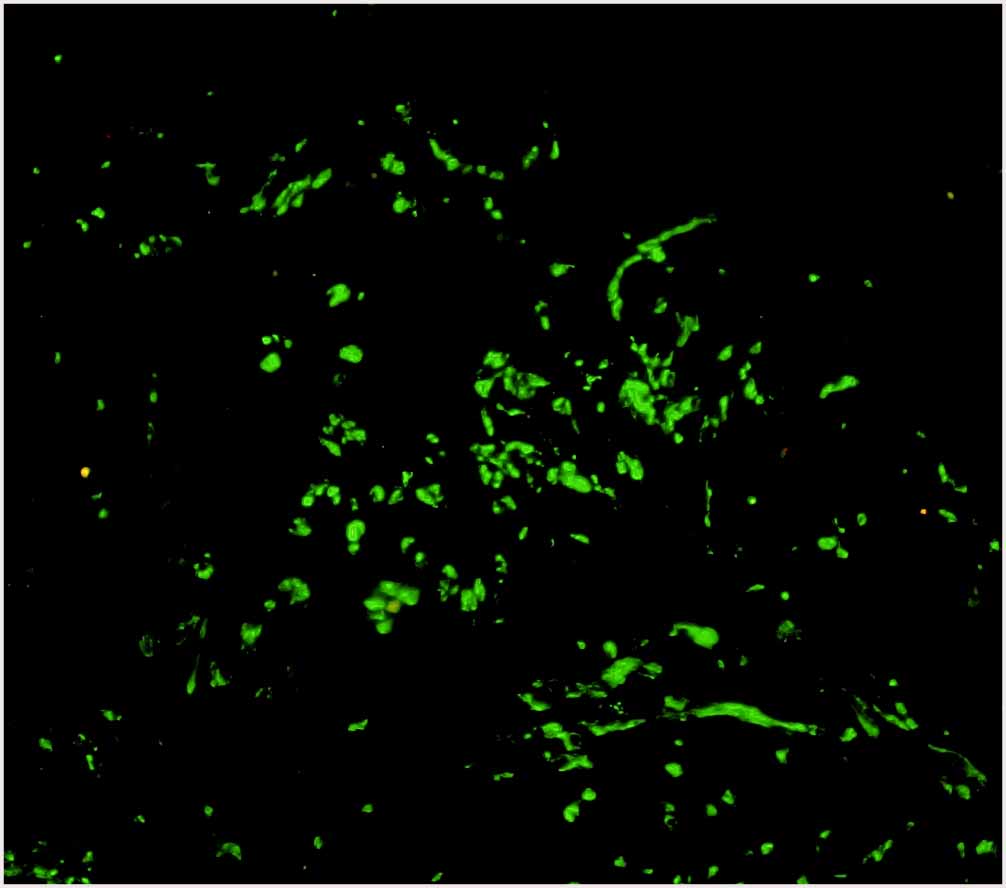

Supplement: Supplementary file 1 [file DataSheet1.ZIP › raw data/Fig.4/CD68/Andro+SCI/2.jpg]

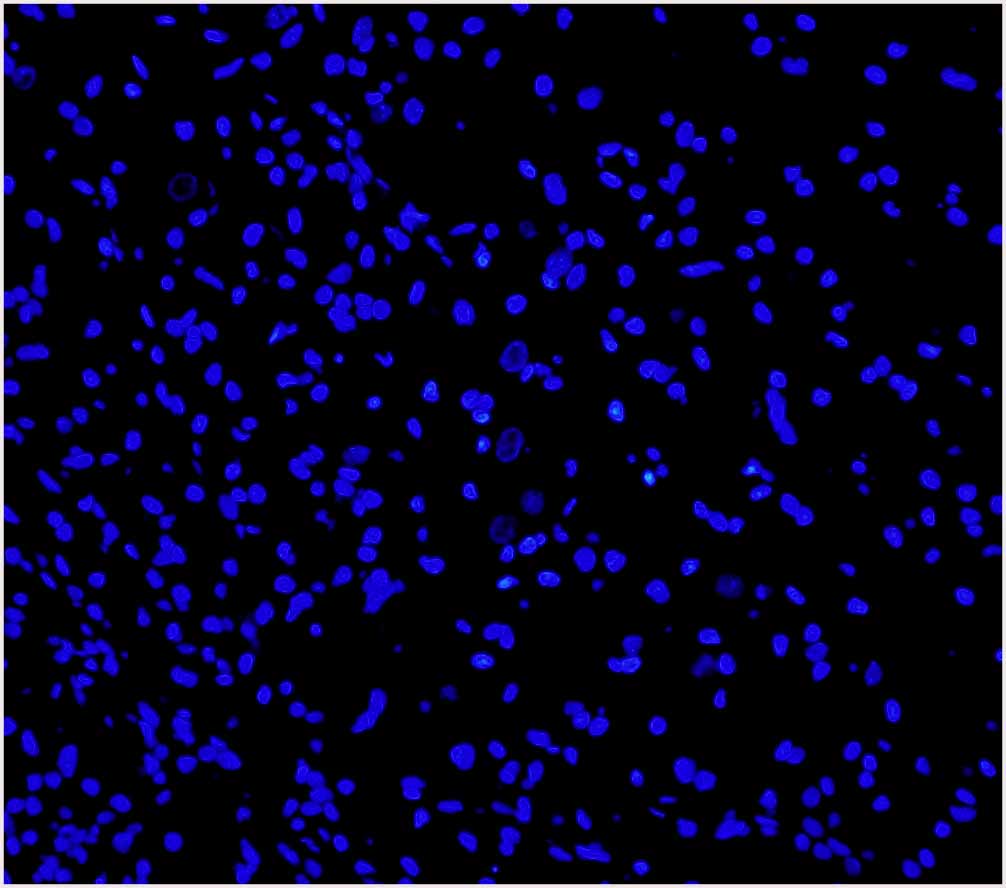

Supplement: Supplementary file 1 [file DataSheet1.ZIP › raw data/Fig.4/CD68/Andro+SCI/3.jpg]

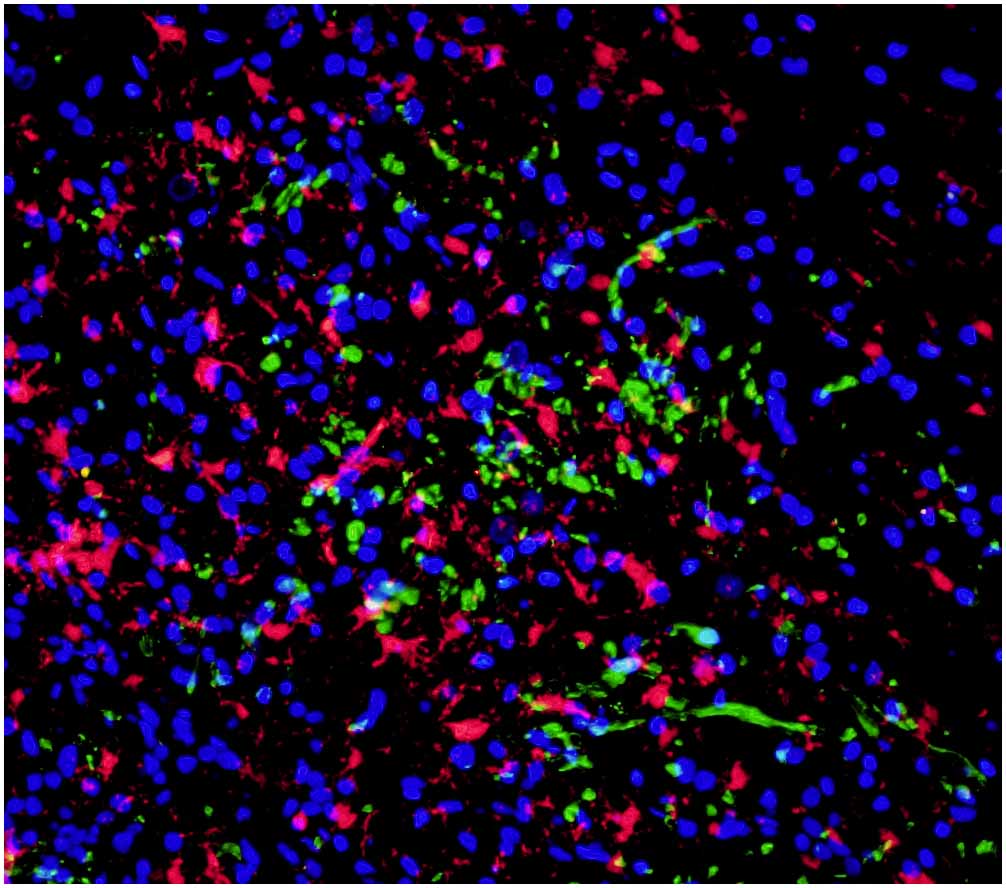

Supplement: Supplementary file 1 [file DataSheet1.ZIP › raw data/Fig.4/CD68/Andro+SCI/4.jpg]

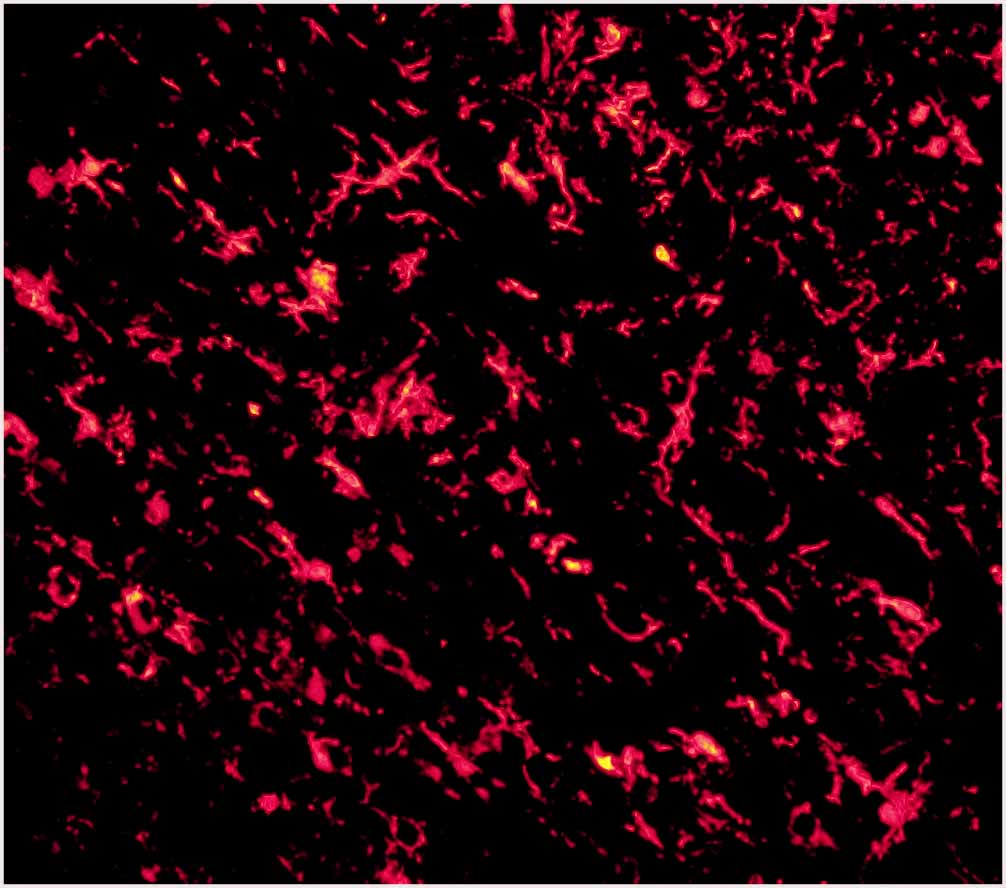

Supplement: Supplementary file 1 [file DataSheet1.ZIP › raw data/Fig.4/CD68/SCI+NS/1.jpg]

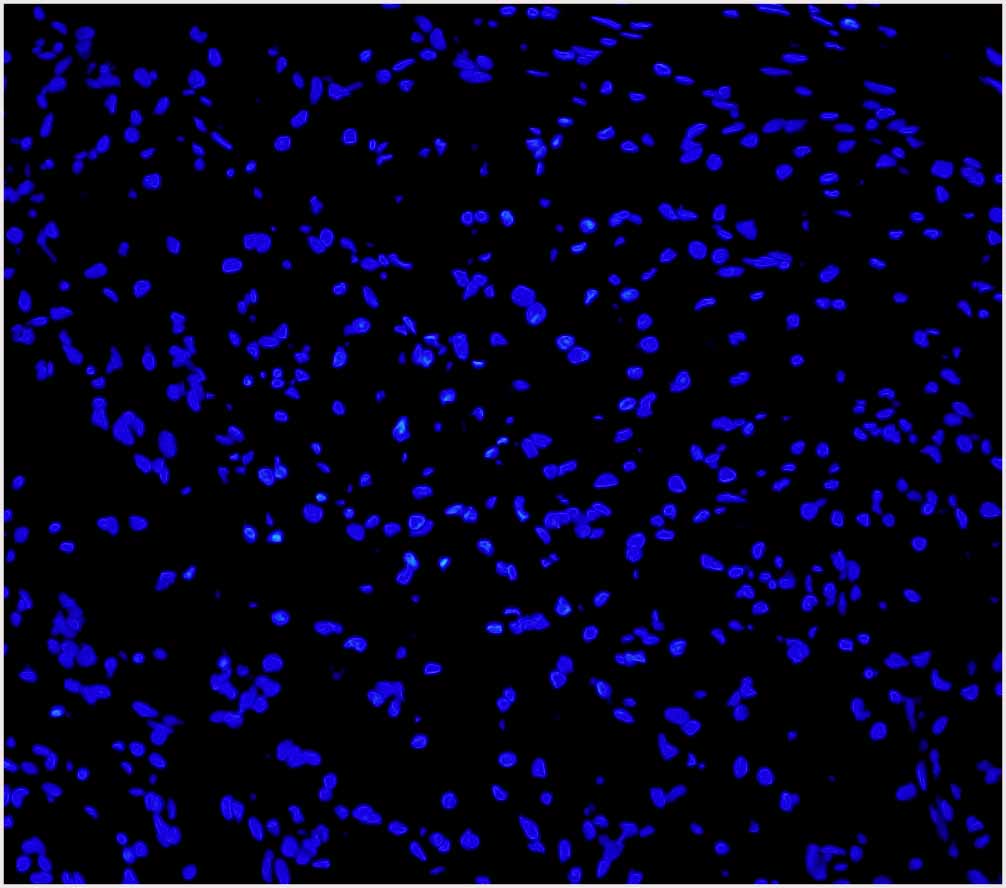

Supplement: Supplementary file 1 [file DataSheet1.ZIP › raw data/Fig.4/CD68/SCI+NS/2.jpg]

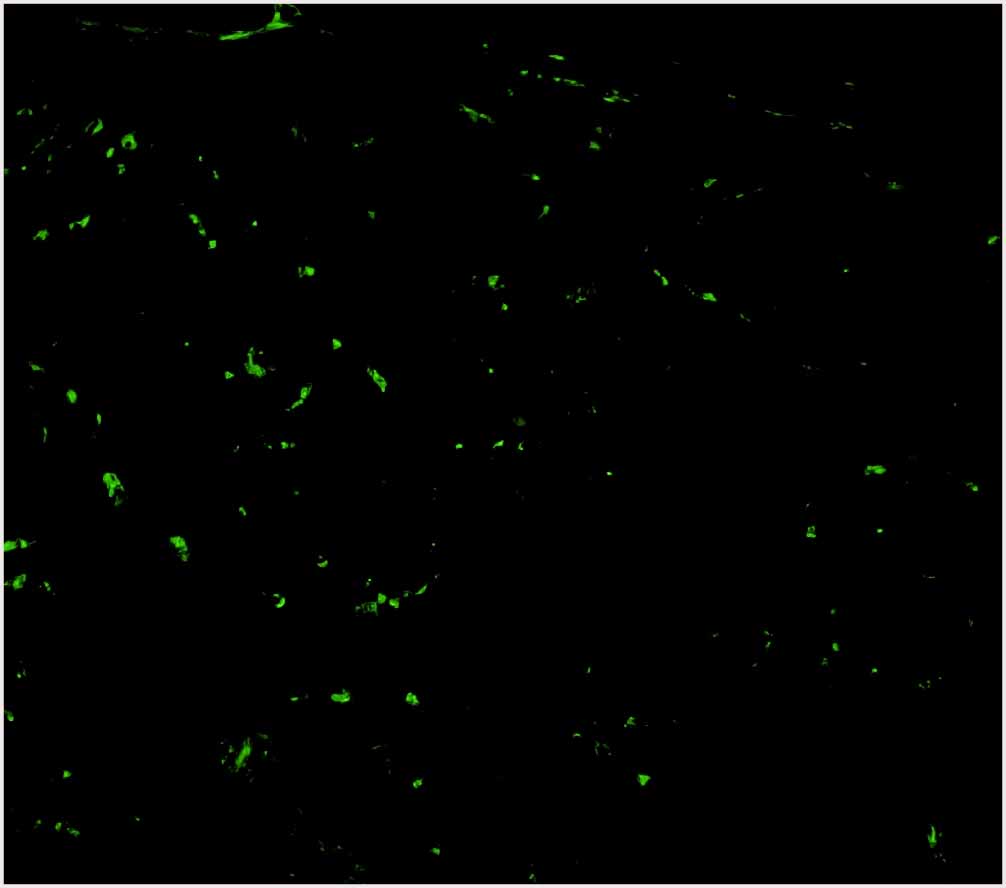

Supplement: Supplementary file 1 [file DataSheet1.ZIP › raw data/Fig.4/CD68/SCI+NS/3.jpg]

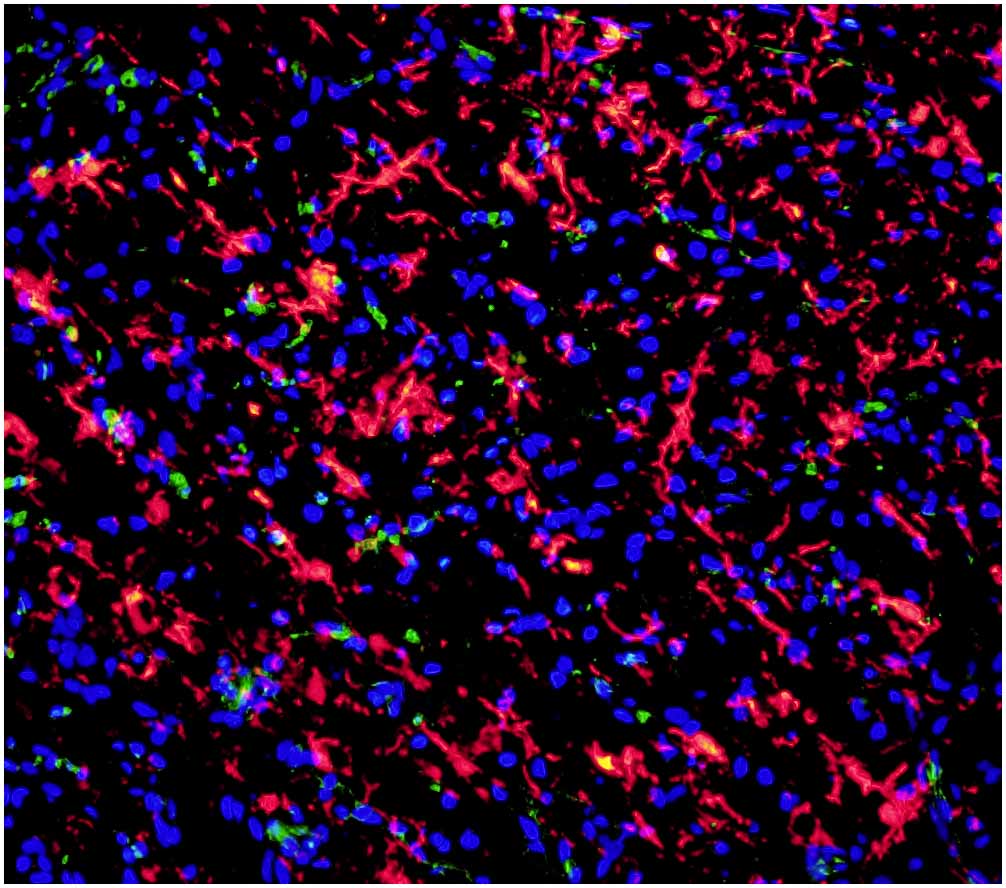

Supplement: Supplementary file 1 [file DataSheet1.ZIP › raw data/Fig.4/CD68/SCI+NS/4.jpg]

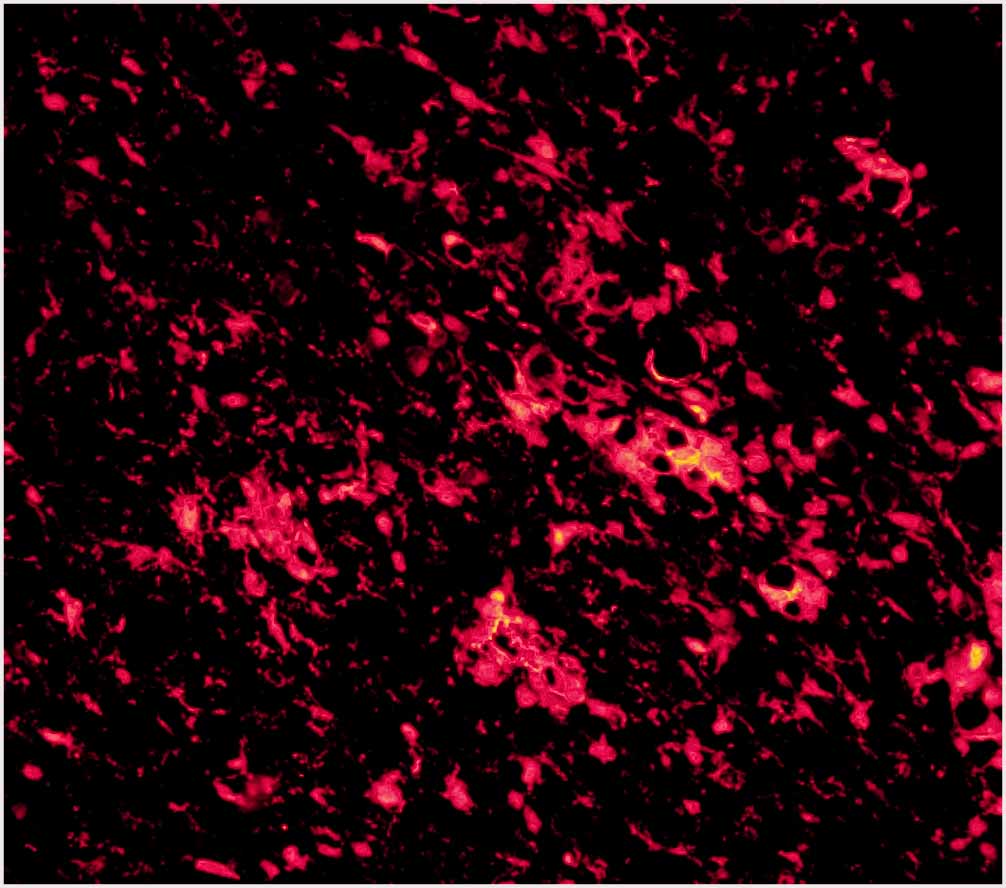

Supplement: Supplementary file 1 [file DataSheet1.ZIP › raw data/Fig.4/CD68/SCI/1.jpg]

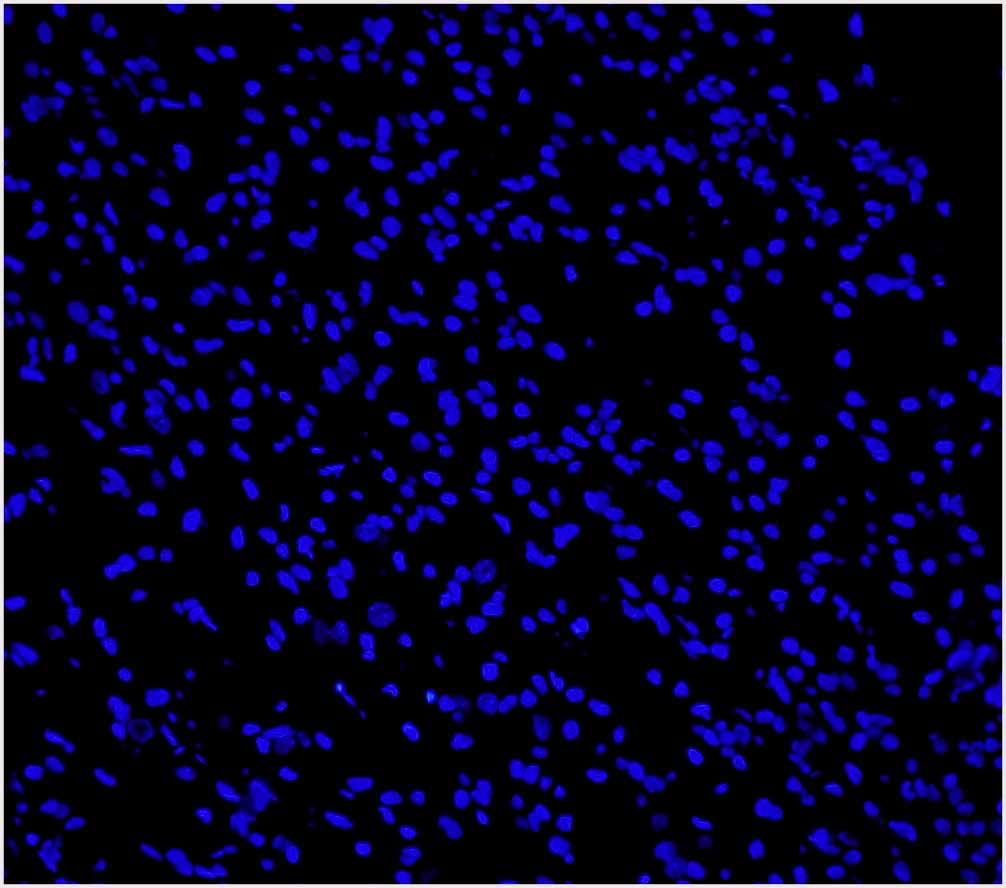

Supplement: Supplementary file 1 [file DataSheet1.ZIP › raw data/Fig.4/CD68/SCI/2.jpg]

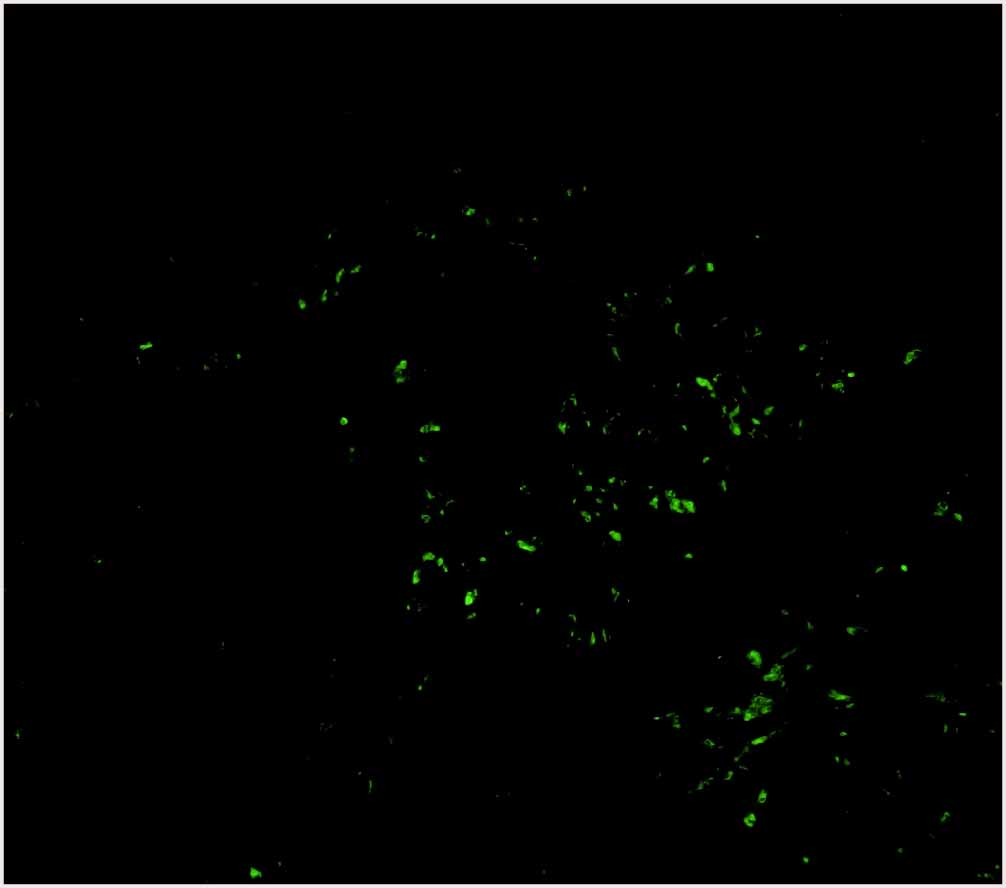

Supplement: Supplementary file 1 [file DataSheet1.ZIP › raw data/Fig.4/CD68/SCI/3.jpg]

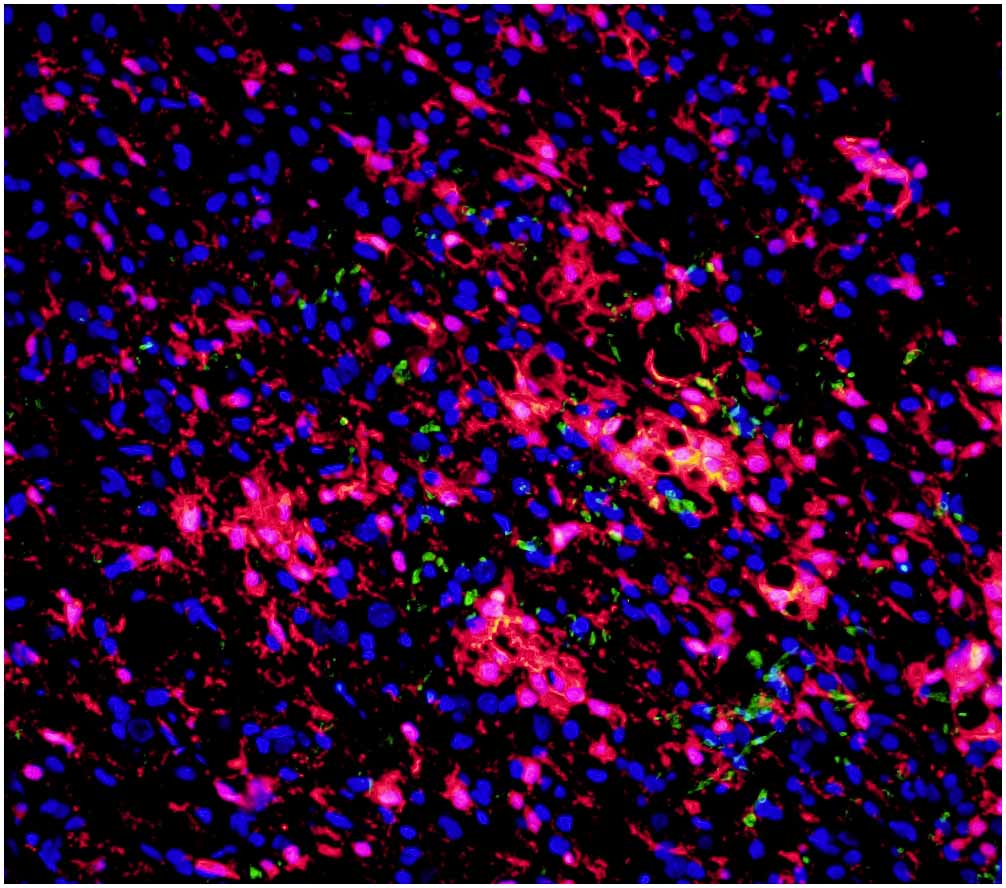

Supplement: Supplementary file 1 [file DataSheet1.ZIP › raw data/Fig.4/CD68/SCI/4.jpg]

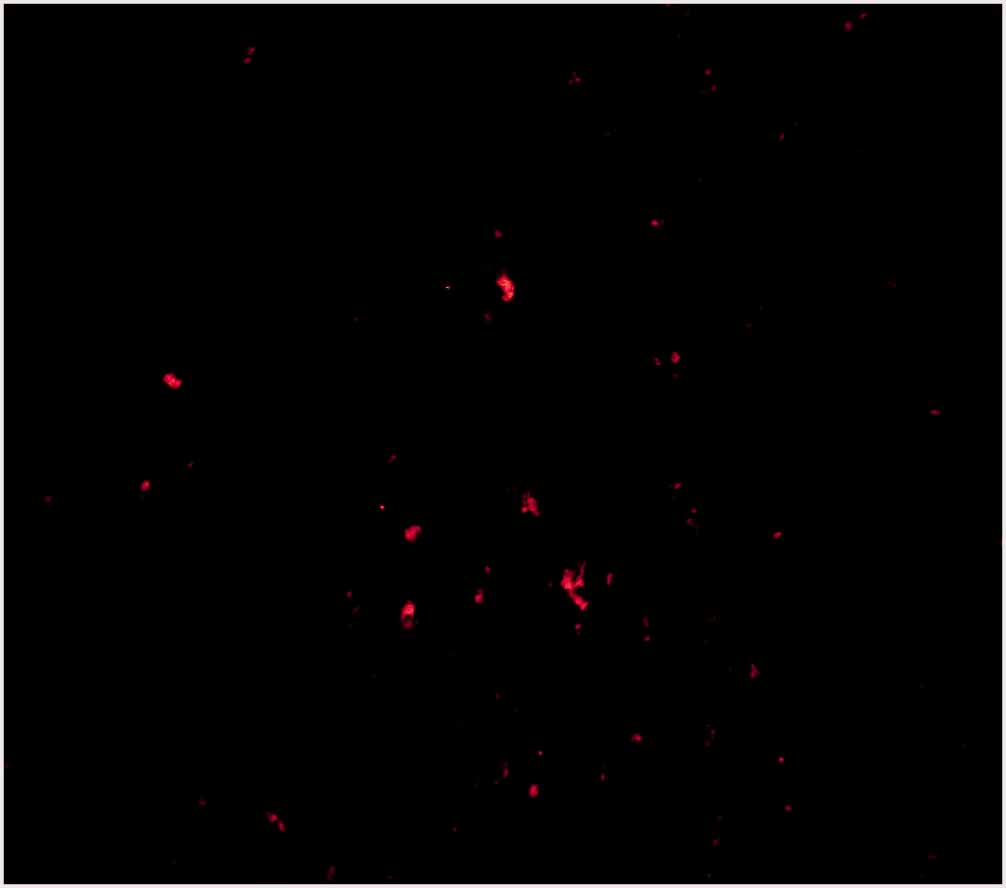

Supplement: Supplementary file 1 [file DataSheet1.ZIP › raw data/Fig.4/CD68/Sham/1.jpg]

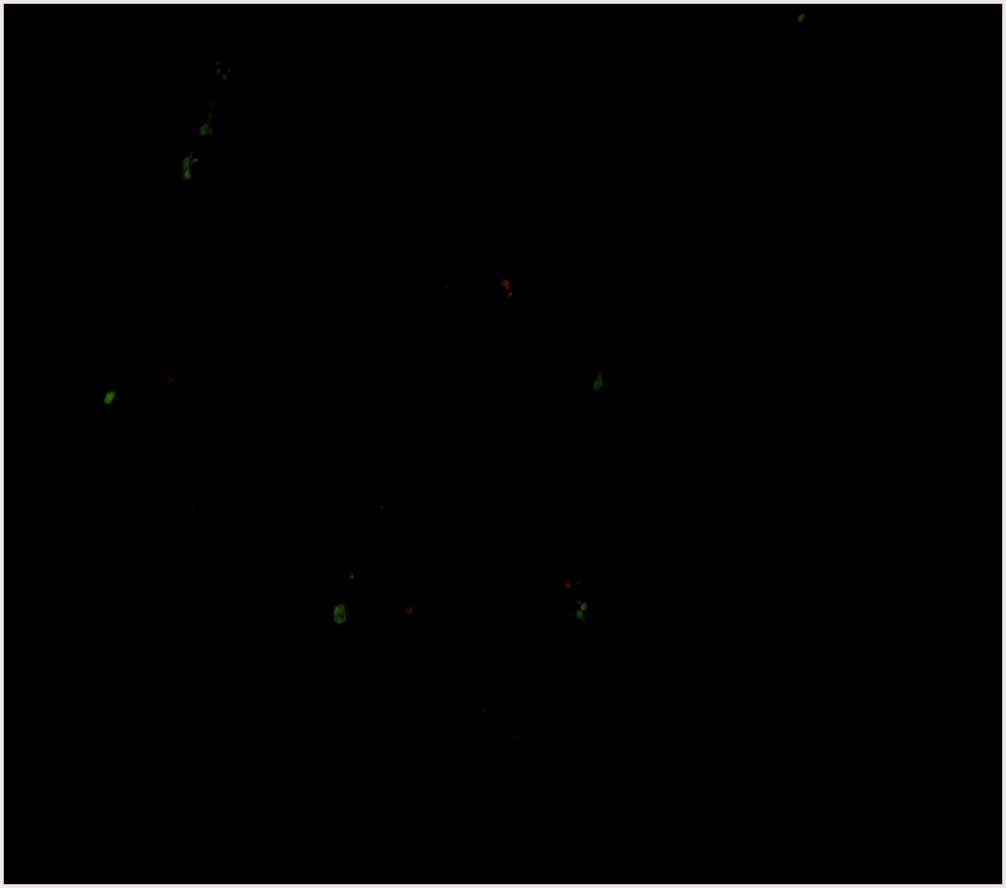

Supplement: Supplementary file 1 [file DataSheet1.ZIP › raw data/Fig.4/CD68/Sham/2.jpg]

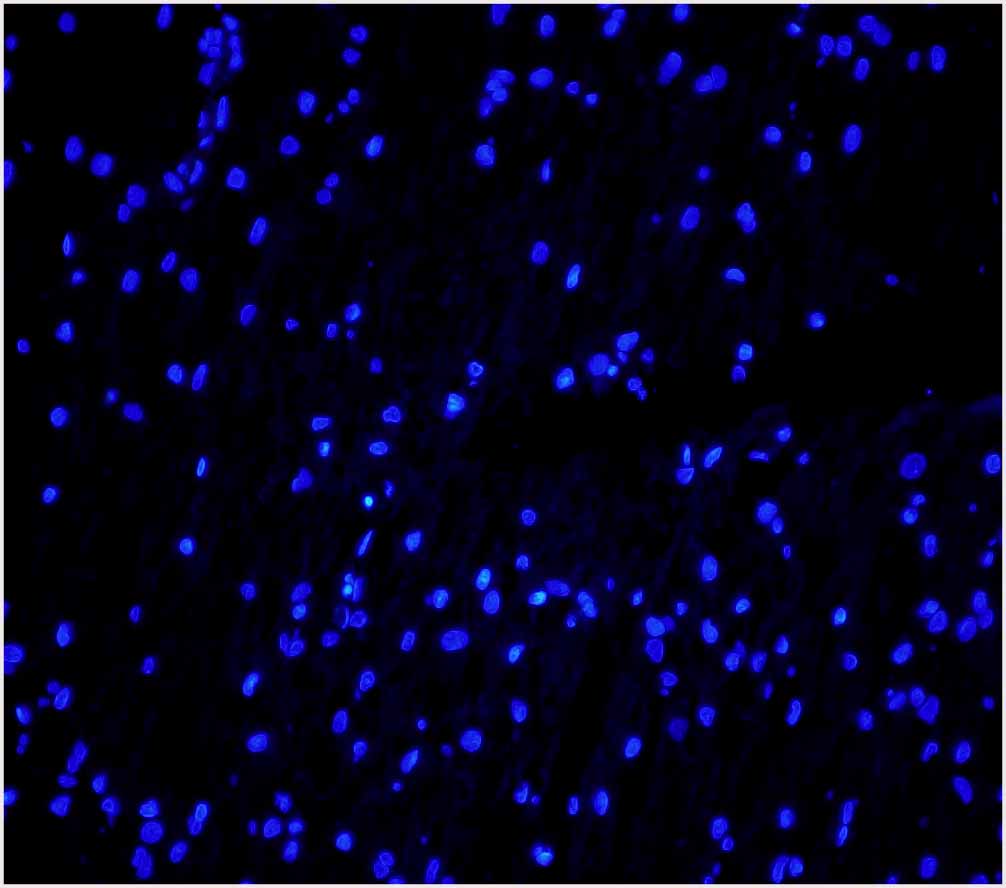

Supplement: Supplementary file 1 [file DataSheet1.ZIP › raw data/Fig.4/CD68/Sham/3.jpg]

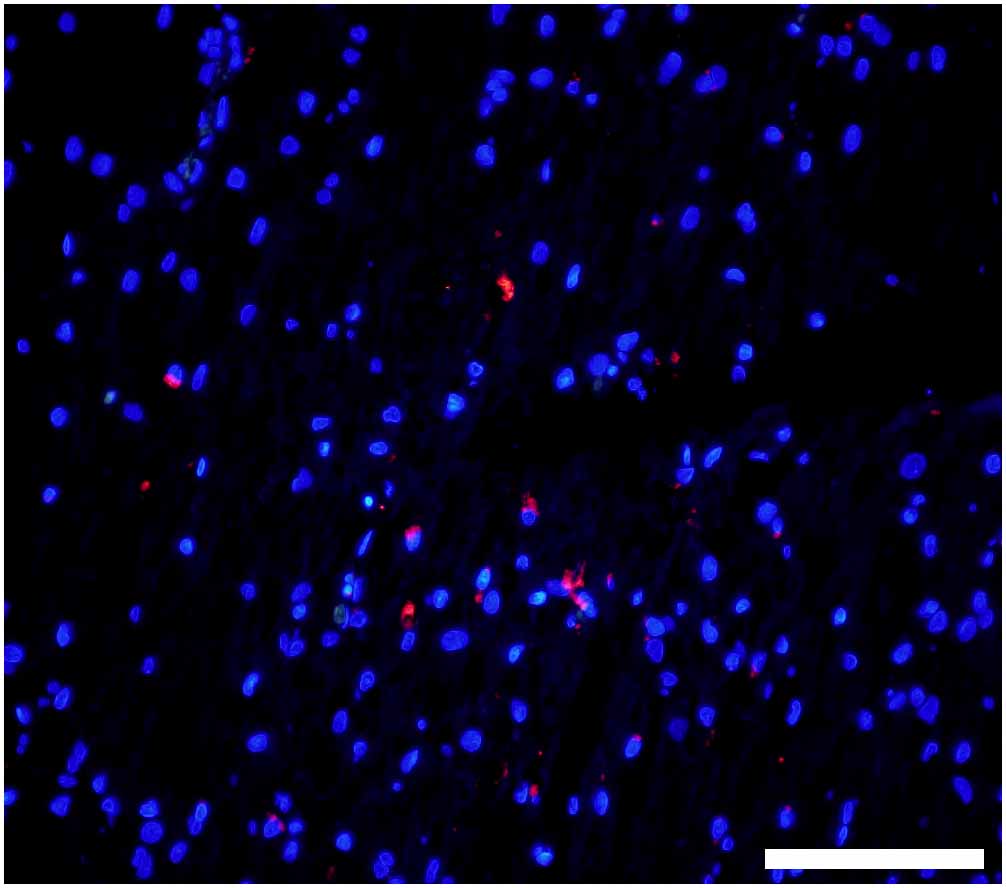

Supplement: Supplementary file 1 [file DataSheet1.ZIP › raw data/Fig.4/CD68/Sham/4-▒Ω│▀.jpg]

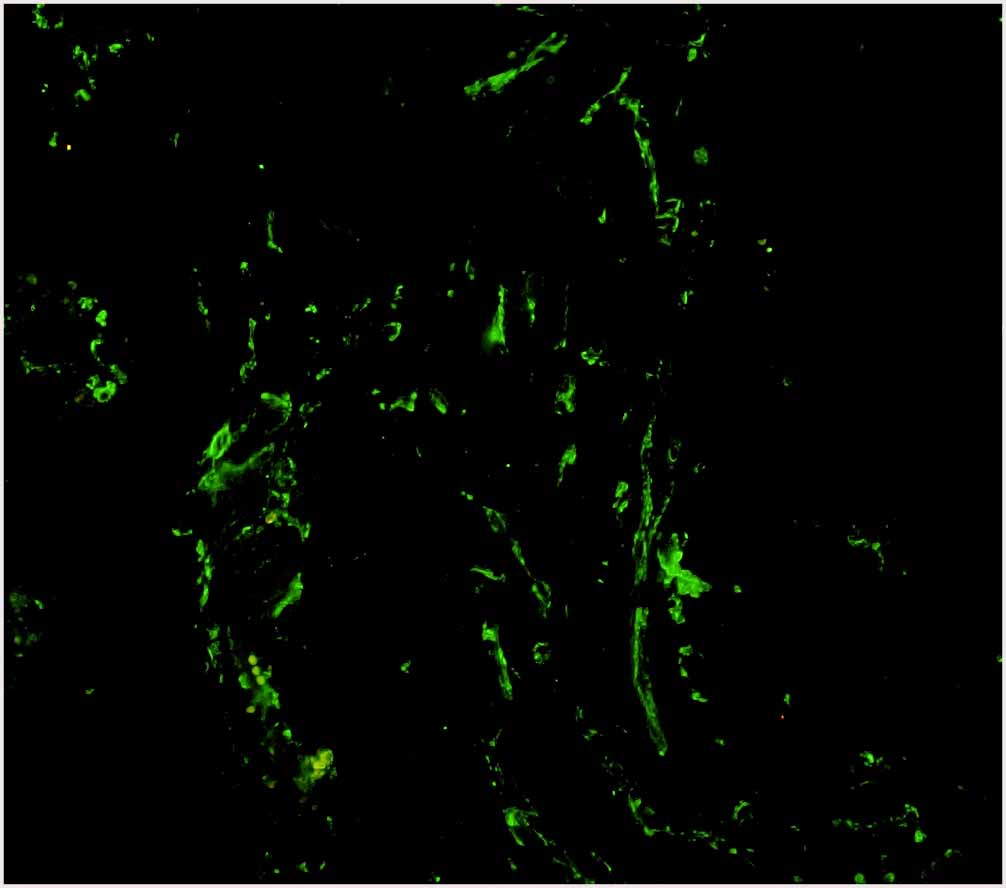

Supplement: Supplementary file 1 [file DataSheet1.ZIP › raw data/Fig.4/Iba-1/Andro+SCI/1.jpg]

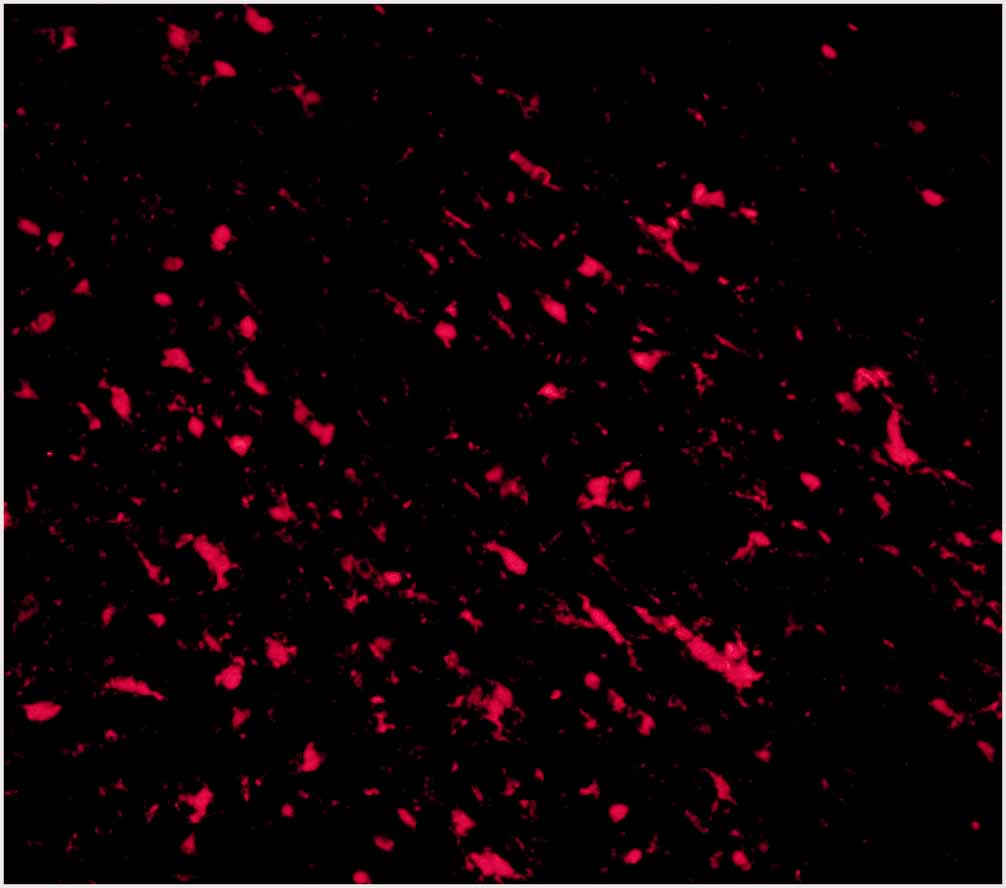

Supplement: Supplementary file 1 [file DataSheet1.ZIP › raw data/Fig.4/Iba-1/Andro+SCI/2.jpg]

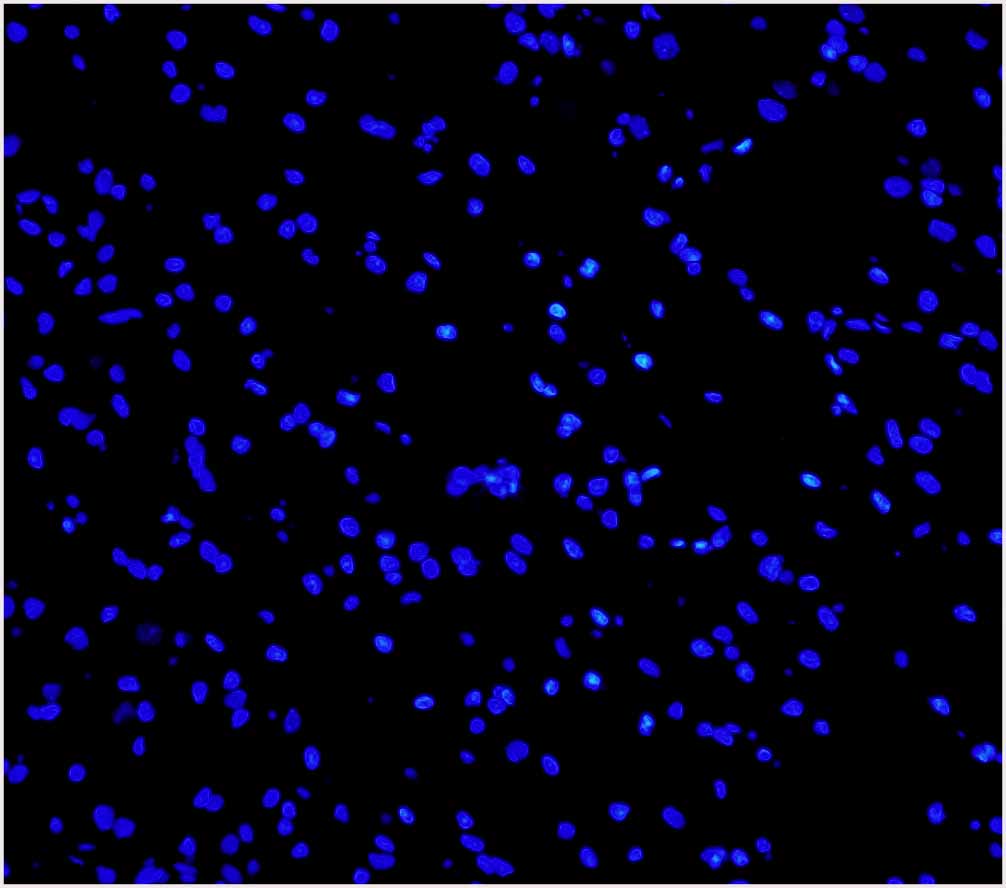

Supplement: Supplementary file 1 [file DataSheet1.ZIP › raw data/Fig.4/Iba-1/Andro+SCI/3.jpg]

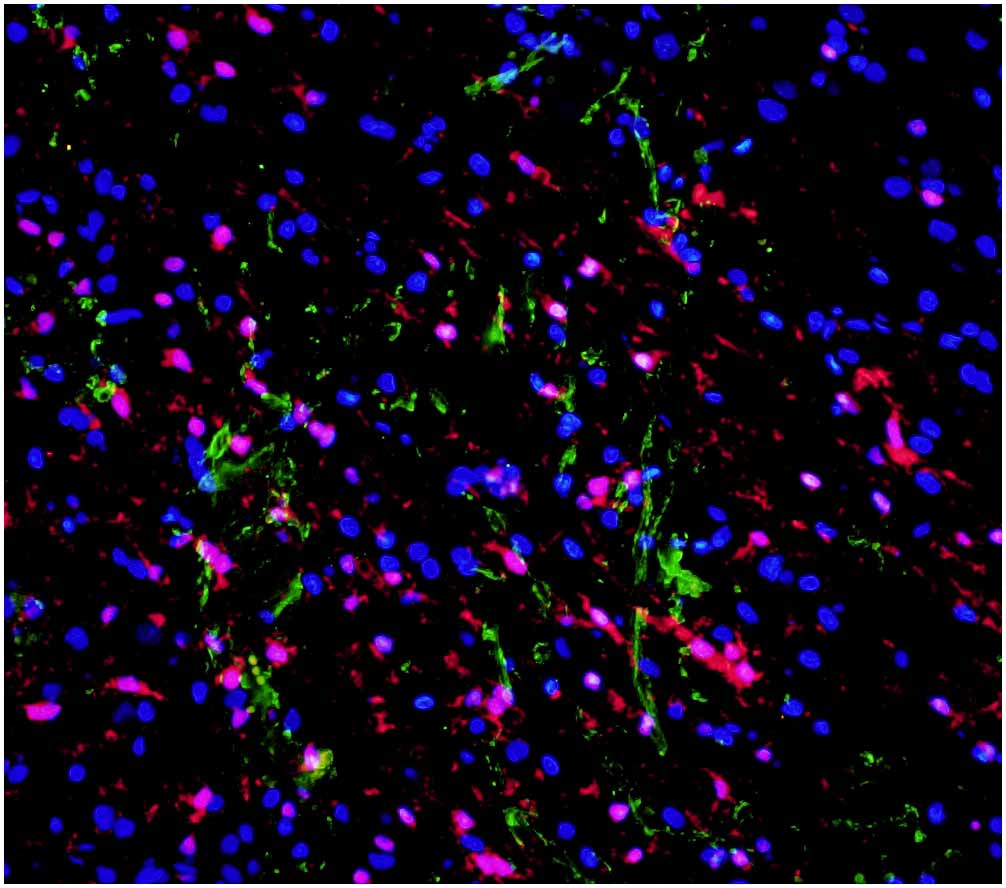

Supplement: Supplementary file 1 [file DataSheet1.ZIP › raw data/Fig.4/Iba-1/Andro+SCI/4.jpg]

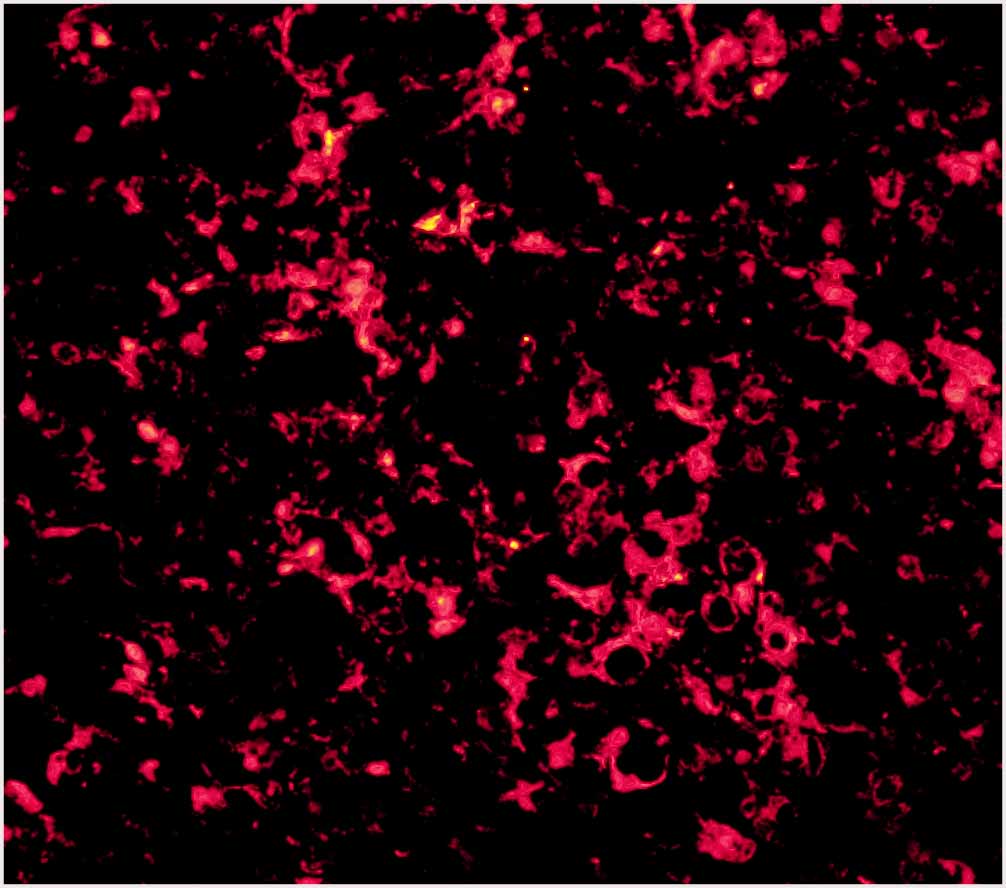

Supplement: Supplementary file 1 [file DataSheet1.ZIP › raw data/Fig.4/Iba-1/SCI+NS/1.jpg]

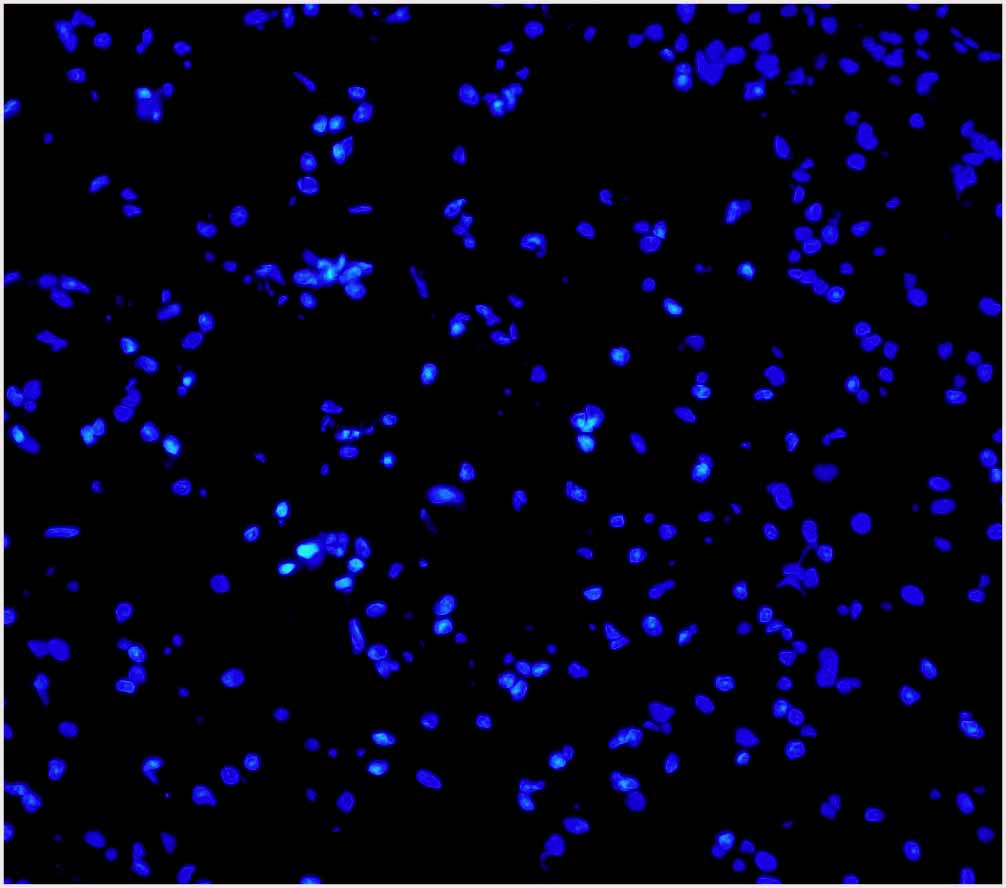

Supplement: Supplementary file 1 [file DataSheet1.ZIP › raw data/Fig.4/Iba-1/SCI+NS/2.jpg]

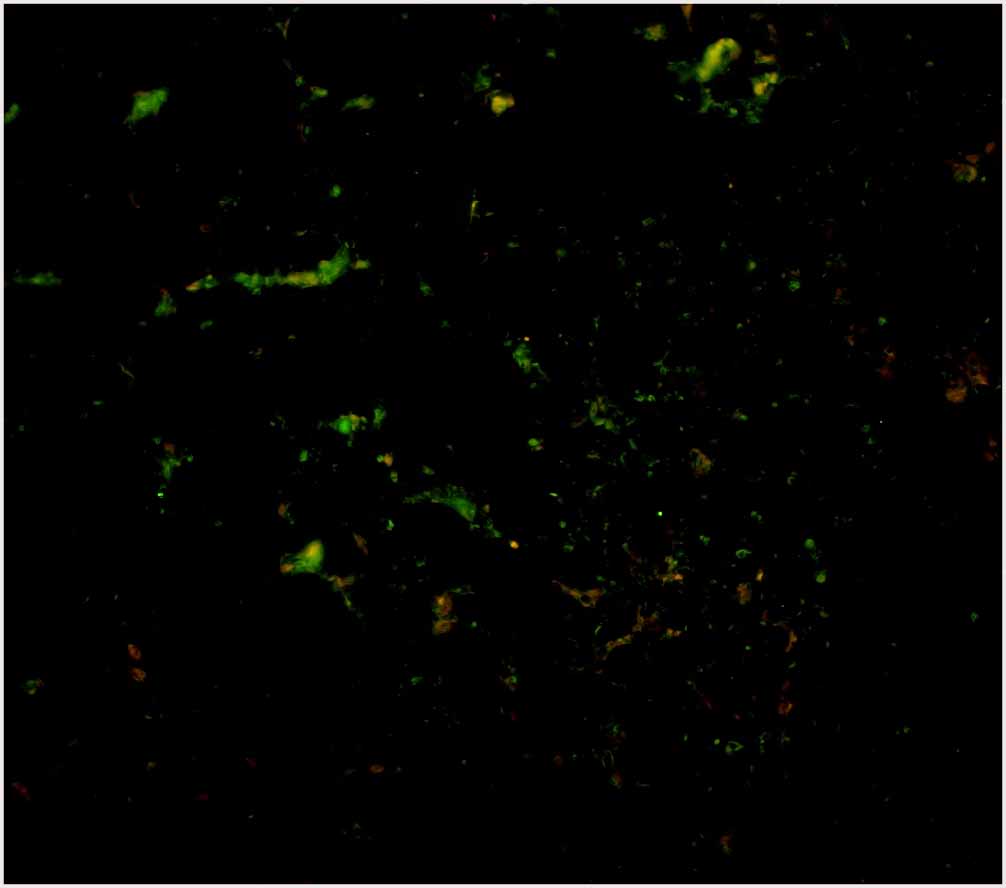

Supplement: Supplementary file 1 [file DataSheet1.ZIP › raw data/Fig.4/Iba-1/SCI+NS/3.jpg]

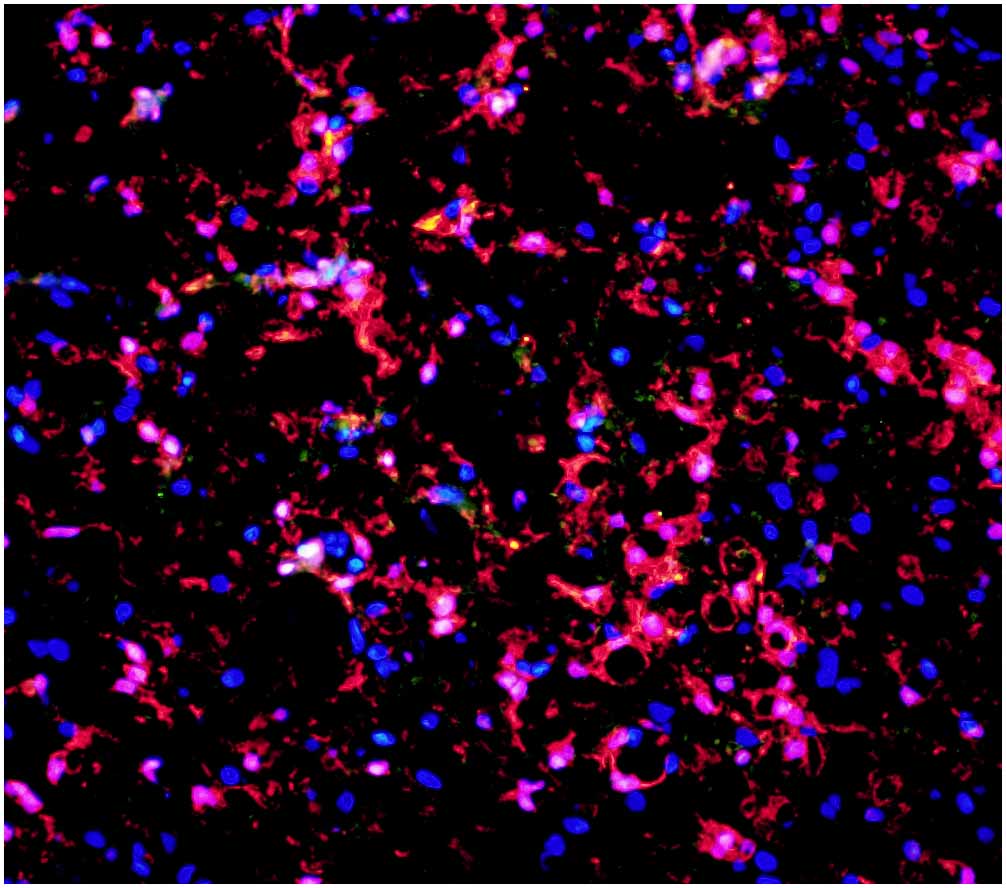

Supplement: Supplementary file 1 [file DataSheet1.ZIP › raw data/Fig.4/Iba-1/SCI+NS/4.jpg]

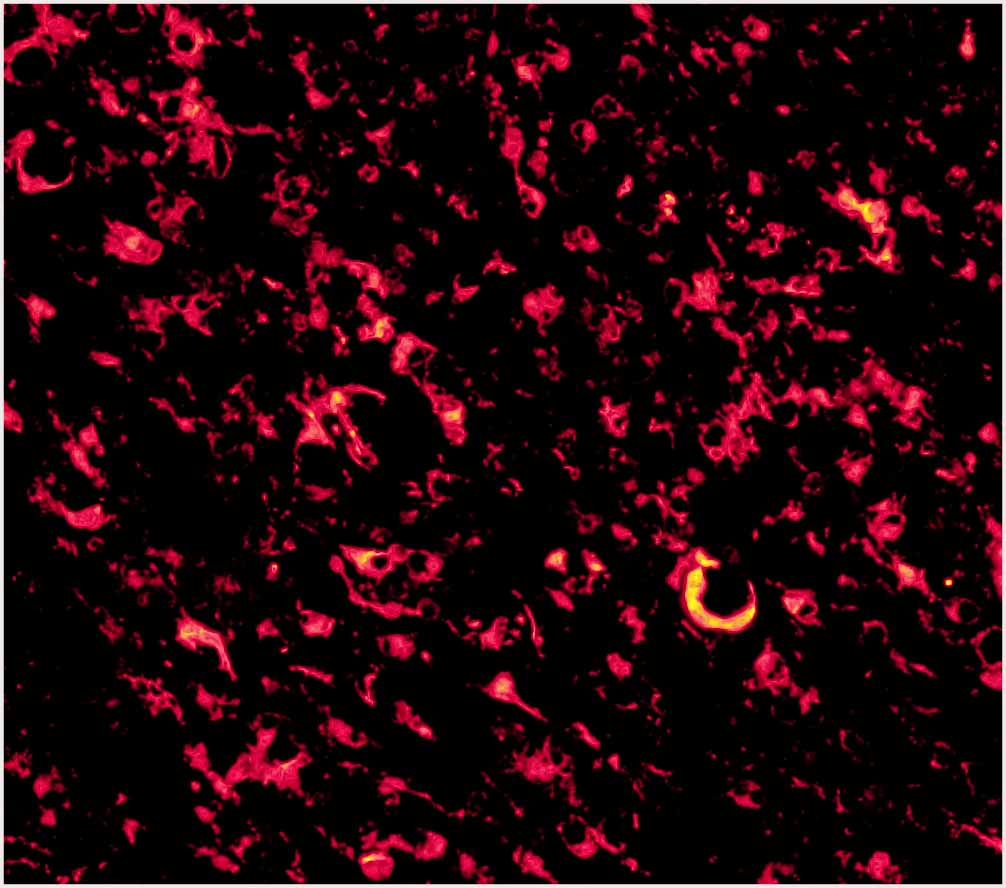

Supplement: Supplementary file 1 [file DataSheet1.ZIP › raw data/Fig.4/Iba-1/SCI/1.jpg]

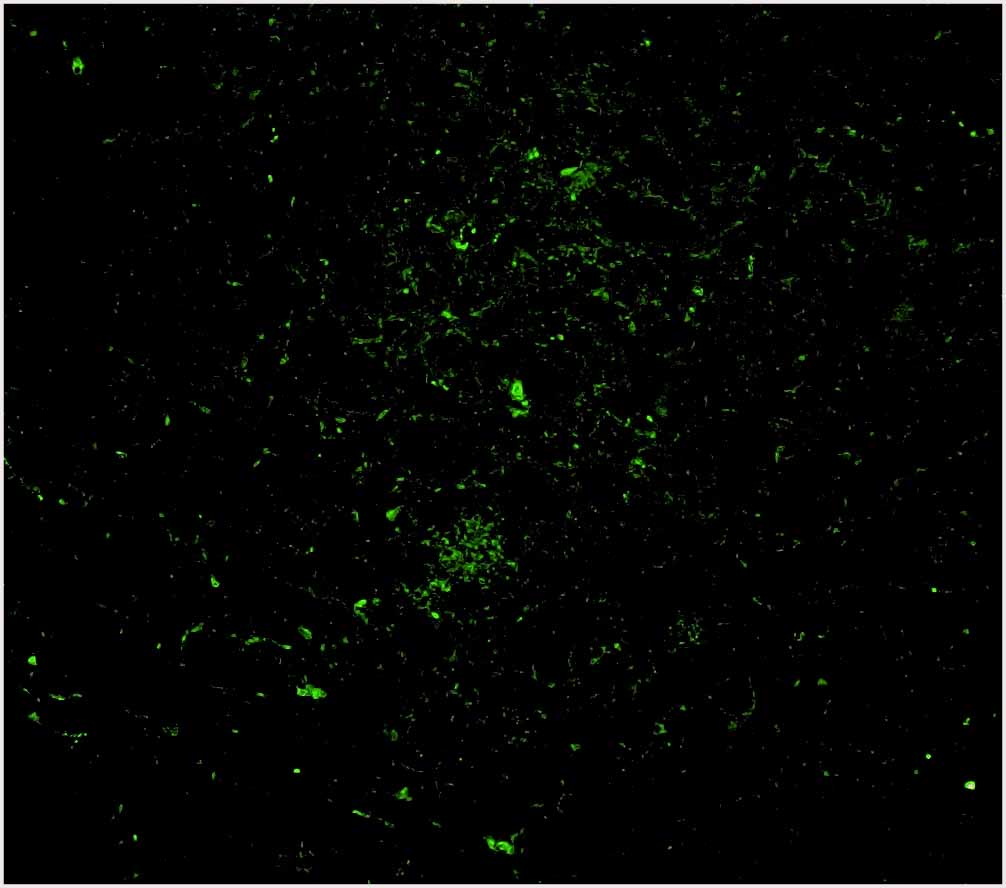

Supplement: Supplementary file 1 [file DataSheet1.ZIP › raw data/Fig.4/Iba-1/SCI/2.jpg]

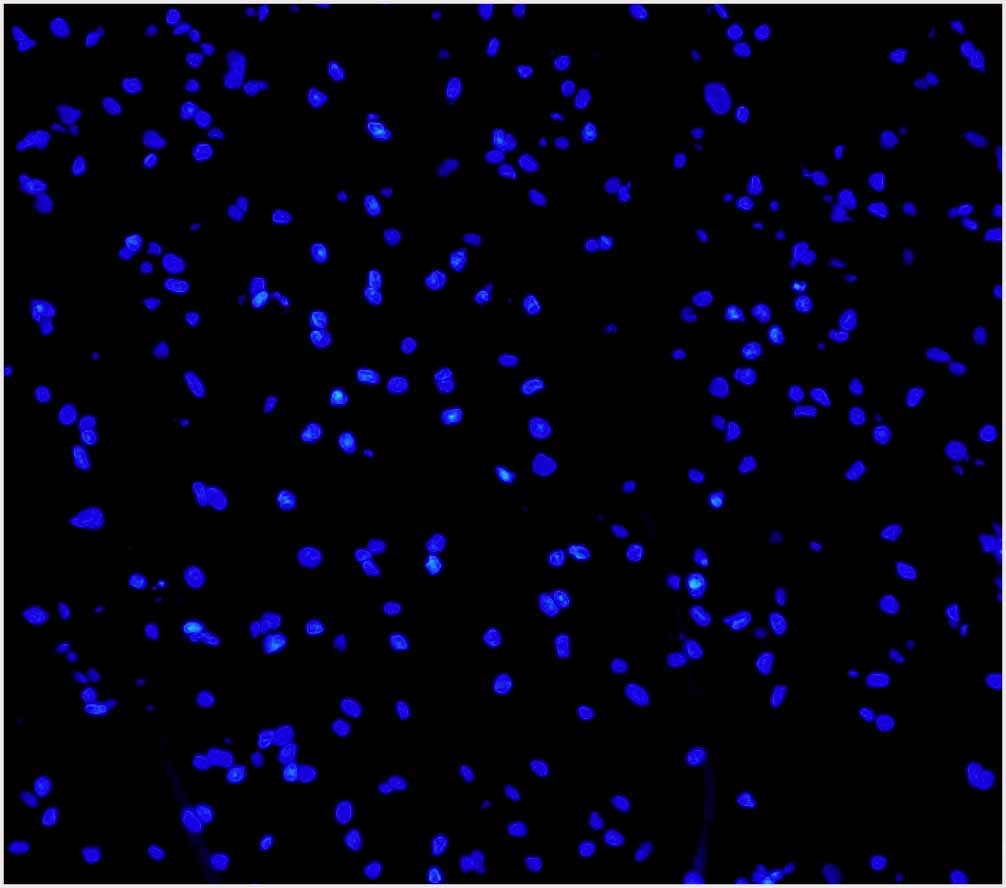

Supplement: Supplementary file 1 [file DataSheet1.ZIP › raw data/Fig.4/Iba-1/SCI/3.jpg]

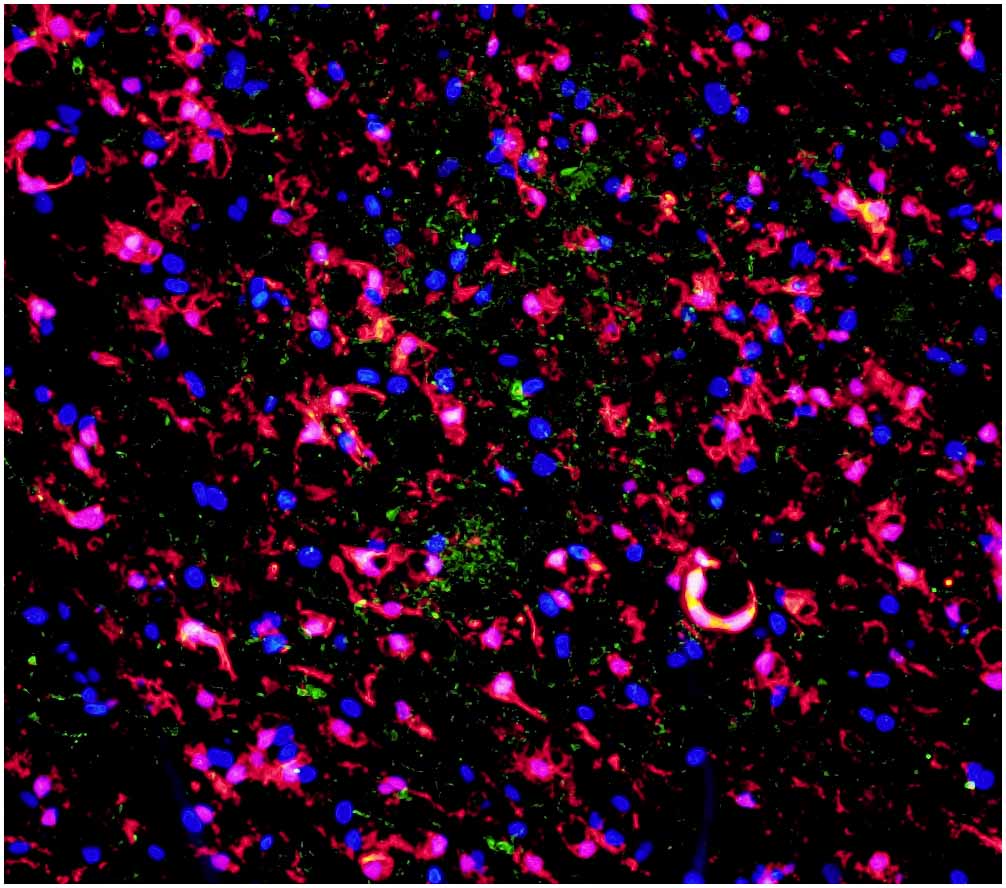

Supplement: Supplementary file 1 [file DataSheet1.ZIP › raw data/Fig.4/Iba-1/SCI/4.jpg]

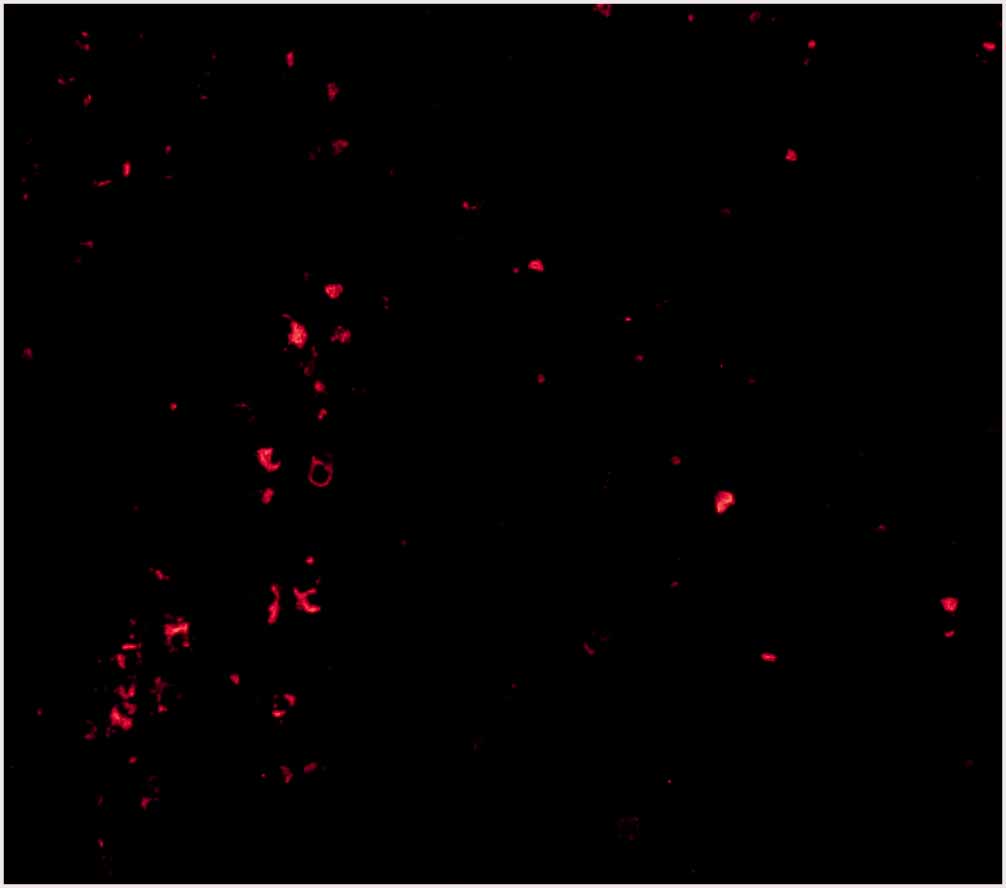

Supplement: Supplementary file 1 [file DataSheet1.ZIP › raw data/Fig.4/Iba-1/Sham/1.jpg]

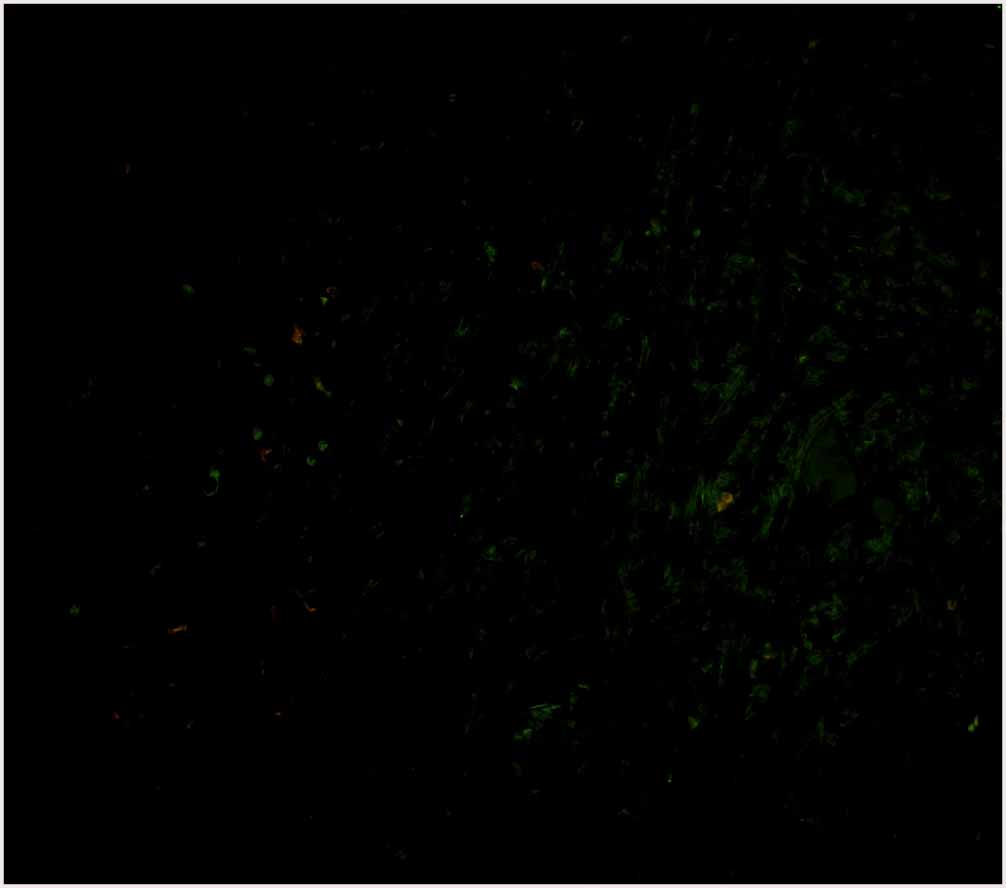

Supplement: Supplementary file 1 [file DataSheet1.ZIP › raw data/Fig.4/Iba-1/Sham/2.jpg]

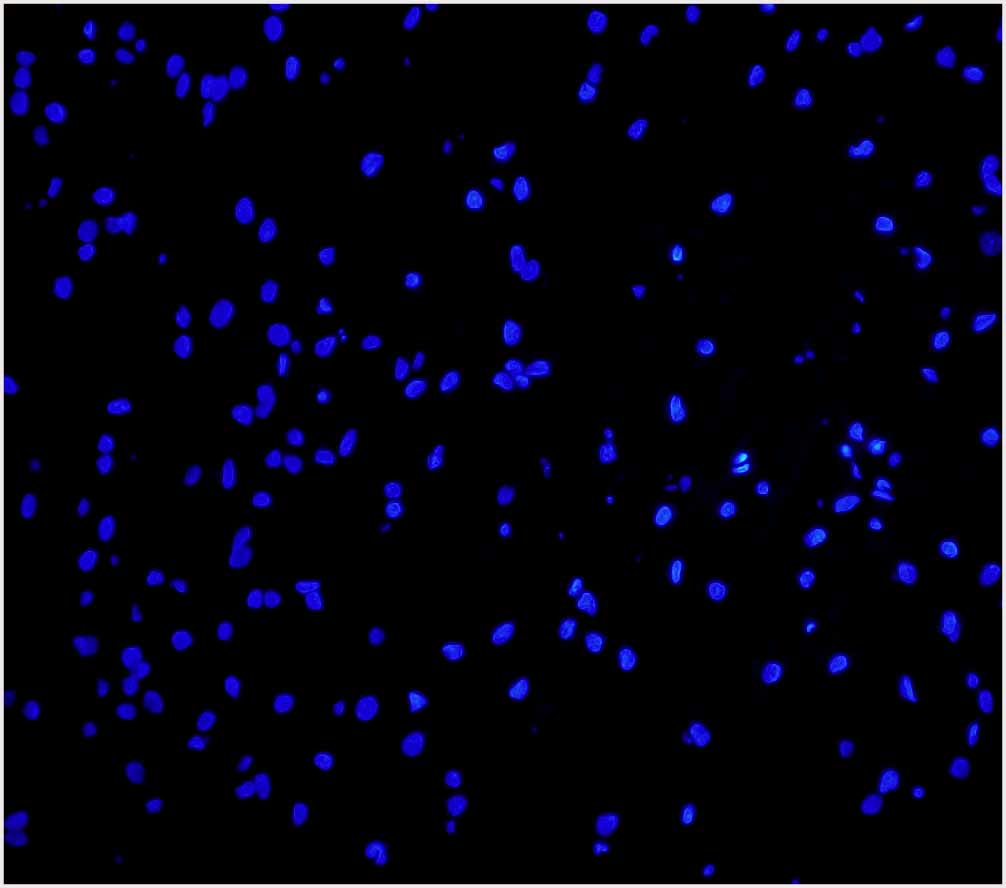

Supplement: Supplementary file 1 [file DataSheet1.ZIP › raw data/Fig.4/Iba-1/Sham/3.jpg]

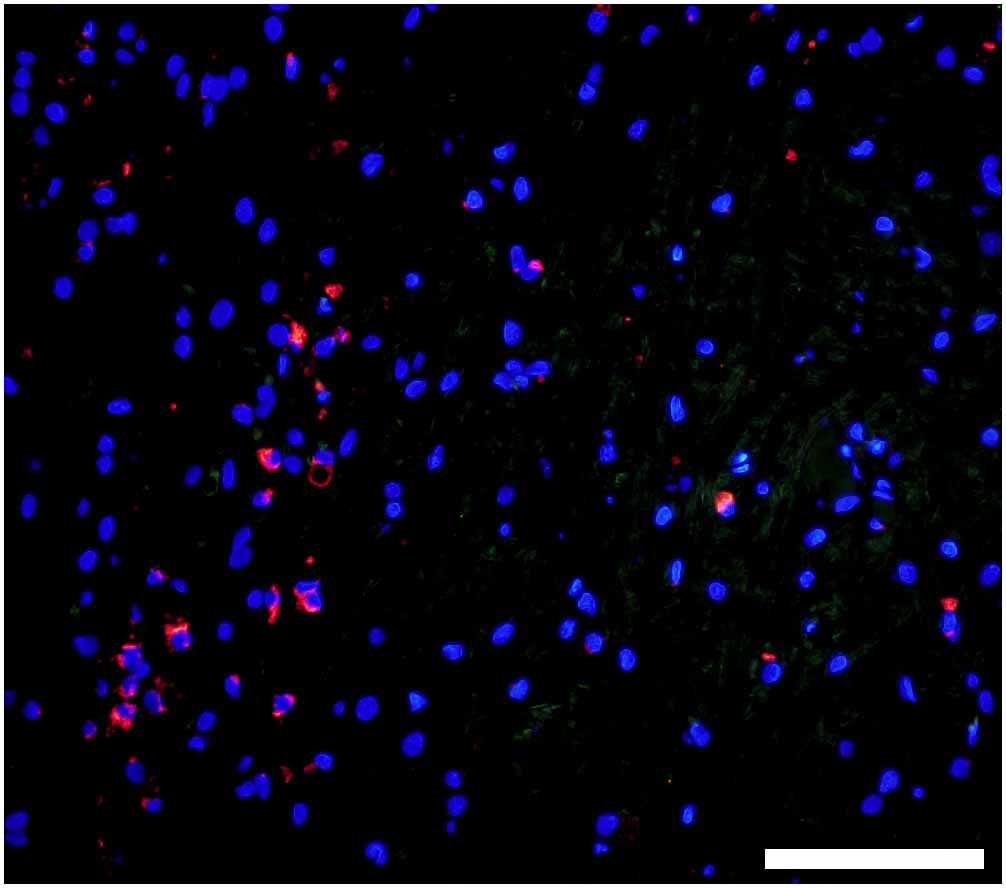

Supplement: Supplementary file 1 [file DataSheet1.ZIP › raw data/Fig.4/Iba-1/Sham/4.jpg]

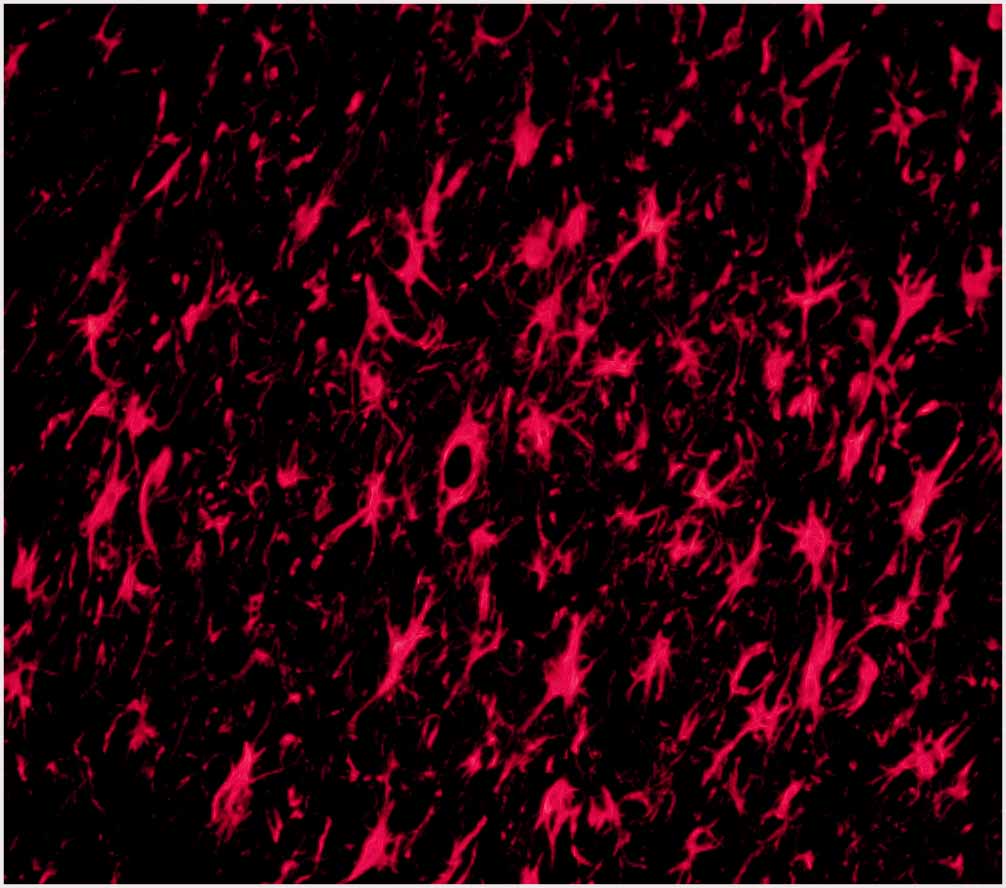

Supplement: Supplementary file 1 [file DataSheet1.ZIP › raw data/Fig.5/IF/SCI+Andro/1.jpg]

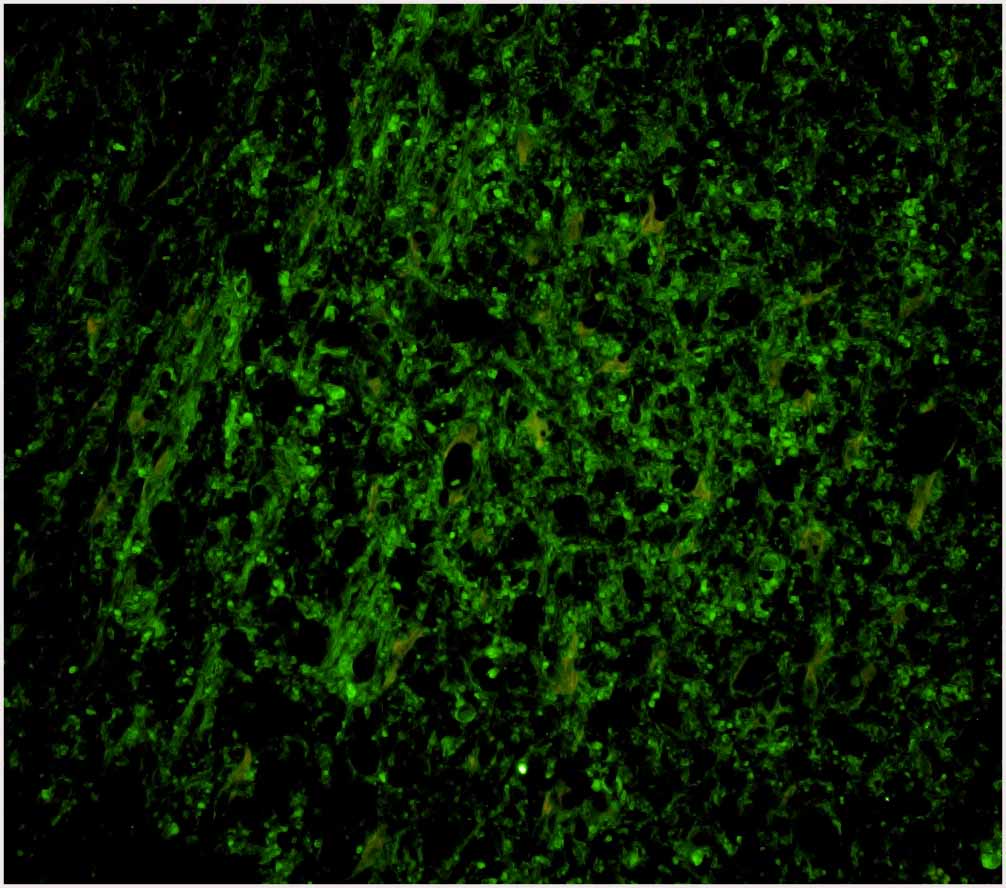

Supplement: Supplementary file 1 [file DataSheet1.ZIP › raw data/Fig.5/IF/SCI+Andro/2.jpg]

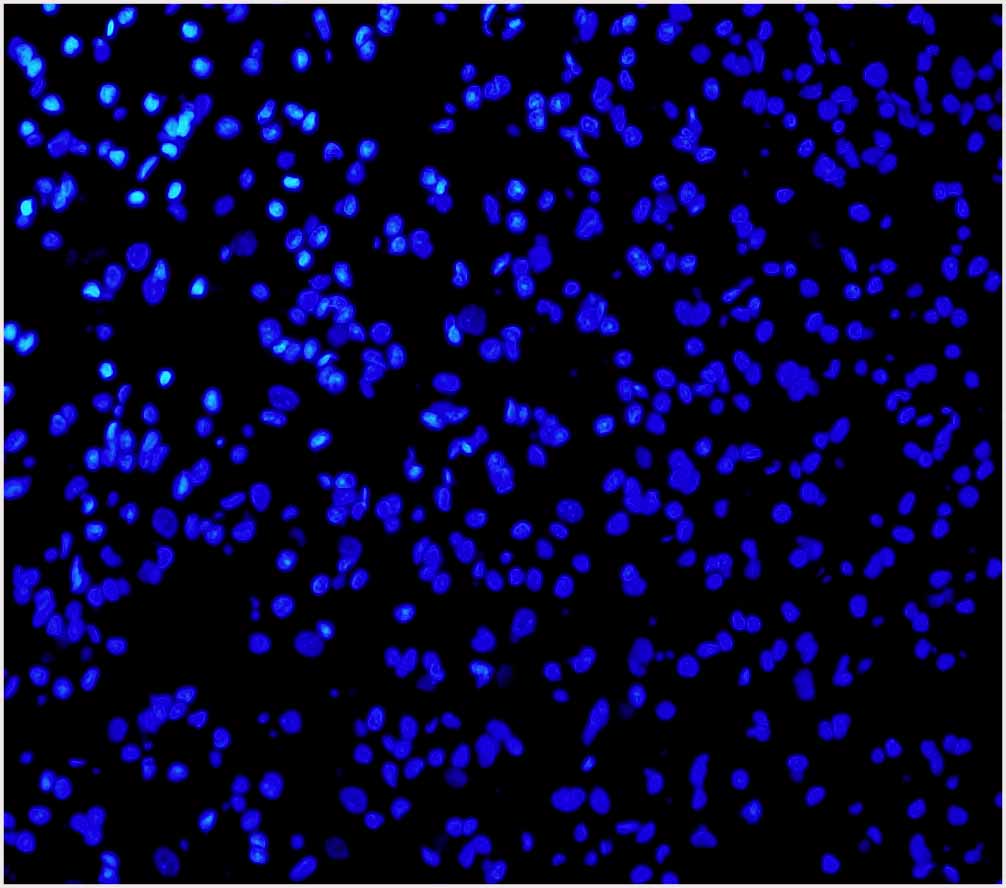

Supplement: Supplementary file 1 [file DataSheet1.ZIP › raw data/Fig.5/IF/SCI+Andro/3.jpg]

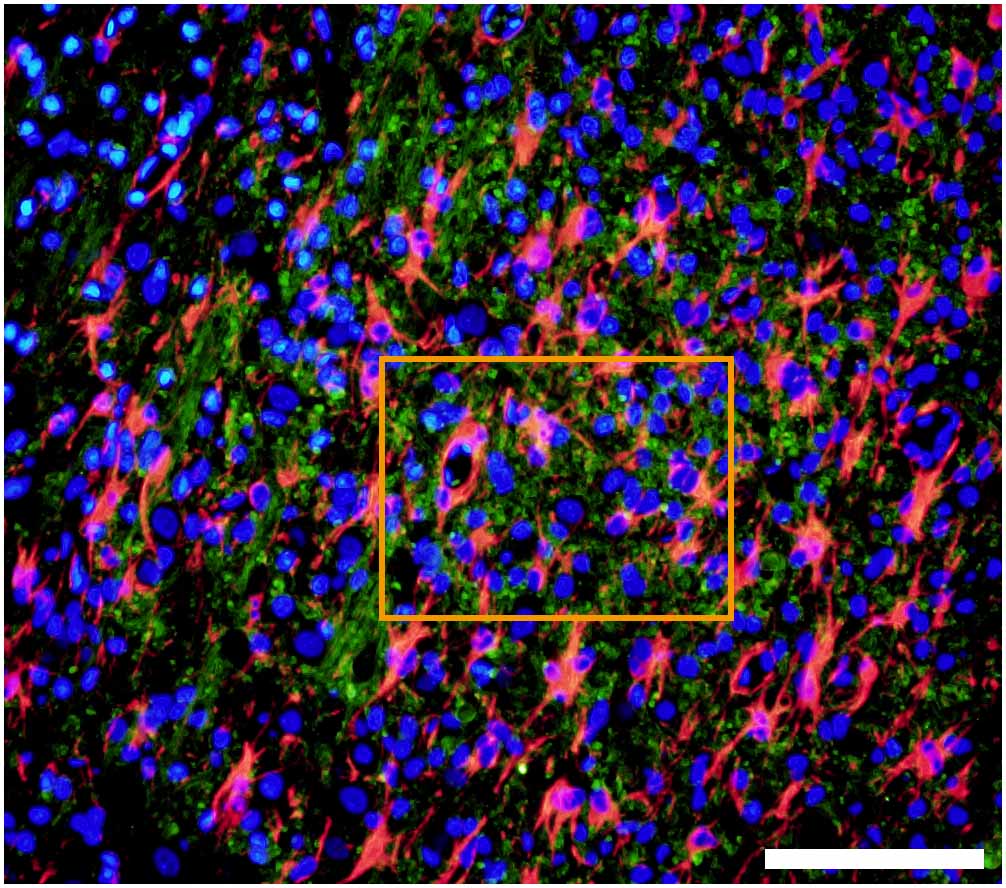

Supplement: Supplementary file 1 [file DataSheet1.ZIP › raw data/Fig.5/IF/SCI+Andro/4.jpg]

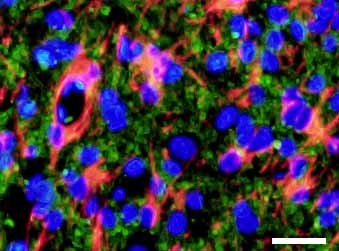

Supplement: Supplementary file 1 [file DataSheet1.ZIP › raw data/Fig.5/IF/SCI+Andro/5.jpg]

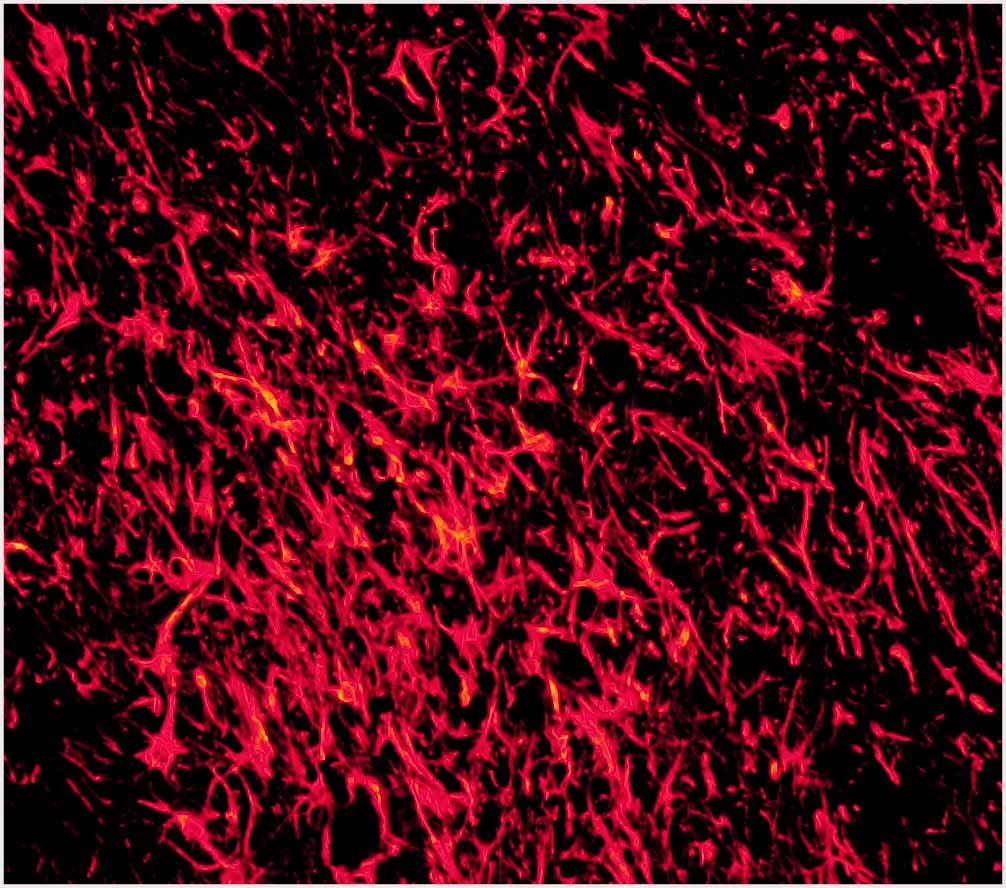

Supplement: Supplementary file 1 [file DataSheet1.ZIP › raw data/Fig.5/IF/SCI+NS/1.jpg]

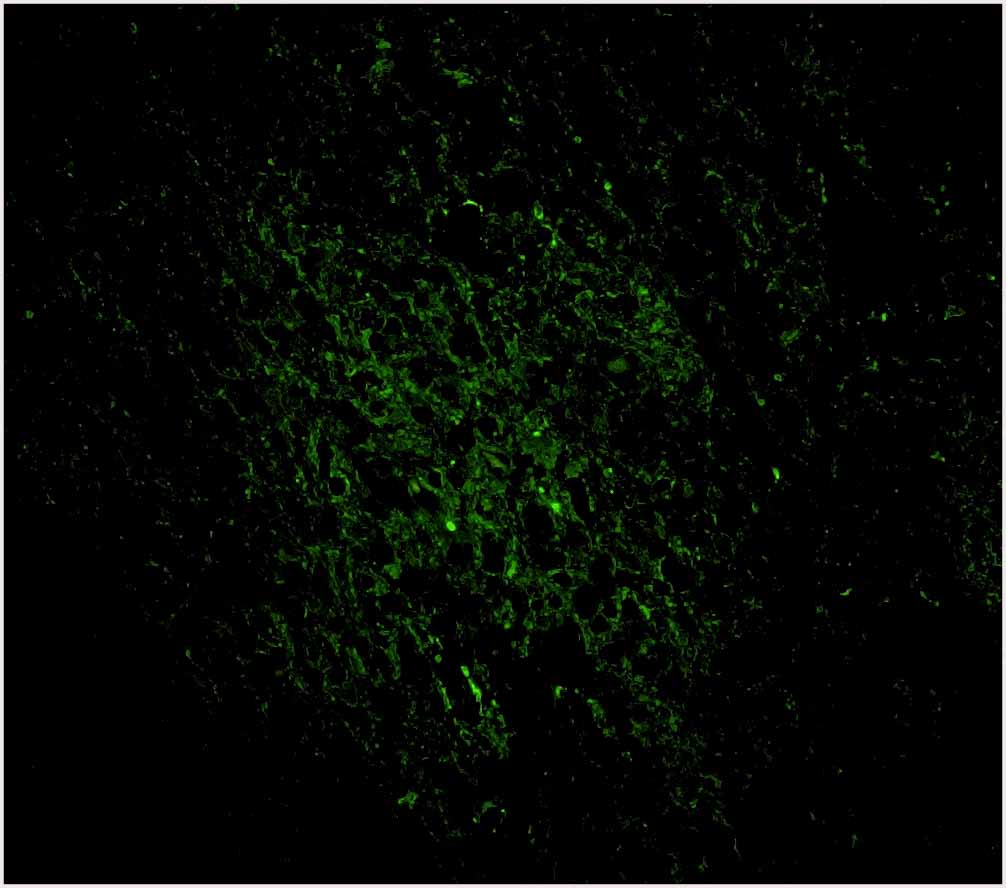

Supplement: Supplementary file 1 [file DataSheet1.ZIP › raw data/Fig.5/IF/SCI+NS/2.jpg]

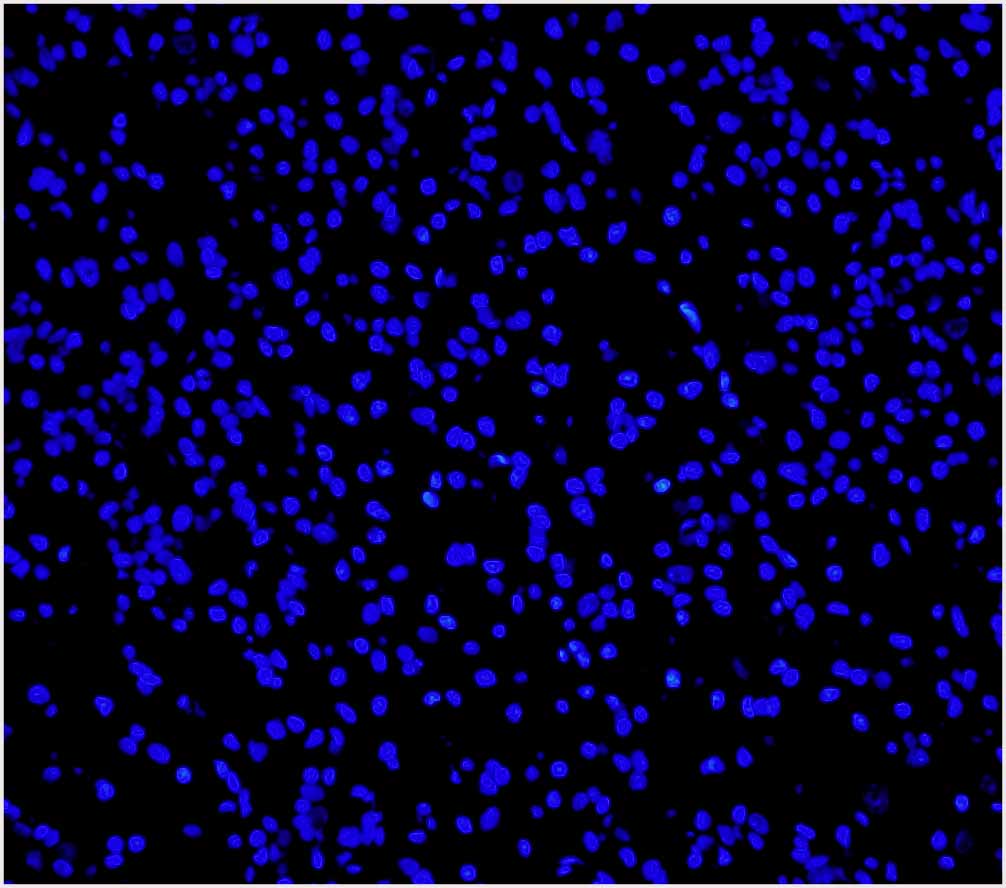

Supplement: Supplementary file 1 [file DataSheet1.ZIP › raw data/Fig.5/IF/SCI+NS/3.jpg]

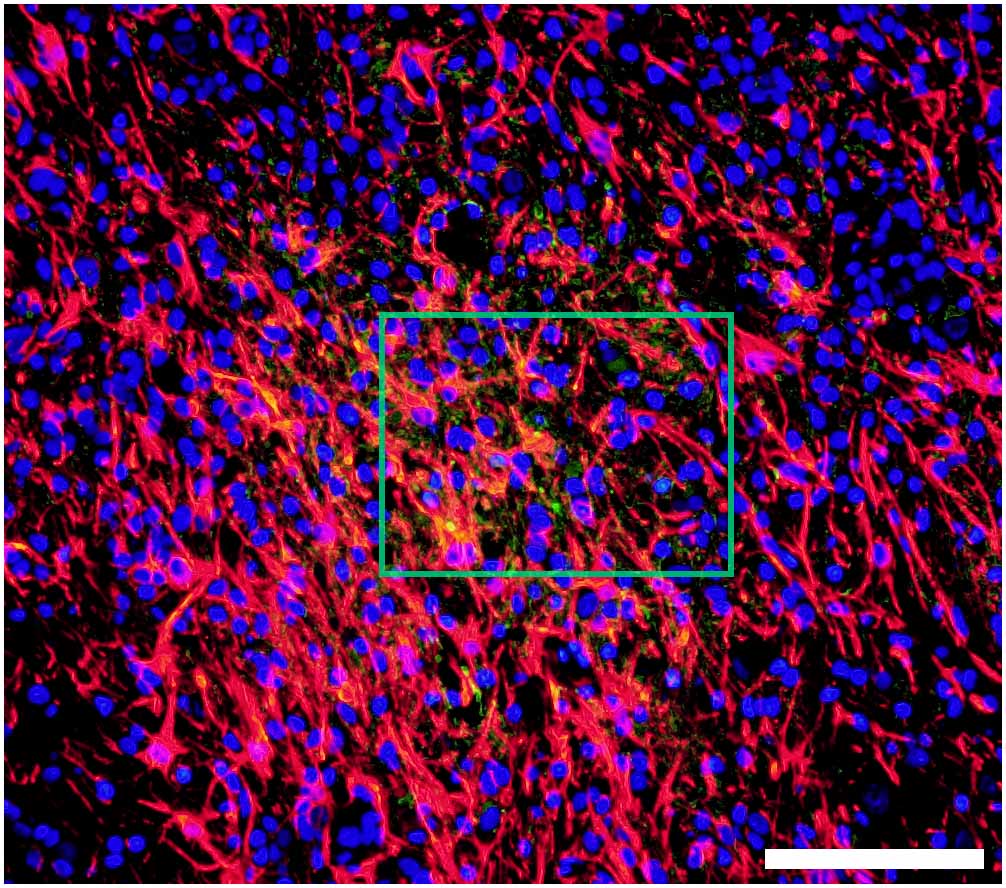

Supplement: Supplementary file 1 [file DataSheet1.ZIP › raw data/Fig.5/IF/SCI+NS/4.jpg]

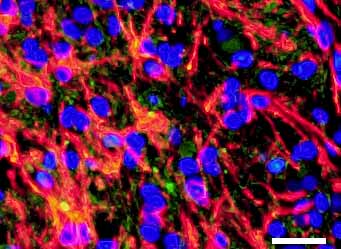

Supplement: Supplementary file 1 [file DataSheet1.ZIP › raw data/Fig.5/IF/SCI+NS/5.jpg]

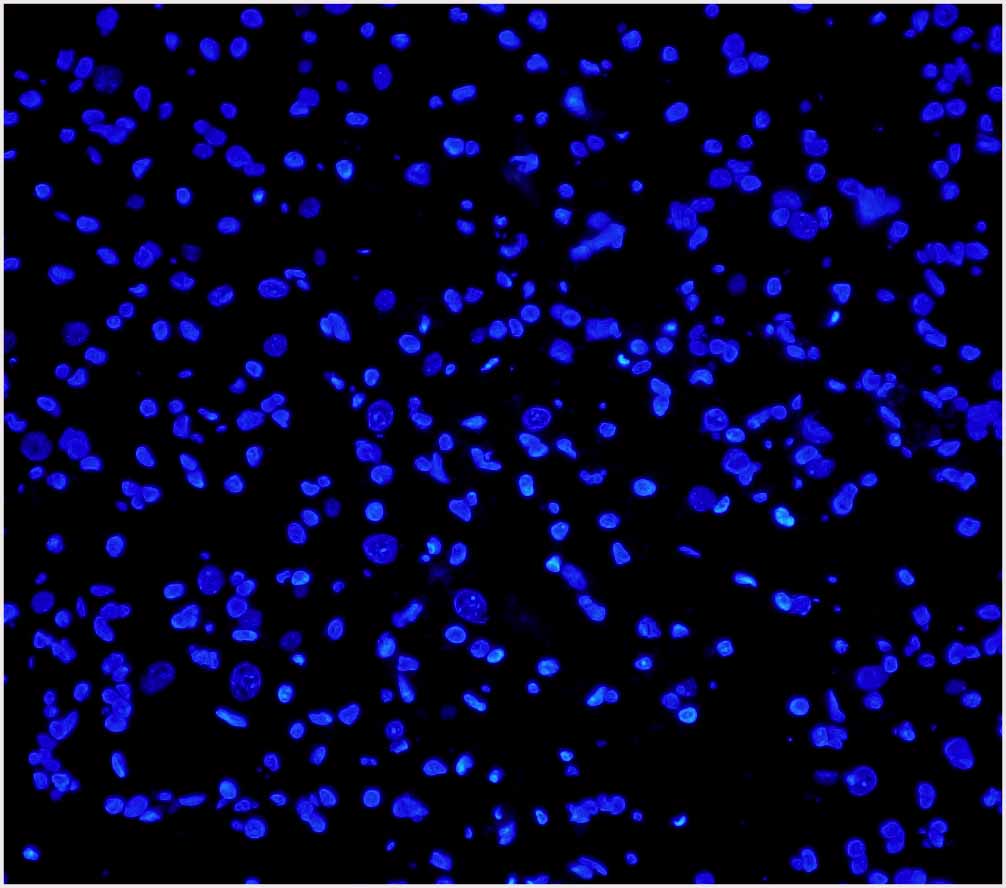

Supplement: Supplementary file 1 [file DataSheet1.ZIP › raw data/Fig.5/IF/SCI/0.jpg]

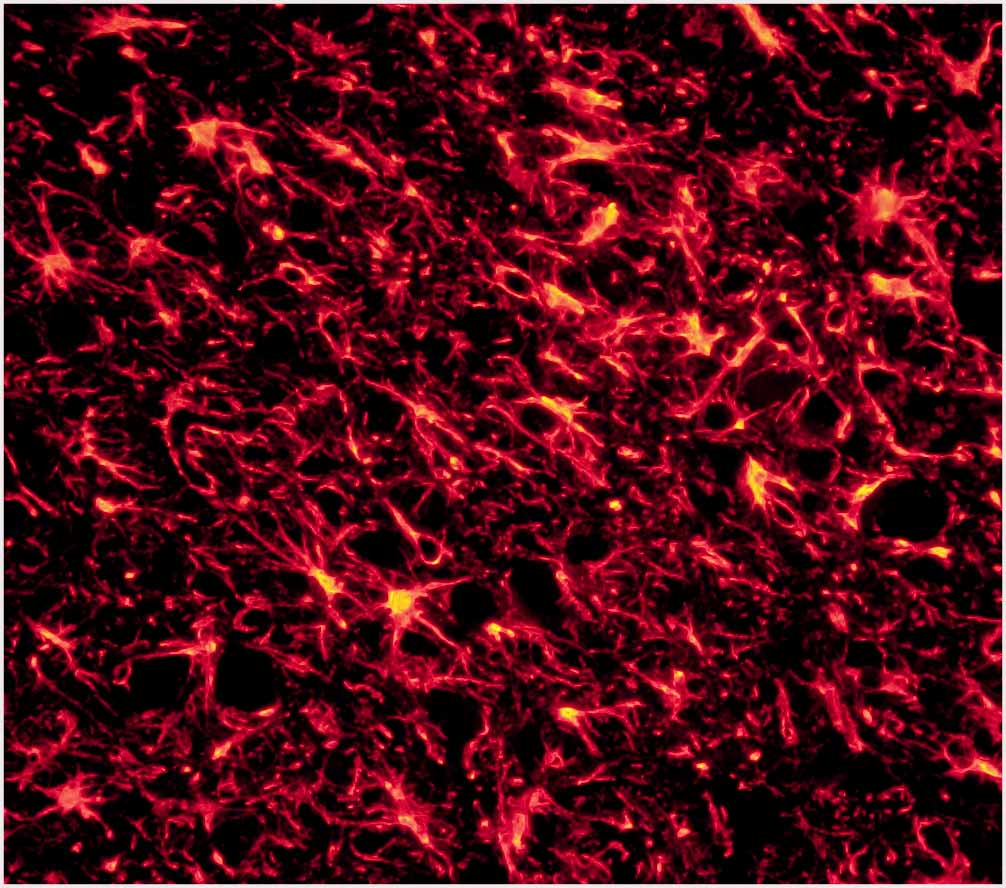

Supplement: Supplementary file 1 [file DataSheet1.ZIP › raw data/Fig.5/IF/SCI/1.jpg]

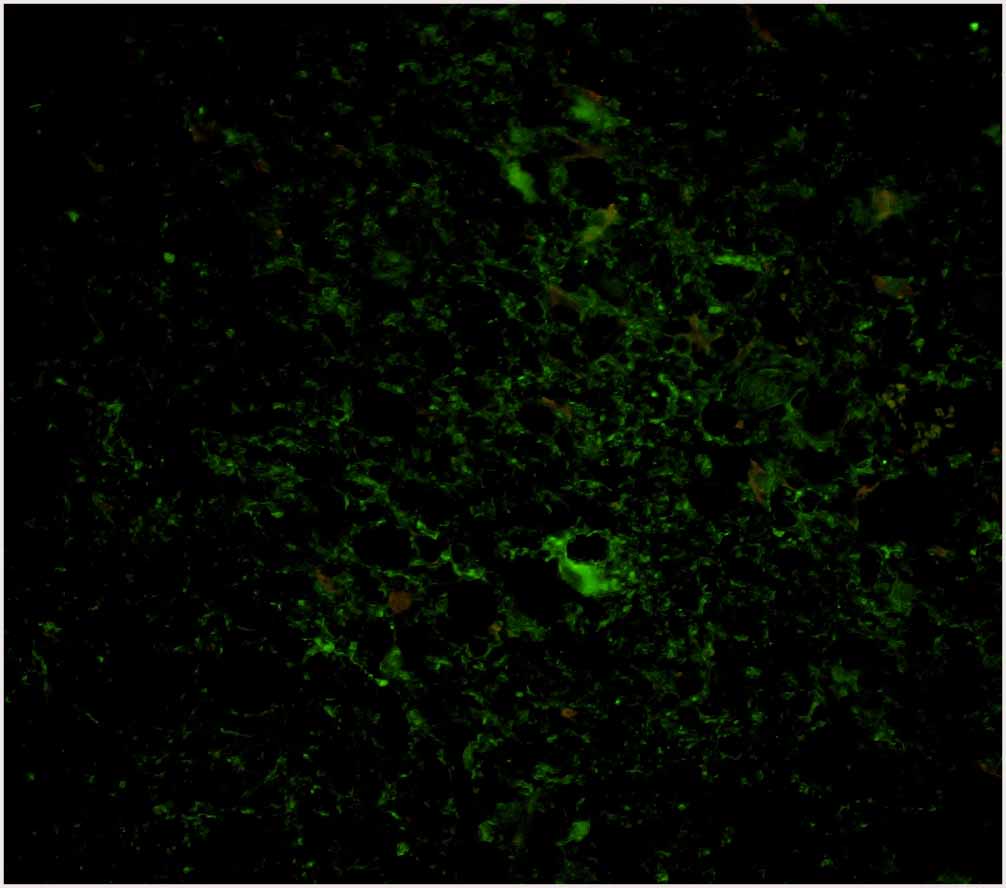

Supplement: Supplementary file 1 [file DataSheet1.ZIP › raw data/Fig.5/IF/SCI/2.jpg]

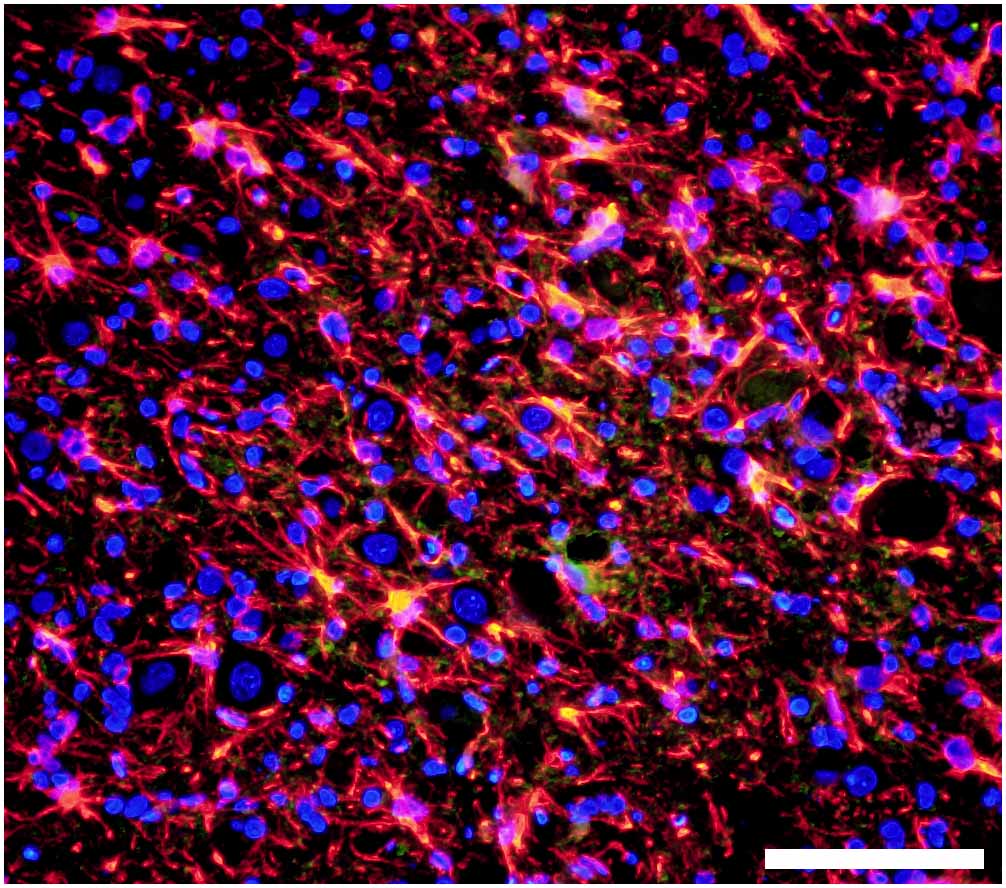

Supplement: Supplementary file 1 [file DataSheet1.ZIP › raw data/Fig.5/IF/SCI/3.jpg]

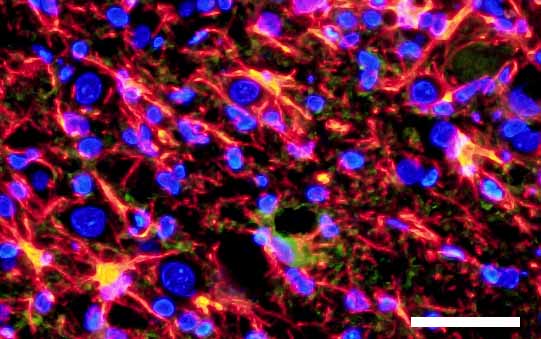

Supplement: Supplementary file 1 [file DataSheet1.ZIP › raw data/Fig.5/IF/SCI/║╧.jpg]

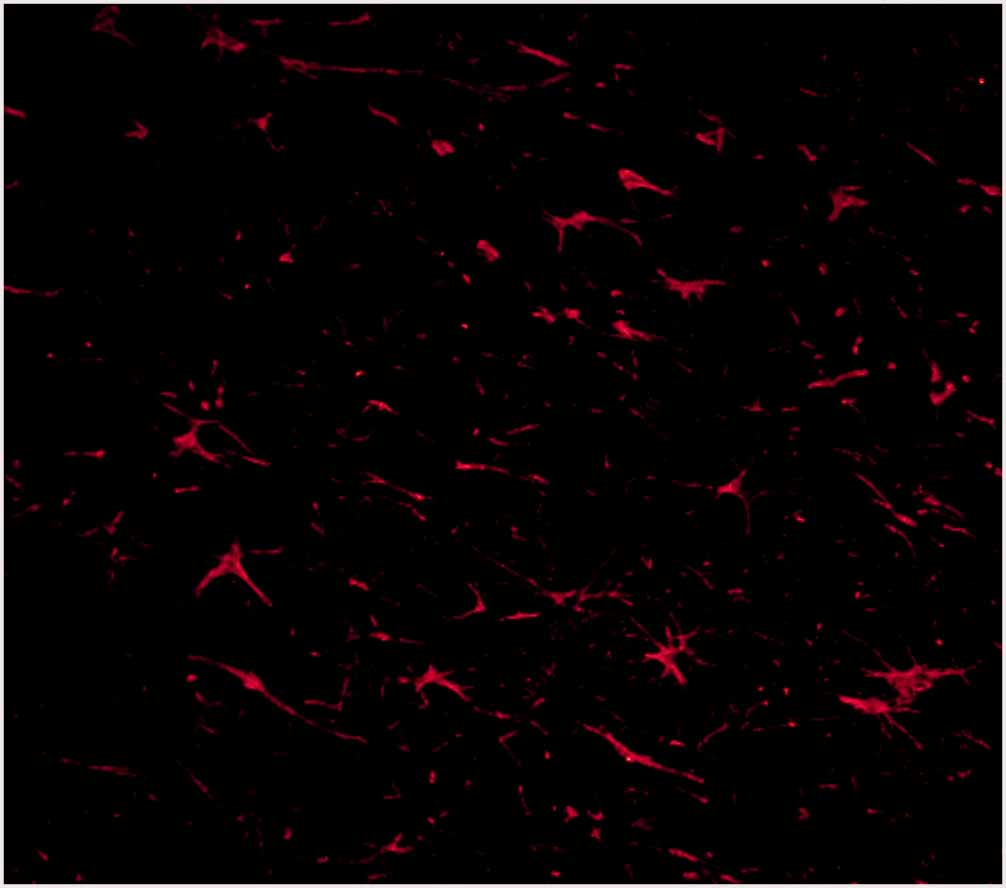

Supplement: Supplementary file 1 [file DataSheet1.ZIP › raw data/Fig.5/IF/Sham/1.jpg]

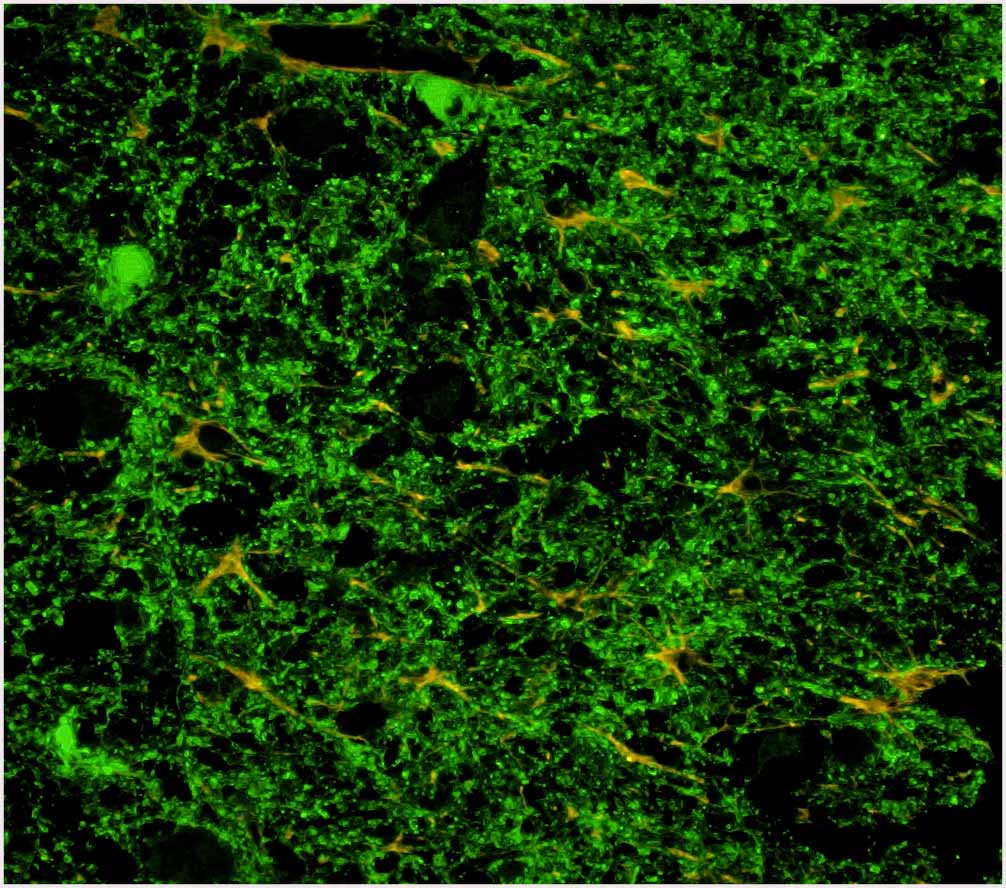

Supplement: Supplementary file 1 [file DataSheet1.ZIP › raw data/Fig.5/IF/Sham/2.jpg]

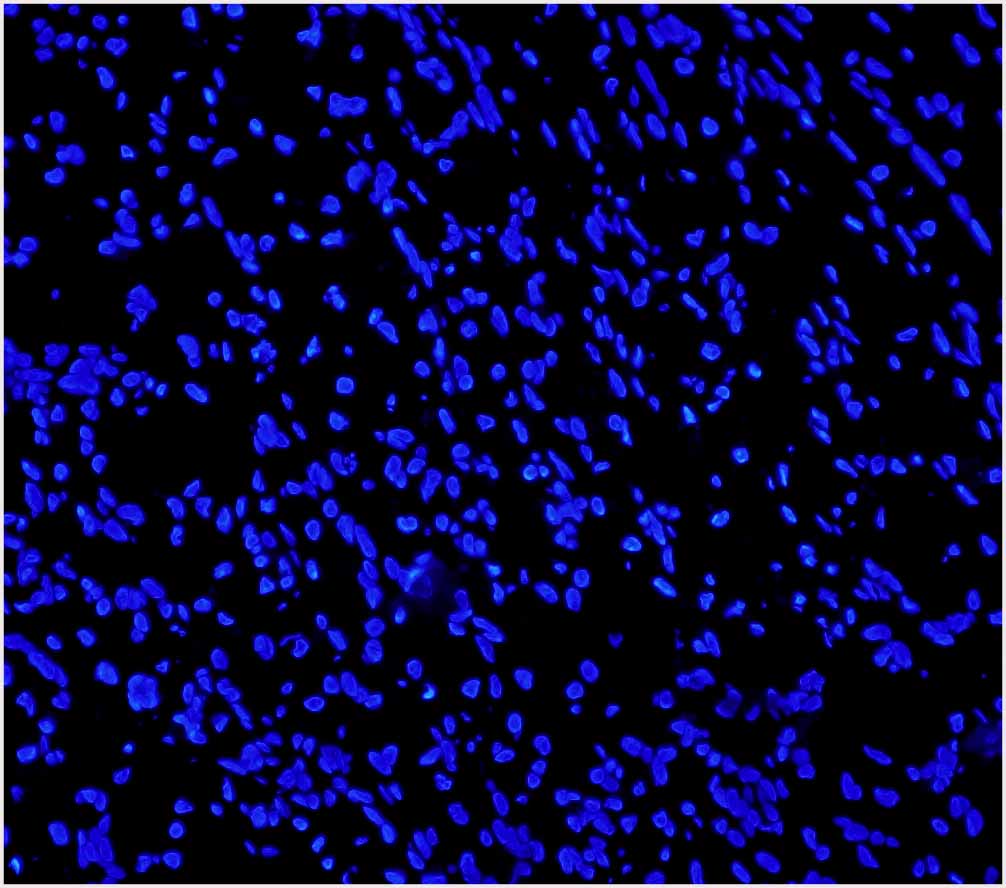

Supplement: Supplementary file 1 [file DataSheet1.ZIP › raw data/Fig.5/IF/Sham/3.jpg]

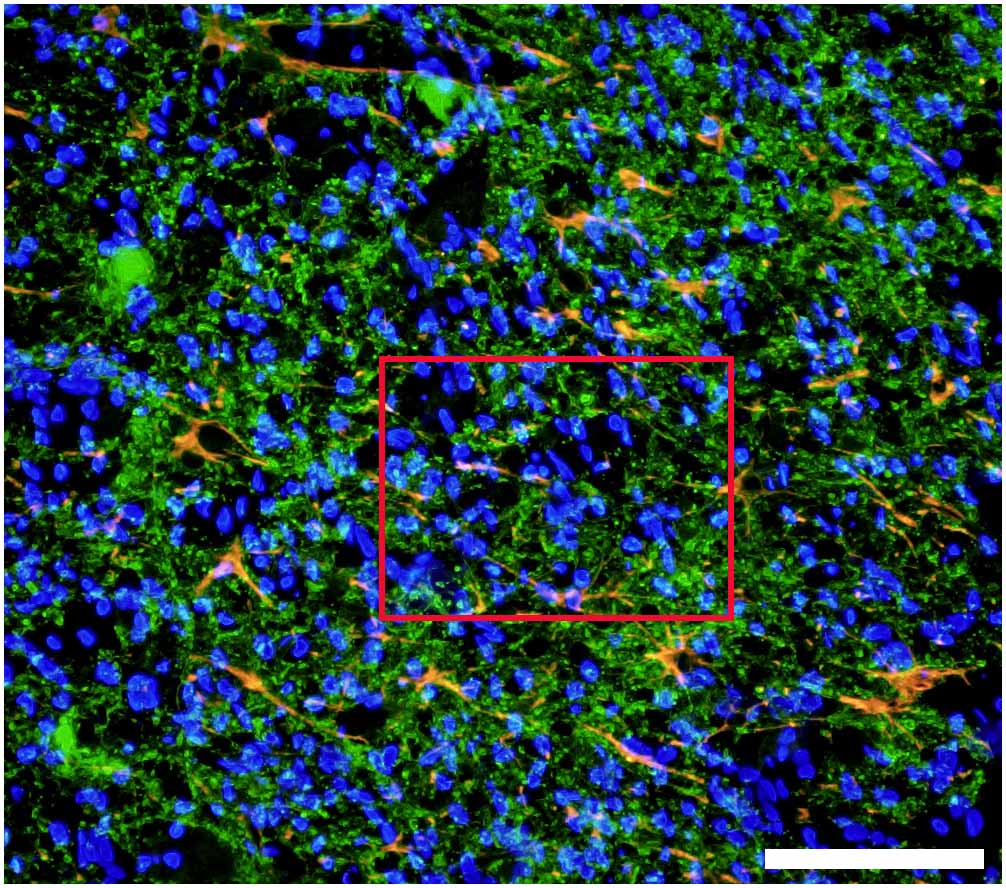

Supplement: Supplementary file 1 [file DataSheet1.ZIP › raw data/Fig.5/IF/Sham/4.jpg]

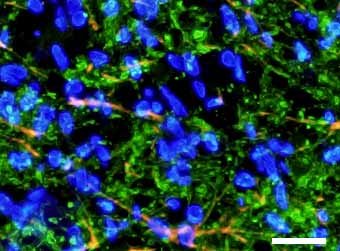

Supplement: Supplementary file 1 [file DataSheet1.ZIP › raw data/Fig.5/IF/Sham/5-.jpg]

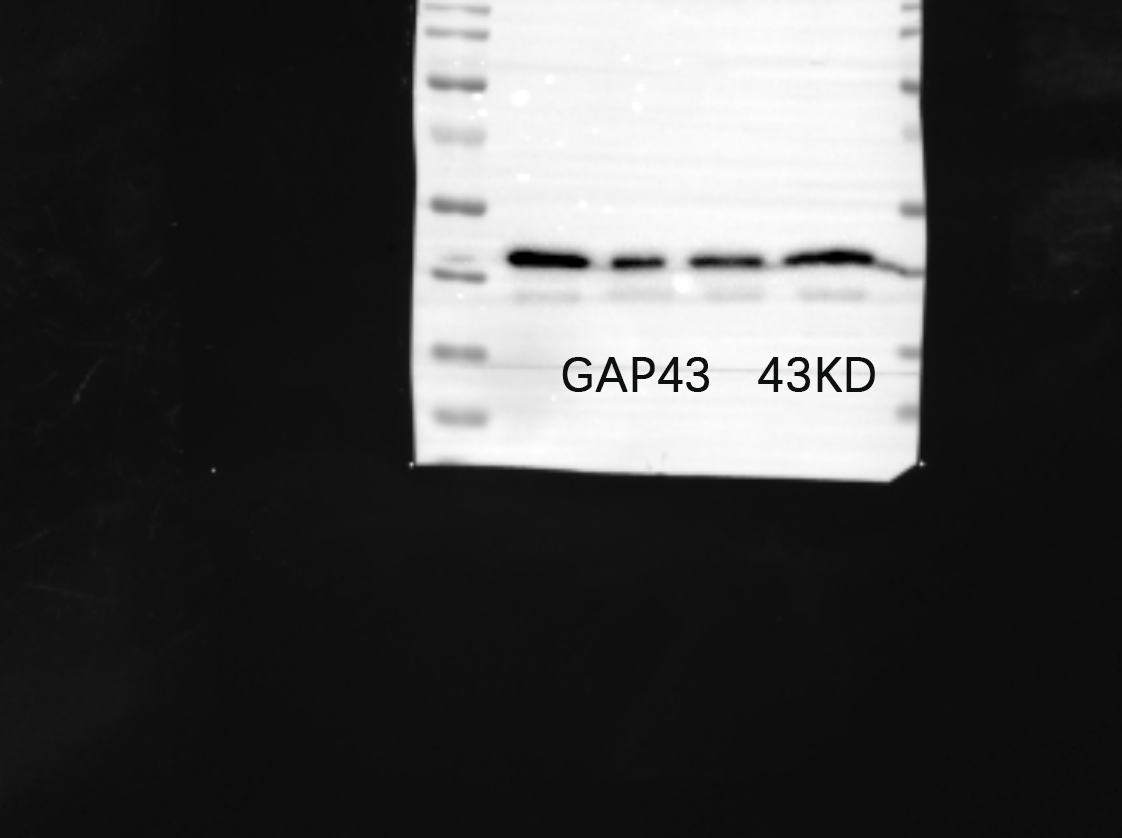

Supplement: Supplementary file 1 [file DataSheet1.ZIP › raw data/Fig.5/Western blot images/GAP43.tif]

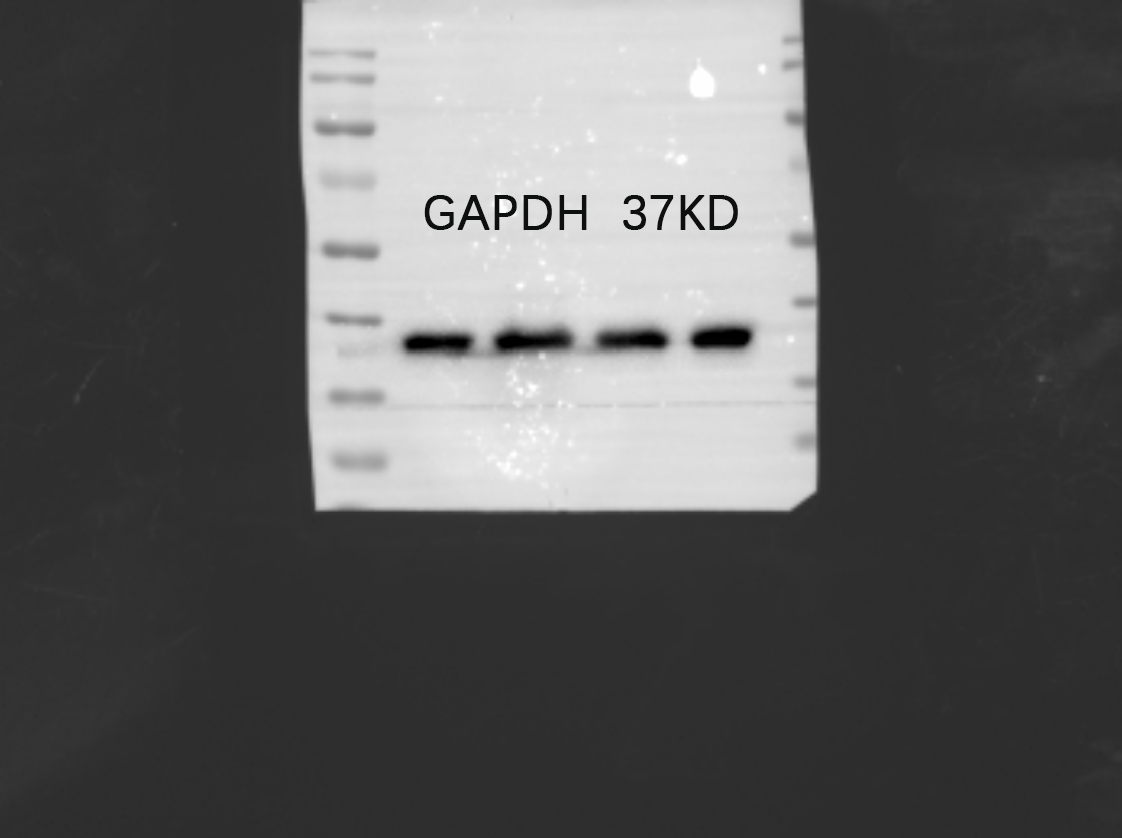

Supplement: Supplementary file 1 [file DataSheet1.ZIP › raw data/Fig.5/Western blot images/GAPDH.tif]

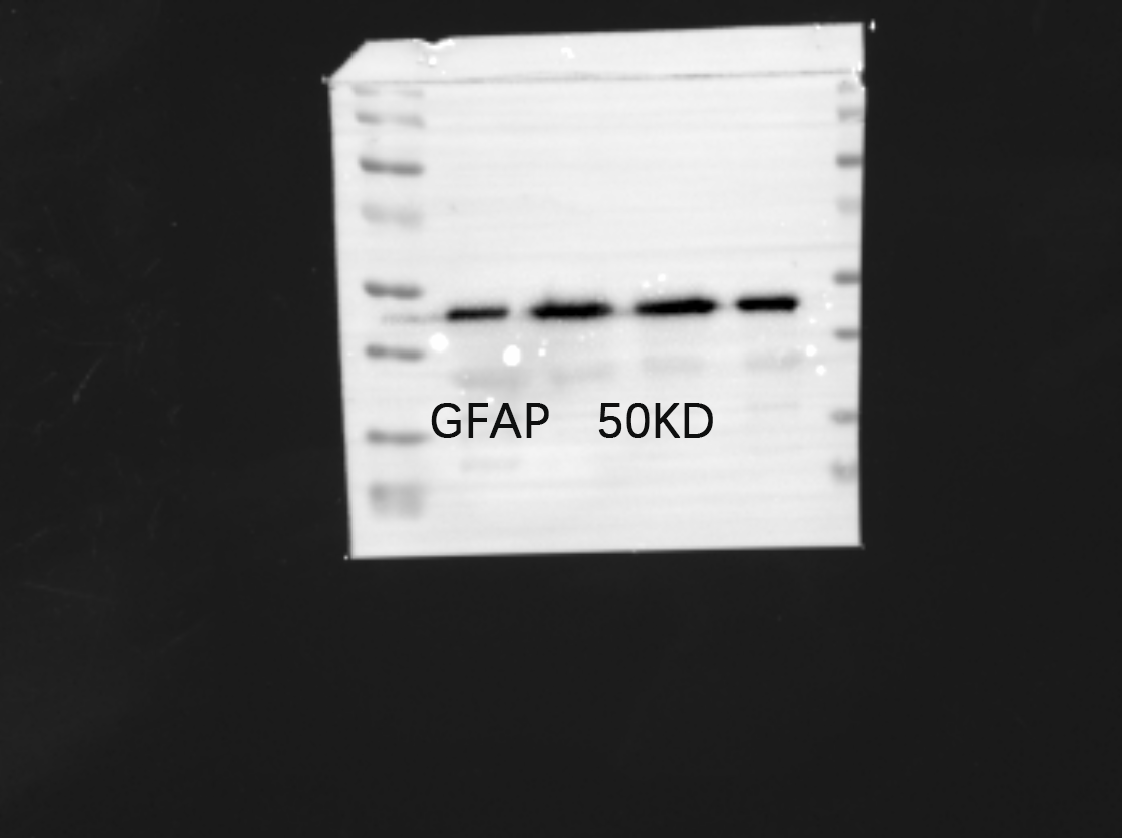

Supplement: Supplementary file 1 [file DataSheet1.ZIP › raw data/Fig.5/Western blot images/GFAP.tif]

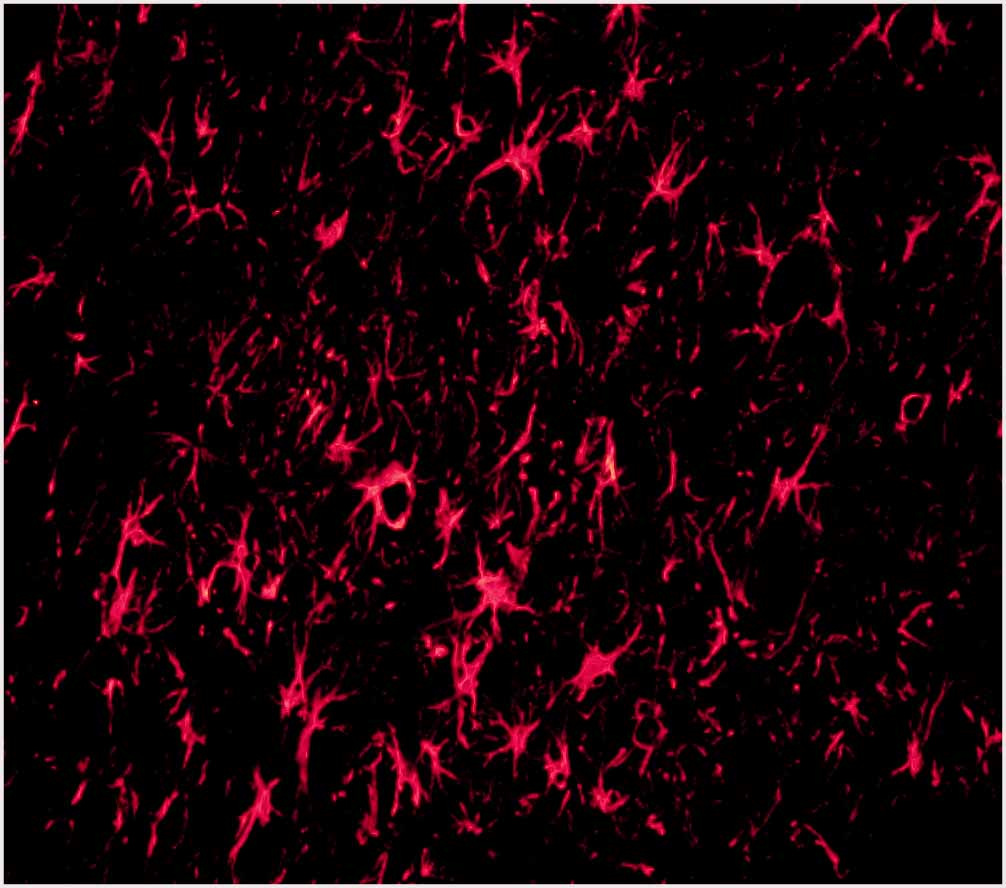

Supplement: Supplementary file 1 [file DataSheet1.ZIP › raw data/Fig.6/IF/SCI+Andro/1.jpg]

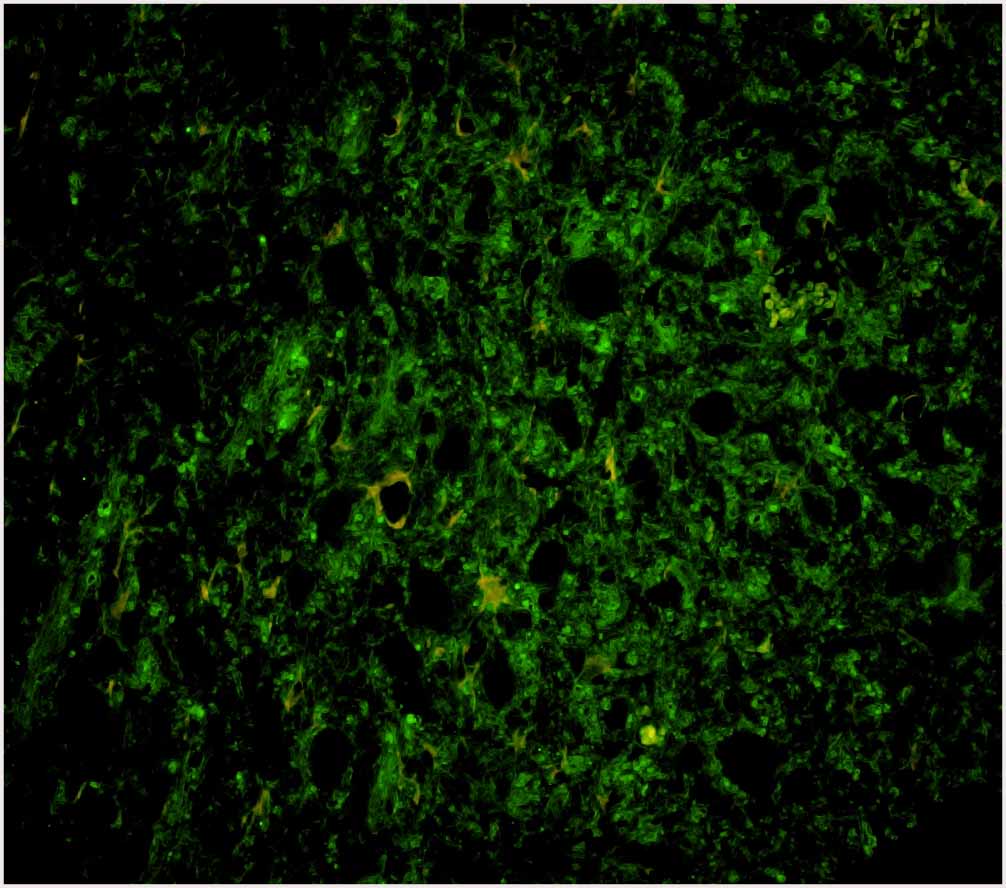

Supplement: Supplementary file 1 [file DataSheet1.ZIP › raw data/Fig.6/IF/SCI+Andro/2.jpg]

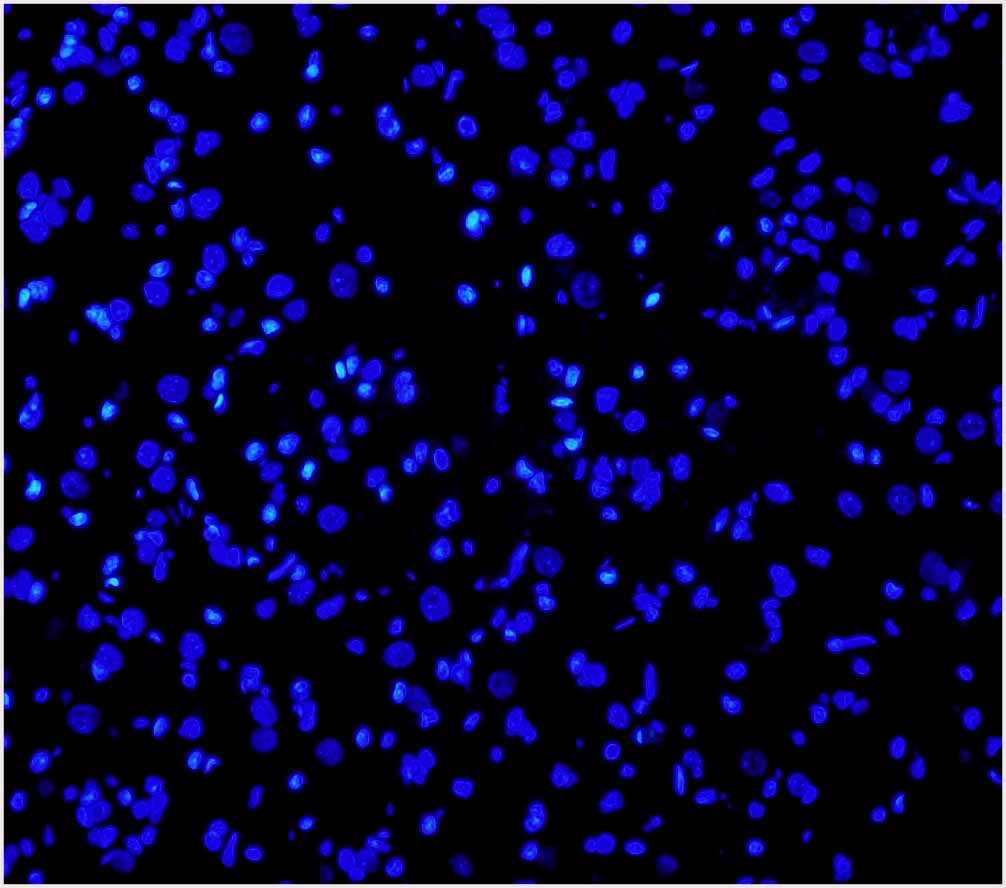

Supplement: Supplementary file 1 [file DataSheet1.ZIP › raw data/Fig.6/IF/SCI+Andro/3.jpg]

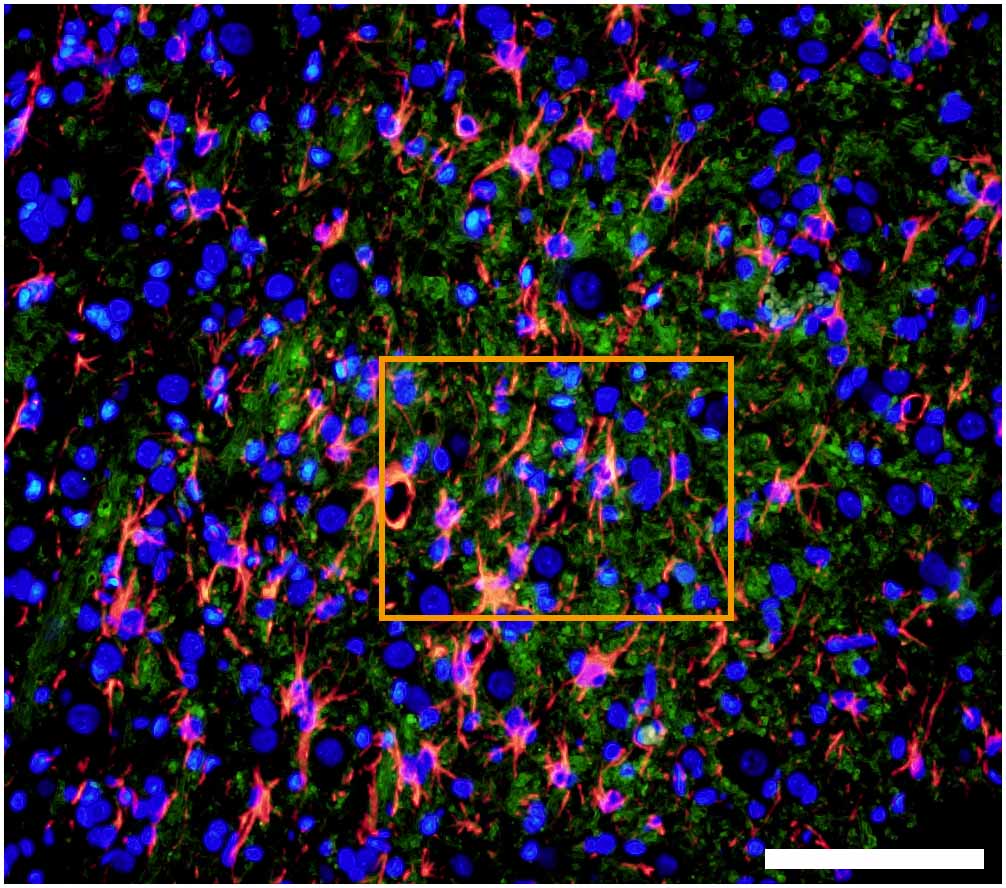

Supplement: Supplementary file 1 [file DataSheet1.ZIP › raw data/Fig.6/IF/SCI+Andro/4.jpg]

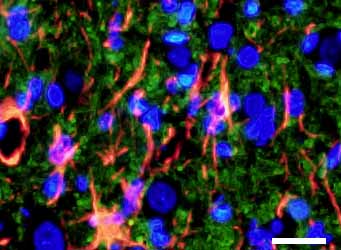

Supplement: Supplementary file 1 [file DataSheet1.ZIP › raw data/Fig.6/IF/SCI+Andro/5.jpg]
